# Novel Autotrophic Organisms Contribute Significantly to the Internal Carbon Cycling Potential of a Boreal Lake

**DOI:** 10.1128/mBio.00916-18

**Published:** 2018-08-14

**Authors:** Sari Peura, Moritz Buck, Sanni L. Aalto, Sergio E. Morales, Hannu Nykänen, Alexander Eiler

**Affiliations:** a Department of Ecology and Evolution, Limnology, Science for Life Laboratory, Uppsala University, Uppsala, Sweden; b Department of Biological and Environmental Science, University of Jyväskylä, Jyväskylä, Finland; c Department of Forest Mycology and Plant Pathology, Science for Life Laboratory, Swedish University of Agricultural Sciences, Uppsala, Sweden; d National Bioinformatics Infrastructure for Life Sciences, Uppsala University, Uppsala, Sweden; e Department of Microbiology and Immunology, University of Otago, Dunedin, New Zealand; Pacific Northwest National Laboratory

**Keywords:** chemoautotrophy, greenhouse gas emissions, iron oxidizers, metagenomics, redox gradient

## Abstract

Oxygen-stratified lakes are typical for the boreal zone and also a major source of greenhouse gas emissions in the region. Due to shallow light penetration, restricting the growth of phototrophic organisms, and large allochthonous organic carbon inputs from the catchment area, the lake metabolism is expected to be dominated by heterotrophic organisms. In this study, we test this assumption and show that the potential for autotrophic carbon fixation and internal carbon cycling is high throughout the water column. Further, we show that during the summer stratification carbon fixation can exceed respiration in a boreal lake even below the euphotic zone. Metagenome-assembled genomes and 16S profiling of a vertical transect of the lake revealed multiple organisms in an oxygen-depleted compartment belonging to novel or poorly characterized phyla. Many of these organisms were chemolithotrophic, potentially deriving their energy from reactions related to sulfur, iron, and nitrogen transformations. The community, as well as the functions, was stratified along the redox gradient. The autotrophic potential in the lake metagenome below the oxygenic zone was high, pointing toward a need for revising our concepts of internal carbon cycling in boreal lakes. Further, the importance of chemolithoautotrophy for the internal carbon cycling suggests that many predicted climate change-associated fluctuations in the physical properties of the lake, such as altered mixing patterns, likely have consequences for the whole-lake metabolism even beyond the impact to the phototrophic community.

## IMPORTANCE

Autotrophic organisms at the base of the food web are the only life form capable of turning inorganic carbon into the organic form, facilitating the survival of all other organisms. In certain environments, the autotrophic production is limited by environmental conditions and the food web is supported by external carbon inputs. One such environment is stratified boreal lakes, which are one of the biggest natural sources of greenhouse gas emissions in the boreal region. Thus, carbon cycling in these habitats is of utmost importance for the future climate. Here, we demonstrate a high potential for internal carbon cycling via phototrophic and novel chemolithotrophic organisms in the anoxic, poorly illuminated layers of a boreal lake. Our results significantly increase our knowledge on the microbial communities and their metabolic potential in oxygen-depleted freshwaters and help to understand and predict how climate change-induced alterations could impact the lake carbon dynamics.

## INTRODUCTION

All life on Earth depends on carbon fixation, where phototrophic organisms convert inorganic carbon dioxide into organic compounds and living biomass. Currently, oxygenic phototrophs, deriving their energy from light, are regarded as the most important carbon fixers. However, on planetary time scales anoxygenic phototrophs and chemotrophs have been more prevalent (1). Even today, chemolithoautotrophy is a major strategy in many environments, such as deep-sea vents and sediments (2–4). In these environments, carbon fixation is driven by a redox gradient (i.e., a biogeochemical gradient of reductants and oxidants sorted by their redox potentials) often located in the border zone between oxic and anoxic conditions. These redox transition zones constitute a major share of the Earth’s biosphere and have a significant impact on surrounding entities, such as elemental cycles and food webs (5–7).

Chemolithotrophy may have a large impact on the carbon cycle in many environments. For example, in boreal lakes there is a steep redox gradient at the oxic-anoxic border, which could facilitate carbon fixation via chemolithotrophy. Still, our knowledge on the chemolithotrophic energy generation in these habitats is poor. Some first studies highlighted the importance of chemolithotrophy for carbon assimilation in freshwater lakes such as Kivu, a freshwater lake in central Africa with a high methane (CH_4_) concentration (2). Furthermore, two recent studies confirmed the genetic potential for autotrophy in microbial communities in the anoxic water masses of boreal lakes (8, 9). This suggests that chemolithotrophy could be a common process fueling assimilation of inorganic carbon in boreal lakes. Thus, dark carbon fixation may play a significant role in internal carbon cycling and, thus, could modulate lake food webs and ultimately whole-lake carbon balances.

Small boreal lakes are important drivers of global greenhouse gas (GHG) emissions (10, 11), and their importance was highlighted in the recent report of the Intergovernmental Panel on Climate Change (IPCC) (12). Globally, water bodies smaller than 0.001 km^2^ contribute 40% of all methane emissions from inland waters (13). Typically, these small lakes and ponds are characterized by high concentrations of dissolved organic carbon (DOC) and shallow light penetration depth, which leads to steep stratification of oxygen and other electron acceptors and donors through most of the year. The stratification coincides with a distinct set of bacterial and archaeal phyla organized according to a vertical redox gradient (9, 14, 15). The microbial communities in these lakes may harbor organisms that have the potential for photoautotrophy under low light intensity (14, 16, 17) and for chemoautotrophy throughout the water column (9). These predictions are based on taxonomic information derived from 16S rRNA genes combined with functional gene inventories and genomic data of related cultivated representatives. Therefore, they are not a true reconstruction of the genetic makeup of individual organisms or whole communities along the redox tower. This lack of a detailed metabolic picture limits our understanding of the functional potential of microbes in boreal lakes.

We studied the potential of the microbial community for chemoautotrophy in the lake Alinen Mustajärvi, a well-characterized boreal lake located in southern Finland (15, 18). This lake exhibits the typical characteristic features of boreal lakes, including (i) a high load of terrestrial organic carbon resulting in net heterotrophy of the system (18); (ii) a gradient of oxygen, temperature, and light; and (iii) stratification of the microbial community (9, 14, 15). We combined dark carbon fixation measurements with a survey of the functional potential of the microbial community (shotgun sequencing of the total DNA) from a vertical transect of the lake water column. Our aim was to link lake chemistry to the prevalence of genes related to energy generation via redox reactions and inorganic carbon assimilation. Moreover, we used annotated metagenome-assembled genomes (MAGs) to obtain metabolic reconstructions of uncharacterized and novel lake microbes and to identify the key chemoautotrophs in the lake. Our hypotheses were that (i) the chemical stratification of the lake was linked to microbial activity and would be reflected in the functional potential and structure of the communities, (ii) the lake harbors an abundant and diverse chemoautotrophic community, and (iii) chemoautotrophic pathways would be enriched below the euphotic zone of the lake, leading to a high potential for internal carbon cycling.

## RESULTS AND DISCUSSION

### Microbial community structure and CO_2_ incorporation in the water column.

**FIG 1:**
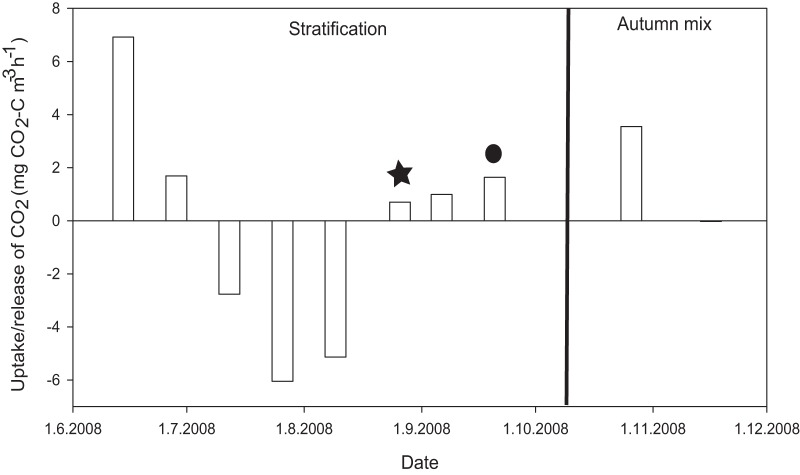
Change in CO_2_ concentration over 24-h *in situ* dark incubations during open water season 2008 at a depth of 3 m. Positive values indicate respiration dominating over CO_2_ assimilation, while negative values show net incorporation of inorganic carbon into biomass. The star depicts the time point at which the clone library was retrieved in 2008, and the circle shows the time point when the amplicon and shotgun samples were taken in 2013.

**FIG 2:**
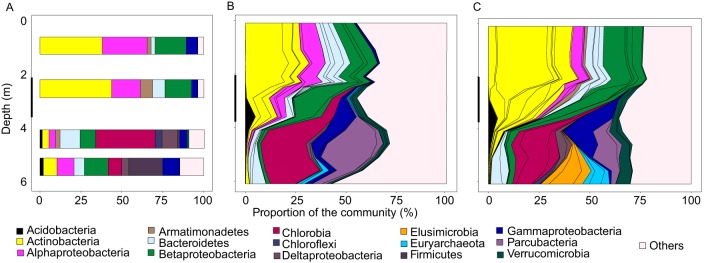
Microbial community composition in the lake Alinen Mustajärvi based on 16S rRNA genes in 2008 (a clone library) (A), 16S rRNA genes in 2013 (Illumina MiSeq) (B), and metagenome-assembled genomes from 2013 (Illumina HiSeq) (C). In panels B and C, different operational taxonomic units (OTUs) of the same phylum are separated by black lines. The black bars on the *y* axes show the location of the oxygen depletion zone.

The metabolic pathways and organisms that could be involved in autotrophic processes in the water column of the lake Alinen Mustajärvi were studied based on molecular (metagenomic shotgun sequencing and 16S rRNA gene amplicons) and geochemical analyses from samples taken in 2013. This vertical transect of the lake covered 13 depths. Additionally, the community composition was surveyed in 2008 using a clone library covering 4 depths and 303 clones of 16S rRNA genes. We also measured inorganic carbon dynamics biweekly in 2008, monthly in 2009, and on 3 occasions in 2010. The carbon measurements indicated that at the 3-m depth the incorporation of inorganic carbon dominated over respiration during parts of the ice-free season (Fig. 1; see also Fig. S1 in the supplemental material). Time of net autotrophy coincided with stratification of the lake and vertical structuring of the microbial community along the vertical gradient (Fig. 2A). The maximum value for dark carbon assimilation at the 3-m depth was 10% of the average net primary production (carbon assimilation in the light) within the euphotic zone (Fig. 1) (18). It should be taken into account that these are point measurements and could be highly time and depth specific. However, they are well in line with dark carbon fixation values measured from lake sediments (19), which share many of the same physicochemical characteristics with the overlying water, such as a gradient of electron donors and acceptors suitable for dark carbon fixation.

10.1128/mBio.00916-18.1 FIG S1  Change in dissolved inorganic carbon concentration during 24-h dark incubations at a 3-m depth in 2008 to 2010. In 2008, measurements were done biweekly; in 2009, they were done monthly; and in 2010, measurements were made three times during the open water season. The autumn mixing period is marked with red boxes. Download FIG S1, PDF file, 0.02 MB. Copyright © 2018 Peura et al. 2018 Peura et al. This content is distributed under the terms of the Creative Commons Attribution 4.0 International license.

**FIG 3:**
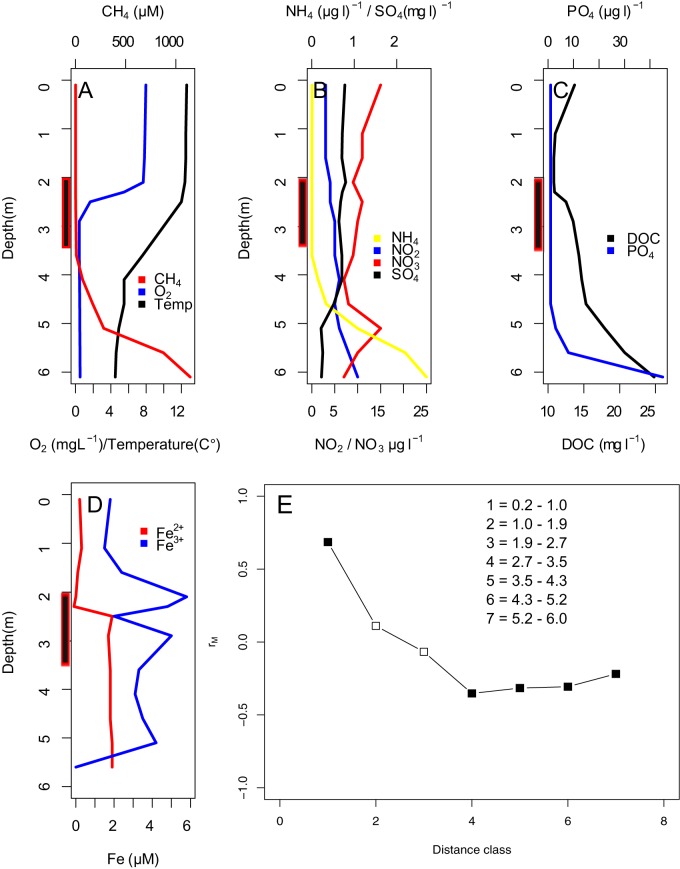
Environmental conditions in the lake in 2013. (A) Concentrations of O_2_ and CH_4_ and water temperature. (B to D) Concentration of NH_4_, NO_2_, NO_3_, and SO_4_ (B); PO_4_ and DOC (C); and Fe^2+^ and Fe^3+^ (D). (E) Bonferroni-corrected Pearson correlations of the community composition (measured as Bray-Curtis distances) of the samples according to sampling distance. Black symbols designate significant correlations with *P* values of <0.005 to all except distance 4, for which *P* is 0.006. The thick bars on the *y* axes of panels A to D illustrate the oxygen depletion zone.

The analysis of 16S rRNA genes indicated that the communities were dominated by similar phyla in 2008 and 2013 (Fig. 2A and B) and that community composition was consistent with previously published profiles from the same lake (15). The water column exhibited a physicochemical stratification with water temperature, oxygen (O_2_), and sulfate (SO_4_) concentrations decreasing with depth, whereas the concentrations of CH_4_, DOC, ammonia (NH_4_), nitrite (NO_2_), and phosphate (PO_4_) were highest at the lake bottom (Fig. 3A to D). The microbial community was stratified into distinct layers with positive correlation between community composition in samples taken within a 1-m distance of each other and negative correlations among samples taken more than 2.6 m apart (Fig. 3E). While the bacterial community was homogenous throughout the epilimnion, below there was a sharp change coinciding with the decrease in oxygen concentration in the metalimnion and a subsequent succession of microbial taxa in the hypolimnion.

### Stratification of microbial functional potential follows the redox gradient.

The functional potential of the microbial community was estimated based on shotgun sequencing of the total DNA. Sequence coverage among samples (i.e., the proportion of reads from each of the samples that could be mapped to contigs) varied from 40.2% at the bottom to 75.5% in the surface (Table 1). The variation in the coverage is a result of an increase in diversity toward the lake bottom; thus, the sequences in the hypolimnion samples were divided across a higher number of distinct genomes, resulting in only the most abundant ones having enough sequencing coverage to be assembled. Thus, our functional analysis may be missing some of the pathways present in the lake but is expected to reflect the dominant metabolic potential in the water column. The abundances of different genes were normalized sample-wise using the abundance of 139 single-copy genes (20). With this approach, the abundance of the markers is expressed as occurrences per genome equivalent (OGE), e.g., a value of 1 suggests the presence of the gene in every genome of the sample. The limitations of the approach are that (i) in the deeper layers a high proportion of the community was composed of members of candidate phyla that are known to lack some of the single-copy genes (21) and (ii) some of the single-copy genes might not be recognized in our screening. Thus, especially in the deeper layers we might underestimate the true genome abundances, leading to overestimation of OGE. It should also be noted that the values suggesting abundances of 1 and above could be explained by the presence of multiple gene copies in the genomes harboring these markers or, as stated above, reflect the impact of underestimated total number of genomes.

**TABLE 1:** Sampling depth, water layer, data size, assembly coverage in each sample, and inverse Simpson index and Pielou’s evenness of the 16S rRNA OTU_0_._03_ data

Depth (m)	Layer	Raw data (Gb)	Coverage (%)	Inverse Simpson index	Pielou’s evenness
0.1	Epilimnion	8.06	61.86	9.90	0.49
1.1	Epilimnion	10.75	73.54	13.39	0.52
1.6	Epilimnion	9.54	75.49	12.01	0.50
2.1	Epilimnion	10.55	72.24	11.52	0.50
2.3	Epilimnion	10.24	73.33	12.50	0.51
2.5	Metalimnion	9.75	67.29	19.76	0.61
2.9	Metalimnion	9.68	61.09	22.28	0.62
3.6	Hypolimnion	10.52	65.57	28.52	0.62
4.1	Hypolimnion	10.51	63.62	9.69	0.53
4.6	Hypolimnion	9.26	60.39	8.41	0.51
5.1	Hypolimnion	8.31	48.72	8.39	0.53
5.6	Hypolimnion	8.05	43.77	11.50	0.60
6.1	Hypolimnion	5.24	40.21	35.97	0.69

**FIG 4:**
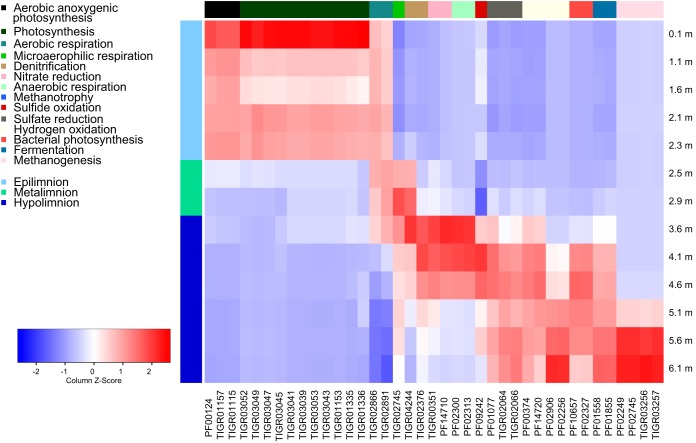
Heat map visualizing the abundances of Pfam/TIGRFAM markers related to energy metabolism (as given in *Z*-score standardized per genome equivalent). Only those Pfams/TIGRFAMs that had significantly different abundances between different layers of the lake are displayed. Colors at the top of each of the columns reflect the function that the marker represents, and colors on the left side of the heat map illustrate different layers of the lake.

Markers indicative of phototrophy could be found throughout the water column (Fig. 4). The genes encoding oxygen-evolving photosystem II and aerobic anoxygenic photosynthesis (AAP) decreased rapidly with depth, with combined abundance of these pathways at the surface being 1 OGE. Despite the shallow light penetration depth (Fig. S2), a second peak in photosynthetic potential was observed in the hypolimnion, where the abundance of the marker for anaerobic anoxygenic photosynthesis (AnAP; bacterial photosynthesis) was up to 0.32 OGE (Fig. S3).

10.1128/mBio.00916-18.2 FIG S2  Photosynthetically active radiation in the lake water column. Download FIG S2, PDF file, 0.01 MB. Copyright © 2018 Peura et al. 2018 Peura et al. This content is distributed under the terms of the Creative Commons Attribution 4.0 International license.

10.1128/mBio.00916-18.3 FIG S3  Average abundance of marker genes for different pathways related to energy generation. Download FIG S3, PDF file, 0.02 MB. Copyright © 2018 Peura et al. 2018 Peura et al. This content is distributed under the terms of the Creative Commons Attribution 4.0 International license.

In the hypolimnion, there were multiple potential pathways for energy generation, some of which were even more abundant than AnAP. One of these was hydrogen oxidation, for which the potential increased toward the lake bottom with the nickel-dependent hydrogenase being more abundant right below the metalimnion and the iron-dependent hydrogenase being more abundant at the lake bottom (Fig. 4), where the abundance of hydrogenases was close to 1 OGE. The relative importance of these different pathways for the actual energy metabolism of the community depends on the activity of the organisms, which we cannot judge based on their genetic profiles. An iron gradient in the water column suggested that redox reactions related to iron could be important pathways for energy acquisition (Fig. 3D), and putative iron oxidizers (order *Ferrovales*) were abundant in the metalimnion. However, there were no specific markers for this pathway; thus, while we have support for the potential for using iron as an energy source, we cannot visualize the trend for this pathway in the water column.

Based on their concentrations, the most important inorganic electron donors in the water column appeared to be CH_4_ in the upper hypolimnion and sulfide-sulfur, which spanned almost throughout the hypolimnion. The peak of the marker indicative for sulfide-sulfur oxidation coincided with the maxima in phototrophic sulfur-oxidizing *Chlorobium*, which were major community members in the hypolimnion. At this depth, the abundance of the markers for sulfide oxidation was 0.39 OGE. The marker specific for sulfate reduction suggested highest potential in the lower hypolimnion. Markers for bacteria using anaerobic ammonia oxidation (anammox) were not detectable in the data set. We could find markers for ammonia monooxygenase, but a manual inspection of the hits to these hidden Markov models (HMMs) indicated that these were in fact to its paralog methane monooxygenase. Thus, these HMMs were used as markers for methanotrophy instead of nitrification.

The potential for aerobic respiration was stable from epilimnion to upper hypolimnion, whereas the highest potential for microaerophilic respiration was right above the depth where oxygen concentration dropped below detection limit, and the potential for anaerobic respiration was highest in the upper hypolimnion. Potentials for using alternative electron acceptors for respiratory reactions followed the classic redox tower, being nitrous oxide (N_2_O), NO_2_, NO_3_, Fe^3+^, SO_4_, and CO_2_ from metalimnion to the lake bottom. The profile suggested that the reactions in the denitrification pathway (reduction of nitrate to N_2_O or further to nitrogen gas N_2_) were divided between multiple organisms inhabiting different redox zones in the oxic-anoxic boundary layer, as the highest potentials for different parts of the pathway were found at different depths (Fig. 4). Previous results on stratified lakes suggest that N_2_O commonly accumulates at the oxycline (22), as it is produced through both anaerobic nitrate reduction in the anoxic hypolimnion and aerobic nitrification in the oxic water layers. Furthermore, the potential for nitrite reduction was higher than the potential for N_2_O reduction, potentially explaining N_2_O accumulation (9, 22). This is due to the expression of the N_2_O reductase gene (*nosZ*) being more sensitive to oxygen than expression of the nitrite reductase gene (*nirK*) (23). In our Pfam/TIGRFAM profiles, the potential for nitrate reduction occurred in the upper hypolimnion and that for N_2_O reduction occurred in the lower metalimnion, while markers for nitrification were sparse, suggesting that N_2_O dynamics is driven by anaerobic processes, potentially using NO_3_ as electron acceptor. The general patterns for Fe^3+^ and SO_4_ reduction could not be assessed due to lack of pathway-specific HMMs, but these were investigated using metagenome-assembled genomes (MAGs) (Table S2).

### Unrecognized metabolic and chemoautotrophic potential in novel bacterial taxa.

**FIG 5:**
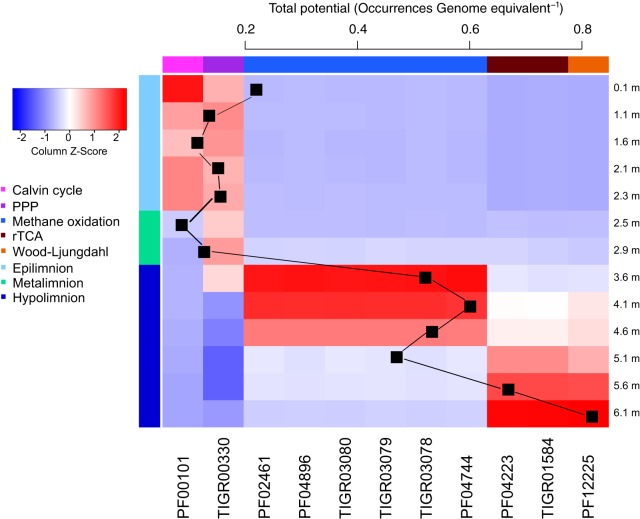
Heat map visualizing the abundances of Pfam/TIGRFAM markers (as given in *Z*-score standardized per genome equivalent) related to carbon fixation and the total abundance of these genes in the data set as a sum of the average abundances of the markers for each pathway, respectively. Only those Pfams/TIGRFAMs that had significantly different abundances between different layers of the lake are displayed. Colors at the top of each of the columns reflect the function that the marker represents, and colors on the left side of the heat map illustrate different layers of the lake.

In general, the microbial community had potential for three different pathways of autotrophic carbon fixation (Fig. 5). In the epilimnion, the Calvin-Benson-Bassham cycle (CBB; also called the reductive pentose phosphate pathway) was highly abundant, while the reductive citric acid cycle (rTCA) and the Wood-Ljungdahl pathway (WL; also called the reductive acetyl coenzyme A [acetyl-CoA] cycle) were mainly found in the hypolimnion. For the fourth carbon assimilation pathway present in the communities, the 3-hydroxypropionate cycle (3HP), no specific HMMs could be found. However, protein annotations from Prokka (24) suggested that multiple organisms possessed an almost full 3HP pathway, but certain genes in the pathway, such as *mcr* (malonyl-CoA reductase), were not present in the data set. The data suggested increasing autotrophic potential toward the bottom of the lake with a peak right below the oxycline and the highest potential at the lake bottom with the total abundance of pathways related to carbon fixation being close to 1 OGE (Fig. 5).

We were able to construct a total of 270 metagenome-assembled genomes (MAGs). Of these, we analyzed all the genomes with >40% completeness and <4% contamination, resulting in 93 MAGs characterized through functional profiling. The cutoff for completeness was set lower than recommended for high-quality MAGs (25) as our focus was on putative chemotrophs and autotrophs, rather than doing a complete metabolic mapping of all the MAGs. Thus, some of these MAGs likely are missing some pathways present in their genomes. However, as the contamination threshold was set to the level of high-quality MAGs (25), we are confident that the pathways that we found truly are present in the MAG in question. These 93 MAGs represented a diverse group of organisms (as determined through PhyloPhlAn [Fig. S4] [26]) and had the potential to use a wide range of different electron donors and acceptors and a full range of metabolic pathways from chemolithoautotrophy to photoheterotrophy (Table S2). Here, we concentrate on the most abundant organisms with chemotrophic potential.

10.1128/mBio.00916-18.4 FIG S4  Phylogenetic tree of the metagenome-assembled genomes depicting their taxonomic placement in the tree of life. Download FIG S4, PDF file, 1 MB. Copyright © 2018 Peura et al. 2018 Peura et al. This content is distributed under the terms of the Creative Commons Attribution 4.0 International license.

As stated above, the chemotrophic organisms appeared to be deriving their energy mainly from oxidation of sulfur, iron, and hydrogen compounds. The most abundant MAGs with potential for sulfur oxidation (*sox* operon typically including genes *soxACXYZ*) included organisms related to *Chlorobium* (bin 4), *Polynucleobacter* (bins 15 and 120), *Comamonadaceae* (bin 108), *Ferrovales* (bin 139a), *Rhizobiales* (bin 139b), and *Acetobacteraceae* (bins 72 and 168). However, only the *Chlorobium* and *Ferrovales* MAGs had potential for autotrophy. *Chlorobium* was located in the lake hypolimnion and had a near-complete complex for oxidation of reduced sulfur compounds and also a near-complete CBB pathway for autotrophic carbon assimilation. Further, two other *Chlorobium* MAGs (bins 52 and 92) had a *dsr* operon, which is used in reverse in *Chlorobium* to oxidize sulfur (27). *Chlorobium* can grow both autotrophically and mixotrophically (28), and they derive the energy for autotrophy from sunlight using sulfur oxidization to produce reducing equivalents for growth. Thus, they are photoautotrophic organisms rather than chemoautotrophs.

The other MAG with capacity for sulfur oxidation and inorganic carbon assimilation was a MAG closely related to the betaproteobacterial *Ferrovales* order (bin 139a). This MAG was 4.6 Mb in size (91.3% complete with 0.67% contamination) and included all of the genes in the CBB and rTCA pathways, suggesting the potential for autotrophy. While the MAG had potential for sulfur oxidation, it also included genes for iron oxidation: cytochrome *cyc1*, cytochrome *c* oxidase (*ctaCDE*), ubiquinol-cytochrome *c* reductase (*petABC*), and NADH:quinone oxidoreductase (*nuoABCDEFGHIJKLMN*). Further, the genome included an iron oxidase, which was located on the same contig as cytochrome *cyc1*, completing the pathway. This is consistent with the closest cultivated organism, iron-oxidizing Ferrovum myxofaciens (29), which thrives in acidic mine drainage (29). The MAG also included markers indicative of aerobic, anaerobic, and microaerophilic respiration. Thus, the electron acceptor could be O_2_ when it is available. However, we could not identify any other electron acceptors for this organism. Thus, the genomic features together with the abundance distribution in the lake suggest that this organism is a chemoautotroph inhabiting suboxic to anoxic environments, using iron or sulfur oxidation as an energy source. In our data, this organism was rather abundant in both 16S rRNA gene amplicon data and the metagenomes but has not been previously found in boreal lakes. This particulate taxon was abundant in a narrow zone (between 2.5 and 3.6 m); thus, a possible reason why it has been previously missed is the sparse sampling schemes of many experiments. Moreover, it represents a recently established bacterial order (29), and this taxon may have been classified as “uncultured *Betaproteobacteria*” in previous studies.

MAGs with autotrophic potential also included organisms that would appear to acquire energy by combining oxidation of hydrogen with sulfate or nitrate reduction. Hydrogenases found in the data represented FeFe- and NiFe-type hydrogenases as described in reference 30, with the latter type being more prevalent among the MAGs. For example, a MAG closely related to *Gallionellaceae* (bin 129) carried a 1e-type hydrogenase, which is specifically used for electron input to sulfur respiration, and the MAG did have potential for sulfur reduction. It also had a full CBB pathway. Bin 129 was closely related to betaproteobacterial Sideroxydans lithotrophicus, which thrives in the same environment as *Ferrovales* (31). Similar to bin 129, this organism has the potential for the CBB cycle; however, it has been suggested to derive its energy from iron rather than hydrogen oxidation (28). Another MAG, closely related to *Desulfobulbaceae* (bin 93), had a 1c-type hydrogenase, also typically related to sulfate respiration, and an almost complete pathway for sulfate reduction. It also appeared to have the potential for CO_2_ fixation through the reductive tricarboxylic acid (rTCA) cycle. The closest relative to this bin, Desulfotalea psychrophila, is also a sulfate reducer but has been reported to be heterotrophic (32). However, D. psychrophila inhabits cold environments, which is consistent with bin 93, which was most abundant in the deep layer of the lake, where the water temperature is around 4°C throughout the year.

In accordance with the redox potentials in the water column and with the literature (33, 34), the reduction of nitrate to N_2_ was dispersed among multiple organisms. “*Candidatus* Methyloumidiphilus alinensis” (bin 10) (35) and a MAG closely affiliated with *Crenotrix* (bin 149) both had a complete *narGHIJ* operon for nitrate reduction and also genes for methane oxidation, as has been previously described for a member of the *Methylobacter* family (36). Gene *nosZ*, coding for N_2_O reductase, was present in two high-quality MAGs, which were taxonomically assigned to *Myxococcales* (bin 233) and *Bacteroidetes* (bin 64). However, these did not appear to be autotrophic organisms. The *norCB* operon, encoding nitric oxide (NO) reductase, was complete in two MAGs, in “*Candidatus* Methyloumidiphilus alinensis” and in a MAG affiliated with *Comamonadaceae* (bin 239). The latter was also carrying the potential for the CBB cycle. The possible electron acceptors for the organism were sulfur and hydrogen. At the very bottom of the lake, we could identify three archaeal MAGs. Two of these were hydrogen oxidizing (hydrogenotrophic methanogenesis; bins 133 and 155, closely related to *Methanolinea* and *Methanoregula*, respectively), while the third one was using acetate (acetogenic methanogenesis; bin 74, closely related to *Methanosaeta*).

### Conclusions.

**FIG 6:**
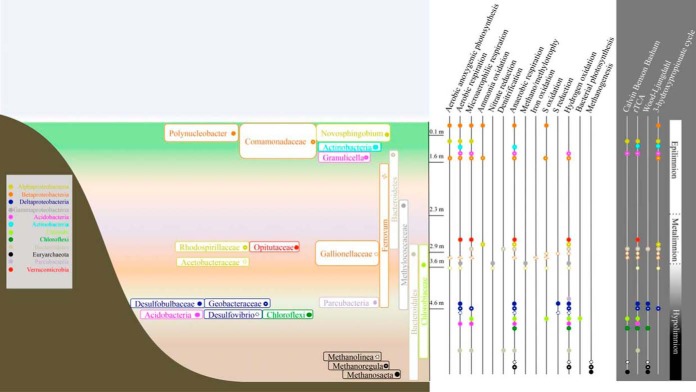
Redox reactions potentially driving the autotrophic processes in the lake and the most abundant organisms harboring these pathways. The colors represent the taxonomic annotation of the organisms at the phylum level or, in the case of *Proteobacteria*, the order. The height of the boxes visualizes the depth of the maximum abundance, and in the case of multiple MAGs of the same taxon, the height of the box is covering the depths where the organisms were most abundant. Also, for the organisms with multiple MAGs with the same taxonomy, dominant pathways are displayed. Only the MAGs with marker genes specific for inorganic carbon fixation pathways are presented.

Consistent with our expectations, the microbial community included a variety of different chemotrophic pathways. Further, the microbial community driving these processes contained abundant novel organisms with the potential for autotrophy. The assembly of the functional potentials was in accordance with redox potentials of the electron acceptors in the lake (Fig. 6). Further, consistent with our hypothesis, there was a diverse set of abundant chemoautotrophic organisms below the euphotic zone of the lake. The autotrophic community had an unexpected major community member closely related to *Ferrovales*, presumably thriving via iron oxidation. We also identified other novel autotrophs, such as an organism related to recently identified autotrophic Sideroxydans lithotrophicus.

The fact that many of the potentially autotrophic microbes were among the most abundant microbes in the lake and the abundance of photoautotrophic *Chlorobium* emphasize the potential role of internal carbon cycling as a process that mitigates the flow of CO_2_ from boreal lakes to the atmosphere. Our results suggest that autotrophic iron-, sulfur-, and hydrogen-oxidizing microbes have a high potential to significantly contribute to inorganic carbon fixation in the lake. In fact, our measurements of inorganic carbon incorporation suggested that a significant amount of CO_2_ originating from degradation of autochthonous and terrestrial carbon can be reincorporated into biomass in the poorly illuminated, anoxic layer of the lake. This is also well in line with results showing that chemolithoautotrophy significantly contributes to carbon and energy flow in meromictic Lake Kivu (2) and with recent results regarding the autotrophic potential in boreal lakes (8). These processes are strongly dependent on the prevalent environmental conditions, which have been predicted to change following the warming of the climate. Thus, our results suggest that if the predicted alterations in lake environment, such as changes in mixing patterns, should happen, we may expect reorganization of the metabolic processes in the lake, which would have unknown implications for the carbon flow in the water column.

## MATERIALS AND METHODS

### Site description and sampling.

The study lake, Alinen Mustajärvi, is situated in southern Finland (61°12′N, 25°06′E). It is a 0.007-km^2^ headwater lake with a maximum depth of 6.5 m and an estimated volume of 31 × 10^3^ m^3^. The catchment area is <0.5 km^2^, and it consists of >90% coniferous forest and <10% peatland. The lake is characterized by steep oxygen stratification during summer and also during the ice cover period, which lasts from late November until late April. The stratification is disrupted by regular autumn and irregular spring mixings. As such, Alinen Mustajärvi is a representative for the millions of lakes and ponds in the arctic and boreal zones.

The metagenome sampling was conducted in the beginning of September 2013, at the end of the stratification period. The lake was sampled at 13 depths: the oxic epilimnion was sampled at 0.1, 1.1, 1.6, 2.1, and 2.3 m; the metalimnion was sampled at 2.5 and 2.9 m; and the hypolimnion was sampled at 3.6, 4.1, 4.6, 5.1, 5.6, and 6.1 m. Water samples were taken with a 20-cm-long acrylic tube sampler (Limnos; volume, 1.1 liter) and subsequently analyzed for nutrients (NO_2_/NO_3_, PO_4_, NH_4_, total N, total P, and SO_4_), gases (CH_4_ and CO_2_), and dissolved organic carbon (DOC) concentration. Nutrient analyses were conducted using standard methods (http://www.sfs.fi/). Gas and DOC analyses were done as described in reference 37, and iron analysis was performed as described in reference 38. The dark carbon fixation was measured in 2008 as an increase or decrease of dissolved inorganic carbon (DIC) concentration during a 24-h incubation at the depth of 3 m in two foil-covered 50-ml glass-stoppered biological oxygen demand (BOD) bottles. The measurements were conducted every second week from the beginning of May until the end of October, and the changes in DIC concentration were analyzed according to reference 39. In August 2008, a clone library was created from the 0.5-, 2.5-, 4.5-, and 5.5-m depths, consisting of a total of 303 sequences (40).

### Amplicon and metagenome analysis.

The samples for metagenomic analysis of lake microbiota were taken by filtering water through 0.2-µm polycarbonate filters which were then frozen at −78°C until further analysis. The DNA was extracted from the filters using the MoBio PowerSoil DNA extraction kit (MoBio Laboratories). Sample preparation for 16S rRNA gene analysis and the following sequence processing were conducted as previously described (41).

Shotgun metagenomic libraries were prepared from 10 ng of genomic DNA. First, the genomic DNA was sheared using a focused ultrasonicator (Covaris E220), and subsequently, sequencing libraries were prepared with the Thruplex FD Prep kit from Rubicon Genomics according to the manufacturer’s protocol (R40048-08, QAM-094-002). Library size selection was made with AMPure XP beads (Beckman Coulter) in a 1:1 ratio. The prepared sample libraries were quantified using the KAPA Biosystems next-generation sequencing library quantitative PCR (qPCR) kit and run on a StepOnePlus (Life Technologies) real-time PCR instrument. The quantified libraries were then prepared for sequencing on the Illumina HiSeq sequencing platform with a TruSeq paired-end cluster kit, v3, and Illumina’s cBot instrument to generate a clustered flow cell for sequencing. Sequencing of the flow cell was performed on the Illumina HiSeq2500 sequencer using Illumina TruSeq SBS sequencing kits, v3, following a 2 × 100 indexed high-output run protocol.

The sequencing produced a total of 120.5 Gb of sequence data. Reads were filtered based on their quality scores using Sickle (version v1.33) (42) and subsequently assembled with Ray (version v2.3.1) (43). Assembled contigs from kmer sizes of 51, 61, 71, and 81 were cut into 1,000-bp pieces and scaffolded with Newbler (454 Life Sciences, Roche Diagnostics). Mapping of the original reads to the Newbler assembly was done using Bowtie 2 (version v2.15.0) (44), while duplicates were removed using Picard-tools (version 1.101; https://github.com/broadinstitute/picard), and for computing coverage, BEDTools (45) was used. Details on the assembly results are presented in Table 1. The data were then normalized using the counts of 139 single-copy genes as previously described (20). Assembled contigs were binned with MetaBAT (version v0.26.3) (46) to reconstruct genomes of the most abundant lake microbes (metagenome-assembled genomes [MAGs]). The quality of the MAGs was evaluated using CheckM (version v1.0.6) (47). Cutoffs for high-quality MAGs were set to ≥40% for completeness and ≤4% for contamination. The placement of the MAGs in the microbial tree of life was estimated using PhyloPhlAn (version v1.1.0) (26).

The functional potential of the metagenomes was assessed from assembled data using the hidden Markov models (HMMs) of the Pfam and TIGRFAM databases (48, 49) and the HMMER3 software (version v3.1b2) (50). Special attention was paid to pathways linked to energy metabolism and carbon cycle. To ensure pathway specificity, marker HMMs were chosen to be unique to specific pathways (see Table S1 in the supplemental material). Normalized coverage information on the contigs combined with HMMs of specific marker genes (Table S1) was used to predict protein domains related to energy metabolism and carbon incorporation to biomass. Only marker genes that were found to exhibit a significantly different distribution between layers are reported (*P* values in Table S1). All of the MAGs were also annotated using Prokka (version v1.11) (24). The metabolic potentials of all high-quality MAGs were evaluated based on Prokka annotations (Table S2). In the case of novel functional combinations, such as combination of AAP with CBB, the contigs were blasted against the NCBI nr database to verify that the closest relatives of the overall genes in these contigs matched the PhyloPhlAn annotation. MAGs with special functional properties were also visualized with nonmetric multidimensional scaling to check the placement of the contigs with markers within all the contigs comprising the MAG in question. All statistical analyses were done using R software (http://www.R-project.org [51]) and packages vegan (52) and mpmcorrelogram (53). Differences in the abundance of marker HMMs between layers were tested using a permutation test (1,000 permutations) on the *t* statistics with the package MetagenomeSeq (54).

10.1128/mBio.00916-18.5 TABLE S1  Table listing HMMs used to compare the abundances of different energy and carbon assimilation pathways between different depth layers and *P* values of these comparisons. Download TABLE S1, TXT file, 0 MB. Copyright © 2018 Peura et al. 2018 Peura et al. This content is distributed under the terms of the Creative Commons Attribution 4.0 International license.

10.1128/mBio.00916-18.6 TABLE S2  Characteristics of the high-quality MAGs. Bin Id as given by Metabat, taxonomic annotation by (i) manual annotation from a phylogenetic tree from PhyloPhlAn and (ii) PhyloPhlAn automatic annotation, size of the MAG, average proportion of the MAG among all samples, depth of the maximum abundance and the proportion in this depth, completeness, contamination and strain heterogeneity of the MAG, and number of marker genes for different energy and carbon assimilation pathways in the MAG. Presence of key genes in each of the pathways is shown with letters. AAP, M = *pufM*; methanogenesis, M = *mcrA*; CBB, R = RuBisCO; PPP = pentose phosphate cycle, Z = glucose-6-phosphate dehydrogenase, P = 6-phosphogluconolactonase, G = 6-phosphogluconate dehydrogenase; rTCA, C = citrate lyase, O = oxoglutarate synthase, P = pyruvate synthase; WL, C = CO dehydrogenase; 3HP, A = acetyl-CoA carboxylase, P = propionyl-CoA carboxylase. For hydrogenases, subgroups are presented as in the work of D. Søndergaard, C. N. Pedersen, and C. Greening, Sci Rep 6:34212, 2016 (https://doi.org/10.1038/srep34212). Download TABLE S2, XLS file, 0.1 MB. Copyright © 2018 Peura et al. 2018 Peura et al. This content is distributed under the terms of the Creative Commons Attribution 4.0 International license.

### Accession number(s).

The raw data have been deposited in the NCBI Sequence Read Archive under accession no. SRP076290.
