# Supplementary figures and images for: Novel Autotrophic Organisms Contribute Significantly to the Internal Carbon Cycling Potential of a Boreal Lake
Source: mBio. 2018 Aug 14;9(4):e00916-18. doi: 10.1128/mBio.00916-18 (PMC6094481; doi:10.1128/mBio.00916-18)

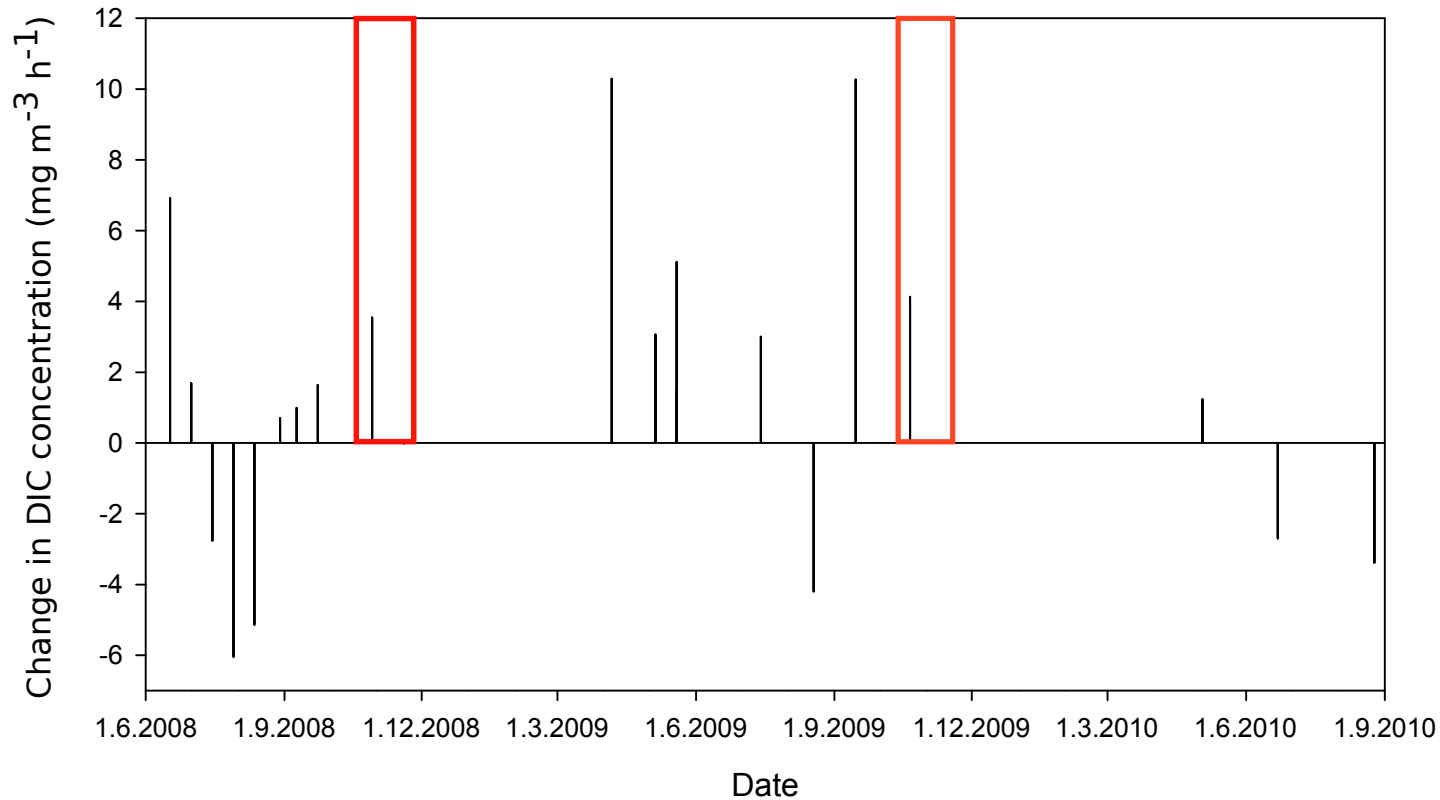

Supplement: FIG S1 [file mbo004184019sf1.pdf]

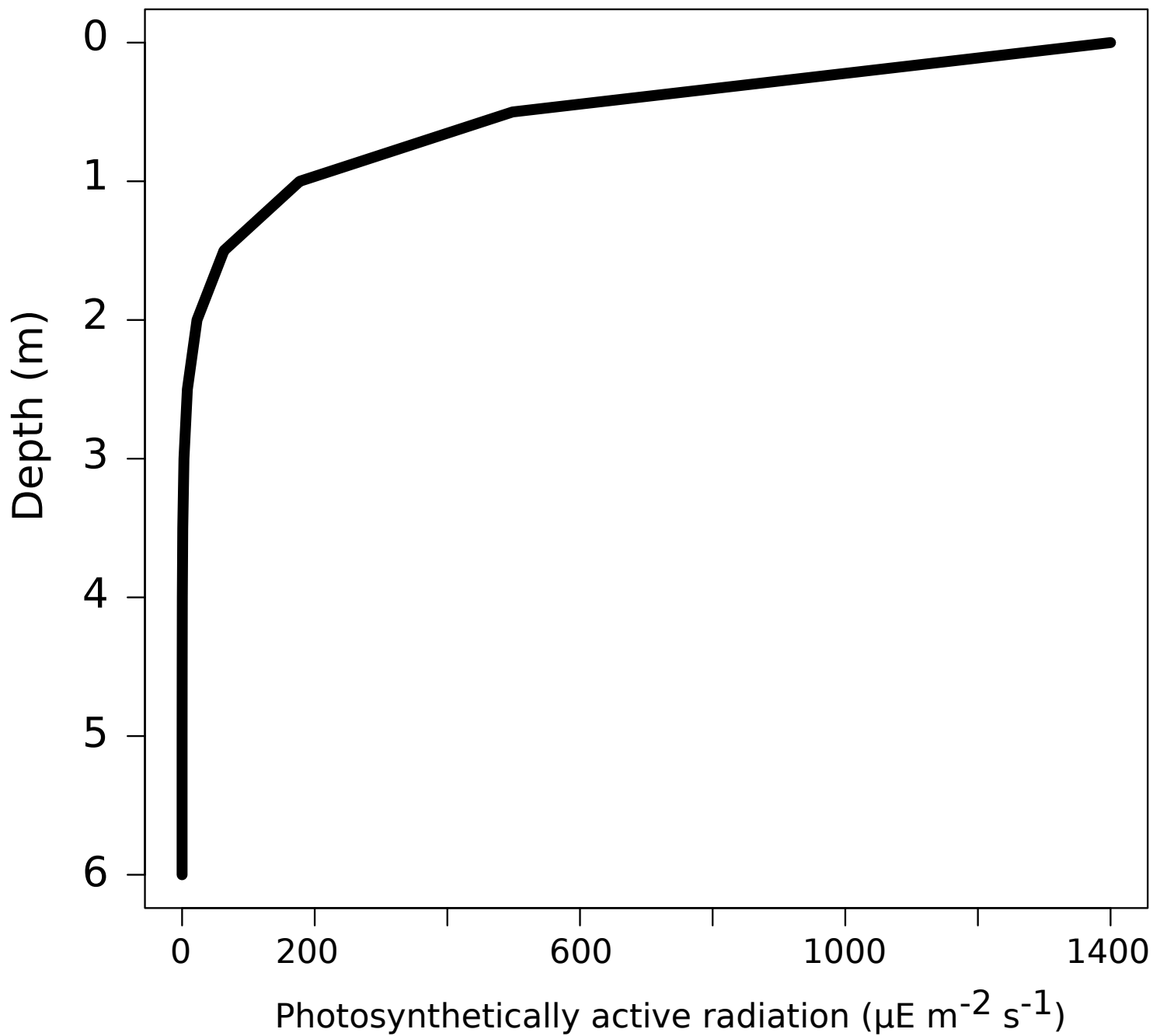

Supplement: FIG S2 [file mbo004184019sf2.pdf]

# A

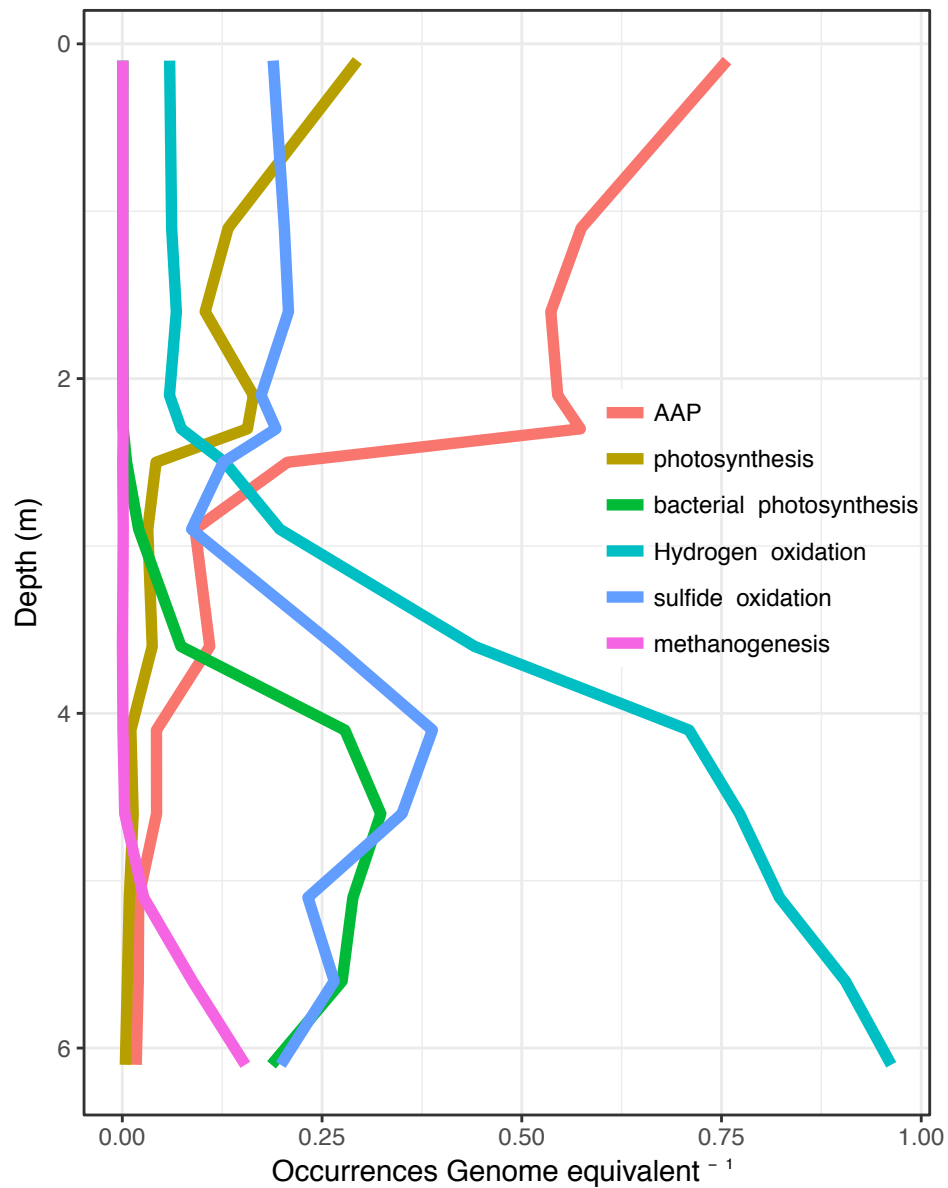

# B

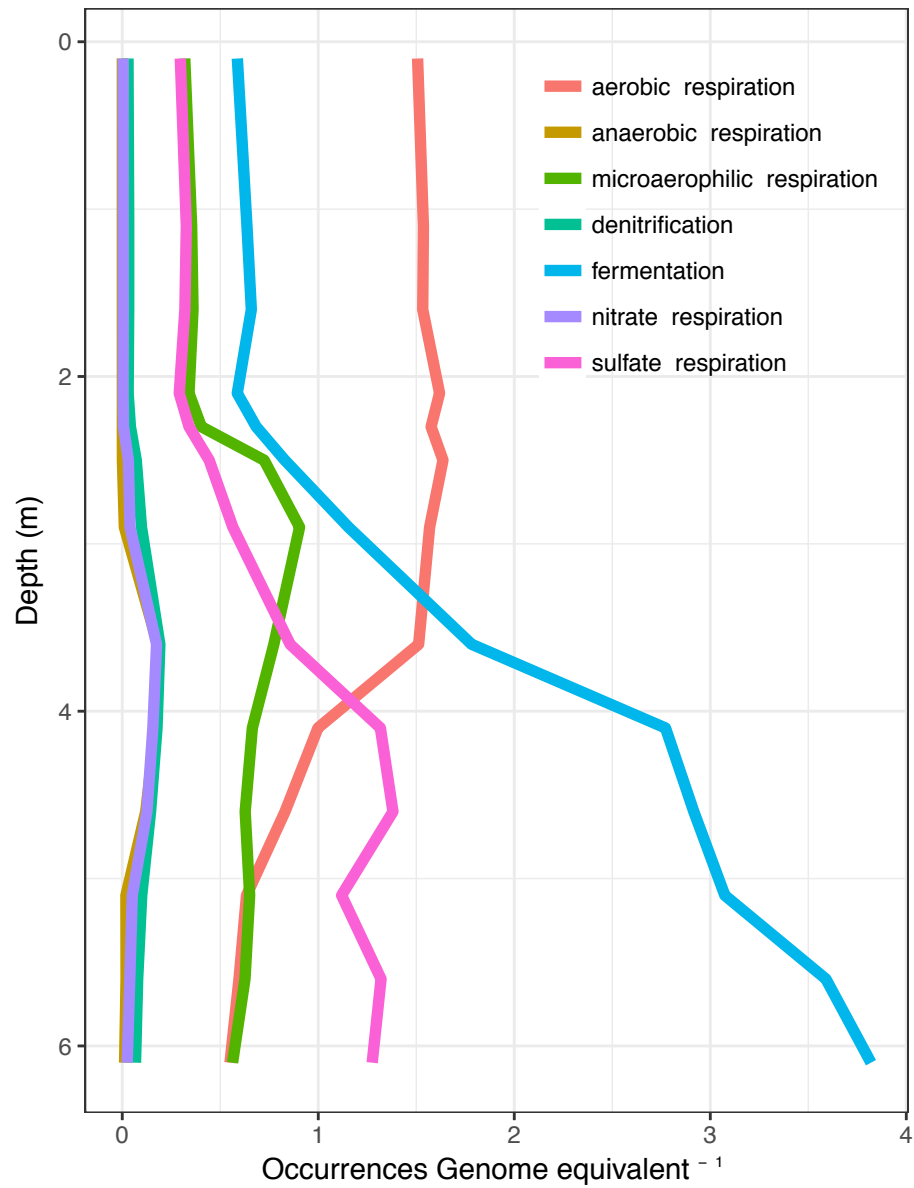

Supplement: FIG S3 [file mbo004184019sf3.pdf]
