# Supplementary material for: Novel Autotrophic Organisms Contribute Significantly to the Internal Carbon Cycling Potential of a Boreal Lake
Source: mBio. 2018 Aug 14;9(4):e00916-18. doi: 10.1128/mBio.00916-18 (PMC6094481; doi:10.1128/mBio.00916-18)
Supplement: FIG S4 [file mbo004184019sf4.pdf]

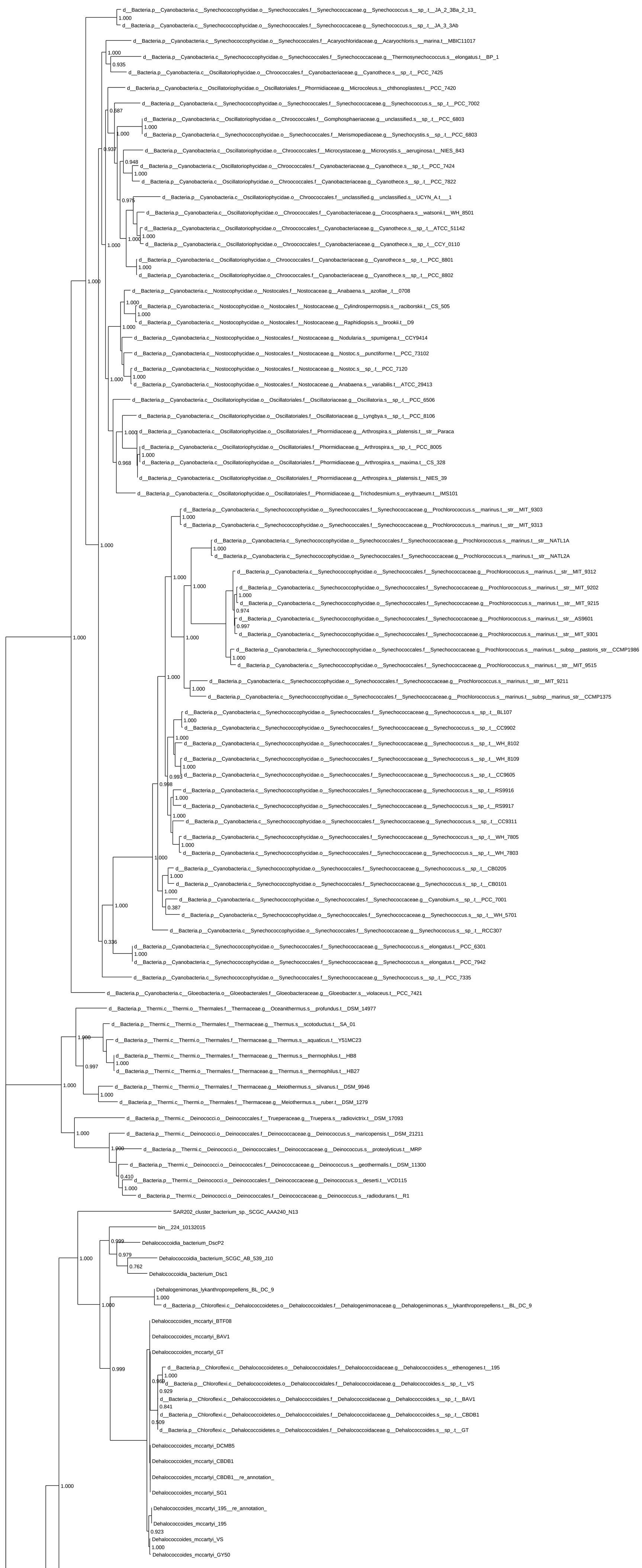
d__Bacteria.p__Cyanobacteria.c__Synechococcophycidae.o__Synechococcales.f__Synechococcaceae.g__Synechococcus.s__sp_.t__JA_2_3Ba_2_13_
d__Bacteria.p__Cyanobacteria.c__Synechococcophycidae.o__Synechococcales.f__Synechococcaceae.g__Synechococcus.s__sp_.t__JA_3_3Ab
d__Bacteria.p__Cyanobacteria.c__Synechococcophycidae.o__Synechococcales.f__Acaryochloridaceae.g__Acaryochloris.s__marina.t__MBIC11017
d__Bacteria.p__Cyanobacteria.c__Synechococcophycidae.o__Synechococcales.f__Synechococcaceae.g__Thermosynechococcus.s__elongatus.t__BP_1
d__Bacteria.p__Cyanobacteria.c__Oscillatoriophycidae.o__Chroococcales.f__Cyanobacteriaceae.g__Cyanothece.s__sp_.t__PCC_7425
d__Bacteria.p__Cyanobacteria.c__Oscillatoriophycidae.o__Oscillatoriales.f__Phormidiaceae.g__Microcoleus.s__chthonoplastes.t__PCC_7420
d__Bacteria.p__Cyanobacteria.c__Synechococcophycidae.o__Synechococcales.f__Synechococcaceae.g__Synechococcus.s__sp_.t__PCC_7002
d__Bacteria.p__Cyanobacteria.c__Oscillatoriophycidae.o__Chroococcales.f__Gomphosphaeriaceae.g__unclassified.s__sp_.t__PCC_6803
d__Bacteria.p__Cyanobacteria.c__Synechococcophycidae.o__Synechococcales.f__Merismopediaceae.g__Synechocystis.s__sp_.t__PCC_6803
d__Bacteria.p__Cyanobacteria.c__Oscillatoriophycidae.o__Chroococcales.f__Microcystaceae.g__Microcystis.s__aeruginosa.t__NIES_843
d__Bacteria.p__Cyanobacteria.c__Oscillatoriophycidae.o__Chroococcales.f__Cyanobacteriaceae.g__Cyanothece.s__sp_.t__PCC_7424
d__Bacteria.p__Cyanobacteria.c__Oscillatoriophycidae.o__Chroococcales.f__Cyanobacteriaceae.g__Cyanothece.s__sp_.t__PCC_7822
d__Bacteria.p__Cyanobacteria.c__Oscillatoriophycidae.o__Chroococcales.f__unclassified.g__unclassified.s__UCYN_A.t__1
d__Bacteria.p__Cyanobacteria.c__Oscillatoriophycidae.o__Chroococcales.f__Cyanobacteriaceae.g__Crocosphaera.s__watsonii.t__WH_8501
d__Bacteria.p__Cyanobacteria.c__Oscillatoriophycidae.o__Chroococcales.f__Cyanobacteriaceae.g__Cyanothece.s__sp_.t__ATCC_51142
d__Bacteria.p__Cyanobacteria.c__Oscillatoriophycidae.o__Chroococcales.f__Cyanobacteriaceae.g__Cyanothece.s__sp_.t__CCY_0110
d__Bacteria.p__Cyanobacteria.c__Oscillatoriophycidae.o__Chroococcales.f__Cyanobacteriaceae.g__Cyanothece.s__sp_.t__PCC_8801
d__Bacteria.p__Cyanobacteria.c__Oscillatoriophycidae.o__Chroococcales.f__Cyanobacteriaceae.g__Cyanothece.s__sp_.t__PCC_8802
d__Bacteria.p__Cyanobacteria.c__Nostocophycidae.o__Nostocales.f__Nostocaceae.g__Anabaena.s__azollae_.t__0708
d__Bacteria.p__Cyanobacteria.c__Nostocophycidae.o__Nostocales.f__Nostocaceae.g__Cylindrospermopsis.s__raciborskii.t__CS_505
d__Bacteria.p__Cyanobacteria.c__Nostocophycidae.o__Nostocales.f__Nostocaceae.g__Raphidiopsis.s__brookii.t__D9
d__Bacteria.p__Cyanobacteria.c__Nostocophycidae.o__Nostocales.f__Nostocaceae.g__Nodularia.s__spumigena.t__CCY9414
d__Bacteria.p__Cyanobacteria.c__Nostocophycidae.o__Nostocales.f__Nostocaceae.g__Nostoc.s__punctiforme.t__PCC_73102
d__Bacteria.p__Cyanobacteria.c__Nostocophycidae.o__Nostocales.f__Nostocaceae.g__Nostoc.s__sp_.t__PCC_7120
d__Bacteria.p__Cyanobacteria.c__Nostocophycidae.o__Nostocales.f__Nostocaceae.g__Anabaena.s__variabilis.t__ATCC_29413
d__Bacteria.p__Cyanobacteria.c__Oscillatoriophycidae.o__Oscillatoriales.f__Oscillatoriaceae.g__Oscillatoria.s__sp_.t__PCC_6506
d__Bacteria.p__Cyanobacteria.c__Oscillatoriophycidae.o__Oscillatoriales.f__Oscillatoriaceae.g__Lyngbya.s__sp_.t__PCC_8106
d__Bacteria.p__Cyanobacteria.c__Oscillatoriophycidae.o__Oscillatoriales.f__Phormidiaceae.g__Arthrospira.s__platensis.t__str__Paraca
d__Bacteria.p__Cyanobacteria.c__Oscillatoriophycidae.o__Oscillatoriales.f__Phormidiaceae.g__Arthrospira.s__sp_.t__PCC_8005
d__Bacteria.p__Cyanobacteria.c__Oscillatoriophycidae.o__Oscillatoriales.f__Phormidiaceae.g__Arthrospira.s__maxima.t__CS_328
d__Bacteria.p__Cyanobacteria.c__Oscillatoriophycidae.o__Oscillatoriales.f__Phormidiaceae.g__Arthrospira.s__platensis.t__NIES_39
d__Bacteria.p__Cyanobacteria.c__Oscillatoriophycidae.o__Oscillatoriales.f__Phormidiaceae.g__Trichodesmium.s__erythraeum.t__IMS101
d__Bacteria.p__Cyanobacteria.c__Synechococcophycidae.o__Synechococcales.f__Synechococcaceae.g__Prochlorococcus.s__marinus.t__str__MIT_9303
d__Bacteria.p__Cyanobacteria.c__Synechococcophycidae.o__Synechococcales.f__Synechococcaceae.g__Prochlorococcus.s__marinus.t__str__MIT_9313
d__Bacteria.p__Cyanobacteria.c__Synechococcophycidae.o__Synechococcales.f__Synechococcaceae.g__Prochlorococcus.s__marinus.t__str__NATL1A
d__Bacteria.p__Cyanobacteria.c__Synechococcophycidae.o__Synechococcales.f__Synechococcaceae.g__Prochlorococcus.s__marinus.t__str__NATL2A
d__Bacteria.p__Cyanobacteria.c__Synechococcophycidae.o__Synechococcales.f__Synechococcaceae.g__Prochlorococcus.s__marinus.t__str__MIT_9312
d__Bacteria.p__Cyanobacteria.c__Synechococcophycidae.o__Synechococcales.f__Synechococcaceae.g__Prochlorococcus.s__marinus.t__str__MIT_9202
d__Bacteria.p__Cyanobacteria.c__Synechococcophycidae.o__Synechococcales.f__Synechococcaceae.g__Prochlorococcus.s__marinus.t__str__MIT_9215
d__Bacteria.p__Cyanobacteria.c__Synechococcophycidae.o__Synechococcales.f__Synechococcaceae.g__Prochlorococcus.s__marinus.t__str__AS9601
d__Bacteria.p__Cyanobacteria.c__Synechococcophycidae.o__Synechococcales.f__Synechococcaceae.g__Prochlorococcus.s__marinus.t__str__MIT_9301
d__Bacteria.p__Cyanobacteria.c__Synechococcophycidae.o__Synechococcales.f__Synechococcaceae.g__Prochlorococcus.s__marinus.t__subsp__pastoris_str__CCMP1986
d__Bacteria.p__Cyanobacteria.c__Synechococcophycidae.o__Synechococcales.f__Synechococcaceae.g__Prochlorococcus.s__marinus.t__str__MIT_9515
d__Bacteria.p__Cyanobacteria.c__Synechococcophycidae.o__Synechococcales.f__Synechococcaceae.g__Prochlorococcus.s__marinus.t__str__MIT_9211
d__Bacteria.p__Cyanobacteria.c__Synechococcophycidae.o__Synechococcales.f__Synechococcaceae.g__Prochlorococcus.s__marinus.t__subsp__marinus_str__CCMP1375
d__Bacteria.p__Cyanobacteria.c__Synechococcophycidae.o__Synechococcales.f__Synechococcaceae.g__Synechococcus.s__sp_.t__BL107
d__Bacteria.p__Cyanobacteria.c__Synechococcophycidae.o__Synechococcales.f__Synechococcaceae.g__Synechococcus.s__sp_.t__CC9902
d__Bacteria.p__Cyanobacteria.c__Synechococcophycidae.o__Synechococcales.f__Synechococcaceae.g__Synechococcus.s__sp_.t__WH_8102
d__Bacteria.p__Cyanobacteria.c__Synechococcophycidae.o__Synechococcales.f__Synechococcaceae.g__Synechococcus.s__sp_.t__WH_8109
d__Bacteria.p__Cyanobacteria.c__Synechococcophycidae.o__Synechococcales.f__Synechococcaceae.g__Synechococcus.s__sp_.t__CC9605
d__Bacteria.p__Cyanobacteria.c__Synechococcophycidae.o__Synechococcales.f__Synechococcaceae.g__Synechococcus.s__sp_.t__RS9916
d__Bacteria.p__Cyanobacteria.c__Synechococcophycidae.o__Synechococcales.f__Synechococcaceae.g__Synechococcus.s__sp_.t__RS9917
d__Bacteria.p__Cyanobacteria.c__Synechococcophycidae.o__Synechococcales.f__Synechococcaceae.g__Synechococcus.s__sp_.t__CC9311
d__Bacteria.p__Cyanobacteria.c__Synechococcophycidae.o__Synechococcales.f__Synechococcaceae.g__Synechococcus.s__sp_.t__WH_7805
d__Bacteria.p__Cyanobacteria.c__Synechococcophycidae.o__Synechococcales.f__Synechococcaceae.g__Synechococcus.s__sp_.t__WH_7803
d__Bacteria.p__Cyanobacteria.c__Synechococcophycidae.o__Synechococcales.f__Synechococcaceae.g__Synechococcus.s__sp_.t__CB0205
d__Bacteria.p__Cyanobacteria.c__Synechococcophycidae.o__Synechococcales.f__Synechococcaceae.g__Synechococcus.s__sp_.t__CB0101
d__Bacteria.p__Cyanobacteria.c__Synechococcophycidae.o__Synechococcales.f__Synechococcaceae.g__Cyanobium.s__sp_.t__PCC_7001
d__Bacteria.p__Cyanobacteria.c__Synechococcophycidae.o__Synechococcales.f__Synechococcaceae.g__Synechococcus.s__sp_.t__WH_5701
d__Bacteria.p__Cyanobacteria.c__Synechococcophycidae.o__Synechococcales.f__Synechococcaceae.g__Synechococcus.s__sp_.t__RCC307
d__Bacteria.p__Cyanobacteria.c__Synechococcophycidae.o__Synechococcales.f__Synechococcaceae.g__Synechococcus.s__elongatus.t__PCC_6301
d__Bacteria.p__Cyanobacteria.c__Synechococcophycidae.o__Synechococcales.f__Synechococcaceae.g__Synechococcus.s__elongatus.t__PCC_7942
d__Bacteria.p__Cyanobacteria.c__Synechococcophycidae.o__Synechococcales.f__Synechococcaceae.g__Synechococcus.s__sp_.t__PCC_7335
d__Bacteria.p__Cyanobacteria.c__Gloeobacteria.o__Gloeobacterales.f__Gloeobacteraceae.g__Gloeobacter.s__violaceus.t__PCC_7421
d__Bacteria.p__Thermi.c__Thermi.o__Thermales.f__Thermaceae.g__Oceanithermus.s__profundus.t__DSM_14977
d__Bacteria.p__Thermi.c__Thermi.o__Thermales.f__Thermaceae.g__Thermus.s__scotoductus.t__SA_01
d__Bacteria.p__Thermi.c__Thermi.o__Thermales.f__Thermaceae.g__Thermus.s__aquaticus.t__Y51MC23
d__Bacteria.p__Thermi.c__Thermi.o__Thermales.f__Thermaceae.g__Thermus.s__thermophilus.t__HB8
d__Bacteria.p__Thermi.c__Thermi.o__Thermales.f__Thermaceae.g__Thermus.s__thermophilus.t__HB27
d__Bacteria.p__Thermi.c__Thermi.o__Thermales.f__Thermaceae.g__Meiothermus.s__silvanus.t__DSM_9946
d__Bacteria.p__Thermi.c__Thermi.o__Thermales.f__Thermaceae.g__Meiothermus.s__ruber.t__DSM_1279
d__Bacteria.p__Thermi.c__Deinococci.o__Deinococcales.f__Trueperaceae.g__Truepera.s__radiovictrix.t__DSM_17093
d__Bacteria.p__Thermi.c__Deinococci.o__Deinococcales.f__Deinococcaceae.g__Deinococcus.s__maricopensis.t__DSM_21211
d__Bacteria.p__Thermi.c__Deinococci.o__Deinococcales.f__Deinococcaceae.g__Deinococcus.s__proteolyticus.t__MRP
d__Bacteria.p__Thermi.c__Deinococci.o__Deinococcales.f__Deinococcaceae.g__Deinococcus.s__geothermalis.t__DSM_11300
d__Bacteria.p__Thermi.c__Deinococci.o__Deinococcales.f__Deinococcaceae.g__Deinococcus.s__deserti.t__VCD115
d__Bacteria.p__Thermi.c__Deinococci.o__Deinococcales.f__Deinococcaceae.g__Deinococcus.s__radiodurans.t__R1
SAR202_cluster_bacterium_sp._SCGC_AAA240_N13
bin__224_10132015
Dehalococcoidia_bacterium_DscP2
Dehalococcoidia_bacterium_SCGC_AB_539_J10
Dehalococcoidia_bacterium_Dsc1
Dehalogenimonas_lykanthroporepellens_BL_DC_9
d__Bacteria.p__Chloroflexi.c__Dehalococcoidetes.o__Dehalococcoidales.f__Dehalogenimonaceae.g__Dehalogenimonas.s__lykanthroporepellens.t__BL_DC_9
Dehalococcoides_mccartyi_BTF08
Dehalococcoides_mccartyi_BAV1
Dehalococcoides_mccartyi_GT
d__Bacteria.p__Chloroflexi.c__Dehalococcoidetes.o__Dehalococcoidales.f__Dehalococcoidaceae.g__Dehalococcoides.s__ethenogenes.t__195
d__Bacteria.p__Chloroflexi.c__Dehalococcoidetes.o__Dehalococcoidales.f__Dehalococcoidaceae.g__Dehalococcoides.s__sp_.t__VS
d__Bacteria.p__Chloroflexi.c__Dehalococcoidetes.o__Dehalococcoidales.f__Dehalococcoidaceae.g__Dehalococcoides.s__sp_.t__BAV1
d__Bacteria.p__Chloroflexi.c__Dehalococcoidetes.o__Dehalococcoidales.f__Dehalococcoidaceae.g__Dehalococcoides.s__sp_.t__CBDB1
d__Bacteria.p__Chloroflexi.c__Dehalococcoidetes.o__Dehalococcoidales.f__Dehalococcoidaceae.g__Dehalococcoides.s__sp_.t__GT
Dehalococcoides_mccartyi_DCMB5
Dehalococcoides_mccartyi_CBDB1
Dehalococcoides_mccartyi_CBDB1__re_annotation_
Dehalococcoides_mccartyi_SG1
Dehalococcoides_mccartyi_195__re_annotation_
Dehalococcoides_mccartyi_195
Dehalococcoides_mccartyi_VS
Dehalococcoides_mccartyi_GY50

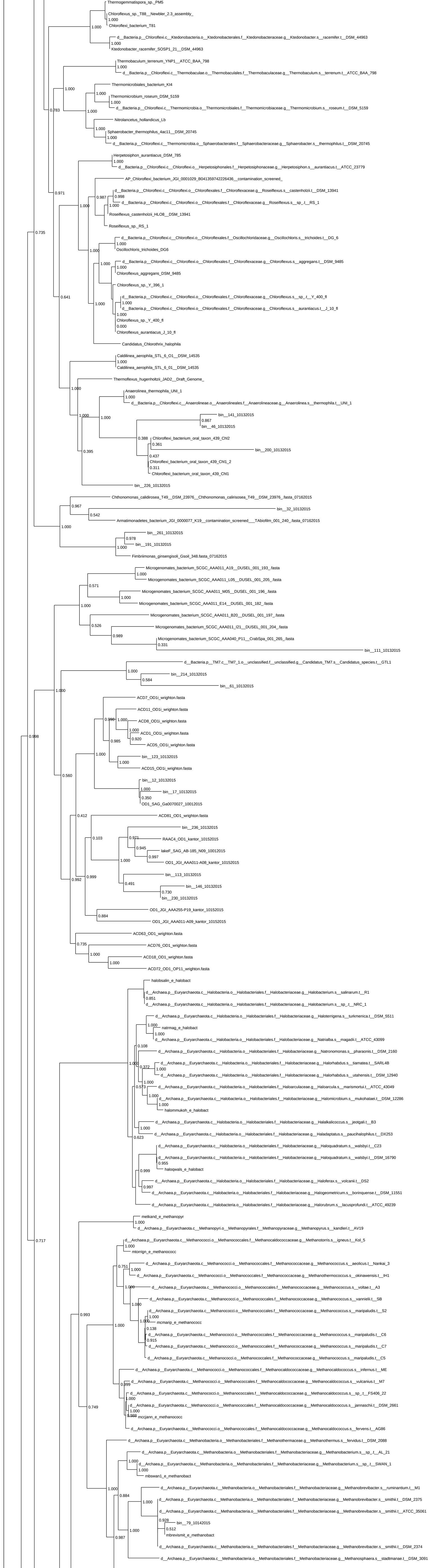

Thermogemmatispora_sp._PM5
Chloroflexus_sp._T88__Newbler_2.3_assembly_
1.000
Chloroflexi_bacterium_T81
1.000
d__Bacteria.p__Chloroflexi.c__Ktedonobacteria.o__Ktedonobacterales.f__Ktedonobacteraceae.g__Ktedonobacter.s__racemifer.t__DSM_44963
1.000
Ktedonobacter_racemifer_SOSP1_21__DSM_44963
Thermobaculum_terrenum_YNP1__ATCC_BAA_798
1.000
d__Bacteria.p__Chloroflexi.c__Thermobaculae.o__Thermobaculales.f__Thermobaculaceae.g__Thermobaculum.s__terrenum.t__ATCC_BAA_798
1.000
Thermomicrobiales_bacterium_Ki4
1.000
Thermomicrobium_roseum_DSM_5159
1.000
0.783
1.000
d__Bacteria.p__Chloroflexi.c__Thermomicrobia.o__Thermomicrobiales.f__Thermomicrobiaceae.g__Thermomicrobium.s__roseum.t__DSM_5159
Nitrolancetus_hollandicus_Lb
1.000
Sphaerobacter_thermophilus_4ac11__DSM_20745
1.000
d__Bacteria.p__Chloroflexi.c__Thermomicrobia.o__Sphaerobacterales.f__Sphaerobacteraceae.g__Sphaerobacter.s__thermophilus.t__DSM_20745
Herpetosiphon_aurantiacus_DSM_785
1.000
d__Bacteria.p__Chloroflexi.c__Chloroflexi.o__Herpetosiphonales.f__Herpetosiphonaceae.g__Herpetosiphon.s__aurantiacus.t__ATCC_23779
AP_Chloroflexi_bacterium_JGI_0001029_B041359742226436__contamination_screened_
d__Bacteria.p__Chloroflexi.c__Chloroflexi.o__Chloroflexales.f__Chloroflexaceae.g__Roseiflexus.s__castenholzii.t__DSM_13941
0.971
0.987
0.998
d__Bacteria.p__Chloroflexi.c__Chloroflexi.o__Chloroflexales.f__Chloroflexaceae.g__Roseiflexus.s__sp_.t__RS_1
1.000
1.000
Roseiflexus_castenholzii_HLO8__DSM_13941
Roseiflexus_sp._RS_1
0.735
d__Bacteria.p__Chloroflexi.c__Chloroflexi.o__Chloroflexales.f__Oscillochloridaceae.g__Oscillochloris.s__trichoides.t__DG_6
1.000
1.000
Oscillochloris_trichoides_DG6
1.000
d__Bacteria.p__Chloroflexi.c__Chloroflexi.o__Chloroflexales.f__Chloroflexaceae.g__Chloroflexus.s__aggregans.t__DSM_9485
1.000
Chloroflexus_aggregans_DSM_9485
Chloroflexus_sp._Y_396_1
d__Bacteria.p__Chloroflexi.c__Chloroflexi.o__Chloroflexales.f__Chloroflexaceae.g__Chloroflexus.s__sp_.t__Y_400_fl
0.641
1.000
1.000
d__Bacteria.p__Chloroflexi.c__Chloroflexi.o__Chloroflexales.f__Chloroflexaceae.g__Chloroflexus.s__aurantiacus.t__J_10_fl
1.000
Chloroflexus_sp._Y_400_fl
0.000
Chloroflexus_aurantiacus_J_10_fl
Candidatus_Chlorothrix_halophila
Caldilinea_aerophila_STL_6_O1__DSM_14535
1.000
Caldilinea_aerophila_STL_6_01__DSM_14535
Thermoflexus_hugenholtzii_JAD2__Draft_Genome_
1.000
Anaerolinea_thermophila_UNI_1
1.000
d__Bacteria.p__Chloroflexi.c__Anaerolineae.o__Anaerolineales.f__Anaerolineaceae.g__Anaerolinea.s__thermophila.t__UNI_1
1.000
1.000
bin__141_10132015
0.867
bin__46_10132015
0.388
Chloroflexi_bacterium_oral_taxon_439_Chl2
0.361
bin__200_10132015
0.395
0.437
Chloroflexi_bacterium_oral_taxon_439_Chl1_2
0.311
Chloroflexi_bacterium_oral_taxon_439_Chl1
bin__226_10132015
Chthonomonas_calidirosea_T49__DSM_23976__Chthonomonas_calirisosea_T49__DSM_23976_.fasta_07162015
0.967
bin__32_10132015
0.542
Armatimonadetes_bacterium_JGI_0000077_K19__contamination_screened___TAbiofilm_001_240_.fasta_07162015
1.000
bin__261_10132015
0.978
bin__191_10132015
1.000
Fimbriimonas_ginsengisoli_Gsoil_348.fasta_07162015
Microgenomates_bacterium_SCGC_AAA011_A19__DUSEL_001_193_.fasta
1.000
Microgenomates_bacterium_SCGC_AAA011_L05__DUSEL_001_205_.fasta
0.571
Microgenomates_bacterium_SCGC_AAA011_M05__DUSEL_001_196_.fasta
1.000
Microgenomates_bacterium_SCGC_AAA011_E14__DUSEL_001_182_.fasta
1.000
Microgenomates_bacterium_SCGC_AAA011_B20__DUSEL_001_197_.fasta
0.526
Microgenomates_bacterium_SCGC_AAA011_I21__DUSEL_001_204_.fasta
0.989
Microgenomates_bacterium_SCGC_AAA040_P11__CrabSpa_001_265_.fasta
0.331
bin__111_10132015
d__Bacteria.p__TM7.c__TM7_1.o__unclassified.f__unclassified.g__Candidatus_TM7.s__Candidatus_species.t__GTL1
1.000
bin__214_10132015
0.584
bin__61_10132015
1.000
ACD7_OD1i_wrighton.fasta
ACD11_OD1i_wrighton.fasta
0.998
1.000
ACD8_OD1i_wrighton.fasta
0.998
1.000
ACD1_OD1i_wrighton.fasta
0.985
0.920
ACD5_OD1i_wrighton.fasta
1.000
bin__123_10132015
1.000
ACD15_OD1i_wrighton.fasta
0.560
bin__12_10132015
1.000
bin__17_10132015
0.350
OD1_SAG_Ga0070027_10012015
ACD81_OD1_wrighton.fasta
0.412
bin__236_10132015
0.103
0.971
RAAC4_OD1_kantor_10152015
0.945
lakeF_SAG_AB-185_N09_10012015
1.000
0.997
OD1_JGI_AAA011-A08_kantor_10152015
0.999
bin__113_10132015
0.992
0.491
bin__146_10132015
0.730
bin__230_10132015
OD1_JGI_AAA255-P19_kantor_10152015
0.884
OD1_JGI_AAA011-A09_kantor_10152015
ACD63_OD1_wrighton.fasta
0.735
ACD76_OD1_wrighton.fasta
1.000
ACD18_OD1_wrighton.fasta
1.000
ACD72_OD1_OP11_wrighton.fasta
halobsalin_e_halobact
d__Archaea.p__Euryarchaeota.c__Halobacteria.o__Halobacteriales.f__Halobacteriaceae.g__Halobacterium.s__salinarum.t__R1
0.851
d__Archaea.p__Euryarchaeota.c__Halobacteria.o__Halobacteriales.f__Halobacteriaceae.g__Halobacterium.s__sp_.t__NRC_1
d__Archaea.p__Euryarchaeota.c__Halobacteria.o__Halobacteriales.f__Halobacteriaceae.g__Haloterrigena.s__turkmenica.t__DSM_5511
1.000
natrmag_e_halobact
1.000
d__Archaea.p__Euryarchaeota.c__Halobacteria.o__Halobacteriales.f__Halobacteriaceae.g__Natrialba.s__magadii.t__ATCC_43099
0.108
d__Archaea.p__Euryarchaeota.c__Halobacteria.o__Halobacteriales.f__Halobacteriaceae.g__Natronomonas.s__pharaonis.t__DSM_2160
d__Archaea.p__Euryarchaeota.c__Halobacteria.o__Halobacteriales.f__Halobacteriaceae.g__Halorhabdus.s__tiamatea.t__SARL4B
1.000
0.372
1.000
d__Archaea.p__Euryarchaeota.c__Halobacteria.o__Halobacteriales.f__Halobacteriaceae.g__Halorhabdus.s__utahensis.t__DSM_12940
1.000
0.573
d__Archaea.p__Euryarchaeota.c__Halobacteria.o__Halobacteriales.f__Haloarculaceae.g__Haloarcula.s__marismortui.t__ATCC_43049
1.000
d__Archaea.p__Euryarchaeota.c__Halobacteria.o__Halobacteriales.f__Halobacteriaceae.g__Halomicrobium.s__mukohataei.t__DSM_12286
1.000
halommukoh_e_halobact
d__Archaea.p__Euryarchaeota.c__Halobacteria.o__Halobacteriales.f__Halobacteriaceae.g__Halalkalicoccus.s__jeotgali.t__B3
1.000
d__Archaea.p__Euryarchaeota.c__Halobacteria.o__Halobacteriales.f__Halobacteriaceae.g__Haladaptatus.s__paucihalophilus.t__DX253
0.623
d__Archaea.p__Euryarchaeota.c__Halobacteria.o__Halobacteriales.f__Halobacteriaceae.g__Haloquadratum.s__walsbyi.t__C23
0.998
d__Archaea.p__Euryarchaeota.c__Halobacteria.o__Halobacteriales.f__Halobacteriaceae.g__Haloquadratum.s__walsbyi.t__DSM_16790
0.955
haloqwals_e_halobact
0.999
d__Archaea.p__Euryarchaeota.c__Halobacteria.o__Halobacteriales.f__Halobacteriaceae.g__Haloferax.s__volcanii.t__DS2
0.997
d__Archaea.p__Euryarchaeota.c__Halobacteria.o__Halobacteriales.f__Halobacteriaceae.g__Halogeometricum.s__borinquense.t__DSM_11551
d__Archaea.p__Euryarchaeota.c__Halobacteria.o__Halobacteriales.f__Halobacteriaceae.g__Halorubrum.s__lacusprofundi.t__ATCC_49239
metkand_e_methanopyr
1.000
d__Archaea.p__Euryarchaeota.c__Methanopyri.o__Methanopyrales.f__Methanopyraceae.g__Methanopyrus.s__kandleri.t__AV19
0.717
d__Archaea.p__Euryarchaeota.c__Methanococci.o__Methanococcales.f__Methanocaldococcaceae.g__Methanotorris.s__igneus.t__Kol_5
1.000
mtorrign_e_methanococc
d__Archaea.p__Euryarchaeota.c__Methanococci.o__Methanococcales.f__Methanococcaceae.g__Methanococcus.s__aeolicus.t__Nankai_3
0.751
1.000
d__Archaea.p__Euryarchaeota.c__Methanococci.o__Methanococcales.f__Methanococcaceae.g__Methanothermococcus.s__okinawensis.t__IH1
1.000
d__Archaea.p__Euryarchaeota.c__Methanococci.o__Methanococcales.f__Methanococcaceae.g__Methanococcus.s__voltae.t__A3
d__Archaea.p__Euryarchaeota.c__Methanococci.o__Methanococcales.f__Methanococcaceae.g__Methanococcus.s__vannielii.t__SB
1.000
d__Archaea.p__Euryarchaeota.c__Methanococci.o__Methanococcales.f__Methanococcaceae.g__Methanococcus.s__maripaludis.t__S2
0.993
1.000
1.000
mcmarip_e_methanococc
1.000
0.138
d__Archaea.p__Euryarchaeota.c__Methanococci.o__Methanococcales.f__Methanococcaceae.g__Methanococcus.s__maripaludis.t__C6
0.915
d__Archaea.p__Euryarchaeota.c__Methanococci.o__Methanococcales.f__Methanococcaceae.g__Methanococcus.s__maripaludis.t__C7
d__Archaea.p__Euryarchaeota.c__Methanococci.o__Methanococcales.f__Methanococcaceae.g__Methanococcus.s__maripaludis.t__C5
d__Archaea.p__Euryarchaeota.c__Methanococci.o__Methanococcales.f__Methanocaldococcaceae.g__Methanocaldococcus.s__infernus.t__ME
0.999
d__Archaea.p__Euryarchaeota.c__Methanococci.o__Methanococcales.f__Methanocaldococcaceae.g__Methanocaldococcus.s__vulcanius.t__M7
d__Archaea.p__Euryarchaeota.c__Methanococci.o__Methanococcales.f__Methanocaldococcaceae.g__Methanocaldococcus.s__sp_.t__FS406_22
1.000
0.749
d__Archaea.p__Euryarchaeota.c__Methanococci.o__Methanococcales.f__Methanocaldococcaceae.g__Methanocaldococcus.s__jannaschii.t__DSM_2661
1.000
0.988
mccjann_e_methanococc
d__Archaea.p__Euryarchaeota.c__Methanococci.o__Methanococcales.f__Methanocaldococcaceae.g__Methanocaldococcus.s__fervens.t__AG86
d__Archaea.p__Euryarchaeota.c__Methanobacteria.o__Methanobacteriales.f__Methanothermaceae.g__Methanothermus.s__fervidus.t__DSM_2088
d__Archaea.p__Euryarchaeota.c__Methanobacteria.o__Methanobacteriales.f__Methanobacteriaceae.g__Methanobacterium.s__sp_.t__AL_21
1.000
d__Archaea.p__Euryarchaeota.c__Methanobacteria.o__Methanobacteriales.f__Methanobacteriaceae.g__Methanobacterium.s__sp_.t__SWAN_1
1.000
mbswan1_e_methanobact
1.000
d__Archaea.p__Euryarchaeota.c__Methanobacteria.o__Methanobacteriales.f__Methanobacteriaceae.g__Methanobrevibacter.s__ruminantium.t__M1
0.884
1.000
d__Archaea.p__Euryarchaeota.c__Methanobacteria.o__Methanobacteriales.f__Methanobacteriaceae.g__Methanobrevibacter.s__smithii.t__DSM_2375
d__Archaea.p__Euryarchaeota.c__Methanobacteria.o__Methanobacteriales.f__Methanobacteriaceae.g__Methanobrevibacter.s__smithii.t__ATCC_35061
0.928
bin__79_10142015
1.000
0.512
mbrevismit_e_methanobact
0.987
d__Archaea.p__Euryarchaeota.c__Methanobacteria.o__Methanobacteriales.f__Methanobacteriaceae.g__Methanobrevibacter.s__smithii.t__DSM_2374
d__Archaea.p__Euryarchaeota.c__Methanobacteria.o__Methanobacteriales.f__Methanobacteriaceae.g__Methanosphaera.s__stadtmanae.t__DSM_3091

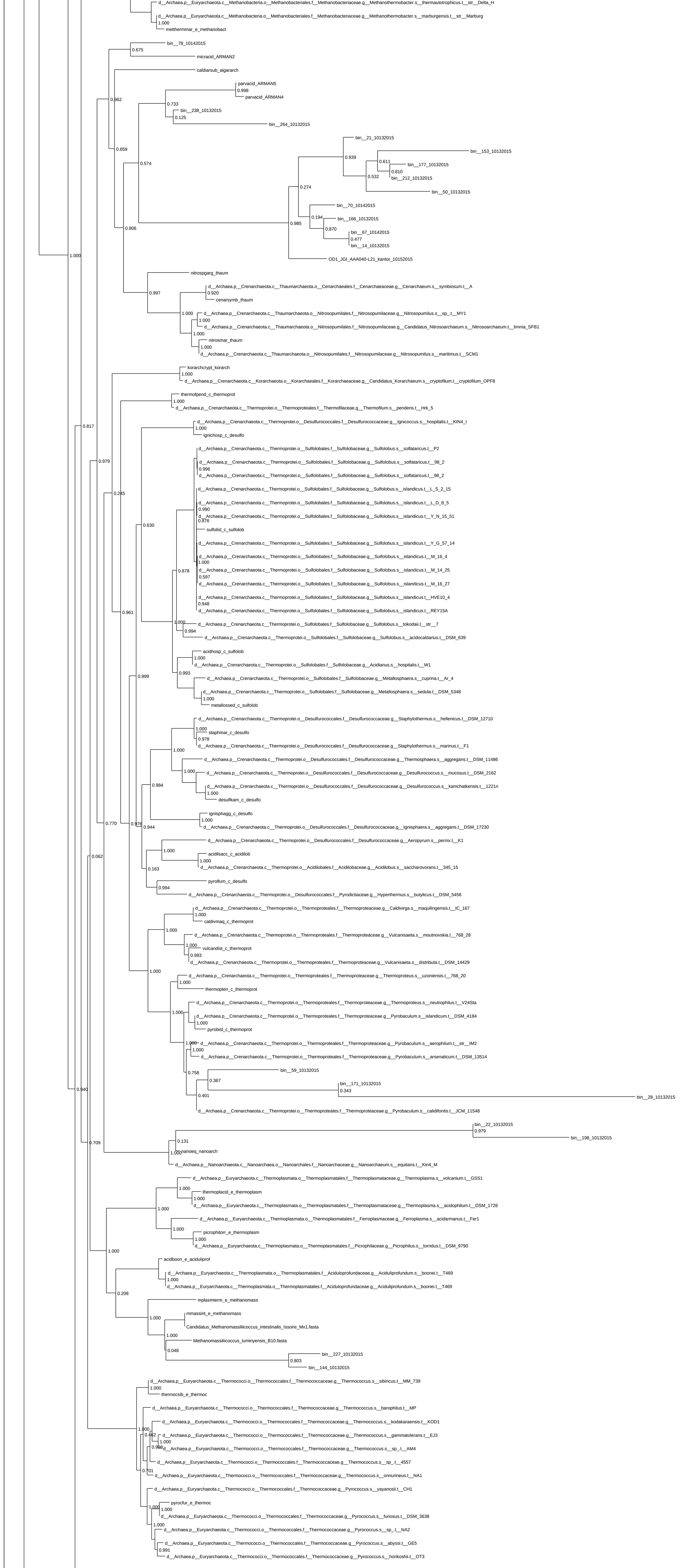
d__Archaea.p__Euryarchaeota.c__Methanobacteria.o__Methanobacteriales.f__Methanobacteriaceae.g__Methanothermobacter.s__thermautotrophicus.t__str__Delta_H
d__Archaea.p__Euryarchaeota.c__Methanobacteria.o__Methanobacteriales.f__Methanobacteriaceae.g__Methanothermobacter.s__marburgensis.t__str__Marburg
metthermmar_e_methanobact
bin__78_10142015
micracid_ARMAN2
caldiarsub_aigararch
parvacid_ARMAN5
parvacid_ARMAN4
bin__238_10132015
bin__264_10132015
bin__21_10132015
bin__153_10132015
bin__177_10132015
bin__212_10132015
bin__50_10132015
bin__70_10142015
bin__166_10132015
bin__67_10142015
bin__14_10132015
OD1_JGI_AAA040-L21_kantor_10152015
nitrospgarg_thaum
d__Archaea.p__Crenarchaeota.c__Thaumarchaeota.o__Cenarchaeales.f__Cenarchaeaceae.g__Cenarchaeum.s__symbiosum.t__A
cenarsymb_thaum
d__Archaea.p__Crenarchaeota.c__Thaumarchaeota.o__Nitrosopumilales.f__Nitrosopumilaceae.g__Nitrosopumilus.s__sp_.t__MY1
d__Archaea.p__Crenarchaeota.c__Thaumarchaeota.o__Nitrosopumilales.f__Nitrosopumilaceae.g__Candidatus_Nitrosoarchaeum.s__Nitrosoarchaeum.t__limnia_SFB1
nitrosmar_thaum
d__Archaea.p__Crenarchaeota.c__Thaumarchaeota.o__Nitrosopumilales.f__Nitrosopumilaceae.g__Nitrosopumilus.s__maritimus.t__SCM1
korarchcrypt_korarch
d__Archaea.p__Crenarchaeota.c__Korarchaeota.o__Korarchaeales.f__Korarchaeaceae.g__Candidatus_Korarchaeum.s__cryptofilum.t__cryptofilum_OPF8
thermofpend_c_thermoprot
d__Archaea.p__Crenarchaeota.c__Thermoprotei.o__Thermoproteales.f__Thermofilaceae.g__Thermofilum.s__pendens.t__Hrk_5
d__Archaea.p__Crenarchaeota.c__Thermoprotei.o__Desulfurococcales.f__Desulfurococcaceae.g__Ignicoccus.s__hospitalis.t__KIN4_I
ignichosp_c_desulfo
d__Archaea.p__Crenarchaeota.c__Thermoprotei.o__Sulfolobales.f__Sulfolobaceae.g__Sulfolobus.s__solfataricus.t__P2
d__Archaea.p__Crenarchaeota.c__Thermoprotei.o__Sulfolobales.f__Sulfolobaceae.g__Sulfolobus.s__solfataricus.t__98_2
d__Archaea.p__Crenarchaeota.c__Thermoprotei.o__Sulfolobales.f__Sulfolobaceae.g__Sulfolobus.s__solfataricus.t__98_2
d__Archaea.p__Crenarchaeota.c__Thermoprotei.o__Sulfolobales.f__Sulfolobaceae.g__Sulfolobus.s__islandicus.t__L_S_2_15
d__Archaea.p__Crenarchaeota.c__Thermoprotei.o__Sulfolobales.f__Sulfolobaceae.g__Sulfolobus.s__islandicus.t__L_D_8_5
d__Archaea.p__Crenarchaeota.c__Thermoprotei.o__Sulfolobales.f__Sulfolobaceae.g__Sulfolobus.s__islandicus.t__Y_N_15_51
sulfolisl_c_sulfolob
d__Archaea.p__Crenarchaeota.c__Thermoprotei.o__Sulfolobales.f__Sulfolobaceae.g__Sulfolobus.s__islandicus.t__Y_G_57_14
d__Archaea.p__Crenarchaeota.c__Thermoprotei.o__Sulfolobales.f__Sulfolobaceae.g__Sulfolobus.s__islandicus.t__M_16_4
d__Archaea.p__Crenarchaeota.c__Thermoprotei.o__Sulfolobales.f__Sulfolobaceae.g__Sulfolobus.s__islandicus.t__M_14_25
d__Archaea.p__Crenarchaeota.c__Thermoprotei.o__Sulfolobales.f__Sulfolobaceae.g__Sulfolobus.s__islandicus.t__M_16_27
d__Archaea.p__Crenarchaeota.c__Thermoprotei.o__Sulfolobales.f__Sulfolobaceae.g__Sulfolobus.s__islandicus.t__HVE10_4
d__Archaea.p__Crenarchaeota.c__Thermoprotei.o__Sulfolobales.f__Sulfolobaceae.g__Sulfolobus.s__islandicus.t__REY15A
d__Archaea.p__Crenarchaeota.c__Thermoprotei.o__Sulfolobales.f__Sulfolobaceae.g__Sulfolobus.s__tokodaii.t__str__7
d__Archaea.p__Crenarchaeota.c__Thermoprotei.o__Sulfolobales.f__Sulfolobaceae.g__Sulfolobus.s__acidocaldarius.t__DSM_639
acidhosp_c_sulfolob
d__Archaea.p__Crenarchaeota.c__Thermoprotei.o__Sulfolobales.f__Sulfolobaceae.g__Acidianus.s__hospitalis.t__W1
d__Archaea.p__Crenarchaeota.c__Thermoprotei.o__Sulfolobales.f__Sulfolobaceae.g__Metallosphaera.s__cuprina.t__Ar_4
d__Archaea.p__Crenarchaeota.c__Thermoprotei.o__Sulfolobales.f__Sulfolobaceae.g__Metallosphaera.s__sedula.t__DSM_5348
metallossed_c_sulfolob
d__Archaea.p__Crenarchaeota.c__Thermoprotei.o__Desulfurococcales.f__Desulfurococcaceae.g__Staphylothermus.s__hellenicus.t__DSM_12710
staphmar_c_desulfo
d__Archaea.p__Crenarchaeota.c__Thermoprotei.o__Desulfurococcales.f__Desulfurococcaceae.g__Staphylothermus.s__marinus.t__F1
d__Archaea.p__Crenarchaeota.c__Thermoprotei.o__Desulfurococcales.f__Desulfurococcaceae.g__Thermosphaera.s__aggregans.t__DSM_11486
d__Archaea.p__Crenarchaeota.c__Thermoprotei.o__Desulfurococcales.f__Desulfurococcaceae.g__Desulfurococcus.s__mucosus.t__DSM_2162
d__Archaea.p__Crenarchaeota.c__Thermoprotei.o__Desulfurococcales.f__Desulfurococcaceae.g__Desulfurococcus.s__kamchatkensis.t__1221n
desulfkam_c_desulfo
ignisphagg_c_desulfo
d__Archaea.p__Crenarchaeota.c__Thermoprotei.o__Desulfurococcales.f__Desulfurococcaceae.g__Ignisphaera.s__aggregans.t__DSM_17230
d__Archaea.p__Crenarchaeota.c__Thermoprotei.o__Desulfurococcales.f__Desulfurococcaceae.g__Aeropyrum.s__pernix.t__K1
acidilsacc_c_acidilob
d__Archaea.p__Crenarchaeota.c__Thermoprotei.o__Acidilobales.f__Acidilobaceae.g__Acidilobus.s__saccharovorans.t__345_15
pyrofum_c_desulfo
d__Archaea.p__Crenarchaeota.c__Thermoprotei.o__Desulfurococcales.f__Pyrodictiaceae.g__Hyperthermus.s__butylicus.t__DSM_5456
d__Archaea.p__Crenarchaeota.c__Thermoprotei.o__Thermoproteales.f__Thermoproteaceae.g__Caldivirga.s__maquilingensis.t__IC_167
caldivmaq_c_thermoprot
d__Archaea.p__Crenarchaeota.c__Thermoprotei.o__Thermoproteales.f__Thermoproteaceae.g__Vulcanisaeta.s__moutnovskia.t__768_28
vulcandist_c_thermoprot
d__Archaea.p__Crenarchaeota.c__Thermoprotei.o__Thermoproteales.f__Thermoproteaceae.g__Vulcanisaeta.s__distributa.t__DSM_14429
d__Archaea.p__Crenarchaeota.c__Thermoprotei.o__Thermoproteales.f__Thermoproteaceae.g__Thermoproteus.s__uzoniensis.t__768_20
thermopten_c_thermoprot
d__Archaea.p__Crenarchaeota.c__Thermoprotei.o__Thermoproteales.f__Thermoproteaceae.g__Thermoproteus.s__neutrophilus.t__V24Sta
d__Archaea.p__Crenarchaeota.c__Thermoprotei.o__Thermoproteales.f__Thermoproteaceae.g__Pyrobaculum.s__islandicum.t__DSM_4184
pyrobisl_c_thermoprot
d__Archaea.p__Crenarchaeota.c__Thermoprotei.o__Thermoproteales.f__Thermoproteaceae.g__Pyrobaculum.s__aerophilum.t__str__IM2
d__Archaea.p__Crenarchaeota.c__Thermoprotei.o__Thermoproteales.f__Thermoproteaceae.g__Pyrobaculum.s__arsenaticum.t__DSM_13514
bin__59_10132015
bin__171_10132015
bin__28_10132015
d__Archaea.p__Crenarchaeota.c__Thermoprotei.o__Thermoproteales.f__Thermoproteaceae.g__Pyrobaculum.s__calidifontis.t__JCM_11548
bin__22_10132015
bin__198_10132015
nanoeq_nanoarch
d__Archaea.p__Nanoarchaeota.c__Nanoarchaea.o__Nanoarchales.f__Nanoarchaceae.g__Nanoarchaeum.s__equitans.t__Kin4_M
d__Archaea.p__Euryarchaeota.c__Thermoplasmata.o__Thermoplasmatales.f__Thermoplasmataceae.g__Thermoplasma.s__volcanium.t__GSS1
thermoplacid_e_thermoplasm
d__Archaea.p__Euryarchaeota.c__Thermoplasmata.o__Thermoplasmatales.f__Thermoplasmataceae.g__Thermoplasma.s__acidophilum.t__DSM_1728
d__Archaea.p__Euryarchaeota.c__Thermoplasmata.o__Thermoplasmatales.f__Ferroplasmaceae.g__Ferroplasma.s__acidarmanus.t__Fer1
picrophitorr_e_thermoplasm
d__Archaea.p__Euryarchaeota.c__Thermoplasmata.o__Thermoplasmatales.f__Picrophilaceae.g__Picrophilus.s__torridus.t__DSM_9790
acidboon_e_aciduliprof
d__Archaea.p__Euryarchaeota.c__Thermoplasmata.o__Thermoplasmatales.f__Aciduloprofundaceae.g__Aciduliprofundum.s__boonei.t__T469
d__Archaea.p__Euryarchaeota.c__Thermoplasmata.o__Thermoplasmatales.f__Aciduloprofundaceae.g__Aciduliprofundum.s__boonei.t__T469
mplasmterm_e_methanomass
mmassint_e_methanomass
Candidatus_Methanomassiliicoccus_intestinalis_Issoire_Mx1.fasta
Methanomassiliicoccus_luminyensis_B10.fasta
bin__227_10132015
bin__144_10132015
d__Archaea.p__Euryarchaeota.c__Thermococci.o__Thermococcales.f__Thermococcaceae.g__Thermococcus.s__sibiricus.t__MM_739
thermocsib_e_thermoc
d__Archaea.p__Euryarchaeota.c__Thermococci.o__Thermococcales.f__Thermococcaceae.g__Thermococcus.s__barophilus.t__MP
d__Archaea.p__Euryarchaeota.c__Thermococci.o__Thermococcales.f__Thermococcaceae.g__Thermococcus.s__kodakaraensis.t__KOD1
d__Archaea.p__Euryarchaeota.c__Thermococci.o__Thermococcales.f__Thermococcaceae.g__Thermococcus.s__gammatolerans.t__EJ3
d__Archaea.p__Euryarchaeota.c__Thermococci.o__Thermococcales.f__Thermococcaceae.g__Thermococcus.s__sp_.t__AM4
d__Archaea.p__Euryarchaeota.c__Thermococci.o__Thermococcales.f__Thermococcaceae.g__Thermococcus.s__sp_.t__4557
d__Archaea.p__Euryarchaeota.c__Thermococci.o__Thermococcales.f__Thermococcaceae.g__Thermococcus.s__onnurineus.t__NA1
d__Archaea.p__Euryarchaeota.c__Thermococci.o__Thermococcales.f__Thermococcaceae.g__Pyrococcus.s__yayanosii.t__CH1
pyrocfur_e_thermoc
d__Archaea.p__Euryarchaeota.c__Thermococci.o__Thermococcales.f__Thermococcaceae.g__Pyrococcus.s__furiosus.t__DSM_3638
d__Archaea.p__Euryarchaeota.c__Thermococci.o__Thermococcales.f__Thermococcaceae.g__Pyrococcus.s__sp_.t__NA2
d__Archaea.p__Euryarchaeota.c__Thermococci.o__Thermococcales.f__Thermococcaceae.g__Pyrococcus.s__abyssi.t__GE5
d__Archaea.p__Euryarchaeota.c__Thermococci.o__Thermococcales.f__Thermococcaceae.g__Pyrococcus.s__horikoshii.t__OT3

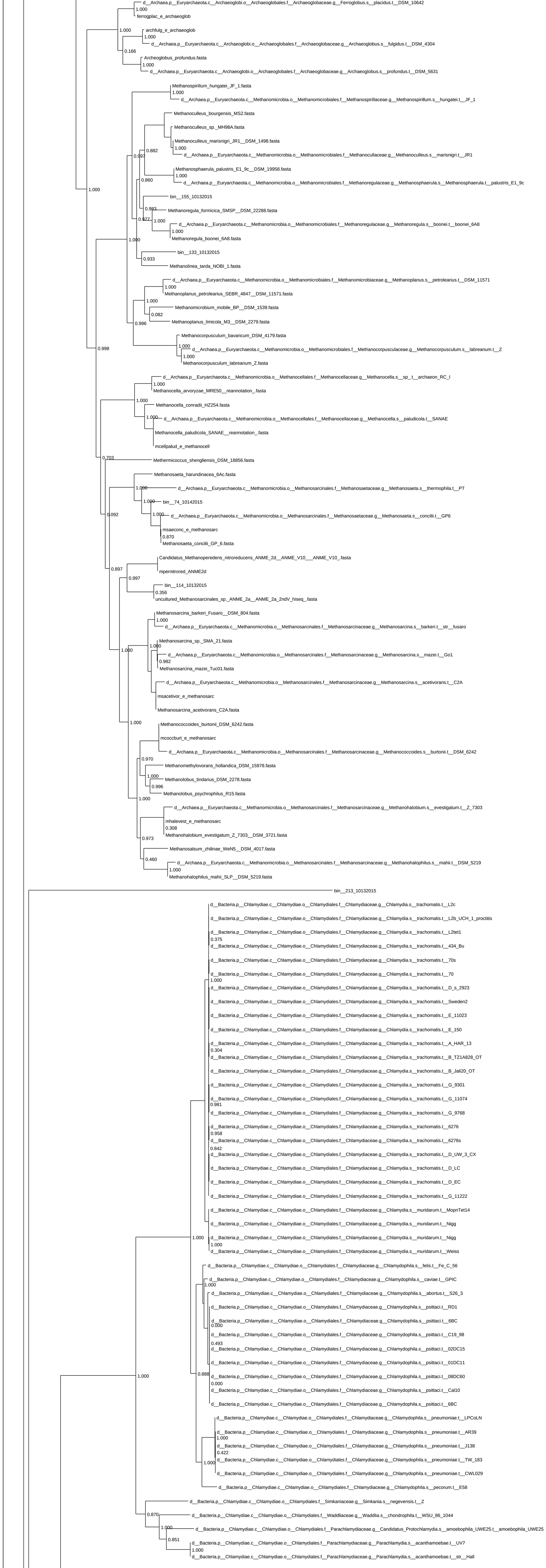

d__Archaea.p__Euryarchaeota.c__Archaeoglobi.o__Archaeoglobales.f__Archaeoglobaceae.g__Ferroglobus.s__placidus.t__DSM_10642
ferrogplac_e_archaeoglob
archfulg_e_archaeoglob
d__Archaea.p__Euryarchaeota.c__Archaeoglobi.o__Archaeoglobales.f__Archaeoglobaceae.g__Archaeoglobus.s__fulgidus.t__DSM_4304
Archeoglobus_profundus.fasta
d__Archaea.p__Euryarchaeota.c__Archaeoglobi.o__Archaeoglobales.f__Archaeoglobaceae.g__Archaeoglobus.s__profundus.t__DSM_5631
Methanospirillum_hungatei_JF_1.fasta
d__Archaea.p__Euryarchaeota.c__Methanomicrobia.o__Methanomicrobiales.f__Methanospirillaceae.g__Methanospirillum.s__hungatei.t__JF_1
Methanoculleus_bourgensis_MS2.fasta
Methanoculleus_sp._MH98A.fasta
Methanoculleus_marisnigri_JR1__DSM_1498.fasta
d__Archaea.p__Euryarchaeota.c__Methanomicrobia.o__Methanomicrobiales.f__Methanocullaceae.g__Methanoculleus.s__marisnigri.t__JR1
Methanosphaerula_palustris_E1_9c__DSM_19958.fasta
d__Archaea.p__Euryarchaeota.c__Methanomicrobia.o__Methanomicrobiales.f__Methanoregulaceae.g__Methanosphaerula.s__Methanosphaerula.t__palustris_E1_9c
bin__155_10132015
Methanoregula_formicica_SMSP__DSM_22288.fasta
d__Archaea.p__Euryarchaeota.c__Methanomicrobia.o__Methanomicrobiales.f__Methanoregulaceae.g__Methanoregula.s__boonei.t__boonei_6A8
Methanoregula_boonei_6A8.fasta
bin__133_10132015
Methanolinea_tarda_NOBI_1.fasta
d__Archaea.p__Euryarchaeota.c__Methanomicrobia.o__Methanomicrobiales.f__Methanomicrobiaceae.g__Methanoplanus.s__petrolearius.t__DSM_11571
Methanoplanus_petrolearius_SEBR_4847__DSM_11571.fasta
Methanomicrobium_mobile_BP__DSM_1539.fasta
Methanoplanus_limicola_M3__DSM_2279.fasta
Methanocorpusculum_bavaricum_DSM_4179.fasta
d__Archaea.p__Euryarchaeota.c__Methanomicrobia.o__Methanomicrobiales.f__Methanocorpusculaceae.g__Methanocorpusculum.s__labreanum.t__Z
Methanocorpusculum_labreanum_Z.fasta
d__Archaea.p__Euryarchaeota.c__Methanomicrobia.o__Methanocellales.f__Methanocellaceae.g__Methanocella.s__sp._t__archaeon_RC_I
Methanocella_arvoryzae_MRE50__reannotation_.fasta
Methanocella_conradii_HZ254.fasta
d__Archaea.p__Euryarchaeota.c__Methanomicrobia.o__Methanocellales.f__Methanocellaceae.g__Methanocella.s__paludicola.t__SANAE
Methanocella_paludicola_SANAE__reannotation_.fasta
mcellpalud_e_methanocell
Methermicoccus_shengliensis_DSM_18856.fasta
Methanosaeta_harundinacea_6Ac.fasta
d__Archaea.p__Euryarchaeota.c__Methanomicrobia.o__Methanosarcinales.f__Methanosaetaceae.g__Methanosaeta.s__thermophila.t__PT
bin__74_10142015
d__Archaea.p__Euryarchaeota.c__Methanomicrobia.o__Methanosarcinales.f__Methanosaetaceae.g__Methanosaeta.s__concilii.t__GP6
msaeconc_e_methanosarc
Methanosaeta_concilii_GP_6.fasta
Candidatus_Methanoperedens_nitroreducens_ANME_2d__ANME_V10___ANME_V10_.fasta
mpernitrored_ANME2d
bin__114_10132015
uncultured_Methanosarcinales_sp._ANME_2a__ANME_2a_2ndV_hiseq_.fasta
Methanosarcina_barkeri_Fusaro__DSM_804.fasta
d__Archaea.p__Euryarchaeota.c__Methanomicrobia.o__Methanosarcinales.f__Methanosarcinaceae.g__Methanosarcina.s__barkeri.t__str__fusaro
Methanosarcina_sp._SMA_21.fasta
d__Archaea.p__Euryarchaeota.c__Methanomicrobia.o__Methanosarcinales.f__Methanosarcinaceae.g__Methanosarcina.s__mazei.t__Go1
Methanosarcina_mazei_Tuc01.fasta
d__Archaea.p__Euryarchaeota.c__Methanomicrobia.o__Methanosarcinales.f__Methanosarcinaceae.g__Methanosarcina.s__acetivorans.t__C2A
msacetivor_e_methanosarc
Methanosarcina_acetivorans_C2A.fasta
Methanococcoides_burtonii_DSM_6242.fasta
mcoccburt_e_methanosarc
d__Archaea.p__Euryarchaeota.c__Methanomicrobia.o__Methanosarcinales.f__Methanosarcinaceae.g__Methanococcoides.s__burtonii.t__DSM_6242
Methanomethylovorans_hollandica_DSM_15978.fasta
Methanolobus_tindarius_DSM_2278.fasta
Methanolobus_psychrophilus_R15.fasta
d__Archaea.p__Euryarchaeota.c__Methanomicrobia.o__Methanosarcinales.f__Methanosarcinaceae.g__Methanohalobium.s__evestigatum.t__Z_7303
mhalevest_e_methanosarc
Methanohalobium_evestigatum_Z_7303__DSM_3721.fasta
Methanosalsum_zhilinae_WeN5__DSM_4017.fasta
d__Archaea.p__Euryarchaeota.c__Methanomicrobia.o__Methanosarcinales.f__Methanosarcinaceae.g__Methanohalophilus.s__mahii.t__DSM_5219
Methanohalophilus_mahii_SLP__DSM_5219.fasta
bin__213_10132015
d__Bacteria.p__Chlamydiae.c__Chlamydiae.o__Chlamydiales.f__Chlamydiaceae.g__Chlamydia.s__trachomatis.t__L2c
d__Bacteria.p__Chlamydiae.c__Chlamydiae.o__Chlamydiales.f__Chlamydiaceae.g__Chlamydia.s__trachomatis.t__L2b_UCH_1_proctitis
d__Bacteria.p__Chlamydiae.c__Chlamydiae.o__Chlamydiales.f__Chlamydiaceae.g__Chlamydia.s__trachomatis.t__L2tet1
d__Bacteria.p__Chlamydiae.c__Chlamydiae.o__Chlamydiales.f__Chlamydiaceae.g__Chlamydia.s__trachomatis.t__434_Bu
d__Bacteria.p__Chlamydiae.c__Chlamydiae.o__Chlamydiales.f__Chlamydiaceae.g__Chlamydia.s__trachomatis.t__70s
d__Bacteria.p__Chlamydiae.c__Chlamydiae.o__Chlamydiales.f__Chlamydiaceae.g__Chlamydia.s__trachomatis.t__70
d__Bacteria.p__Chlamydiae.c__Chlamydiae.o__Chlamydiales.f__Chlamydiaceae.g__Chlamydia.s__trachomatis.t__D_s_2923
d__Bacteria.p__Chlamydiae.c__Chlamydiae.o__Chlamydiales.f__Chlamydiaceae.g__Chlamydia.s__trachomatis.t__Sweden2
d__Bacteria.p__Chlamydiae.c__Chlamydiae.o__Chlamydiales.f__Chlamydiaceae.g__Chlamydia.s__trachomatis.t__E_11023
d__Bacteria.p__Chlamydiae.c__Chlamydiae.o__Chlamydiales.f__Chlamydiaceae.g__Chlamydia.s__trachomatis.t__E_150
d__Bacteria.p__Chlamydiae.c__Chlamydiae.o__Chlamydiales.f__Chlamydiaceae.g__Chlamydia.s__trachomatis.t__A_HAR_13
d__Bacteria.p__Chlamydiae.c__Chlamydiae.o__Chlamydiales.f__Chlamydiaceae.g__Chlamydia.s__trachomatis.t__B_TZ1A828_OT
d__Bacteria.p__Chlamydiae.c__Chlamydiae.o__Chlamydiales.f__Chlamydiaceae.g__Chlamydia.s__trachomatis.t__B_Jali20_OT
d__Bacteria.p__Chlamydiae.c__Chlamydiae.o__Chlamydiales.f__Chlamydiaceae.g__Chlamydia.s__trachomatis.t__G_9301
d__Bacteria.p__Chlamydiae.c__Chlamydiae.o__Chlamydiales.f__Chlamydiaceae.g__Chlamydia.s__trachomatis.t__G_11074
d__Bacteria.p__Chlamydiae.c__Chlamydiae.o__Chlamydiales.f__Chlamydiaceae.g__Chlamydia.s__trachomatis.t__G_9768
d__Bacteria.p__Chlamydiae.c__Chlamydiae.o__Chlamydiales.f__Chlamydiaceae.g__Chlamydia.s__trachomatis.t__6276
d__Bacteria.p__Chlamydiae.c__Chlamydiae.o__Chlamydiales.f__Chlamydiaceae.g__Chlamydia.s__trachomatis.t__6276s
d__Bacteria.p__Chlamydiae.c__Chlamydiae.o__Chlamydiales.f__Chlamydiaceae.g__Chlamydia.s__trachomatis.t__D_UW_3_CX
d__Bacteria.p__Chlamydiae.c__Chlamydiae.o__Chlamydiales.f__Chlamydiaceae.g__Chlamydia.s__trachomatis.t__D_LC
d__Bacteria.p__Chlamydiae.c__Chlamydiae.o__Chlamydiales.f__Chlamydiaceae.g__Chlamydia.s__trachomatis.t__D_EC
d__Bacteria.p__Chlamydiae.c__Chlamydiae.o__Chlamydiales.f__Chlamydiaceae.g__Chlamydia.s__trachomatis.t__G_11222
d__Bacteria.p__Chlamydiae.c__Chlamydiae.o__Chlamydiales.f__Chlamydiaceae.g__Chlamydia.s__muridarum.t__MopnTet14
d__Bacteria.p__Chlamydiae.c__Chlamydiae.o__Chlamydiales.f__Chlamydiaceae.g__Chlamydia.s__muridarum.t__Nigg
d__Bacteria.p__Chlamydiae.c__Chlamydiae.o__Chlamydiales.f__Chlamydiaceae.g__Chlamydia.s__muridarum.t__Nigg
d__Bacteria.p__Chlamydiae.c__Chlamydiae.o__Chlamydiales.f__Chlamydiaceae.g__Chlamydia.s__muridarum.t__Weiss
d__Bacteria.p__Chlamydiae.c__Chlamydiae.o__Chlamydiales.f__Chlamydiaceae.g__Chlamydophila.s__felis.t__Fe_C_56
d__Bacteria.p__Chlamydiae.c__Chlamydiae.o__Chlamydiales.f__Chlamydiaceae.g__Chlamydophila.s__caviae.t__GPIC
d__Bacteria.p__Chlamydiae.c__Chlamydiae.o__Chlamydiales.f__Chlamydiaceae.g__Chlamydophila.s__abortus.t__S26_3
d__Bacteria.p__Chlamydiae.c__Chlamydiae.o__Chlamydiales.f__Chlamydiaceae.g__Chlamydophila.s__psittaci.t__RD1
d__Bacteria.p__Chlamydiae.c__Chlamydiae.o__Chlamydiales.f__Chlamydiaceae.g__Chlamydophila.s__psittaci.t__6BC
d__Bacteria.p__Chlamydiae.c__Chlamydiae.o__Chlamydiales.f__Chlamydiaceae.g__Chlamydophila.s__psittaci.t__C19_98
d__Bacteria.p__Chlamydiae.c__Chlamydiae.o__Chlamydiales.f__Chlamydiaceae.g__Chlamydophila.s__psittaci.t__02DC15
d__Bacteria.p__Chlamydiae.c__Chlamydiae.o__Chlamydiales.f__Chlamydiaceae.g__Chlamydophila.s__psittaci.t__01DC11
d__Bacteria.p__Chlamydiae.c__Chlamydiae.o__Chlamydiales.f__Chlamydiaceae.g__Chlamydophila.s__psittaci.t__08DC60
d__Bacteria.p__Chlamydiae.c__Chlamydiae.o__Chlamydiales.f__Chlamydiaceae.g__Chlamydophila.s__psittaci.t__Cal10
d__Bacteria.p__Chlamydiae.c__Chlamydiae.o__Chlamydiales.f__Chlamydiaceae.g__Chlamydophila.s__psittaci.t__6BC
d__Bacteria.p__Chlamydiae.c__Chlamydiae.o__Chlamydiales.f__Chlamydiaceae.g__Chlamydophila.s__pneumoniae.t__LPCoLN
d__Bacteria.p__Chlamydiae.c__Chlamydiae.o__Chlamydiales.f__Chlamydiaceae.g__Chlamydophila.s__pneumoniae.t__AR39
d__Bacteria.p__Chlamydiae.c__Chlamydiae.o__Chlamydiales.f__Chlamydiaceae.g__Chlamydophila.s__pneumoniae.t__J138
d__Bacteria.p__Chlamydiae.c__Chlamydiae.o__Chlamydiales.f__Chlamydiaceae.g__Chlamydophila.s__pneumoniae.t__TW_183
d__Bacteria.p__Chlamydiae.c__Chlamydiae.o__Chlamydiales.f__Chlamydiaceae.g__Chlamydophila.s__pneumoniae.t__CWL029
d__Bacteria.p__Chlamydiae.c__Chlamydiae.o__Chlamydiales.f__Chlamydiaceae.g__Chlamydophila.s__pecorum.t__E58
d__Bacteria.p__Chlamydiae.c__Chlamydiae.o__Chlamydiales.f__Simkaniaceae.g__Simkania.s__negevensis.t__Z
d__Bacteria.p__Chlamydiae.c__Chlamydiae.o__Chlamydiales.f__Waddliaceae.g__Waddlia.s__chondrophila.t__WSU_86_1044
d__Bacteria.p__Chlamydiae.c__Chlamydiae.o__Chlamydiales.f__Parachlamydiaceae.g__Candidatus_Protochlamydia.s__amoebophila_UWE25.t__amoebophila_UWE25
d__Bacteria.p__Chlamydiae.c__Chlamydiae.o__Chlamydiales.f__Parachlamydiaceae.g__Parachlamydia.s__acanthamoebae.t__UV7
d__Bacteria.p__Chlamydiae.c__Chlamydiae.o__Chlamydiales.f__Parachlamydiaceae.g__Parachlamydia.s__acanthamoebae.t__str__Hall

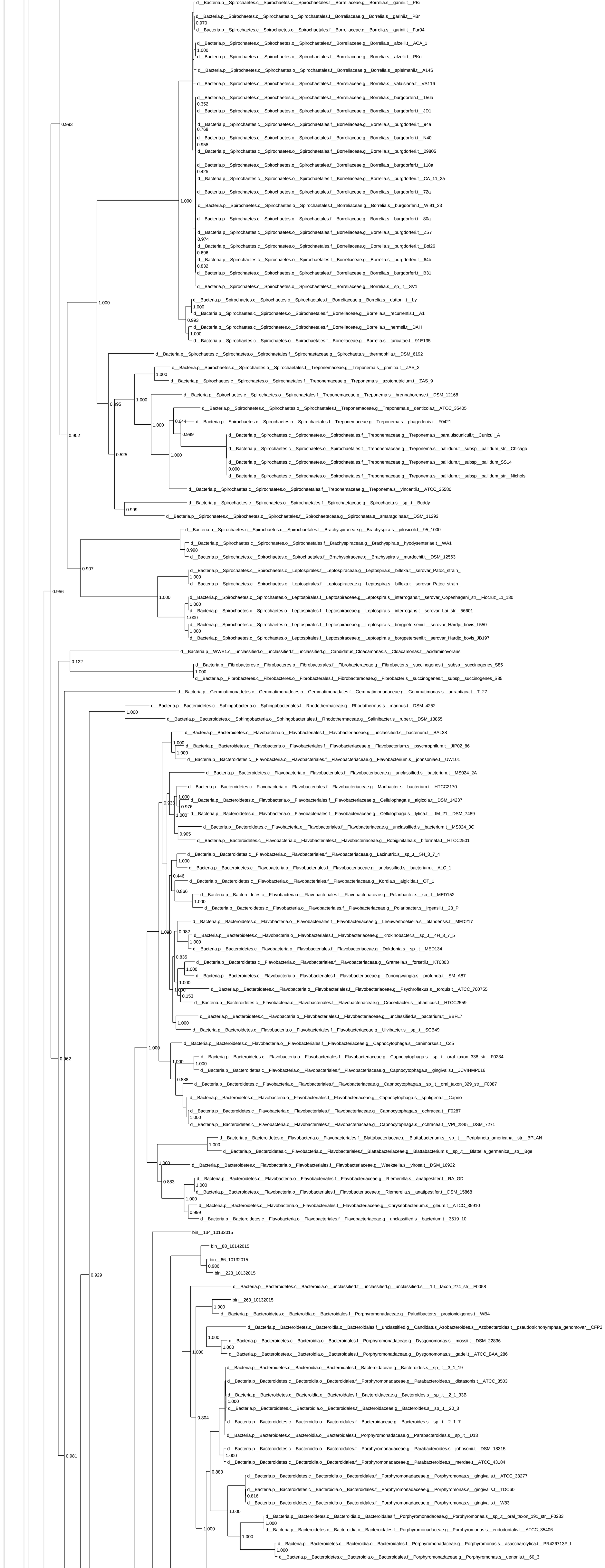
d__Bacteria.p__Spirochaetes.c__Spirochaetes.o__Spirochaetales.f__Borreliaceae.g__Borrelia.s__garinii.t__PBi
d__Bacteria.p__Spirochaetes.c__Spirochaetes.o__Spirochaetales.f__Borreliaceae.g__Borrelia.s__garinii.t__PBr
d__Bacteria.p__Spirochaetes.c__Spirochaetes.o__Spirochaetales.f__Borreliaceae.g__Borrelia.s__garinii.t__Far04
d__Bacteria.p__Spirochaetes.c__Spirochaetes.o__Spirochaetales.f__Borreliaceae.g__Borrelia.s__afzelii.t__ACA_1
d__Bacteria.p__Spirochaetes.c__Spirochaetes.o__Spirochaetales.f__Borreliaceae.g__Borrelia.s__afzelii.t__PKo
d__Bacteria.p__Spirochaetes.c__Spirochaetes.o__Spirochaetales.f__Borreliaceae.g__Borrelia.s__spielmanii.t__A14S
d__Bacteria.p__Spirochaetes.c__Spirochaetes.o__Spirochaetales.f__Borreliaceae.g__Borrelia.s__valaisiana.t__VS116
d__Bacteria.p__Spirochaetes.c__Spirochaetes.o__Spirochaetales.f__Borreliaceae.g__Borrelia.s__burgdorferi.t__156a
d__Bacteria.p__Spirochaetes.c__Spirochaetes.o__Spirochaetales.f__Borreliaceae.g__Borrelia.s__burgdorferi.t__JD1
d__Bacteria.p__Spirochaetes.c__Spirochaetes.o__Spirochaetales.f__Borreliaceae.g__Borrelia.s__burgdorferi.t__94a
d__Bacteria.p__Spirochaetes.c__Spirochaetes.o__Spirochaetales.f__Borreliaceae.g__Borrelia.s__burgdorferi.t__N40
d__Bacteria.p__Spirochaetes.c__Spirochaetes.o__Spirochaetales.f__Borreliaceae.g__Borrelia.s__burgdorferi.t__29805
d__Bacteria.p__Spirochaetes.c__Spirochaetes.o__Spirochaetales.f__Borreliaceae.g__Borrelia.s__burgdorferi.t__118a
d__Bacteria.p__Spirochaetes.c__Spirochaetes.o__Spirochaetales.f__Borreliaceae.g__Borrelia.s__burgdorferi.t__CA_11_2a
d__Bacteria.p__Spirochaetes.c__Spirochaetes.o__Spirochaetales.f__Borreliaceae.g__Borrelia.s__burgdorferi.t__72a
d__Bacteria.p__Spirochaetes.c__Spirochaetes.o__Spirochaetales.f__Borreliaceae.g__Borrelia.s__burgdorferi.t__WI91_23
d__Bacteria.p__Spirochaetes.c__Spirochaetes.o__Spirochaetales.f__Borreliaceae.g__Borrelia.s__burgdorferi.t__80a
d__Bacteria.p__Spirochaetes.c__Spirochaetes.o__Spirochaetales.f__Borreliaceae.g__Borrelia.s__burgdorferi.t__ZS7
d__Bacteria.p__Spirochaetes.c__Spirochaetes.o__Spirochaetales.f__Borreliaceae.g__Borrelia.s__burgdorferi.t__Bol26
d__Bacteria.p__Spirochaetes.c__Spirochaetes.o__Spirochaetales.f__Borreliaceae.g__Borrelia.s__burgdorferi.t__64b
d__Bacteria.p__Spirochaetes.c__Spirochaetes.o__Spirochaetales.f__Borreliaceae.g__Borrelia.s__burgdorferi.t__B31
d__Bacteria.p__Spirochaetes.c__Spirochaetes.o__Spirochaetales.f__Borreliaceae.g__Borrelia.s__sp_.t__SV1
d__Bacteria.p__Spirochaetes.c__Spirochaetes.o__Spirochaetales.f__Borreliaceae.g__Borrelia.s__duttonii.t__Ly
d__Bacteria.p__Spirochaetes.c__Spirochaetes.o__Spirochaetales.f__Borreliaceae.g__Borrelia.s__recurrentis.t__A1
d__Bacteria.p__Spirochaetes.c__Spirochaetes.o__Spirochaetales.f__Borreliaceae.g__Borrelia.s__hermsii.t__DAH
d__Bacteria.p__Spirochaetes.c__Spirochaetes.o__Spirochaetales.f__Borreliaceae.g__Borrelia.s__turicatae.t__91E135
d__Bacteria.p__Spirochaetes.c__Spirochaetes.o__Spirochaetales.f__Spirochaetaceae.g__Spirochaeta.s__thermophila.t__DSM_6192
d__Bacteria.p__Spirochaetes.c__Spirochaetes.o__Spirochaetales.f__Treponemaceae.g__Treponema.s__primitia.t__ZAS_2
d__Bacteria.p__Spirochaetes.c__Spirochaetes.o__Spirochaetales.f__Treponemaceae.g__Treponema.s__azotonutricium.t__ZAS_9
d__Bacteria.p__Spirochaetes.c__Spirochaetes.o__Spirochaetales.f__Treponemaceae.g__Treponema.s__brennaborense.t__DSM_12168
d__Bacteria.p__Spirochaetes.c__Spirochaetes.o__Spirochaetales.f__Treponemaceae.g__Treponema.s__denticola.t__ATCC_35405
d__Bacteria.p__Spirochaetes.c__Spirochaetes.o__Spirochaetales.f__Treponemaceae.g__Treponema.s__phagedenis.t__F0421
d__Bacteria.p__Spirochaetes.c__Spirochaetes.o__Spirochaetales.f__Treponemaceae.g__Treponema.s__paraluiscuniculi.t__Cuniculi_A
d__Bacteria.p__Spirochaetes.c__Spirochaetes.o__Spirochaetales.f__Treponemaceae.g__Treponema.s__pallidum.t__subsp__pallidum_str__Chicago
d__Bacteria.p__Spirochaetes.c__Spirochaetes.o__Spirochaetales.f__Treponemaceae.g__Treponema.s__pallidum.t__subsp__pallidum_SS14
d__Bacteria.p__Spirochaetes.c__Spirochaetes.o__Spirochaetales.f__Treponemaceae.g__Treponema.s__pallidum.t__subsp__pallidum_str__Nichols
d__Bacteria.p__Spirochaetes.c__Spirochaetes.o__Spirochaetales.f__Treponemaceae.g__Treponema.s__vincentii.t__ATCC_35580
d__Bacteria.p__Spirochaetes.c__Spirochaetes.o__Spirochaetales.f__Spirochaetaceae.g__Spirochaeta.s__sp_.t__Buddy
d__Bacteria.p__Spirochaetes.c__Spirochaetes.o__Spirochaetales.f__Spirochaetaceae.g__Spirochaeta.s__smaragdinae.t__DSM_11293
d__Bacteria.p__Spirochaetes.c__Spirochaetes.o__Spirochaetales.f__Brachyspiraceae.g__Brachyspira.s__pilosicoli.t__95_1000
d__Bacteria.p__Spirochaetes.c__Spirochaetes.o__Spirochaetales.f__Brachyspiraceae.g__Brachyspira.s__hyodysenteriae.t__WA1
d__Bacteria.p__Spirochaetes.c__Spirochaetes.o__Spirochaetales.f__Brachyspiraceae.g__Brachyspira.s__murdochii.t__DSM_12563
d__Bacteria.p__Spirochaetes.c__Spirochaetes.o__Leptospirales.f__Leptospiraceae.g__Leptospira.s__biflexa.t__serovar_Patoc_strain_
d__Bacteria.p__Spirochaetes.c__Spirochaetes.o__Leptospirales.f__Leptospiraceae.g__Leptospira.s__biflexa.t__serovar_Patoc_strain_
d__Bacteria.p__Spirochaetes.c__Spirochaetes.o__Leptospirales.f__Leptospiraceae.g__Leptospira.s__interrogans.t__serovar_Copenhageni_str__Fiocruz_L1_130
d__Bacteria.p__Spirochaetes.c__Spirochaetes.o__Leptospirales.f__Leptospiraceae.g__Leptospira.s__interrogans.t__serovar_Lai_str__56601
d__Bacteria.p__Spirochaetes.c__Spirochaetes.o__Leptospirales.f__Leptospiraceae.g__Leptospira.s__borgpetersenii.t__serovar_Hardjo_bovis_L550
d__Bacteria.p__Spirochaetes.c__Spirochaetes.o__Leptospirales.f__Leptospiraceae.g__Leptospira.s__borgpetersenii.t__serovar_Hardjo_bovis_JB197
d__Bacteria.p__WWE1.c__unclassified.o__unclassified.f__unclassified.g__Candidatus_Cloacamonas.s__Cloacamonas.t__acidaminovorans
d__Bacteria.p__Fibrobacteres.c__Fibrobacteres.o__Fibrobacterales.f__Fibrobacteraceae.g__Fibrobacter.s__succinogenes.t__subsp__succinogenes_S85
d__Bacteria.p__Fibrobacteres.c__Fibrobacteres.o__Fibrobacterales.f__Fibrobacteraceae.g__Fibrobacter.s__succinogenes.t__subsp__succinogenes_S85
d__Bacteria.p__Gemmatimonadetes.c__Gemmatimonadetes.o__Gemmatimonadales.f__Gemmatimonadaceae.g__Gemmatimonas.s__aurantiaca.t__T_27
d__Bacteria.p__Bacteroidetes.c__Sphingobacteria.o__Sphingobacteriales.f__Rhodothermaceae.g__Rhodothermus.s__marinus.t__DSM_4252
d__Bacteria.p__Bacteroidetes.c__Sphingobacteria.o__Sphingobacteriales.f__Rhodothermaceae.g__Salinibacter.s__ruber.t__DSM_13855
d__Bacteria.p__Bacteroidetes.c__Flavobacteria.o__Flavobacteriales.f__Flavobacteriaceae.g__unclassified.s__bacterium.t__BAL38
d__Bacteria.p__Bacteroidetes.c__Flavobacteria.o__Flavobacteriales.f__Flavobacteriaceae.g__Flavobacterium.s__psychrophilum.t__JIP02_86
d__Bacteria.p__Bacteroidetes.c__Flavobacteria.o__Flavobacteriales.f__Flavobacteriaceae.g__Flavobacterium.s__johnsoniae.t__UW101
d__Bacteria.p__Bacteroidetes.c__Flavobacteria.o__Flavobacteriales.f__Flavobacteriaceae.g__unclassified.s__bacterium.t__MS024_2A
d__Bacteria.p__Bacteroidetes.c__Flavobacteria.o__Flavobacteriales.f__Flavobacteriaceae.g__Maribacter.s__bacterium.t__HTCC2170
d__Bacteria.p__Bacteroidetes.c__Flavobacteria.o__Flavobacteriales.f__Flavobacteriaceae.g__Cellulophaga.s__algicola.t__DSM_14237
d__Bacteria.p__Bacteroidetes.c__Flavobacteria.o__Flavobacteriales.f__Flavobacteriaceae.g__Cellulophaga.s__lytica.t__LIM_21__DSM_7489
d__Bacteria.p__Bacteroidetes.c__Flavobacteria.o__Flavobacteriales.f__Flavobacteriaceae.g__unclassified.s__bacterium.t__MS024_3C
d__Bacteria.p__Bacteroidetes.c__Flavobacteria.o__Flavobacteriales.f__Flavobacteriaceae.g__Robiginitalea.s__biformata.t__HTCC2501
d__Bacteria.p__Bacteroidetes.c__Flavobacteria.o__Flavobacteriales.f__Flavobacteriaceae.g__Lacinutrix.s__sp_.t__SH_3_7_4
d__Bacteria.p__Bacteroidetes.c__Flavobacteria.o__Flavobacteriales.f__Flavobacteriaceae.g__unclassified.s__bacterium.t__ALC_1
d__Bacteria.p__Bacteroidetes.c__Flavobacteria.o__Flavobacteriales.f__Flavobacteriaceae.g__Kordia.s__algicida.t__OT_1
d__Bacteria.p__Bacteroidetes.c__Flavobacteria.o__Flavobacteriales.f__Flavobacteriaceae.g__Polaribacter.s__sp_.t__MED152
d__Bacteria.p__Bacteroidetes.c__Flavobacteria.o__Flavobacteriales.f__Flavobacteriaceae.g__Polaribacter.s__irgensii.t__23_P
d__Bacteria.p__Bacteroidetes.c__Flavobacteria.o__Flavobacteriales.f__Flavobacteriaceae.g__Leeuwenhoekiella.s__blandensis.t__MED217
d__Bacteria.p__Bacteroidetes.c__Flavobacteria.o__Flavobacteriales.f__Flavobacteriaceae.g__Krokinobacter.s__sp_.t__4H_3_7_5
d__Bacteria.p__Bacteroidetes.c__Flavobacteria.o__Flavobacteriales.f__Flavobacteriaceae.g__Dokdonia.s__sp_.t__MED134
d__Bacteria.p__Bacteroidetes.c__Flavobacteria.o__Flavobacteriales.f__Flavobacteriaceae.g__Gramella.s__forsetii.t__KT0803
d__Bacteria.p__Bacteroidetes.c__Flavobacteria.o__Flavobacteriales.f__Flavobacteriaceae.g__Zunongwangia.s__profunda.t__SM_A87
d__Bacteria.p__Bacteroidetes.c__Flavobacteria.o__Flavobacteriales.f__Flavobacteriaceae.g__Psychroflexus.s__torquis.t__ATCC_700755
d__Bacteria.p__Bacteroidetes.c__Flavobacteria.o__Flavobacteriales.f__Flavobacteriaceae.g__Croceibacter.s__atlanticus.t__HTCC2559
d__Bacteria.p__Bacteroidetes.c__Flavobacteria.o__Flavobacteriales.f__Flavobacteriaceae.g__unclassified.s__bacterium.t__BBFL7
d__Bacteria.p__Bacteroidetes.c__Flavobacteria.o__Flavobacteriales.f__Flavobacteriaceae.g__Ulvibacter.s__sp_.t__SCB49
d__Bacteria.p__Bacteroidetes.c__Flavobacteria.o__Flavobacteriales.f__Flavobacteriaceae.g__Capnocytophaga.s__canimorsus.t__Cc5
d__Bacteria.p__Bacteroidetes.c__Flavobacteria.o__Flavobacteriales.f__Flavobacteriaceae.g__Capnocytophaga.s__sp_.t__oral_taxon_338_str__F0234
d__Bacteria.p__Bacteroidetes.c__Flavobacteria.o__Flavobacteriales.f__Flavobacteriaceae.g__Capnocytophaga.s__gingivalis.t__JCVIHMP016
d__Bacteria.p__Bacteroidetes.c__Flavobacteria.o__Flavobacteriales.f__Flavobacteriaceae.g__Capnocytophaga.s__sp_.t__oral_taxon_329_str__F0087
d__Bacteria.p__Bacteroidetes.c__Flavobacteria.o__Flavobacteriales.f__Flavobacteriaceae.g__Capnocytophaga.s__sputigena.t__Capno
d__Bacteria.p__Bacteroidetes.c__Flavobacteria.o__Flavobacteriales.f__Flavobacteriaceae.g__Capnocytophaga.s__ochracea.t__F0287
d__Bacteria.p__Bacteroidetes.c__Flavobacteria.o__Flavobacteriales.f__Flavobacteriaceae.g__Capnocytophaga.s__ochracea.t__VPI_2845__DSM_7271
d__Bacteria.p__Bacteroidetes.c__Flavobacteria.o__Flavobacteriales.f__Blattabacteriaceae.g__Blattabacterium.s__sp_.t__Periplaneta_americana__str__BPLAN
d__Bacteria.p__Bacteroidetes.c__Flavobacteria.o__Flavobacteriales.f__Blattabacteriaceae.g__Blattabacterium.s__sp_.t__Blattella_germanica__str__Bge
d__Bacteria.p__Bacteroidetes.c__Flavobacteria.o__Flavobacteriales.f__Flavobacteriaceae.g__Weeksella.s__virosa.t__DSM_16922
d__Bacteria.p__Bacteroidetes.c__Flavobacteria.o__Flavobacteriales.f__Flavobacteriaceae.g__Riemerella.s__anatipestifer.t__RA_GD
d__Bacteria.p__Bacteroidetes.c__Flavobacteria.o__Flavobacteriales.f__Flavobacteriaceae.g__Riemerella.s__anatipestifer.t__DSM_15868
d__Bacteria.p__Bacteroidetes.c__Flavobacteria.o__Flavobacteriales.f__Flavobacteriaceae.g__Chryseobacterium.s__gleum.t__ATCC_35910
d__Bacteria.p__Bacteroidetes.c__Flavobacteria.o__Flavobacteriales.f__Flavobacteriaceae.g__unclassified.s__bacterium.t__3519_10
bin__134_10132015
bin__88_10142015
bin__66_10132015
bin__223_10132015
d__Bacteria.p__Bacteroidetes.c__Bacteroidia.o__unclassified.f__unclassified.g__unclassified.s___1.t__taxon_274_str__F0058
bin__263_10132015
d__Bacteria.p__Bacteroidetes.c__Bacteroidia.o__Bacteroidales.f__Porphyromonadaceae.g__Paludibacter.s__propionicigenes.t__WB4
d__Bacteria.p__Bacteroidetes.c__Bacteroidia.o__Bacteroidales.f__unclassified.g__Candidatus_Azobacteroides.s__Azobacteroides.t__pseudotrichonymphae_genomovar__CFP2
d__Bacteria.p__Bacteroidetes.c__Bacteroidia.o__Bacteroidales.f__Porphyromonadaceae.g__Dysgonomonas.s__mossii.t__DSM_22836
d__Bacteria.p__Bacteroidetes.c__Bacteroidia.o__Bacteroidales.f__Porphyromonadaceae.g__Dysgonomonas.s__gadei.t__ATCC_BAA_286
d__Bacteria.p__Bacteroidetes.c__Bacteroidia.o__Bacteroidales.f__Bacteroidaceae.g__Bacteroides.s__sp_.t__3_1_19
d__Bacteria.p__Bacteroidetes.c__Bacteroidia.o__Bacteroidales.f__Porphyromonadaceae.g__Parabacteroides.s__distasonis.t__ATCC_8503
d__Bacteria.p__Bacteroidetes.c__Bacteroidia.o__Bacteroidales.f__Bacteroidaceae.g__Bacteroides.s__sp_.t__2_1_33B
d__Bacteria.p__Bacteroidetes.c__Bacteroidia.o__Bacteroidales.f__Bacteroidaceae.g__Bacteroides.s__sp_.t__20_3
d__Bacteria.p__Bacteroidetes.c__Bacteroidia.o__Bacteroidales.f__Bacteroidaceae.g__Bacteroides.s__sp_.t__2_1_7
d__Bacteria.p__Bacteroidetes.c__Bacteroidia.o__Bacteroidales.f__Porphyromonadaceae.g__Parabacteroides.s__sp_.t__D13
d__Bacteria.p__Bacteroidetes.c__Bacteroidia.o__Bacteroidales.f__Porphyromonadaceae.g__Parabacteroides.s__johnsonii.t__DSM_18315
d__Bacteria.p__Bacteroidetes.c__Bacteroidia.o__Bacteroidales.f__Porphyromonadaceae.g__Parabacteroides.s__merdae.t__ATCC_43184
d__Bacteria.p__Bacteroidetes.c__Bacteroidia.o__Bacteroidales.f__Porphyromonadaceae.g__Porphyromonas.s__gingivalis.t__ATCC_33277
d__Bacteria.p__Bacteroidetes.c__Bacteroidia.o__Bacteroidales.f__Porphyromonadaceae.g__Porphyromonas.s__gingivalis.t__TDC60
d__Bacteria.p__Bacteroidetes.c__Bacteroidia.o__Bacteroidales.f__Porphyromonadaceae.g__Porphyromonas.s__gingivalis.t__W83
d__Bacteria.p__Bacteroidetes.c__Bacteroidia.o__Bacteroidales.f__Porphyromonadaceae.g__Porphyromonas.s__sp_.t__oral_taxon_191_str__F0233
d__Bacteria.p__Bacteroidetes.c__Bacteroidia.o__Bacteroidales.f__Porphyromonadaceae.g__Porphyromonas.s__endodontalis.t__ATCC_35406
d__Bacteria.p__Bacteroidetes.c__Bacteroidia.o__Bacteroidales.f__Porphyromonadaceae.g__Porphyromonas.s__asaccharolytica.t__PR426713P_I
d__Bacteria.p__Bacteroidetes.c__Bacteroidia.o__Bacteroidales.f__Porphyromonadaceae.g__Porphyromonas.s__uenonis.t__60_3

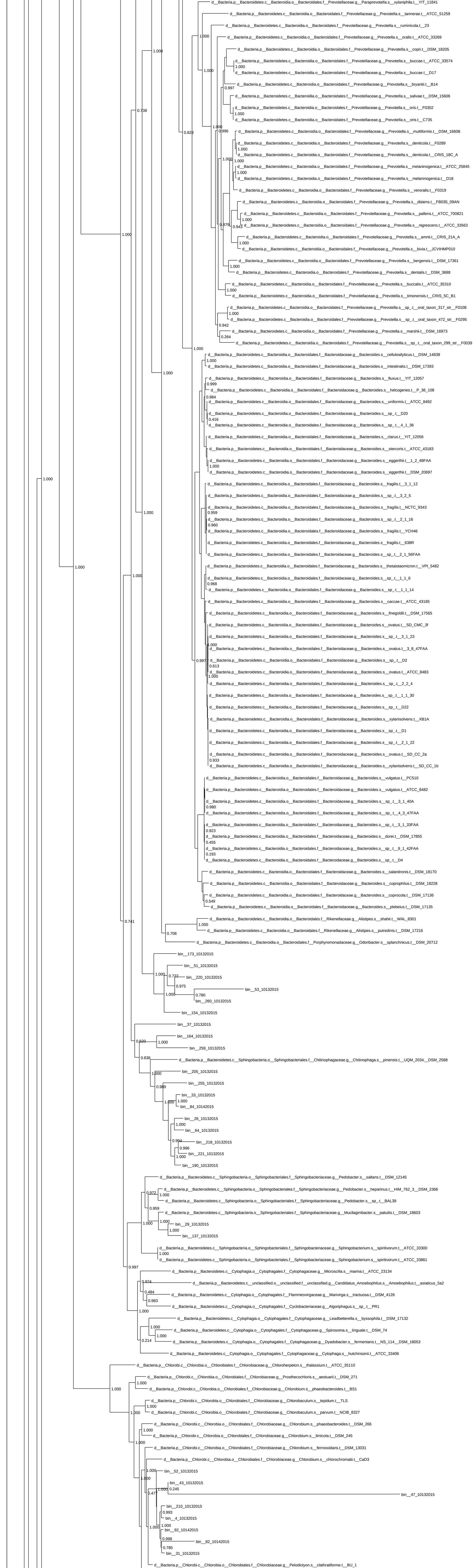

d__Bacteria.p__Bacteroidetes.c__Bacteroidia.o__Bacteroidales.f__Prevotellaceae.g__Paraprevotella.s__xylaniphila.t__YIT_11841
d__Bacteria.p__Bacteroidetes.c__Bacteroidia.o__Bacteroidales.f__Prevotellaceae.g__Prevotella.s__tannerae.t__ATCC_51259
d__Bacteria.p__Bacteroidetes.c__Bacteroidia.o__Bacteroidales.f__Prevotellaceae.g__Prevotella.s__ruminicola.t__23
d__Bacteria.p__Bacteroidetes.c__Bacteroidia.o__Bacteroidales.f__Prevotellaceae.g__Prevotella.s__oralis.t__ATCC_33269
d__Bacteria.p__Bacteroidetes.c__Bacteroidia.o__Bacteroidales.f__Prevotellaceae.g__Prevotella.s__copri.t__DSM_18205
d__Bacteria.p__Bacteroidetes.c__Bacteroidia.o__Bacteroidales.f__Prevotellaceae.g__Prevotella.s__buccae.t__ATCC_33574
d__Bacteria.p__Bacteroidetes.c__Bacteroidia.o__Bacteroidales.f__Prevotellaceae.g__Prevotella.s__buccae.t__D17
d__Bacteria.p__Bacteroidetes.c__Bacteroidia.o__Bacteroidales.f__Prevotellaceae.g__Prevotella.s__bryantii.t__B14
d__Bacteria.p__Bacteroidetes.c__Bacteroidia.o__Bacteroidales.f__Prevotellaceae.g__Prevotella.s__salivae.t__DSM_15606
d__Bacteria.p__Bacteroidetes.c__Bacteroidia.o__Bacteroidales.f__Prevotellaceae.g__Prevotella.s__oris.t__F0302
d__Bacteria.p__Bacteroidetes.c__Bacteroidia.o__Bacteroidales.f__Prevotellaceae.g__Prevotella.s__oris.t__C735
d__Bacteria.p__Bacteroidetes.c__Bacteroidia.o__Bacteroidales.f__Prevotellaceae.g__Prevotella.s__multiformis.t__DSM_16608
d__Bacteria.p__Bacteroidetes.c__Bacteroidia.o__Bacteroidales.f__Prevotellaceae.g__Prevotella.s__denticola.t__F0289
d__Bacteria.p__Bacteroidetes.c__Bacteroidia.o__Bacteroidales.f__Prevotellaceae.g__Prevotella.s__denticola.t__CRIS_18C_A
d__Bacteria.p__Bacteroidetes.c__Bacteroidia.o__Bacteroidales.f__Prevotellaceae.g__Prevotella.s__melaninogenica.t__ATCC_25845
d__Bacteria.p__Bacteroidetes.c__Bacteroidia.o__Bacteroidales.f__Prevotellaceae.g__Prevotella.s__melaninogenica.t__D18
d__Bacteria.p__Bacteroidetes.c__Bacteroidia.o__Bacteroidales.f__Prevotellaceae.g__Prevotella.s__veroralis.t__F0319
d__Bacteria.p__Bacteroidetes.c__Bacteroidia.o__Bacteroidales.f__Prevotellaceae.g__Prevotella.s__disiens.t__FB035_09AN
d__Bacteria.p__Bacteroidetes.c__Bacteroidia.o__Bacteroidales.f__Prevotellaceae.g__Prevotella.s__pallens.t__ATCC_700821
d__Bacteria.p__Bacteroidetes.c__Bacteroidia.o__Bacteroidales.f__Prevotellaceae.g__Prevotella.s__nigrescens.t__ATCC_33563
d__Bacteria.p__Bacteroidetes.c__Bacteroidia.o__Bacteroidales.f__Prevotellaceae.g__Prevotella.s__amnii.t__CRIS_21A_A
d__Bacteria.p__Bacteroidetes.c__Bacteroidia.o__Bacteroidales.f__Prevotellaceae.g__Prevotella.s__bivia.t__JCVIHMP010
d__Bacteria.p__Bacteroidetes.c__Bacteroidia.o__Bacteroidales.f__Prevotellaceae.g__Prevotella.s__bergensis.t__DSM_17361
d__Bacteria.p__Bacteroidetes.c__Bacteroidia.o__Bacteroidales.f__Prevotellaceae.g__Prevotella.s__dentalis.t__DSM_3688
d__Bacteria.p__Bacteroidetes.c__Bacteroidia.o__Bacteroidales.f__Prevotellaceae.g__Prevotella.s__buccalis.t__ATCC_35310
d__Bacteria.p__Bacteroidetes.c__Bacteroidia.o__Bacteroidales.f__Prevotellaceae.g__Prevotella.s__timonensis.t__CRIS_5C_B1
d__Bacteria.p__Bacteroidetes.c__Bacteroidia.o__Bacteroidales.f__Prevotellaceae.g__Prevotella.s__sp_.t__oral_taxon_317_str__F0108
d__Bacteria.p__Bacteroidetes.c__Bacteroidia.o__Bacteroidales.f__Prevotellaceae.g__Prevotella.s__sp_.t__oral_taxon_472_str__F0295
d__Bacteria.p__Bacteroidetes.c__Bacteroidia.o__Bacteroidales.f__Prevotellaceae.g__Prevotella.s__marshii.t__DSM_16973
d__Bacteria.p__Bacteroidetes.c__Bacteroidia.o__Bacteroidales.f__Prevotellaceae.g__Prevotella.s__sp_.t__oral_taxon_299_str__F0039
d__Bacteria.p__Bacteroidetes.c__Bacteroidia.o__Bacteroidales.f__Bacteroidaceae.g__Bacteroides.s__cellulosilyticus.t__DSM_14838
d__Bacteria.p__Bacteroidetes.c__Bacteroidia.o__Bacteroidales.f__Bacteroidaceae.g__Bacteroides.s__intestinalis.t__DSM_17393
d__Bacteria.p__Bacteroidetes.c__Bacteroidia.o__Bacteroidales.f__Bacteroidaceae.g__Bacteroides.s__fluxus.t__YIT_12057
d__Bacteria.p__Bacteroidetes.c__Bacteroidia.o__Bacteroidales.f__Bacteroidaceae.g__Bacteroides.s__helcogenes.t__P_36_108
d__Bacteria.p__Bacteroidetes.c__Bacteroidia.o__Bacteroidales.f__Bacteroidaceae.g__Bacteroides.s__uniformis.t__ATCC_8492
d__Bacteria.p__Bacteroidetes.c__Bacteroidia.o__Bacteroidales.f__Bacteroidaceae.g__Bacteroides.s__sp_.t__D20
d__Bacteria.p__Bacteroidetes.c__Bacteroidia.o__Bacteroidales.f__Bacteroidaceae.g__Bacteroides.s__sp_.t__4_1_36
d__Bacteria.p__Bacteroidetes.c__Bacteroidia.o__Bacteroidales.f__Bacteroidaceae.g__Bacteroides.s__clarus.t__YIT_12056
d__Bacteria.p__Bacteroidetes.c__Bacteroidia.o__Bacteroidales.f__Bacteroidaceae.g__Bacteroides.s__stercoris.t__ATCC_43183
d__Bacteria.p__Bacteroidetes.c__Bacteroidia.o__Bacteroidales.f__Bacteroidaceae.g__Bacteroides.s__eggerthii.t__1_2_48FAA
d__Bacteria.p__Bacteroidetes.c__Bacteroidia.o__Bacteroidales.f__Bacteroidaceae.g__Bacteroides.s__eggerthii.t__DSM_20697
d__Bacteria.p__Bacteroidetes.c__Bacteroidia.o__Bacteroidales.f__Bacteroidaceae.g__Bacteroides.s__fragilis.t__3_1_12
d__Bacteria.p__Bacteroidetes.c__Bacteroidia.o__Bacteroidales.f__Bacteroidaceae.g__Bacteroides.s__sp_.t__3_2_5
d__Bacteria.p__Bacteroidetes.c__Bacteroidia.o__Bacteroidales.f__Bacteroidaceae.g__Bacteroides.s__fragilis.t__NCTC_9343
d__Bacteria.p__Bacteroidetes.c__Bacteroidia.o__Bacteroidales.f__Bacteroidaceae.g__Bacteroides.s__sp_.t__2_1_16
d__Bacteria.p__Bacteroidetes.c__Bacteroidia.o__Bacteroidales.f__Bacteroidaceae.g__Bacteroides.s__fragilis.t__YCH46
d__Bacteria.p__Bacteroidetes.c__Bacteroidia.o__Bacteroidales.f__Bacteroidaceae.g__Bacteroides.s__fragilis.t__638R
d__Bacteria.p__Bacteroidetes.c__Bacteroidia.o__Bacteroidales.f__Bacteroidaceae.g__Bacteroides.s__sp_.t__2_1_56FAA
d__Bacteria.p__Bacteroidetes.c__Bacteroidia.o__Bacteroidales.f__Bacteroidaceae.g__Bacteroides.s__thetaiotaomicron.t__VPI_5482
d__Bacteria.p__Bacteroidetes.c__Bacteroidia.o__Bacteroidales.f__Bacteroidaceae.g__Bacteroides.s__sp_.t__1_1_6
d__Bacteria.p__Bacteroidetes.c__Bacteroidia.o__Bacteroidales.f__Bacteroidaceae.g__Bacteroides.s__sp_.t__1_1_14
d__Bacteria.p__Bacteroidetes.c__Bacteroidia.o__Bacteroidales.f__Bacteroidaceae.g__Bacteroides.s__caccae.t__ATCC_43185
d__Bacteria.p__Bacteroidetes.c__Bacteroidia.o__Bacteroidales.f__Bacteroidaceae.g__Bacteroides.s__finegoldii.t__DSM_17565
d__Bacteria.p__Bacteroidetes.c__Bacteroidia.o__Bacteroidales.f__Bacteroidaceae.g__Bacteroides.s__ovatus.t__SD_CMC_3f
d__Bacteria.p__Bacteroidetes.c__Bacteroidia.o__Bacteroidales.f__Bacteroidaceae.g__Bacteroides.s__sp_.t__3_1_23
d__Bacteria.p__Bacteroidetes.c__Bacteroidia.o__Bacteroidales.f__Bacteroidaceae.g__Bacteroides.s__ovatus.t__3_8_47FAA
d__Bacteria.p__Bacteroidetes.c__Bacteroidia.o__Bacteroidales.f__Bacteroidaceae.g__Bacteroides.s__sp_.t__D2
d__Bacteria.p__Bacteroidetes.c__Bacteroidia.o__Bacteroidales.f__Bacteroidaceae.g__Bacteroides.s__ovatus.t__ATCC_8483
d__Bacteria.p__Bacteroidetes.c__Bacteroidia.o__Bacteroidales.f__Bacteroidaceae.g__Bacteroides.s__sp_.t__2_2_4
d__Bacteria.p__Bacteroidetes.c__Bacteroidia.o__Bacteroidales.f__Bacteroidaceae.g__Bacteroides.s__sp_.t__1_1_30
d__Bacteria.p__Bacteroidetes.c__Bacteroidia.o__Bacteroidales.f__Bacteroidaceae.g__Bacteroides.s__sp_.t__D22
d__Bacteria.p__Bacteroidetes.c__Bacteroidia.o__Bacteroidales.f__Bacteroidaceae.g__Bacteroides.s__xylanisolvens.t__XB1A
d__Bacteria.p__Bacteroidetes.c__Bacteroidia.o__Bacteroidales.f__Bacteroidaceae.g__Bacteroides.s__sp_.t__D1
d__Bacteria.p__Bacteroidetes.c__Bacteroidia.o__Bacteroidales.f__Bacteroidaceae.g__Bacteroides.s__sp_.t__2_1_22
d__Bacteria.p__Bacteroidetes.c__Bacteroidia.o__Bacteroidales.f__Bacteroidaceae.g__Bacteroides.s__ovatus.t__SD_CC_2a
d__Bacteria.p__Bacteroidetes.c__Bacteroidia.o__Bacteroidales.f__Bacteroidaceae.g__Bacteroides.s__xylanisolvens.t__SD_CC_1b
d__Bacteria.p__Bacteroidetes.c__Bacteroidia.o__Bacteroidales.f__Bacteroidaceae.g__Bacteroides.s__vulgatus.t__PC510
d__Bacteria.p__Bacteroidetes.c__Bacteroidia.o__Bacteroidales.f__Bacteroidaceae.g__Bacteroides.s__vulgatus.t__ATCC_8482
d__Bacteria.p__Bacteroidetes.c__Bacteroidia.o__Bacteroidales.f__Bacteroidaceae.g__Bacteroides.s__sp_.t__3_1_40A
d__Bacteria.p__Bacteroidetes.c__Bacteroidia.o__Bacteroidales.f__Bacteroidaceae.g__Bacteroides.s__sp_.t__4_3_47FAA
d__Bacteria.p__Bacteroidetes.c__Bacteroidia.o__Bacteroidales.f__Bacteroidaceae.g__Bacteroides.s__sp_.t__3_1_33FAA
d__Bacteria.p__Bacteroidetes.c__Bacteroidia.o__Bacteroidales.f__Bacteroidaceae.g__Bacteroides.s__dorei.t__DSM_17855
d__Bacteria.p__Bacteroidetes.c__Bacteroidia.o__Bacteroidales.f__Bacteroidaceae.g__Bacteroides.s__sp_.t__9_1_42FAA
d__Bacteria.p__Bacteroidetes.c__Bacteroidia.o__Bacteroidales.f__Bacteroidaceae.g__Bacteroides.s__sp_.t__D4
d__Bacteria.p__Bacteroidetes.c__Bacteroidia.o__Bacteroidales.f__Bacteroidaceae.g__Bacteroides.s__salanitronis.t__DSM_18170
d__Bacteria.p__Bacteroidetes.c__Bacteroidia.o__Bacteroidales.f__Bacteroidaceae.g__Bacteroides.s__coprophilus.t__DSM_18228
d__Bacteria.p__Bacteroidetes.c__Bacteroidia.o__Bacteroidales.f__Bacteroidaceae.g__Bacteroides.s__coprocola.t__DSM_17136
d__Bacteria.p__Bacteroidetes.c__Bacteroidia.o__Bacteroidales.f__Bacteroidaceae.g__Bacteroides.s__plebeius.t__DSM_17135
d__Bacteria.p__Bacteroidetes.c__Bacteroidia.o__Bacteroidales.f__Rikenellaceae.g__Alistipes.s__shahii.t__WAL_8301
d__Bacteria.p__Bacteroidetes.c__Bacteroidia.o__Bacteroidales.f__Rikenellaceae.g__Alistipes.s__putredinis.t__DSM_17216
d__Bacteria.p__Bacteroidetes.c__Bacteroidia.o__Bacteroidales.f__Porphyromonadaceae.g__Odoribacter.s__splanchnicus.t__DSM_20712
bin__173_10132015
bin__51_10132015
bin__220_10132015
bin__53_10132015
bin__260_10132015
bin__154_10132015
bin__37_10132015
bin__164_10132015
bin__259_10132015
d__Bacteria.p__Bacteroidetes.c__Sphingobacteria.o__Sphingobacteriales.f__Chitinophagaceae.g__Chitinophaga.s__pinensis.t__UQM_2034__DSM_2588
bin__205_10132015
bin__255_10132015
bin__33_10132015
bin__84_10142015
bin__26_10132015
bin__64_10132015
bin__218_10132015
bin__221_10132015
bin__190_10132015
d__Bacteria.p__Bacteroidetes.c__Sphingobacteria.o__Sphingobacteriales.f__Sphingobacteriaceae.g__Pedobacter.s__saltans.t__DSM_12145
d__Bacteria.p__Bacteroidetes.c__Sphingobacteria.o__Sphingobacteriales.f__Sphingobacteriaceae.g__Pedobacter.s__heparinus.t__HIM_762_3__DSM_2366
d__Bacteria.p__Bacteroidetes.c__Sphingobacteria.o__Sphingobacteriales.f__Sphingobacteriaceae.g__Pedobacter.s__sp_.t__BAL39
d__Bacteria.p__Bacteroidetes.c__Sphingobacteria.o__Sphingobacteriales.f__Sphingobacteriaceae.g__Mucilaginibacter.s__paludis.t__DSM_18603
bin__29_10132015
bin__137_10132015
d__Bacteria.p__Bacteroidetes.c__Sphingobacteria.o__Sphingobacteriales.f__Sphingobacteriaceae.g__Sphingobacterium.s__spiritivorum.t__ATCC_33300
d__Bacteria.p__Bacteroidetes.c__Sphingobacteria.o__Sphingobacteriales.f__Sphingobacteriaceae.g__Sphingobacterium.s__spiritivorum.t__ATCC_33861
d__Bacteria.p__Bacteroidetes.c__Cytophagia.o__Cytophagales.f__Cytophagaceae.g__Microscilla.s__marina.t__ATCC_23134
d__Bacteria.p__Bacteroidetes.c__unclassified.o__unclassified.f__unclassified.g__Candidatus_Amoebophilus.s__Amoebophilus.t__asiaticus_5a2
d__Bacteria.p__Bacteroidetes.c__Cytophagia.o__Cytophagales.f__Flammeovirgaceae.g__Marivirga.s__tractuosa.t__DSM_4126
d__Bacteria.p__Bacteroidetes.c__Cytophagia.o__Cytophagales.f__Cyclobacteriaceae.g__Algoriphagus.s__sp_.t__PR1
d__Bacteria.p__Bacteroidetes.c__Cytophagia.o__Cytophagales.f__Cytophagaceae.g__Leadbetterella.s__byssophila.t__DSM_17132
d__Bacteria.p__Bacteroidetes.c__Cytophagia.o__Cytophagales.f__Cytophagaceae.g__Spirosoma.s__linguale.t__DSM_74
d__Bacteria.p__Bacteroidetes.c__Cytophagia.o__Cytophagales.f__Cytophagaceae.g__Dyadobacter.s__fermentans.t__NS_114__DSM_18053
d__Bacteria.p__Bacteroidetes.c__Cytophagia.o__Cytophagales.f__Cytophagaceae.g__Cytophaga.s__hutchinsonii.t__ATCC_33406
d__Bacteria.p__Chlorobi.c__Chlorobia.o__Chlorobiales.f__Chlorobiaceae.g__Chloroherpeton.s__thalassium.t__ATCC_35110
d__Bacteria.p__Chlorobi.c__Chlorobia.o__Chlorobiales.f__Chlorobiaceae.g__Prosthecochloris.s__aestuarii.t__DSM_271
d__Bacteria.p__Chlorobi.c__Chlorobia.o__Chlorobiales.f__Chlorobiaceae.g__Chlorobium.s__phaeobacteroides.t__BS1
d__Bacteria.p__Chlorobi.c__Chlorobia.o__Chlorobiales.f__Chlorobiaceae.g__Chlorobaculum.s__tepidum.t__TLS
d__Bacteria.p__Chlorobi.c__Chlorobia.o__Chlorobiales.f__Chlorobiaceae.g__Chlorobaculum.s__parvum.t__NCIB_8327
d__Bacteria.p__Chlorobi.c__Chlorobia.o__Chlorobiales.f__Chlorobiaceae.g__Chlorobium.s__phaeobacteroides.t__DSM_266
d__Bacteria.p__Chlorobi.c__Chlorobia.o__Chlorobiales.f__Chlorobiaceae.g__Chlorobium.s__limicola.t__DSM_245
d__Bacteria.p__Chlorobi.c__Chlorobia.o__Chlorobiales.f__Chlorobiaceae.g__Chlorobium.s__ferrooxidans.t__DSM_13031
d__Bacteria.p__Chlorobi.c__Chlorobia.o__Chlorobiales.f__Chlorobiaceae.g__Chlorobium.s__chlorochromatii.t__CaD3
bin__52_10132015
bin__43_10132015
bin__47_10132015
bin__210_10132015
bin__4_10132015
bin__92_10142015
bin__82_10142015
bin__31_10132015
d__Bacteria.p__Chlorobi.c__Chlorobia.o__Chlorobiales.f__Chlorobiaceae.g__Pelodictyon.s__clathratiforme.t__BU_1

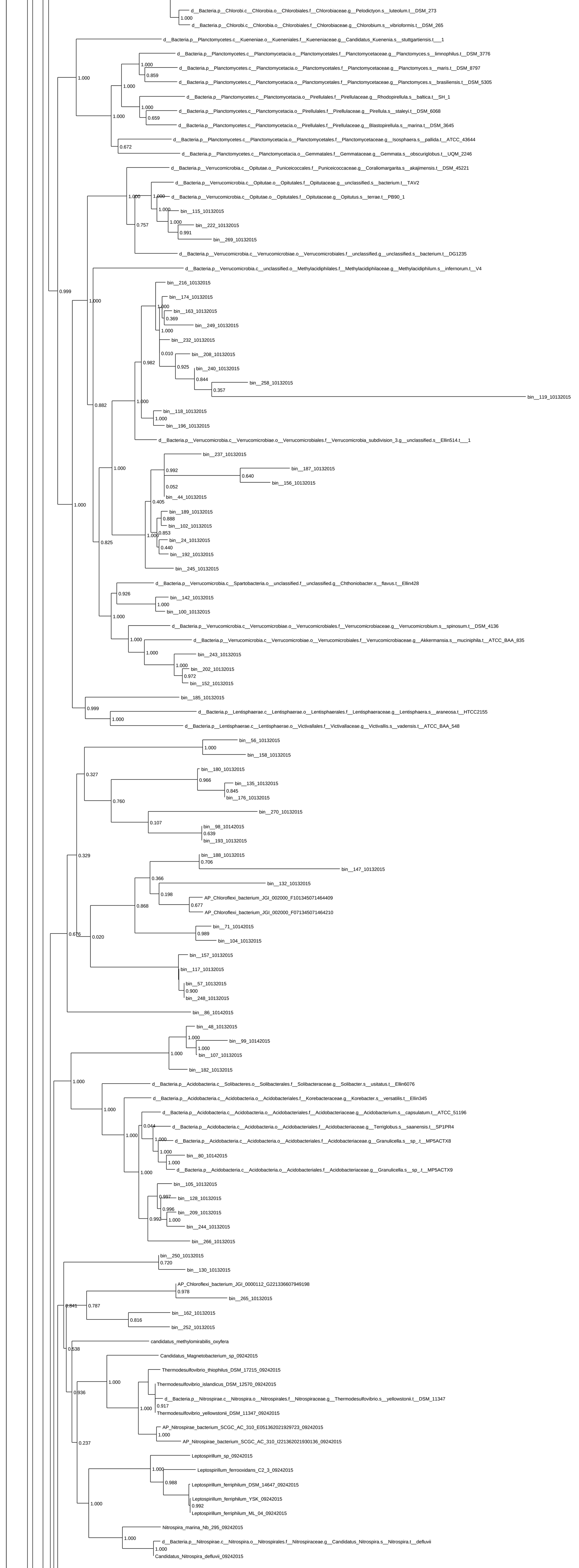
d__Bacteria.p__Chlorobi.c__Chlorobia.o__Chlorobiales.f__Chlorobiaceae.g__Pelodictyon.s__luteolum.t__DSM_273
d__Bacteria.p__Chlorobi.c__Chlorobia.o__Chlorobiales.f__Chlorobiaceae.g__Chlorobium.s__vibrioformis.t__DSM_265
d__Bacteria.p__Planctomycetes.c__Kueneniae.o__Kueneniales.f__Kueneniaceae.g__Candidatus_Kuenenia.s__stuttgartiensis.t__1
d__Bacteria.p__Planctomycetes.c__Planctomycetacia.o__Planctomycetales.f__Planctomycetaceae.g__Planctomyces.s__limnophilus.t__DSM_3776
d__Bacteria.p__Planctomycetes.c__Planctomycetacia.o__Planctomycetales.f__Planctomycetaceae.g__Planctomyces.s__maris.t__DSM_8797
d__Bacteria.p__Planctomycetes.c__Planctomycetacia.o__Planctomycetales.f__Planctomycetaceae.g__Planctomyces.s__brasiliensis.t__DSM_5305
d__Bacteria.p__Planctomycetes.c__Planctomycetacia.o__Pirellulales.f__Pirellulaceae.g__Rhodopirellula.s__baltica.t__SH_1
d__Bacteria.p__Planctomycetes.c__Planctomycetacia.o__Pirellulales.f__Pirellulaceae.g__Pirellula.s__staleyi.t__DSM_6068
d__Bacteria.p__Planctomycetes.c__Planctomycetacia.o__Pirellulales.f__Pirellulaceae.g__Blastopirellula.s__marina.t__DSM_3645
d__Bacteria.p__Planctomycetes.c__Planctomycetacia.o__Planctomycetales.f__Planctomycetaceae.g__Isosphaera.s__pallida.t__ATCC_43644
d__Bacteria.p__Planctomycetes.c__Planctomycetacia.o__Gemmatales.f__Gemmataceae.g__Gemmata.s__obscuriglobus.t__UQM_2246
d__Bacteria.p__Verrucomicrobia.c__Opitutae.o__Puniceicoccales.f__Puniceicoccaceae.g__Coraliomargarita.s__akajimensis.t__DSM_45221
d__Bacteria.p__Verrucomicrobia.c__Opitutae.o__Opitutales.f__Opitutaceae.g__unclassified.s__bacterium.t__TAV2
d__Bacteria.p__Verrucomicrobia.c__Opitutae.o__Opitutales.f__Opitutaceae.g__Opitutus.s__terrae.t__PB90_1
bin__115_10132015
bin__222_10132015
bin__269_10132015
d__Bacteria.p__Verrucomicrobia.c__Verrucomicrobiae.o__Verrucomicrobiales.f__unclassified.g__unclassified.s__bacterium.t__DG1235
d__Bacteria.p__Verrucomicrobia.c__unclassified.o__Methylacidiphilales.f__Methylacidiphilaceae.g__Methylacidiphilum.s__infernorum.t__V4
bin__216_10132015
bin__174_10132015
bin__163_10132015
bin__249_10132015
bin__232_10132015
bin__208_10132015
bin__240_10132015
bin__258_10132015
bin__119_10132015
bin__118_10132015
bin__196_10132015
d__Bacteria.p__Verrucomicrobia.c__Verrucomicrobiae.o__Verrucomicrobiales.f__Verrucomicrobia_subdivision_3.g__unclassified.s__Ellin514.t__1
bin__237_10132015
bin__187_10132015
bin__156_10132015
bin__44_10132015
bin__189_10132015
bin__102_10132015
bin__24_10132015
bin__192_10132015
bin__245_10132015
d__Bacteria.p__Verrucomicrobia.c__Spartobacteria.o__unclassified.f__unclassified.g__Chthoniobacter.s__flavus.t__Ellin428
bin__142_10132015
bin__100_10132015
d__Bacteria.p__Verrucomicrobia.c__Verrucomicrobiae.o__Verrucomicrobiales.f__Verrucomicrobiaceae.g__Verrucomicrobium.s__spinosum.t__DSM_4136
d__Bacteria.p__Verrucomicrobia.c__Verrucomicrobiae.o__Verrucomicrobiales.f__Verrucomicrobiaceae.g__Akkermansia.s__muciniphila.t__ATCC_BAA_835
bin__243_10132015
bin__202_10132015
bin__152_10132015
bin__185_10132015
d__Bacteria.p__Lentisphaerae.c__Lentisphaerae.o__Lentisphaerales.f__Lentisphaeraceae.g__Lentisphaera.s__araneosa.t__HTCC2155
d__Bacteria.p__Lentisphaerae.c__Lentisphaerae.o__Victivallales.f__Victivallaceae.g__Victivallis.s__vadensis.t__ATCC_BAA_548
bin__56_10132015
bin__158_10132015
bin__180_10132015
bin__135_10132015
bin__176_10132015
bin__270_10132015
bin__98_10142015
bin__193_10132015
bin__188_10132015
bin__147_10132015
bin__132_10132015
AP_Chloroflexi_bacterium_JGI_002000_F101345071464409
AP_Chloroflexi_bacterium_JGI_002000_F071345071464210
bin__71_10142015
bin__104_10132015
bin__157_10132015
bin__117_10132015
bin__57_10132015
bin__248_10132015
bin__86_10142015
bin__48_10132015
bin__99_10142015
bin__107_10132015
bin__182_10132015
d__Bacteria.p__Acidobacteria.c__Solibacteres.o__Solibacterales.f__Solibacteraceae.g__Solibacter.s__usitatus.t__Ellin6076
d__Bacteria.p__Acidobacteria.c__Acidobacteria.o__Acidobacteriales.f__Korebacteraceae.g__Korebacter.s__versatilis.t__Ellin345
d__Bacteria.p__Acidobacteria.c__Acidobacteria.o__Acidobacteriales.f__Acidobacteriaceae.g__Acidobacterium.s__capsulatum.t__ATCC_51196
d__Bacteria.p__Acidobacteria.c__Acidobacteria.o__Acidobacteriales.f__Acidobacteriaceae.g__Terriglobus.s__saanensis.t__SP1PR4
d__Bacteria.p__Acidobacteria.c__Acidobacteria.o__Acidobacteriales.f__Acidobacteriaceae.g__Granulicella.s__sp_.t__MP5ACTX8
bin__80_10142015
d__Bacteria.p__Acidobacteria.c__Acidobacteria.o__Acidobacteriales.f__Acidobacteriaceae.g__Granulicella.s__sp_.t__MP5ACTX9
bin__105_10132015
bin__128_10132015
bin__209_10132015
bin__244_10132015
bin__266_10132015
bin__250_10132015
bin__130_10132015
AP_Chloroflexi_bacterium_JGI_0000112_G221336607949198
bin__265_10132015
bin__162_10132015
bin__252_10132015
candidatus_methylomirabilis_oxyfera
Candidatus_Magnetobacterium_sp_09242015
Thermodesulfovibrio_thiophilus_DSM_17215_09242015
Thermodesulfovibrio_islandicus_DSM_12570_09242015
d__Bacteria.p__Nitrospirae.c__Nitrospira.o__Nitrospirales.f__Nitrospiraceae.g__Thermodesulfovibrio.s__yellowstonii.t__DSM_11347
Thermodesulfovibrio_yellowstonii_DSM_11347_09242015
AP_Nitrospirae_bacterium_SCGC_AC_310_E051362021929723_09242015
AP_Nitrospirae_bacterium_SCGC_AC_310_I221362021930136_09242015
Leptospirillum_sp_09242015
Leptospirillum_ferrooxidans_C2_3_09242015
Leptospirillum_ferriphilum_DSM_14647_09242015
Leptospirillum_ferriphilum_YSK_09242015
Leptospirillum_ferriphilum_ML_04_09242015
Nitrospira_marina_Nb_295_09242015
d__Bacteria.p__Nitrospirae.c__Nitrospira.o__Nitrospirales.f__Nitrospiraceae.g__Candidatus_Nitrospira.s__Nitrospira.t__defluvii
Candidatus_Nitrospira_defluvii_09242015

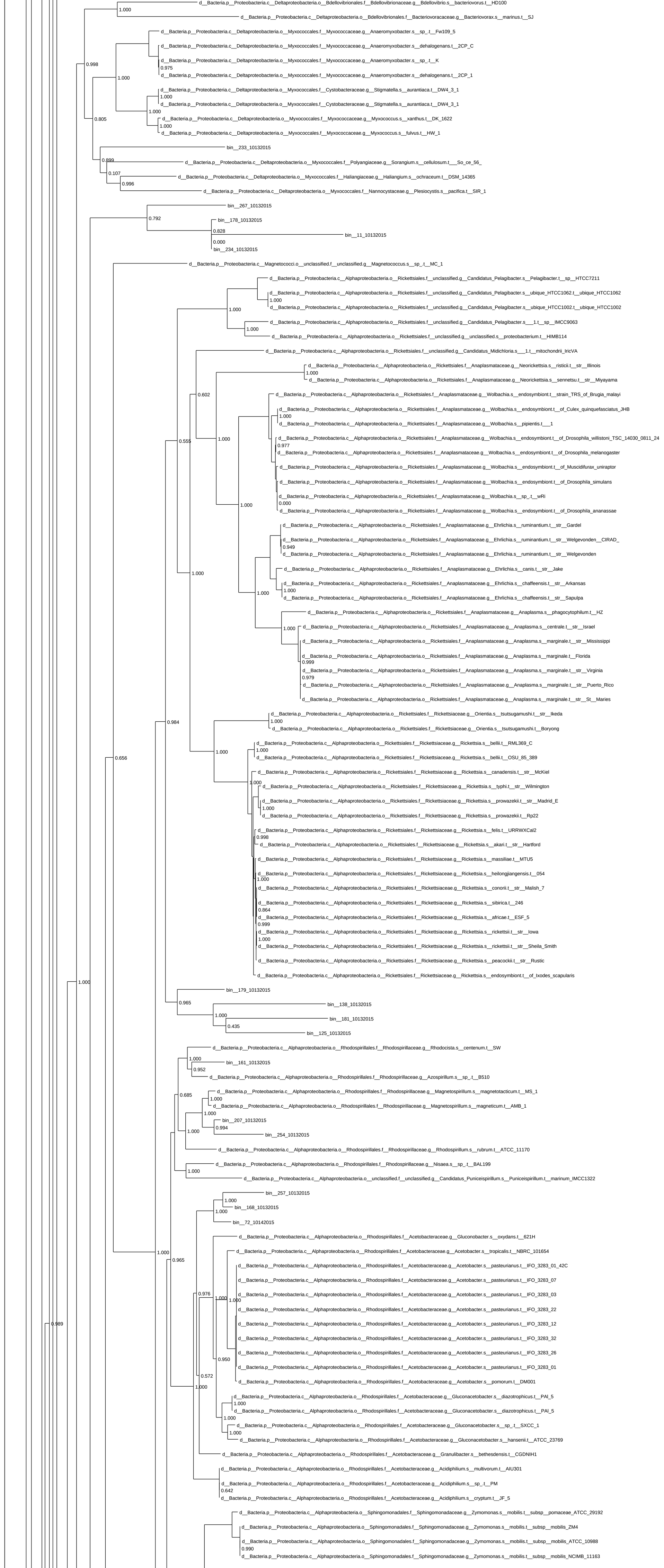
d__Bacteria.p__Proteobacteria.c__Deltaproteobacteria.o__Bdellovibrionales.f__Bdellovibrionaceae.g__Bdellovibrio.s__bacteriovorus.t__HD100
d__Bacteria.p__Proteobacteria.c__Deltaproteobacteria.o__Bdellovibrionales.f__Bacteriovoracaceae.g__Bacteriovorax.s__marinus.t__SJ
d__Bacteria.p__Proteobacteria.c__Deltaproteobacteria.o__Myxococcales.f__Myxococcaceae.g__Anaeromyxobacter.s__sp_.t__Fw109_5
d__Bacteria.p__Proteobacteria.c__Deltaproteobacteria.o__Myxococcales.f__Myxococcaceae.g__Anaeromyxobacter.s__dehalogenans.t__2CP_C
d__Bacteria.p__Proteobacteria.c__Deltaproteobacteria.o__Myxococcales.f__Myxococcaceae.g__Anaeromyxobacter.s__sp_.t__K
d__Bacteria.p__Proteobacteria.c__Deltaproteobacteria.o__Myxococcales.f__Myxococcaceae.g__Anaeromyxobacter.s__dehalogenans.t__2CP_1
d__Bacteria.p__Proteobacteria.c__Deltaproteobacteria.o__Myxococcales.f__Cystobacteraceae.g__Stigmatella.s__aurantiaca.t__DW4_3_1
d__Bacteria.p__Proteobacteria.c__Deltaproteobacteria.o__Myxococcales.f__Cystobacteraceae.g__Stigmatella.s__aurantiaca.t__DW4_3_1
d__Bacteria.p__Proteobacteria.c__Deltaproteobacteria.o__Myxococcales.f__Myxococcaceae.g__Myxococcus.s__xanthus.t__DK_1622
d__Bacteria.p__Proteobacteria.c__Deltaproteobacteria.o__Myxococcales.f__Myxococcaceae.g__Myxococcus.s__fulvus.t__HW_1
bin__233_10132015
d__Bacteria.p__Proteobacteria.c__Deltaproteobacteria.o__Myxococcales.f__Polyangiaceae.g__Sorangium.s__cellulosum.t___So_ce_56_
d__Bacteria.p__Proteobacteria.c__Deltaproteobacteria.o__Myxococcales.f__Haliangiaceae.g__Haliangium.s__ochraceum.t__DSM_14365
d__Bacteria.p__Proteobacteria.c__Deltaproteobacteria.o__Myxococcales.f__Nannocystaceae.g__Plesiocystis.s__pacifica.t__SIR_1
bin__267_10132015
bin__178_10132015
bin__11_10132015
bin__234_10132015
d__Bacteria.p__Proteobacteria.c__Magnetococci.o__unclassified.f__unclassified.g__Magnetococcus.s__sp_.t__MC_1
d__Bacteria.p__Proteobacteria.c__Alphaproteobacteria.o__Rickettsiales.f__unclassified.g__Candidatus_Pelagibacter.s__Pelagibacter.t__sp__HTCC7211
d__Bacteria.p__Proteobacteria.c__Alphaproteobacteria.o__Rickettsiales.f__unclassified.g__Candidatus_Pelagibacter.s__ubique_HTCC1062.t__ubique_HTCC1062
d__Bacteria.p__Proteobacteria.c__Alphaproteobacteria.o__Rickettsiales.f__unclassified.g__Candidatus_Pelagibacter.s__ubique_HTCC1002.t__ubique_HTCC1002
d__Bacteria.p__Proteobacteria.c__Alphaproteobacteria.o__Rickettsiales.f__unclassified.g__Candidatus_Pelagibacter.s___1.t__sp__IMCC9063
d__Bacteria.p__Proteobacteria.c__Alphaproteobacteria.o__Rickettsiales.f__unclassified.g__unclassified.s__proteobacterium.t__HIMB114
d__Bacteria.p__Proteobacteria.c__Alphaproteobacteria.o__Rickettsiales.f__unclassified.g__Candidatus_Midichloria.s___1.t__mitochondrii_IricVA
d__Bacteria.p__Proteobacteria.c__Alphaproteobacteria.o__Rickettsiales.f__Anaplasmataceae.g__Neorickettsia.s__risticii.t__str__Illinois
d__Bacteria.p__Proteobacteria.c__Alphaproteobacteria.o__Rickettsiales.f__Anaplasmataceae.g__Neorickettsia.s__sennetsu.t__str__Miyayama
d__Bacteria.p__Proteobacteria.c__Alphaproteobacteria.o__Rickettsiales.f__Anaplasmataceae.g__Wolbachia.s__endosymbiont.t__strain_TRS_of_Brugia_malayi
d__Bacteria.p__Proteobacteria.c__Alphaproteobacteria.o__Rickettsiales.f__Anaplasmataceae.g__Wolbachia.s__endosymbiont.t__of_Culex_quinquefasciatus_JHB
d__Bacteria.p__Proteobacteria.c__Alphaproteobacteria.o__Rickettsiales.f__Anaplasmataceae.g__Wolbachia.s__pipientis.t___1
d__Bacteria.p__Proteobacteria.c__Alphaproteobacteria.o__Rickettsiales.f__Anaplasmataceae.g__Wolbachia.s__endosymbiont.t__of_Drosophila_willistoni_TSC_14030_0811_24
d__Bacteria.p__Proteobacteria.c__Alphaproteobacteria.o__Rickettsiales.f__Anaplasmataceae.g__Wolbachia.s__endosymbiont.t__of_Drosophila_melanogaster
d__Bacteria.p__Proteobacteria.c__Alphaproteobacteria.o__Rickettsiales.f__Anaplasmataceae.g__Wolbachia.s__endosymbiont.t__of_Muscidifurax_uniraptor
d__Bacteria.p__Proteobacteria.c__Alphaproteobacteria.o__Rickettsiales.f__Anaplasmataceae.g__Wolbachia.s__endosymbiont.t__of_Drosophila_simulans
d__Bacteria.p__Proteobacteria.c__Alphaproteobacteria.o__Rickettsiales.f__Anaplasmataceae.g__Wolbachia.s__sp_.t__wRi
d__Bacteria.p__Proteobacteria.c__Alphaproteobacteria.o__Rickettsiales.f__Anaplasmataceae.g__Wolbachia.s__endosymbiont.t__of_Drosophila_ananassae
d__Bacteria.p__Proteobacteria.c__Alphaproteobacteria.o__Rickettsiales.f__Anaplasmataceae.g__Ehrlichia.s__ruminantium.t__str__Gardel
d__Bacteria.p__Proteobacteria.c__Alphaproteobacteria.o__Rickettsiales.f__Anaplasmataceae.g__Ehrlichia.s__ruminantium.t__str__Welgevonden__CIRAD_
d__Bacteria.p__Proteobacteria.c__Alphaproteobacteria.o__Rickettsiales.f__Anaplasmataceae.g__Ehrlichia.s__ruminantium.t__str__Welgevonden
d__Bacteria.p__Proteobacteria.c__Alphaproteobacteria.o__Rickettsiales.f__Anaplasmataceae.g__Ehrlichia.s__canis.t__str__Jake
d__Bacteria.p__Proteobacteria.c__Alphaproteobacteria.o__Rickettsiales.f__Anaplasmataceae.g__Ehrlichia.s__chaffeensis.t__str__Arkansas
d__Bacteria.p__Proteobacteria.c__Alphaproteobacteria.o__Rickettsiales.f__Anaplasmataceae.g__Ehrlichia.s__chaffeensis.t__str__Sapulpa
d__Bacteria.p__Proteobacteria.c__Alphaproteobacteria.o__Rickettsiales.f__Anaplasmataceae.g__Anaplasma.s__phagocytophilum.t__HZ
d__Bacteria.p__Proteobacteria.c__Alphaproteobacteria.o__Rickettsiales.f__Anaplasmataceae.g__Anaplasma.s__centrale.t__str__Israel
d__Bacteria.p__Proteobacteria.c__Alphaproteobacteria.o__Rickettsiales.f__Anaplasmataceae.g__Anaplasma.s__marginale.t__str__Mississippi
d__Bacteria.p__Proteobacteria.c__Alphaproteobacteria.o__Rickettsiales.f__Anaplasmataceae.g__Anaplasma.s__marginale.t__Florida
d__Bacteria.p__Proteobacteria.c__Alphaproteobacteria.o__Rickettsiales.f__Anaplasmataceae.g__Anaplasma.s__marginale.t__str__Virginia
d__Bacteria.p__Proteobacteria.c__Alphaproteobacteria.o__Rickettsiales.f__Anaplasmataceae.g__Anaplasma.s__marginale.t__str__Puerto_Rico
d__Bacteria.p__Proteobacteria.c__Alphaproteobacteria.o__Rickettsiales.f__Anaplasmataceae.g__Anaplasma.s__marginale.t__str__St__Maries
d__Bacteria.p__Proteobacteria.c__Alphaproteobacteria.o__Rickettsiales.f__Rickettsiaceae.g__Orientia.s__tsutsugamushi.t__str__Ikeda
d__Bacteria.p__Proteobacteria.c__Alphaproteobacteria.o__Rickettsiales.f__Rickettsiaceae.g__Orientia.s__tsutsugamushi.t__Boryong
d__Bacteria.p__Proteobacteria.c__Alphaproteobacteria.o__Rickettsiales.f__Rickettsiaceae.g__Rickettsia.s__bellii.t__RML369_C
d__Bacteria.p__Proteobacteria.c__Alphaproteobacteria.o__Rickettsiales.f__Rickettsiaceae.g__Rickettsia.s__bellii.t__OSU_85_389
d__Bacteria.p__Proteobacteria.c__Alphaproteobacteria.o__Rickettsiales.f__Rickettsiaceae.g__Rickettsia.s__canadensis.t__str__McKiel
d__Bacteria.p__Proteobacteria.c__Alphaproteobacteria.o__Rickettsiales.f__Rickettsiaceae.g__Rickettsia.s__typhi.t__str__Wilmington
d__Bacteria.p__Proteobacteria.c__Alphaproteobacteria.o__Rickettsiales.f__Rickettsiaceae.g__Rickettsia.s__prowazekii.t__str__Madrid_E
d__Bacteria.p__Proteobacteria.c__Alphaproteobacteria.o__Rickettsiales.f__Rickettsiaceae.g__Rickettsia.s__prowazekii.t__Rp22
d__Bacteria.p__Proteobacteria.c__Alphaproteobacteria.o__Rickettsiales.f__Rickettsiaceae.g__Rickettsia.s__felis.t__URRWXCal2
d__Bacteria.p__Proteobacteria.c__Alphaproteobacteria.o__Rickettsiales.f__Rickettsiaceae.g__Rickettsia.s__akari.t__str__Hartford
d__Bacteria.p__Proteobacteria.c__Alphaproteobacteria.o__Rickettsiales.f__Rickettsiaceae.g__Rickettsia.s__massiliae.t__MTU5
d__Bacteria.p__Proteobacteria.c__Alphaproteobacteria.o__Rickettsiales.f__Rickettsiaceae.g__Rickettsia.s__heilongjiangensis.t__054
d__Bacteria.p__Proteobacteria.c__Alphaproteobacteria.o__Rickettsiales.f__Rickettsiaceae.g__Rickettsia.s__conorii.t__str__Malish_7
d__Bacteria.p__Proteobacteria.c__Alphaproteobacteria.o__Rickettsiales.f__Rickettsiaceae.g__Rickettsia.s__sibirica.t__246
d__Bacteria.p__Proteobacteria.c__Alphaproteobacteria.o__Rickettsiales.f__Rickettsiaceae.g__Rickettsia.s__africae.t__ESF_5
d__Bacteria.p__Proteobacteria.c__Alphaproteobacteria.o__Rickettsiales.f__Rickettsiaceae.g__Rickettsia.s__rickettsii.t__str__Iowa
d__Bacteria.p__Proteobacteria.c__Alphaproteobacteria.o__Rickettsiales.f__Rickettsiaceae.g__Rickettsia.s__rickettsii.t__str__Sheila_Smith
d__Bacteria.p__Proteobacteria.c__Alphaproteobacteria.o__Rickettsiales.f__Rickettsiaceae.g__Rickettsia.s__peacockii.t__str__Rustic
d__Bacteria.p__Proteobacteria.c__Alphaproteobacteria.o__Rickettsiales.f__Rickettsiaceae.g__Rickettsia.s__endosymbiont.t__of_Ixodes_scapularis
bin__179_10132015
bin__138_10132015
bin__181_10132015
bin__125_10132015
d__Bacteria.p__Proteobacteria.c__Alphaproteobacteria.o__Rhodospirillales.f__Rhodospirillaceae.g__Rhodocista.s__centenum.t__SW
bin__161_10132015
d__Bacteria.p__Proteobacteria.c__Alphaproteobacteria.o__Rhodospirillales.f__Rhodospirillaceae.g__Azospirillum.s__sp_.t__B510
d__Bacteria.p__Proteobacteria.c__Alphaproteobacteria.o__Rhodospirillales.f__Rhodospirillaceae.g__Magnetospirillum.s__magnetotacticum.t__MS_1
d__Bacteria.p__Proteobacteria.c__Alphaproteobacteria.o__Rhodospirillales.f__Rhodospirillaceae.g__Magnetospirillum.s__magneticum.t__AMB_1
bin__207_10132015
bin__254_10132015
d__Bacteria.p__Proteobacteria.c__Alphaproteobacteria.o__Rhodospirillales.f__Rhodospirillaceae.g__Rhodospirillum.s__rubrum.t__ATCC_11170
d__Bacteria.p__Proteobacteria.c__Alphaproteobacteria.o__Rhodospirillales.f__Rhodospirillaceae.g__Nisaea.s__sp_.t__BAL199
d__Bacteria.p__Proteobacteria.c__Alphaproteobacteria.o__unclassified.f__unclassified.g__Candidatus_Puniceispirillum.s__Puniceispirillum.t__marinum_IMCC1322
bin__257_10132015
bin__168_10132015
bin__72_10142015
d__Bacteria.p__Proteobacteria.c__Alphaproteobacteria.o__Rhodospirillales.f__Acetobacteraceae.g__Gluconobacter.s__oxydans.t__621H
d__Bacteria.p__Proteobacteria.c__Alphaproteobacteria.o__Rhodospirillales.f__Acetobacteraceae.g__Acetobacter.s__tropicalis.t__NBRC_101654
d__Bacteria.p__Proteobacteria.c__Alphaproteobacteria.o__Rhodospirillales.f__Acetobacteraceae.g__Acetobacter.s__pasteurianus.t__IFO_3283_01_42C
d__Bacteria.p__Proteobacteria.c__Alphaproteobacteria.o__Rhodospirillales.f__Acetobacteraceae.g__Acetobacter.s__pasteurianus.t__IFO_3283_07
d__Bacteria.p__Proteobacteria.c__Alphaproteobacteria.o__Rhodospirillales.f__Acetobacteraceae.g__Acetobacter.s__pasteurianus.t__IFO_3283_03
d__Bacteria.p__Proteobacteria.c__Alphaproteobacteria.o__Rhodospirillales.f__Acetobacteraceae.g__Acetobacter.s__pasteurianus.t__IFO_3283_22
d__Bacteria.p__Proteobacteria.c__Alphaproteobacteria.o__Rhodospirillales.f__Acetobacteraceae.g__Acetobacter.s__pasteurianus.t__IFO_3283_12
d__Bacteria.p__Proteobacteria.c__Alphaproteobacteria.o__Rhodospirillales.f__Acetobacteraceae.g__Acetobacter.s__pasteurianus.t__IFO_3283_32
d__Bacteria.p__Proteobacteria.c__Alphaproteobacteria.o__Rhodospirillales.f__Acetobacteraceae.g__Acetobacter.s__pasteurianus.t__IFO_3283_26
d__Bacteria.p__Proteobacteria.c__Alphaproteobacteria.o__Rhodospirillales.f__Acetobacteraceae.g__Acetobacter.s__pasteurianus.t__IFO_3283_01
d__Bacteria.p__Proteobacteria.c__Alphaproteobacteria.o__Rhodospirillales.f__Acetobacteraceae.g__Acetobacter.s__pomorum.t__DM001
d__Bacteria.p__Proteobacteria.c__Alphaproteobacteria.o__Rhodospirillales.f__Acetobacteraceae.g__Gluconacetobacter.s__diazotrophicus.t__PAl_5
d__Bacteria.p__Proteobacteria.c__Alphaproteobacteria.o__Rhodospirillales.f__Acetobacteraceae.g__Gluconacetobacter.s__diazotrophicus.t__PAl_5
d__Bacteria.p__Proteobacteria.c__Alphaproteobacteria.o__Rhodospirillales.f__Acetobacteraceae.g__Gluconacetobacter.s__sp_.t__SXCC_1
d__Bacteria.p__Proteobacteria.c__Alphaproteobacteria.o__Rhodospirillales.f__Acetobacteraceae.g__Gluconacetobacter.s__hansenii.t__ATCC_23769
d__Bacteria.p__Proteobacteria.c__Alphaproteobacteria.o__Rhodospirillales.f__Acetobacteraceae.g__Granulibacter.s__bethesdensis.t__CGDNIH1
d__Bacteria.p__Proteobacteria.c__Alphaproteobacteria.o__Rhodospirillales.f__Acetobacteraceae.g__Acidiphilium.s__multivorum.t__AIU301
d__Bacteria.p__Proteobacteria.c__Alphaproteobacteria.o__Rhodospirillales.f__Acetobacteraceae.g__Acidiphilium.s__sp_.t__PM
d__Bacteria.p__Proteobacteria.c__Alphaproteobacteria.o__Rhodospirillales.f__Acetobacteraceae.g__Acidiphilium.s__cryptum.t__JF_5
d__Bacteria.p__Proteobacteria.c__Alphaproteobacteria.o__Sphingomonadales.f__Sphingomonadaceae.g__Zymomonas.s__mobilis.t__subsp__pomaceae_ATCC_29192
d__Bacteria.p__Proteobacteria.c__Alphaproteobacteria.o__Sphingomonadales.f__Sphingomonadaceae.g__Zymomonas.s__mobilis.t__subsp__mobilis_ZM4
d__Bacteria.p__Proteobacteria.c__Alphaproteobacteria.o__Sphingomonadales.f__Sphingomonadaceae.g__Zymomonas.s__mobilis.t__subsp__mobilis_ATCC_10988
d__Bacteria.p__Proteobacteria.c__Alphaproteobacteria.o__Sphingomonadales.f__Sphingomonadaceae.g__Zymomonas.s__mobilis.t__subsp__mobilis_NCIMB_11163

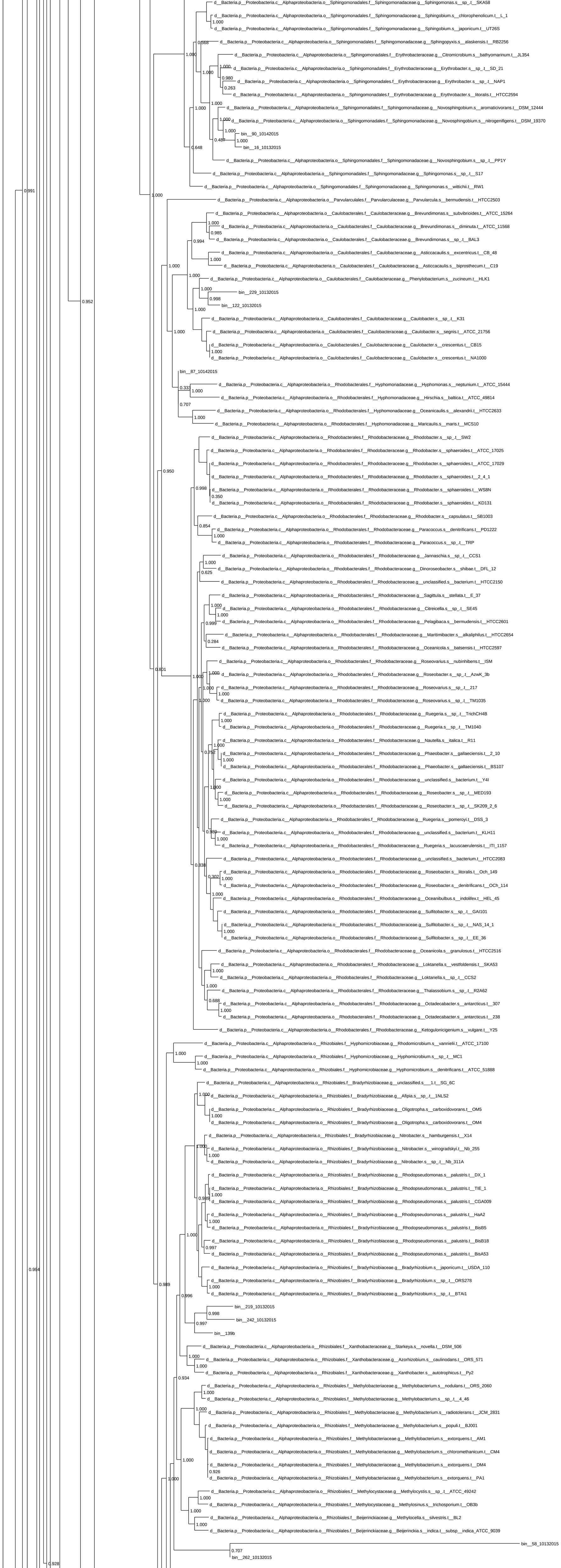

d__Bacteria.p__Proteobacteria.c__Alphaproteobacteria.o__Sphingomonadales.f__Sphingomonadaceae.g__Sphingomonas.s__sp._t__SKA58
d__Bacteria.p__Proteobacteria.c__Alphaproteobacteria.o__Sphingomonadales.f__Sphingomonadaceae.g__Sphingobium.s__chlorophenolicum.t__L_1
d__Bacteria.p__Proteobacteria.c__Alphaproteobacteria.o__Sphingomonadales.f__Sphingomonadaceae.g__Sphingobium.s__japonicum.t__UT26S
d__Bacteria.p__Proteobacteria.c__Alphaproteobacteria.o__Sphingomonadales.f__Sphingomonadaceae.g__Sphingopyxis.s__alaskensis.t__RB2256
d__Bacteria.p__Proteobacteria.c__Alphaproteobacteria.o__Sphingomonadales.f__Erythrobacteraceae.g__Citromicrobium.s__bathyomarinum.t__JL354
d__Bacteria.p__Proteobacteria.c__Alphaproteobacteria.o__Sphingomonadales.f__Erythrobacteraceae.g__Erythrobacter.s__sp._t__SD_21
d__Bacteria.p__Proteobacteria.c__Alphaproteobacteria.o__Sphingomonadales.f__Erythrobacteraceae.g__Erythrobacter.s__sp._t__NAP1
d__Bacteria.p__Proteobacteria.c__Alphaproteobacteria.o__Sphingomonadales.f__Erythrobacteraceae.g__Erythrobacter.s__litoralis.t__HTCC2594
d__Bacteria.p__Proteobacteria.c__Alphaproteobacteria.o__Sphingomonadales.f__Sphingomonadaceae.g__Novosphingobium.s__aromaticivorans.t__DSM_12444
d__Bacteria.p__Proteobacteria.c__Alphaproteobacteria.o__Sphingomonadales.f__Sphingomonadaceae.g__Novosphingobium.s__nitrogenifigens.t__DSM_19370
bin__90_10142015
bin__16_10132015
d__Bacteria.p__Proteobacteria.c__Alphaproteobacteria.o__Sphingomonadales.f__Sphingomonadaceae.g__Novosphingobium.s__sp._t__PP1Y
d__Bacteria.p__Proteobacteria.c__Alphaproteobacteria.o__Sphingomonadales.f__Sphingomonadaceae.g__Sphingomonas.s__sp._t__S17
d__Bacteria.p__Proteobacteria.c__Alphaproteobacteria.o__Sphingomonadales.f__Sphingomonadaceae.g__Sphingomonas.s__wittichii.t__RW1
d__Bacteria.p__Proteobacteria.c__Alphaproteobacteria.o__Parvularculales.f__Parvularculaceae.g__Parvularcula.s__bermudensis.t__HTCC2503
d__Bacteria.p__Proteobacteria.c__Alphaproteobacteria.o__Caulobacterales.f__Caulobacteraceae.g__Brevundimonas.s__subvibrioides.t__ATCC_15264
d__Bacteria.p__Proteobacteria.c__Alphaproteobacteria.o__Caulobacterales.f__Caulobacteraceae.g__Brevundimonas.s__diminuta.t__ATCC_11568
d__Bacteria.p__Proteobacteria.c__Alphaproteobacteria.o__Caulobacterales.f__Caulobacteraceae.g__Brevundimonas.s__sp._t__BAL3
d__Bacteria.p__Proteobacteria.c__Alphaproteobacteria.o__Caulobacterales.f__Caulobacteraceae.g__Asticcacaulis.s__excentricus.t__CB_48
d__Bacteria.p__Proteobacteria.c__Alphaproteobacteria.o__Caulobacterales.f__Caulobacteraceae.g__Asticcacaulis.s__biprosthecum.t__C19
d__Bacteria.p__Proteobacteria.c__Alphaproteobacteria.o__Caulobacterales.f__Caulobacteraceae.g__Phenylobacterium.s__zucineum.t__HLK1
bin__229_10132015
bin__122_10132015
d__Bacteria.p__Proteobacteria.c__Alphaproteobacteria.o__Caulobacterales.f__Caulobacteraceae.g__Caulobacter.s__sp._t__K31
d__Bacteria.p__Proteobacteria.c__Alphaproteobacteria.o__Caulobacterales.f__Caulobacteraceae.g__Caulobacter.s__segnis.t__ATCC_21756
d__Bacteria.p__Proteobacteria.c__Alphaproteobacteria.o__Caulobacterales.f__Caulobacteraceae.g__Caulobacter.s__crescentus.t__CB15
d__Bacteria.p__Proteobacteria.c__Alphaproteobacteria.o__Caulobacterales.f__Caulobacteraceae.g__Caulobacter.s__crescentus.t__NA1000
bin__87_10142015
d__Bacteria.p__Proteobacteria.c__Alphaproteobacteria.o__Rhodobacterales.f__Hyphomonadaceae.g__Hyphomonas.s__neptunium.t__ATCC_15444
d__Bacteria.p__Proteobacteria.c__Alphaproteobacteria.o__Rhodobacterales.f__Hyphomonadaceae.g__Hirschia.s__baltica.t__ATCC_49814
d__Bacteria.p__Proteobacteria.c__Alphaproteobacteria.o__Rhodobacterales.f__Hyphomonadaceae.g__Oceanicaulis.s__alexandrii.t__HTCC2633
d__Bacteria.p__Proteobacteria.c__Alphaproteobacteria.o__Rhodobacterales.f__Hyphomonadaceae.g__Maricaulis.s__maris.t__MCS10
d__Bacteria.p__Proteobacteria.c__Alphaproteobacteria.o__Rhodobacterales.f__Rhodobacteraceae.g__Rhodobacter.s__sp._t__SW2
d__Bacteria.p__Proteobacteria.c__Alphaproteobacteria.o__Rhodobacterales.f__Rhodobacteraceae.g__Rhodobacter.s__sphaeroides.t__ATCC_17025
d__Bacteria.p__Proteobacteria.c__Alphaproteobacteria.o__Rhodobacterales.f__Rhodobacteraceae.g__Rhodobacter.s__sphaeroides.t__ATCC_17029
d__Bacteria.p__Proteobacteria.c__Alphaproteobacteria.o__Rhodobacterales.f__Rhodobacteraceae.g__Rhodobacter.s__sphaeroides.t__2_4_1
d__Bacteria.p__Proteobacteria.c__Alphaproteobacteria.o__Rhodobacterales.f__Rhodobacteraceae.g__Rhodobacter.s__sphaeroides.t__WS8N
d__Bacteria.p__Proteobacteria.c__Alphaproteobacteria.o__Rhodobacterales.f__Rhodobacteraceae.g__Rhodobacter.s__sphaeroides.t__KD131
d__Bacteria.p__Proteobacteria.c__Alphaproteobacteria.o__Rhodobacterales.f__Rhodobacteraceae.g__Rhodobacter.s__capsulatus.t__SB1003
d__Bacteria.p__Proteobacteria.c__Alphaproteobacteria.o__Rhodobacterales.f__Rhodobacteraceae.g__Paracoccus.s__denitrificans.t__PD1222
d__Bacteria.p__Proteobacteria.c__Alphaproteobacteria.o__Rhodobacterales.f__Rhodobacteraceae.g__Paracoccus.s__sp._t__TRP
d__Bacteria.p__Proteobacteria.c__Alphaproteobacteria.o__Rhodobacterales.f__Rhodobacteraceae.g__Jannaschia.s__sp._t__CCS1
d__Bacteria.p__Proteobacteria.c__Alphaproteobacteria.o__Rhodobacterales.f__Rhodobacteraceae.g__Dinoroseobacter.s__shibae.t__DFL_12
d__Bacteria.p__Proteobacteria.c__Alphaproteobacteria.o__Rhodobacterales.f__Rhodobacteraceae.g__unclassified.s__bacterium.t__HTCC2150
d__Bacteria.p__Proteobacteria.c__Alphaproteobacteria.o__Rhodobacterales.f__Rhodobacteraceae.g__Sagittula.s__stellata.t__E_37
d__Bacteria.p__Proteobacteria.c__Alphaproteobacteria.o__Rhodobacterales.f__Rhodobacteraceae.g__Citreicella.s__sp._t__SE45
d__Bacteria.p__Proteobacteria.c__Alphaproteobacteria.o__Rhodobacterales.f__Rhodobacteraceae.g__Pelagibaca.s__bermudensis.t__HTCC2601
d__Bacteria.p__Proteobacteria.c__Alphaproteobacteria.o__Rhodobacterales.f__Rhodobacteraceae.g__Maritimibacter.s__alkaliphilus.t__HTCC2654
d__Bacteria.p__Proteobacteria.c__Alphaproteobacteria.o__Rhodobacterales.f__Rhodobacteraceae.g__Oceanicola.s__batsensis.t__HTCC2597
d__Bacteria.p__Proteobacteria.c__Alphaproteobacteria.o__Rhodobacterales.f__Rhodobacteraceae.g__Roseovarius.s__nubinhibens.t__ISM
d__Bacteria.p__Proteobacteria.c__Alphaproteobacteria.o__Rhodobacterales.f__Rhodobacteraceae.g__Roseobacter.s__sp._t__AzwK_3b
d__Bacteria.p__Proteobacteria.c__Alphaproteobacteria.o__Rhodobacterales.f__Rhodobacteraceae.g__Roseovarius.s__sp._t__217
d__Bacteria.p__Proteobacteria.c__Alphaproteobacteria.o__Rhodobacterales.f__Rhodobacteraceae.g__Roseovarius.s__sp._t__TM1035
d__Bacteria.p__Proteobacteria.c__Alphaproteobacteria.o__Rhodobacterales.f__Rhodobacteraceae.g__Ruegeria.s__sp._t__TrichCH4B
d__Bacteria.p__Proteobacteria.c__Alphaproteobacteria.o__Rhodobacterales.f__Rhodobacteraceae.g__Ruegeria.s__sp._t__TM1040
d__Bacteria.p__Proteobacteria.c__Alphaproteobacteria.o__Rhodobacterales.f__Rhodobacteraceae.g__Nautella.s__italica.t__R11
d__Bacteria.p__Proteobacteria.c__Alphaproteobacteria.o__Rhodobacterales.f__Rhodobacteraceae.g__Phaeobacter.s__gallaeciensis.t__2_10
d__Bacteria.p__Proteobacteria.c__Alphaproteobacteria.o__Rhodobacterales.f__Rhodobacteraceae.g__Phaeobacter.s__gallaeciensis.t__BS107
d__Bacteria.p__Proteobacteria.c__Alphaproteobacteria.o__Rhodobacterales.f__Rhodobacteraceae.g__unclassified.s__bacterium.t__Y4I
d__Bacteria.p__Proteobacteria.c__Alphaproteobacteria.o__Rhodobacterales.f__Rhodobacteraceae.g__Roseobacter.s__sp._t__MED193
d__Bacteria.p__Proteobacteria.c__Alphaproteobacteria.o__Rhodobacterales.f__Rhodobacteraceae.g__Roseobacter.s__sp._t__SK209_2_6
d__Bacteria.p__Proteobacteria.c__Alphaproteobacteria.o__Rhodobacterales.f__Rhodobacteraceae.g__Ruegeria.s__pomeroyi.t__DSS_3
d__Bacteria.p__Proteobacteria.c__Alphaproteobacteria.o__Rhodobacterales.f__Rhodobacteraceae.g__unclassified.s__bacterium.t__KLH11
d__Bacteria.p__Proteobacteria.c__Alphaproteobacteria.o__Rhodobacterales.f__Rhodobacteraceae.g__Ruegeria.s__lacuscaerulensis.t__ITI_1157
d__Bacteria.p__Proteobacteria.c__Alphaproteobacteria.o__Rhodobacterales.f__Rhodobacteraceae.g__unclassified.s__bacterium.t__HTCC2083
d__Bacteria.p__Proteobacteria.c__Alphaproteobacteria.o__Rhodobacterales.f__Rhodobacteraceae.g__Roseobacter.s__litoralis.t__Och_149
d__Bacteria.p__Proteobacteria.c__Alphaproteobacteria.o__Rhodobacterales.f__Rhodobacteraceae.g__Roseobacter.s__denitrificans.t__OCh_114
d__Bacteria.p__Proteobacteria.c__Alphaproteobacteria.o__Rhodobacterales.f__Rhodobacteraceae.g__Oceanibulbus.s__indolifex.t__HEL_45
d__Bacteria.p__Proteobacteria.c__Alphaproteobacteria.o__Rhodobacterales.f__Rhodobacteraceae.g__Sulfitobacter.s__sp._t__GAI101
d__Bacteria.p__Proteobacteria.c__Alphaproteobacteria.o__Rhodobacterales.f__Rhodobacteraceae.g__Sulfitobacter.s__sp._t__NAS_14_1
d__Bacteria.p__Proteobacteria.c__Alphaproteobacteria.o__Rhodobacterales.f__Rhodobacteraceae.g__Sulfitobacter.s__sp._t__EE_36
d__Bacteria.p__Proteobacteria.c__Alphaproteobacteria.o__Rhodobacterales.f__Rhodobacteraceae.g__Oceanicola.s__granulosus.t__HTCC2516
d__Bacteria.p__Proteobacteria.c__Alphaproteobacteria.o__Rhodobacterales.f__Rhodobacteraceae.g__Loktanella.s__vestfoldensis.t__SKA53
d__Bacteria.p__Proteobacteria.c__Alphaproteobacteria.o__Rhodobacterales.f__Rhodobacteraceae.g__Loktanella.s__sp._t__CCS2
d__Bacteria.p__Proteobacteria.c__Alphaproteobacteria.o__Rhodobacterales.f__Rhodobacteraceae.g__Thalassobium.s__sp._t__R2A62
d__Bacteria.p__Proteobacteria.c__Alphaproteobacteria.o__Rhodobacterales.f__Rhodobacteraceae.g__Octadecabacter.s__antarcticus.t__307
d__Bacteria.p__Proteobacteria.c__Alphaproteobacteria.o__Rhodobacterales.f__Rhodobacteraceae.g__Octadecabacter.s__antarcticus.t__238
d__Bacteria.p__Proteobacteria.c__Alphaproteobacteria.o__Rhodobacterales.f__Rhodobacteraceae.g__Ketogulonicigenium.s__vulgare.t__Y25
d__Bacteria.p__Proteobacteria.c__Alphaproteobacteria.o__Rhizobiales.f__Hyphomicrobiaceae.g__Rhodomicrobium.s__vannielii.t__ATCC_17100
d__Bacteria.p__Proteobacteria.c__Alphaproteobacteria.o__Rhizobiales.f__Hyphomicrobiaceae.g__Hyphomicrobium.s__sp._t__MC1
d__Bacteria.p__Proteobacteria.c__Alphaproteobacteria.o__Rhizobiales.f__Hyphomicrobiaceae.g__Hyphomicrobium.s__denitrificans.t__ATCC_51888
d__Bacteria.p__Proteobacteria.c__Alphaproteobacteria.o__Rhizobiales.f__Bradyrhizobiaceae.g__unclassified.s__1.t__SG_6C
d__Bacteria.p__Proteobacteria.c__Alphaproteobacteria.o__Rhizobiales.f__Bradyrhizobiaceae.g__Afipia.s__sp._t__1NLS2
d__Bacteria.p__Proteobacteria.c__Alphaproteobacteria.o__Rhizobiales.f__Bradyrhizobiaceae.g__Oligotropha.s__carboxidovorans.t__OM5
d__Bacteria.p__Proteobacteria.c__Alphaproteobacteria.o__Rhizobiales.f__Bradyrhizobiaceae.g__Oligotropha.s__carboxidovorans.t__OM4
d__Bacteria.p__Proteobacteria.c__Alphaproteobacteria.o__Rhizobiales.f__Bradyrhizobiaceae.g__Nitrobacter.s__hamburgensis.t__X14
d__Bacteria.p__Proteobacteria.c__Alphaproteobacteria.o__Rhizobiales.f__Bradyrhizobiaceae.g__Nitrobacter.s__winogradskyi.t__Nb_255
d__Bacteria.p__Proteobacteria.c__Alphaproteobacteria.o__Rhizobiales.f__Bradyrhizobiaceae.g__Nitrobacter.s__sp._t__Nb_311A
d__Bacteria.p__Proteobacteria.c__Alphaproteobacteria.o__Rhizobiales.f__Bradyrhizobiaceae.g__Rhodopseudomonas.s__palustris.t__DX_1
d__Bacteria.p__Proteobacteria.c__Alphaproteobacteria.o__Rhizobiales.f__Bradyrhizobiaceae.g__Rhodopseudomonas.s__palustris.t__TIE_1
d__Bacteria.p__Proteobacteria.c__Alphaproteobacteria.o__Rhizobiales.f__Bradyrhizobiaceae.g__Rhodopseudomonas.s__palustris.t__CGA009
d__Bacteria.p__Proteobacteria.c__Alphaproteobacteria.o__Rhizobiales.f__Bradyrhizobiaceae.g__Rhodopseudomonas.s__palustris.t__HaA2
d__Bacteria.p__Proteobacteria.c__Alphaproteobacteria.o__Rhizobiales.f__Bradyrhizobiaceae.g__Rhodopseudomonas.s__palustris.t__BisB5
d__Bacteria.p__Proteobacteria.c__Alphaproteobacteria.o__Rhizobiales.f__Bradyrhizobiaceae.g__Rhodopseudomonas.s__palustris.t__BisB18
d__Bacteria.p__Proteobacteria.c__Alphaproteobacteria.o__Rhizobiales.f__Bradyrhizobiaceae.g__Rhodopseudomonas.s__palustris.t__BisA53
d__Bacteria.p__Proteobacteria.c__Alphaproteobacteria.o__Rhizobiales.f__Bradyrhizobiaceae.g__Bradyrhizobium.s__japonicum.t__USDA_110
d__Bacteria.p__Proteobacteria.c__Alphaproteobacteria.o__Rhizobiales.f__Bradyrhizobiaceae.g__Bradyrhizobium.s__sp._t__ORS278
d__Bacteria.p__Proteobacteria.c__Alphaproteobacteria.o__Rhizobiales.f__Bradyrhizobiaceae.g__Bradyrhizobium.s__sp._t__BTAi1
bin__219_10132015
bin__242_10132015
bin__139b
d__Bacteria.p__Proteobacteria.c__Alphaproteobacteria.o__Rhizobiales.f__Xanthobacteraceae.g__Starkeya.s__novella.t__DSM_506
d__Bacteria.p__Proteobacteria.c__Alphaproteobacteria.o__Rhizobiales.f__Xanthobacteraceae.g__Azorhizobium.s__caulinodans.t__ORS_571
d__Bacteria.p__Proteobacteria.c__Alphaproteobacteria.o__Rhizobiales.f__Xanthobacteraceae.g__Xanthobacter.s__autotrophicus.t__Py2
d__Bacteria.p__Proteobacteria.c__Alphaproteobacteria.o__Rhizobiales.f__Methylobacteriaceae.g__Methylobacterium.s__nodulans.t__ORS_2060
d__Bacteria.p__Proteobacteria.c__Alphaproteobacteria.o__Rhizobiales.f__Methylobacteriaceae.g__Methylobacterium.s__sp._t__4_46
d__Bacteria.p__Proteobacteria.c__Alphaproteobacteria.o__Rhizobiales.f__Methylobacteriaceae.g__Methylobacterium.s__radiotolerans.t__JCM_2831
d__Bacteria.p__Proteobacteria.c__Alphaproteobacteria.o__Rhizobiales.f__Methylobacteriaceae.g__Methylobacterium.s__populi.t__BJ001
d__Bacteria.p__Proteobacteria.c__Alphaproteobacteria.o__Rhizobiales.f__Methylobacteriaceae.g__Methylobacterium.s__extorquens.t__AM1
d__Bacteria.p__Proteobacteria.c__Alphaproteobacteria.o__Rhizobiales.f__Methylobacteriaceae.g__Methylobacterium.s__chloromethanicum.t__CM4
d__Bacteria.p__Proteobacteria.c__Alphaproteobacteria.o__Rhizobiales.f__Methylobacteriaceae.g__Methylobacterium.s__extorquens.t__DM4
d__Bacteria.p__Proteobacteria.c__Alphaproteobacteria.o__Rhizobiales.f__Methylobacteriaceae.g__Methylobacterium.s__extorquens.t__PA1
d__Bacteria.p__Proteobacteria.c__Alphaproteobacteria.o__Rhizobiales.f__Methylocystaceae.g__Methylocystis.s__sp._t__ATCC_49242
d__Bacteria.p__Proteobacteria.c__Alphaproteobacteria.o__Rhizobiales.f__Methylocystaceae.g__Methylosinus.s__trichosporium.t__OB3b
d__Bacteria.p__Proteobacteria.c__Alphaproteobacteria.o__Rhizobiales.f__Beijerinckiaceae.g__Methylocella.s__silvestris.t__BL2
d__Bacteria.p__Proteobacteria.c__Alphaproteobacteria.o__Rhizobiales.f__Beijerinckiaceae.g__Beijerinckia.s__indica.t__subsp__indica_ATCC_9039
bin__58_10132015
bin__262_10132015

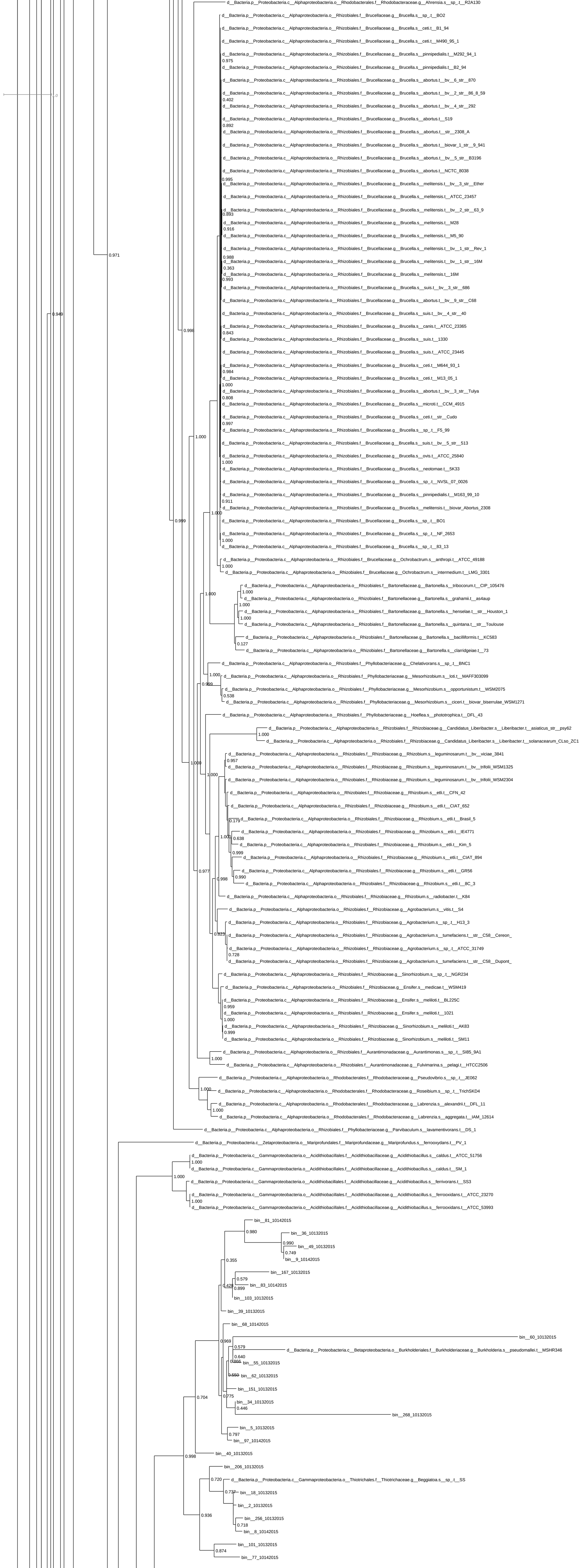

d__Bacteria.p__Proteobacteria.c__Alphaproteobacteria.o__Rhodobacterales.f__Rhodobacteraceae.g__Ahrensia.s__sp_.t__R2A130
d__Bacteria.p__Proteobacteria.c__Alphaproteobacteria.o__Rhizobiales.f__Brucellaceae.g__Brucella.s__sp_.t__BO2
d__Bacteria.p__Proteobacteria.c__Alphaproteobacteria.o__Rhizobiales.f__Brucellaceae.g__Brucella.s__ceti.t__B1_94
d__Bacteria.p__Proteobacteria.c__Alphaproteobacteria.o__Rhizobiales.f__Brucellaceae.g__Brucella.s__ceti.t__M490_95_1
d__Bacteria.p__Proteobacteria.c__Alphaproteobacteria.o__Rhizobiales.f__Brucellaceae.g__Brucella.s__pinnipedialis.t__M292_94_1
0.975
d__Bacteria.p__Proteobacteria.c__Alphaproteobacteria.o__Rhizobiales.f__Brucellaceae.g__Brucella.s__pinnipedialis.t__B2_94
d__Bacteria.p__Proteobacteria.c__Alphaproteobacteria.o__Rhizobiales.f__Brucellaceae.g__Brucella.s__abortus.t__bv__6_str__870
d__Bacteria.p__Proteobacteria.c__Alphaproteobacteria.o__Rhizobiales.f__Brucellaceae.g__Brucella.s__abortus.t__bv__2_str__86_8_59
0.402
d__Bacteria.p__Proteobacteria.c__Alphaproteobacteria.o__Rhizobiales.f__Brucellaceae.g__Brucella.s__abortus.t__bv__4_str__292
d__Bacteria.p__Proteobacteria.c__Alphaproteobacteria.o__Rhizobiales.f__Brucellaceae.g__Brucella.s__abortus.t__S19
0.892
d__Bacteria.p__Proteobacteria.c__Alphaproteobacteria.o__Rhizobiales.f__Brucellaceae.g__Brucella.s__abortus.t__str__2308_A
d__Bacteria.p__Proteobacteria.c__Alphaproteobacteria.o__Rhizobiales.f__Brucellaceae.g__Brucella.s__abortus.t__biovar_1_str__9_941
d__Bacteria.p__Proteobacteria.c__Alphaproteobacteria.o__Rhizobiales.f__Brucellaceae.g__Brucella.s__abortus.t__bv__5_str__B3196
d__Bacteria.p__Proteobacteria.c__Alphaproteobacteria.o__Rhizobiales.f__Brucellaceae.g__Brucella.s__abortus.t__NCTC_8038
0.995
d__Bacteria.p__Proteobacteria.c__Alphaproteobacteria.o__Rhizobiales.f__Brucellaceae.g__Brucella.s__melitensis.t__bv__3_str__Ether
d__Bacteria.p__Proteobacteria.c__Alphaproteobacteria.o__Rhizobiales.f__Brucellaceae.g__Brucella.s__melitensis.t__ATCC_23457
d__Bacteria.p__Proteobacteria.c__Alphaproteobacteria.o__Rhizobiales.f__Brucellaceae.g__Brucella.s__melitensis.t__bv__2_str__63_9
0.893
d__Bacteria.p__Proteobacteria.c__Alphaproteobacteria.o__Rhizobiales.f__Brucellaceae.g__Brucella.s__melitensis.t__M28
0.916
d__Bacteria.p__Proteobacteria.c__Alphaproteobacteria.o__Rhizobiales.f__Brucellaceae.g__Brucella.s__melitensis.t__M5_90
d__Bacteria.p__Proteobacteria.c__Alphaproteobacteria.o__Rhizobiales.f__Brucellaceae.g__Brucella.s__melitensis.t__bv__1_str__Rev_1
0.971
0.988
d__Bacteria.p__Proteobacteria.c__Alphaproteobacteria.o__Rhizobiales.f__Brucellaceae.g__Brucella.s__melitensis.t__bv__1_str__16M
0.363
d__Bacteria.p__Proteobacteria.c__Alphaproteobacteria.o__Rhizobiales.f__Brucellaceae.g__Brucella.s__melitensis.t__16M
0.993
d__Bacteria.p__Proteobacteria.c__Alphaproteobacteria.o__Rhizobiales.f__Brucellaceae.g__Brucella.s__suis.t__bv__3_str__686
d__Bacteria.p__Proteobacteria.c__Alphaproteobacteria.o__Rhizobiales.f__Brucellaceae.g__Brucella.s__abortus.t__bv__9_str__C68
0.949
d__Bacteria.p__Proteobacteria.c__Alphaproteobacteria.o__Rhizobiales.f__Brucellaceae.g__Brucella.s__suis.t__bv__4_str__40
d__Bacteria.p__Proteobacteria.c__Alphaproteobacteria.o__Rhizobiales.f__Brucellaceae.g__Brucella.s__canis.t__ATCC_23365
0.998
0.843
d__Bacteria.p__Proteobacteria.c__Alphaproteobacteria.o__Rhizobiales.f__Brucellaceae.g__Brucella.s__suis.t__1330
d__Bacteria.p__Proteobacteria.c__Alphaproteobacteria.o__Rhizobiales.f__Brucellaceae.g__Brucella.s__suis.t__ATCC_23445
d__Bacteria.p__Proteobacteria.c__Alphaproteobacteria.o__Rhizobiales.f__Brucellaceae.g__Brucella.s__ceti.t__M644_93_1
0.984
d__Bacteria.p__Proteobacteria.c__Alphaproteobacteria.o__Rhizobiales.f__Brucellaceae.g__Brucella.s__ceti.t__M13_05_1
1.000
d__Bacteria.p__Proteobacteria.c__Alphaproteobacteria.o__Rhizobiales.f__Brucellaceae.g__Brucella.s__abortus.t__bv__3_str__Tulya
0.808
d__Bacteria.p__Proteobacteria.c__Alphaproteobacteria.o__Rhizobiales.f__Brucellaceae.g__Brucella.s__microti.t__CCM_4915
d__Bacteria.p__Proteobacteria.c__Alphaproteobacteria.o__Rhizobiales.f__Brucellaceae.g__Brucella.s__ceti.t__str__Cudo
0.997
d__Bacteria.p__Proteobacteria.c__Alphaproteobacteria.o__Rhizobiales.f__Brucellaceae.g__Brucella.s__sp_.t__F5_99
1.000
d__Bacteria.p__Proteobacteria.c__Alphaproteobacteria.o__Rhizobiales.f__Brucellaceae.g__Brucella.s__suis.t__bv__5_str__513
d__Bacteria.p__Proteobacteria.c__Alphaproteobacteria.o__Rhizobiales.f__Brucellaceae.g__Brucella.s__ovis.t__ATCC_25840
1.000
d__Bacteria.p__Proteobacteria.c__Alphaproteobacteria.o__Rhizobiales.f__Brucellaceae.g__Brucella.s__neotomae.t__5K33
d__Bacteria.p__Proteobacteria.c__Alphaproteobacteria.o__Rhizobiales.f__Brucellaceae.g__Brucella.s__sp_.t__NVSL_07_0026
d__Bacteria.p__Proteobacteria.c__Alphaproteobacteria.o__Rhizobiales.f__Brucellaceae.g__Brucella.s__pinnipedialis.t__M163_99_10
0.911
d__Bacteria.p__Proteobacteria.c__Alphaproteobacteria.o__Rhizobiales.f__Brucellaceae.g__Brucella.s__melitensis.t__biovar_Abortus_2308
1.000
0.999
d__Bacteria.p__Proteobacteria.c__Alphaproteobacteria.o__Rhizobiales.f__Brucellaceae.g__Brucella.s__sp_.t__BO1
d__Bacteria.p__Proteobacteria.c__Alphaproteobacteria.o__Rhizobiales.f__Brucellaceae.g__Brucella.s__sp_.t__NF_2653
1.000
d__Bacteria.p__Proteobacteria.c__Alphaproteobacteria.o__Rhizobiales.f__Brucellaceae.g__Brucella.s__sp_.t__83_13
d__Bacteria.p__Proteobacteria.c__Alphaproteobacteria.o__Rhizobiales.f__Brucellaceae.g__Ochrobactrum.s__anthropi.t__ATCC_49188
1.000
d__Bacteria.p__Proteobacteria.c__Alphaproteobacteria.o__Rhizobiales.f__Brucellaceae.g__Ochrobactrum.s__intermedium.t__LMG_3301
d__Bacteria.p__Proteobacteria.c__Alphaproteobacteria.o__Rhizobiales.f__Bartonellaceae.g__Bartonella.s__tribocorum.t__CIP_105476
1.000
1.000
d__Bacteria.p__Proteobacteria.c__Alphaproteobacteria.o__Rhizobiales.f__Bartonellaceae.g__Bartonella.s__grahamii.t__as4aup
1.000
d__Bacteria.p__Proteobacteria.c__Alphaproteobacteria.o__Rhizobiales.f__Bartonellaceae.g__Bartonella.s__henselae.t__str__Houston_1
1.000
d__Bacteria.p__Proteobacteria.c__Alphaproteobacteria.o__Rhizobiales.f__Bartonellaceae.g__Bartonella.s__quintana.t__str__Toulouse
d__Bacteria.p__Proteobacteria.c__Alphaproteobacteria.o__Rhizobiales.f__Bartonellaceae.g__Bartonella.s__bacilliformis.t__KC583
0.127
d__Bacteria.p__Proteobacteria.c__Alphaproteobacteria.o__Rhizobiales.f__Bartonellaceae.g__Bartonella.s__clarridgeiae.t__73
d__Bacteria.p__Proteobacteria.c__Alphaproteobacteria.o__Rhizobiales.f__Phyllobacteriaceae.g__Chelativorans.s__sp_.t__BNC1
1.000
d__Bacteria.p__Proteobacteria.c__Alphaproteobacteria.o__Rhizobiales.f__Phyllobacteriaceae.g__Mesorhizobium.s__loti.t__MAFF303099
0.995
d__Bacteria.p__Proteobacteria.c__Alphaproteobacteria.o__Rhizobiales.f__Phyllobacteriaceae.g__Mesorhizobium.s__opportunistum.t__WSM2075
0.538
d__Bacteria.p__Proteobacteria.c__Alphaproteobacteria.o__Rhizobiales.f__Phyllobacteriaceae.g__Mesorhizobium.s__ciceri.t__biovar_biserrulae_WSM1271
d__Bacteria.p__Proteobacteria.c__Alphaproteobacteria.o__Rhizobiales.f__Phyllobacteriaceae.g__Hoeflea.s__phototrophica.t__DFL_43
d__Bacteria.p__Proteobacteria.c__Alphaproteobacteria.o__Rhizobiales.f__Rhizobiaceae.g__Candidatus_Liberibacter.s__Liberibacter.t__asiaticus_str__psy62
1.000
d__Bacteria.p__Proteobacteria.c__Alphaproteobacteria.o__Rhizobiales.f__Rhizobiaceae.g__Candidatus_Liberibacter.s__Liberibacter.t__solanacearum_CLso_ZC1
d__Bacteria.p__Proteobacteria.c__Alphaproteobacteria.o__Rhizobiales.f__Rhizobiaceae.g__Rhizobium.s__leguminosarum.t__bv__viciae_3841
0.957
1.000
d__Bacteria.p__Proteobacteria.c__Alphaproteobacteria.o__Rhizobiales.f__Rhizobiaceae.g__Rhizobium.s__leguminosarum.t__bv__trifolii_WSM1325
1.000
d__Bacteria.p__Proteobacteria.c__Alphaproteobacteria.o__Rhizobiales.f__Rhizobiaceae.g__Rhizobium.s__leguminosarum.t__bv__trifolii_WSM2304
d__Bacteria.p__Proteobacteria.c__Alphaproteobacteria.o__Rhizobiales.f__Rhizobiaceae.g__Rhizobium.s__etli.t__CFN_42
d__Bacteria.p__Proteobacteria.c__Alphaproteobacteria.o__Rhizobiales.f__Rhizobiaceae.g__Rhizobium.s__etli.t__CIAT_652
0.179
d__Bacteria.p__Proteobacteria.c__Alphaproteobacteria.o__Rhizobiales.f__Rhizobiaceae.g__Rhizobium.s__etli.t__Brasil_5
d__Bacteria.p__Proteobacteria.c__Alphaproteobacteria.o__Rhizobiales.f__Rhizobiaceae.g__Rhizobium.s__etli.t__IE4771
1.000
0.638
d__Bacteria.p__Proteobacteria.c__Alphaproteobacteria.o__Rhizobiales.f__Rhizobiaceae.g__Rhizobium.s__etli.t__Kim_5
0.999
d__Bacteria.p__Proteobacteria.c__Alphaproteobacteria.o__Rhizobiales.f__Rhizobiaceae.g__Rhizobium.s__etli.t__CIAT_894
0.977
d__Bacteria.p__Proteobacteria.c__Alphaproteobacteria.o__Rhizobiales.f__Rhizobiaceae.g__Rhizobium.s__etli.t__GR56
0.998
0.990
d__Bacteria.p__Proteobacteria.c__Alphaproteobacteria.o__Rhizobiales.f__Rhizobiaceae.g__Rhizobium.s__etli.t__8C_3
d__Bacteria.p__Proteobacteria.c__Alphaproteobacteria.o__Rhizobiales.f__Rhizobiaceae.g__Rhizobium.s__radiobacter.t__K84
d__Bacteria.p__Proteobacteria.c__Alphaproteobacteria.o__Rhizobiales.f__Rhizobiaceae.g__Agrobacterium.s__vitis.t__S4
d__Bacteria.p__Proteobacteria.c__Alphaproteobacteria.o__Rhizobiales.f__Rhizobiaceae.g__Agrobacterium.s__sp_.t__H13_3
0.823
d__Bacteria.p__Proteobacteria.c__Alphaproteobacteria.o__Rhizobiales.f__Rhizobiaceae.g__Agrobacterium.s__tumefaciens.t__str__C58__Cereon_
d__Bacteria.p__Proteobacteria.c__Alphaproteobacteria.o__Rhizobiales.f__Rhizobiaceae.g__Agrobacterium.s__sp_.t__ATCC_31749
0.728
d__Bacteria.p__Proteobacteria.c__Alphaproteobacteria.o__Rhizobiales.f__Rhizobiaceae.g__Agrobacterium.s__tumefaciens.t__str__C58__Dupont_
d__Bacteria.p__Proteobacteria.c__Alphaproteobacteria.o__Rhizobiales.f__Rhizobiaceae.g__Sinorhizobium.s__sp_.t__NGR234
d__Bacteria.p__Proteobacteria.c__Alphaproteobacteria.o__Rhizobiales.f__Rhizobiaceae.g__Ensifer.s__medicae.t__WSM419
d__Bacteria.p__Proteobacteria.c__Alphaproteobacteria.o__Rhizobiales.f__Rhizobiaceae.g__Ensifer.s__meliloti.t__BL225C
0.959
d__Bacteria.p__Proteobacteria.c__Alphaproteobacteria.o__Rhizobiales.f__Rhizobiaceae.g__Ensifer.s__meliloti.t__1021
1.000
d__Bacteria.p__Proteobacteria.c__Alphaproteobacteria.o__Rhizobiales.f__Rhizobiaceae.g__Sinorhizobium.s__meliloti.t__AK83
0.999
d__Bacteria.p__Proteobacteria.c__Alphaproteobacteria.o__Rhizobiales.f__Rhizobiaceae.g__Sinorhizobium.s__meliloti.t__SM11
d__Bacteria.p__Proteobacteria.c__Alphaproteobacteria.o__Rhizobiales.f__Aurantimonadaceae.g__Aurantimonas.s__sp_.t__SI85_9A1
1.000
d__Bacteria.p__Proteobacteria.c__Alphaproteobacteria.o__Rhizobiales.f__Aurantimonadaceae.g__Fulvimarina.s__pelagi.t__HTCC2506
d__Bacteria.p__Proteobacteria.c__Alphaproteobacteria.o__Rhodobacterales.f__Rhodobacteraceae.g__Pseudovibrio.s__sp_.t__JE062
1.000
d__Bacteria.p__Proteobacteria.c__Alphaproteobacteria.o__Rhodobacterales.f__Rhodobacteraceae.g__Roseibium.s__sp_.t__TrichSKD4
d__Bacteria.p__Proteobacteria.c__Alphaproteobacteria.o__Rhodobacterales.f__Rhodobacteraceae.g__Labrenzia.s__alexandrii.t__DFL_11
1.000
d__Bacteria.p__Proteobacteria.c__Alphaproteobacteria.o__Rhodobacterales.f__Rhodobacteraceae.g__Labrenzia.s__aggregata.t__IAM_12614
d__Bacteria.p__Proteobacteria.c__Alphaproteobacteria.o__Rhizobiales.f__Phyllobacteriaceae.g__Parvibaculum.s__lavamentivorans.t__DS_1
d__Bacteria.p__Proteobacteria.c__Zetaproteobacteria.o__Mariprofundales.f__Mariprofundaceae.g__Mariprofundus.s__ferrooxydans.t__PV_1
d__Bacteria.p__Proteobacteria.c__Gammaproteobacteria.o__Acidithiobacillales.f__Acidithiobacillaceae.g__Acidithiobacillus.s__caldus.t__ATCC_51756
1.000
d__Bacteria.p__Proteobacteria.c__Gammaproteobacteria.o__Acidithiobacillales.f__Acidithiobacillaceae.g__Acidithiobacillus.s__caldus.t__SM_1
1.000
d__Bacteria.p__Proteobacteria.c__Gammaproteobacteria.o__Acidithiobacillales.f__Acidithiobacillaceae.g__Acidithiobacillus.s__ferrivorans.t__SS3
d__Bacteria.p__Proteobacteria.c__Gammaproteobacteria.o__Acidithiobacillales.f__Acidithiobacillaceae.g__Acidithiobacillus.s__ferrooxidans.t__ATCC_23270
1.000
d__Bacteria.p__Proteobacteria.c__Gammaproteobacteria.o__Acidithiobacillales.f__Acidithiobacillaceae.g__Acidithiobacillus.s__ferrooxidans.t__ATCC_53993
bin__81_10142015
0.980
bin__36_10132015
0.990
bin__49_10132015
0.749
bin__9_10142015
0.355
bin__167_10132015
0.579
0.428
bin__83_10142015
0.899
bin__103_10132015
bin__39_10132015
bin__68_10142015
bin__60_10132015
0.969
0.579
d__Bacteria.p__Proteobacteria.c__Betaproteobacteria.o__Burkholderiales.f__Burkholderiaceae.g__Burkholderia.s__pseudomallei.t__MSHR346
0.640
0.866
bin__55_10132015
0.550
bin__62_10132015
bin__151_10132015
0.704
0.775
bin__34_10132015
0.446
bin__268_10132015
bin__5_10132015
0.797
bin__97_10142015
bin__40_10132015
0.998
bin__206_10132015
0.720
d__Bacteria.p__Proteobacteria.c__Gammaproteobacteria.o__Thiotrichales.f__Thiotrichaceae.g__Beggiatoa.s__sp_.t__SS
0.737
bin__18_10132015
bin__2_10132015
0.936
bin__256_10132015
0.718
bin__8_10142015
bin__101_10132015
0.874
bin__77_10142015

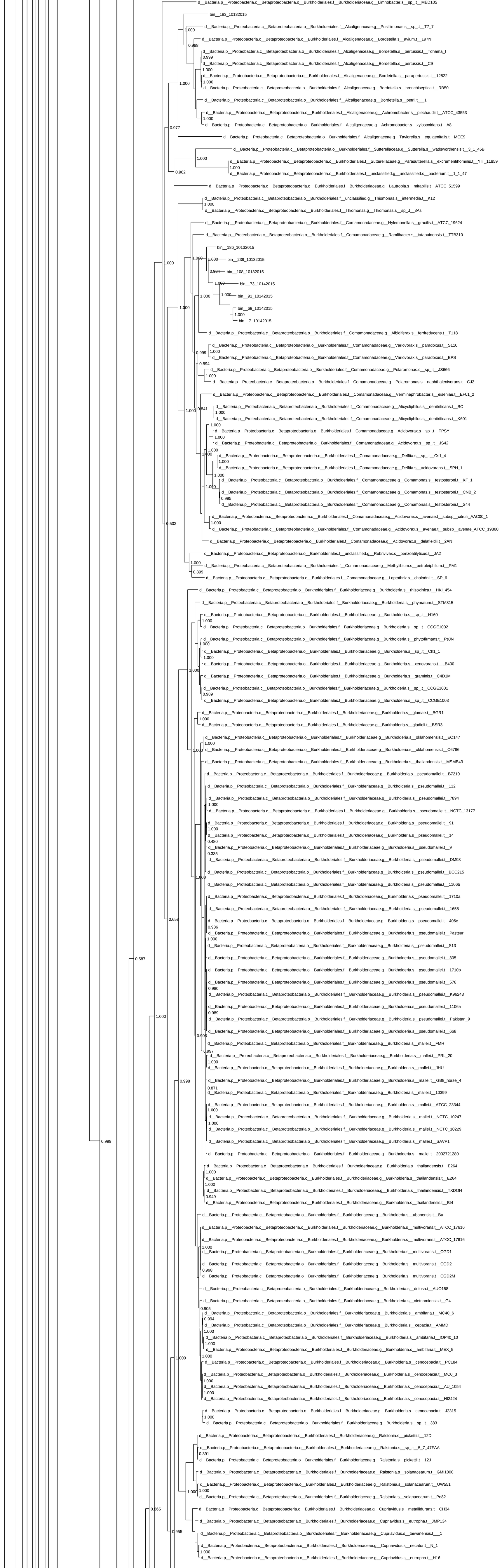

d__Bacteria.p__Proteobacteria.c__Betaproteobacteria.o__Burkholderiales.f__Burkholderiaceae.g__Limnobacter.s__sp_.t__MED105
bin__183_10132015
d__Bacteria.p__Proteobacteria.c__Betaproteobacteria.o__Burkholderiales.f__Alcaligenaceae.g__Pusillimonas.s__sp_.t__T7_7
d__Bacteria.p__Proteobacteria.c__Betaproteobacteria.o__Burkholderiales.f__Alcaligenaceae.g__Bordetella.s__avium.t__197N
d__Bacteria.p__Proteobacteria.c__Betaproteobacteria.o__Burkholderiales.f__Alcaligenaceae.g__Bordetella.s__pertussis.t__Tohama_I
d__Bacteria.p__Proteobacteria.c__Betaproteobacteria.o__Burkholderiales.f__Alcaligenaceae.g__Bordetella.s__pertussis.t__CS
d__Bacteria.p__Proteobacteria.c__Betaproteobacteria.o__Burkholderiales.f__Alcaligenaceae.g__Bordetella.s__parapertussis.t__12822
d__Bacteria.p__Proteobacteria.c__Betaproteobacteria.o__Burkholderiales.f__Alcaligenaceae.g__Bordetella.s__bronchiseptica.t__RB50
d__Bacteria.p__Proteobacteria.c__Betaproteobacteria.o__Burkholderiales.f__Alcaligenaceae.g__Bordetella.s__petrii.t___1
d__Bacteria.p__Proteobacteria.c__Betaproteobacteria.o__Burkholderiales.f__Alcaligenaceae.g__Achromobacter.s__piechaudii.t__ATCC_43553
d__Bacteria.p__Proteobacteria.c__Betaproteobacteria.o__Burkholderiales.f__Alcaligenaceae.g__Achromobacter.s__xylosoxidans.t__A8
d__Bacteria.p__Proteobacteria.c__Betaproteobacteria.o__Burkholderiales.f__Alcaligenaceae.g__Taylorella.s__equigenitalis.t__MCE9
d__Bacteria.p__Proteobacteria.c__Betaproteobacteria.o__Burkholderiales.f__Sutterellaceae.g__Sutterella.s__wadsworthensis.t__3_1_45B
d__Bacteria.p__Proteobacteria.c__Betaproteobacteria.o__Burkholderiales.f__Sutterellaceae.g__Parasutterella.s__excrementihominis.t__YIT_11859
d__Bacteria.p__Proteobacteria.c__Betaproteobacteria.o__Burkholderiales.f__unclassified.g__unclassified.s__bacterium.t__1_1_47
d__Bacteria.p__Proteobacteria.c__Betaproteobacteria.o__Burkholderiales.f__Burkholderiaceae.g__Lautropia.s__mirabilis.t__ATCC_51599
d__Bacteria.p__Proteobacteria.c__Betaproteobacteria.o__Burkholderiales.f__unclassified.g__Thiomonas.s__intermedia.t__K12
d__Bacteria.p__Proteobacteria.c__Betaproteobacteria.o__Burkholderiales.f__Thiomonas.g__Thiomonas.s__sp_.t__3As
d__Bacteria.p__Proteobacteria.c__Betaproteobacteria.o__Burkholderiales.f__Comamonadaceae.g__Hylemonella.s__gracilis.t__ATCC_19624
d__Bacteria.p__Proteobacteria.c__Betaproteobacteria.o__Burkholderiales.f__Comamonadaceae.g__Ramlibacter.s__tataouinensis.t__TTB310
bin__186_10132015
bin__239_10132015
bin__108_10132015
bin__73_10142015
bin__91_10142015
bin__69_10142015
bin__7_10142015
d__Bacteria.p__Proteobacteria.c__Betaproteobacteria.o__Burkholderiales.f__Comamonadaceae.g__Albidiferax.s__ferrireducens.t__T118
d__Bacteria.p__Proteobacteria.c__Betaproteobacteria.o__Burkholderiales.f__Comamonadaceae.g__Variovorax.s__paradoxus.t__S110
d__Bacteria.p__Proteobacteria.c__Betaproteobacteria.o__Burkholderiales.f__Comamonadaceae.g__Variovorax.s__paradoxus.t__EPS
d__Bacteria.p__Proteobacteria.c__Betaproteobacteria.o__Burkholderiales.f__Comamonadaceae.g__Polaromonas.s__sp_.t__JS666
d__Bacteria.p__Proteobacteria.c__Betaproteobacteria.o__Burkholderiales.f__Comamonadaceae.g__Polaromonas.s__naphthalenivorans.t__CJ2
d__Bacteria.p__Proteobacteria.c__Betaproteobacteria.o__Burkholderiales.f__Comamonadaceae.g__Verminephrobacter.s__eiseniae.t__EF01_2
d__Bacteria.p__Proteobacteria.c__Betaproteobacteria.o__Burkholderiales.f__Comamonadaceae.g__Alicycliphilus.s__denitrificans.t__BC
d__Bacteria.p__Proteobacteria.c__Betaproteobacteria.o__Burkholderiales.f__Comamonadaceae.g__Alicycliphilus.s__denitrificans.t__K601
d__Bacteria.p__Proteobacteria.c__Betaproteobacteria.o__Burkholderiales.f__Comamonadaceae.g__Acidovorax.s__sp_.t__TPSY
d__Bacteria.p__Proteobacteria.c__Betaproteobacteria.o__Burkholderiales.f__Comamonadaceae.g__Acidovorax.s__sp_.t__JS42
d__Bacteria.p__Proteobacteria.c__Betaproteobacteria.o__Burkholderiales.f__Comamonadaceae.g__Delftia.s__sp_.t__Cs1_4
d__Bacteria.p__Proteobacteria.c__Betaproteobacteria.o__Burkholderiales.f__Comamonadaceae.g__Delftia.s__acidovorans.t__SPH_1
d__Bacteria.p__Proteobacteria.c__Betaproteobacteria.o__Burkholderiales.f__Comamonadaceae.g__Comamonas.s__testosteroni.t__KF_1
d__Bacteria.p__Proteobacteria.c__Betaproteobacteria.o__Burkholderiales.f__Comamonadaceae.g__Comamonas.s__testosteroni.t__CNB_2
d__Bacteria.p__Proteobacteria.c__Betaproteobacteria.o__Burkholderiales.f__Comamonadaceae.g__Comamonas.s__testosteroni.t__S44
d__Bacteria.p__Proteobacteria.c__Betaproteobacteria.o__Burkholderiales.f__Comamonadaceae.g__Acidovorax.s__avenae.t__subsp__citrulli_AAC00_1
d__Bacteria.p__Proteobacteria.c__Betaproteobacteria.o__Burkholderiales.f__Comamonadaceae.g__Acidovorax.s__avenae.t__subsp__avenae_ATCC_19860
d__Bacteria.p__Proteobacteria.c__Betaproteobacteria.o__Burkholderiales.f__Comamonadaceae.g__Acidovorax.s__delafieldii.t__2AN
d__Bacteria.p__Proteobacteria.c__Betaproteobacteria.o__Burkholderiales.f__unclassified.g__Rubrivivax.s__benzoatilyticus.t__JA2
d__Bacteria.p__Proteobacteria.c__Betaproteobacteria.o__Burkholderiales.f__Comamonadaceae.g__Methylibium.s__petroleiphilum.t__PM1
d__Bacteria.p__Proteobacteria.c__Betaproteobacteria.o__Burkholderiales.f__Comamonadaceae.g__Leptothrix.s__cholodnii.t__SP_6
d__Bacteria.p__Proteobacteria.c__Betaproteobacteria.o__Burkholderiales.f__Burkholderiaceae.g__Burkholderia.s__rhizoxinica.t__HKI_454
d__Bacteria.p__Proteobacteria.c__Betaproteobacteria.o__Burkholderiales.f__Burkholderiaceae.g__Burkholderia.s__phymatum.t__STM815
d__Bacteria.p__Proteobacteria.c__Betaproteobacteria.o__Burkholderiales.f__Burkholderiaceae.g__Burkholderia.s__sp_.t__H160
d__Bacteria.p__Proteobacteria.c__Betaproteobacteria.o__Burkholderiales.f__Burkholderiaceae.g__Burkholderia.s__sp_.t__CCGE1002
d__Bacteria.p__Proteobacteria.c__Betaproteobacteria.o__Burkholderiales.f__Burkholderiaceae.g__Burkholderia.s__phytofirmans.t__PsJN
d__Bacteria.p__Proteobacteria.c__Betaproteobacteria.o__Burkholderiales.f__Burkholderiaceae.g__Burkholderia.s__sp_.t__Ch1_1
d__Bacteria.p__Proteobacteria.c__Betaproteobacteria.o__Burkholderiales.f__Burkholderiaceae.g__Burkholderia.s__xenovorans.t__LB400
d__Bacteria.p__Proteobacteria.c__Betaproteobacteria.o__Burkholderiales.f__Burkholderiaceae.g__Burkholderia.s__graminis.t__C4D1M
d__Bacteria.p__Proteobacteria.c__Betaproteobacteria.o__Burkholderiales.f__Burkholderiaceae.g__Burkholderia.s__sp_.t__CCGE1001
d__Bacteria.p__Proteobacteria.c__Betaproteobacteria.o__Burkholderiales.f__Burkholderiaceae.g__Burkholderia.s__sp_.t__CCGE1003
d__Bacteria.p__Proteobacteria.c__Betaproteobacteria.o__Burkholderiales.f__Burkholderiaceae.g__Burkholderia.s__glumae.t__BGR1
d__Bacteria.p__Proteobacteria.c__Betaproteobacteria.o__Burkholderiales.f__Burkholderiaceae.g__Burkholderia.s__gladioli.t__BSR3
d__Bacteria.p__Proteobacteria.c__Betaproteobacteria.o__Burkholderiales.f__Burkholderiaceae.g__Burkholderia.s__oklahomensis.t__EO147
d__Bacteria.p__Proteobacteria.c__Betaproteobacteria.o__Burkholderiales.f__Burkholderiaceae.g__Burkholderia.s__oklahomensis.t__C6786
d__Bacteria.p__Proteobacteria.c__Betaproteobacteria.o__Burkholderiales.f__Burkholderiaceae.g__Burkholderia.s__thailandensis.t__MSMB43
d__Bacteria.p__Proteobacteria.c__Betaproteobacteria.o__Burkholderiales.f__Burkholderiaceae.g__Burkholderia.s__pseudomallei.t__B7210
d__Bacteria.p__Proteobacteria.c__Betaproteobacteria.o__Burkholderiales.f__Burkholderiaceae.g__Burkholderia.s__pseudomallei.t__112
d__Bacteria.p__Proteobacteria.c__Betaproteobacteria.o__Burkholderiales.f__Burkholderiaceae.g__Burkholderia.s__pseudomallei.t__7894
d__Bacteria.p__Proteobacteria.c__Betaproteobacteria.o__Burkholderiales.f__Burkholderiaceae.g__Burkholderia.s__pseudomallei.t__NCTC_13177
d__Bacteria.p__Proteobacteria.c__Betaproteobacteria.o__Burkholderiales.f__Burkholderiaceae.g__Burkholderia.s__pseudomallei.t__91
d__Bacteria.p__Proteobacteria.c__Betaproteobacteria.o__Burkholderiales.f__Burkholderiaceae.g__Burkholderia.s__pseudomallei.t__14
d__Bacteria.p__Proteobacteria.c__Betaproteobacteria.o__Burkholderiales.f__Burkholderiaceae.g__Burkholderia.s__pseudomallei.t__9
d__Bacteria.p__Proteobacteria.c__Betaproteobacteria.o__Burkholderiales.f__Burkholderiaceae.g__Burkholderia.s__pseudomallei.t__DM98
d__Bacteria.p__Proteobacteria.c__Betaproteobacteria.o__Burkholderiales.f__Burkholderiaceae.g__Burkholderia.s__pseudomallei.t__BCC215
d__Bacteria.p__Proteobacteria.c__Betaproteobacteria.o__Burkholderiales.f__Burkholderiaceae.g__Burkholderia.s__pseudomallei.t__1106b
d__Bacteria.p__Proteobacteria.c__Betaproteobacteria.o__Burkholderiales.f__Burkholderiaceae.g__Burkholderia.s__pseudomallei.t__1710a
d__Bacteria.p__Proteobacteria.c__Betaproteobacteria.o__Burkholderiales.f__Burkholderiaceae.g__Burkholderia.s__pseudomallei.t__1655
d__Bacteria.p__Proteobacteria.c__Betaproteobacteria.o__Burkholderiales.f__Burkholderiaceae.g__Burkholderia.s__pseudomallei.t__406e
d__Bacteria.p__Proteobacteria.c__Betaproteobacteria.o__Burkholderiales.f__Burkholderiaceae.g__Burkholderia.s__pseudomallei.t__Pasteur
d__Bacteria.p__Proteobacteria.c__Betaproteobacteria.o__Burkholderiales.f__Burkholderiaceae.g__Burkholderia.s__pseudomallei.t__S13
d__Bacteria.p__Proteobacteria.c__Betaproteobacteria.o__Burkholderiales.f__Burkholderiaceae.g__Burkholderia.s__pseudomallei.t__305
d__Bacteria.p__Proteobacteria.c__Betaproteobacteria.o__Burkholderiales.f__Burkholderiaceae.g__Burkholderia.s__pseudomallei.t__1710b
d__Bacteria.p__Proteobacteria.c__Betaproteobacteria.o__Burkholderiales.f__Burkholderiaceae.g__Burkholderia.s__pseudomallei.t__576
d__Bacteria.p__Proteobacteria.c__Betaproteobacteria.o__Burkholderiales.f__Burkholderiaceae.g__Burkholderia.s__pseudomallei.t__K96243
d__Bacteria.p__Proteobacteria.c__Betaproteobacteria.o__Burkholderiales.f__Burkholderiaceae.g__Burkholderia.s__pseudomallei.t__1106a
d__Bacteria.p__Proteobacteria.c__Betaproteobacteria.o__Burkholderiales.f__Burkholderiaceae.g__Burkholderia.s__pseudomallei.t__Pakistan_9
d__Bacteria.p__Proteobacteria.c__Betaproteobacteria.o__Burkholderiales.f__Burkholderiaceae.g__Burkholderia.s__pseudomallei.t__668
d__Bacteria.p__Proteobacteria.c__Betaproteobacteria.o__Burkholderiales.f__Burkholderiaceae.g__Burkholderia.s__mallei.t__FMH
d__Bacteria.p__Proteobacteria.c__Betaproteobacteria.o__Burkholderiales.f__Burkholderiaceae.g__Burkholderia.s__mallei.t__PRL_20
d__Bacteria.p__Proteobacteria.c__Betaproteobacteria.o__Burkholderiales.f__Burkholderiaceae.g__Burkholderia.s__mallei.t__JHU
d__Bacteria.p__Proteobacteria.c__Betaproteobacteria.o__Burkholderiales.f__Burkholderiaceae.g__Burkholderia.s__mallei.t__GB8_horse_4
d__Bacteria.p__Proteobacteria.c__Betaproteobacteria.o__Burkholderiales.f__Burkholderiaceae.g__Burkholderia.s__mallei.t__10399
d__Bacteria.p__Proteobacteria.c__Betaproteobacteria.o__Burkholderiales.f__Burkholderiaceae.g__Burkholderia.s__mallei.t__ATCC_23344
d__Bacteria.p__Proteobacteria.c__Betaproteobacteria.o__Burkholderiales.f__Burkholderiaceae.g__Burkholderia.s__mallei.t__NCTC_10247
d__Bacteria.p__Proteobacteria.c__Betaproteobacteria.o__Burkholderiales.f__Burkholderiaceae.g__Burkholderia.s__mallei.t__NCTC_10229
d__Bacteria.p__Proteobacteria.c__Betaproteobacteria.o__Burkholderiales.f__Burkholderiaceae.g__Burkholderia.s__mallei.t__SAVP1
d__Bacteria.p__Proteobacteria.c__Betaproteobacteria.o__Burkholderiales.f__Burkholderiaceae.g__Burkholderia.s__mallei.t__2002721280
d__Bacteria.p__Proteobacteria.c__Betaproteobacteria.o__Burkholderiales.f__Burkholderiaceae.g__Burkholderia.s__thailandensis.t__E264
d__Bacteria.p__Proteobacteria.c__Betaproteobacteria.o__Burkholderiales.f__Burkholderiaceae.g__Burkholderia.s__thailandensis.t__E264
d__Bacteria.p__Proteobacteria.c__Betaproteobacteria.o__Burkholderiales.f__Burkholderiaceae.g__Burkholderia.s__thailandensis.t__TXDOH
d__Bacteria.p__Proteobacteria.c__Betaproteobacteria.o__Burkholderiales.f__Burkholderiaceae.g__Burkholderia.s__thailandensis.t__Bt4
d__Bacteria.p__Proteobacteria.c__Betaproteobacteria.o__Burkholderiales.f__Burkholderiaceae.g__Burkholderia.s__ubonensis.t__Bu
d__Bacteria.p__Proteobacteria.c__Betaproteobacteria.o__Burkholderiales.f__Burkholderiaceae.g__Burkholderia.s__multivorans.t__ATCC_17616
d__Bacteria.p__Proteobacteria.c__Betaproteobacteria.o__Burkholderiales.f__Burkholderiaceae.g__Burkholderia.s__multivorans.t__ATCC_17616
d__Bacteria.p__Proteobacteria.c__Betaproteobacteria.o__Burkholderiales.f__Burkholderiaceae.g__Burkholderia.s__multivorans.t__CGD1
d__Bacteria.p__Proteobacteria.c__Betaproteobacteria.o__Burkholderiales.f__Burkholderiaceae.g__Burkholderia.s__multivorans.t__CGD2
d__Bacteria.p__Proteobacteria.c__Betaproteobacteria.o__Burkholderiales.f__Burkholderiaceae.g__Burkholderia.s__multivorans.t__CGD2M
d__Bacteria.p__Proteobacteria.c__Betaproteobacteria.o__Burkholderiales.f__Burkholderiaceae.g__Burkholderia.s__dolosa.t__AUO158
d__Bacteria.p__Proteobacteria.c__Betaproteobacteria.o__Burkholderiales.f__Burkholderiaceae.g__Burkholderia.s__vietnamiensis.t__G4
d__Bacteria.p__Proteobacteria.c__Betaproteobacteria.o__Burkholderiales.f__Burkholderiaceae.g__Burkholderia.s__ambifaria.t__MC40_6
d__Bacteria.p__Proteobacteria.c__Betaproteobacteria.o__Burkholderiales.f__Burkholderiaceae.g__Burkholderia.s__cepacia.t__AMMD
d__Bacteria.p__Proteobacteria.c__Betaproteobacteria.o__Burkholderiales.f__Burkholderiaceae.g__Burkholderia.s__ambifaria.t__IOP40_10
d__Bacteria.p__Proteobacteria.c__Betaproteobacteria.o__Burkholderiales.f__Burkholderiaceae.g__Burkholderia.s__ambifaria.t__MEX_5
d__Bacteria.p__Proteobacteria.c__Betaproteobacteria.o__Burkholderiales.f__Burkholderiaceae.g__Burkholderia.s__cenocepacia.t__PC184
d__Bacteria.p__Proteobacteria.c__Betaproteobacteria.o__Burkholderiales.f__Burkholderiaceae.g__Burkholderia.s__cenocepacia.t__MC0_3
d__Bacteria.p__Proteobacteria.c__Betaproteobacteria.o__Burkholderiales.f__Burkholderiaceae.g__Burkholderia.s__cenocepacia.t__AU_1054
d__Bacteria.p__Proteobacteria.c__Betaproteobacteria.o__Burkholderiales.f__Burkholderiaceae.g__Burkholderia.s__cenocepacia.t__HI2424
d__Bacteria.p__Proteobacteria.c__Betaproteobacteria.o__Burkholderiales.f__Burkholderiaceae.g__Burkholderia.s__cenocepacia.t__J2315
d__Bacteria.p__Proteobacteria.c__Betaproteobacteria.o__Burkholderiales.f__Burkholderiaceae.g__Burkholderia.s__sp_.t__383
d__Bacteria.p__Proteobacteria.c__Betaproteobacteria.o__Burkholderiales.f__Burkholderiaceae.g__Ralstonia.s__pickettii.t__12D
d__Bacteria.p__Proteobacteria.c__Betaproteobacteria.o__Burkholderiales.f__Burkholderiaceae.g__Ralstonia.s__sp_.t__5_7_47FAA
d__Bacteria.p__Proteobacteria.c__Betaproteobacteria.o__Burkholderiales.f__Burkholderiaceae.g__Ralstonia.s__pickettii.t__12J
d__Bacteria.p__Proteobacteria.c__Betaproteobacteria.o__Burkholderiales.f__Burkholderiaceae.g__Ralstonia.s__solanacearum.t__GMI1000
d__Bacteria.p__Proteobacteria.c__Betaproteobacteria.o__Burkholderiales.f__Burkholderiaceae.g__Ralstonia.s__solanacearum.t__UW551
d__Bacteria.p__Proteobacteria.c__Betaproteobacteria.o__Burkholderiales.f__Burkholderiaceae.g__Ralstonia.s__solanacearum.t__Po82
d__Bacteria.p__Proteobacteria.c__Betaproteobacteria.o__Burkholderiales.f__Burkholderiaceae.g__Cupriavidus.s__metallidurans.t__CH34
d__Bacteria.p__Proteobacteria.c__Betaproteobacteria.o__Burkholderiales.f__Burkholderiaceae.g__Cupriavidus.s__eutropha.t__JMP134
d__Bacteria.p__Proteobacteria.c__Betaproteobacteria.o__Burkholderiales.f__Burkholderiaceae.g__Cupriavidus.s__taiwanensis.t___1
d__Bacteria.p__Proteobacteria.c__Betaproteobacteria.o__Burkholderiales.f__Burkholderiaceae.g__Cupriavidus.s__necator.t__N_1
d__Bacteria.p__Proteobacteria.c__Betaproteobacteria.o__Burkholderiales.f__Burkholderiaceae.g__Cupriavidus.s__eutropha.t__H16

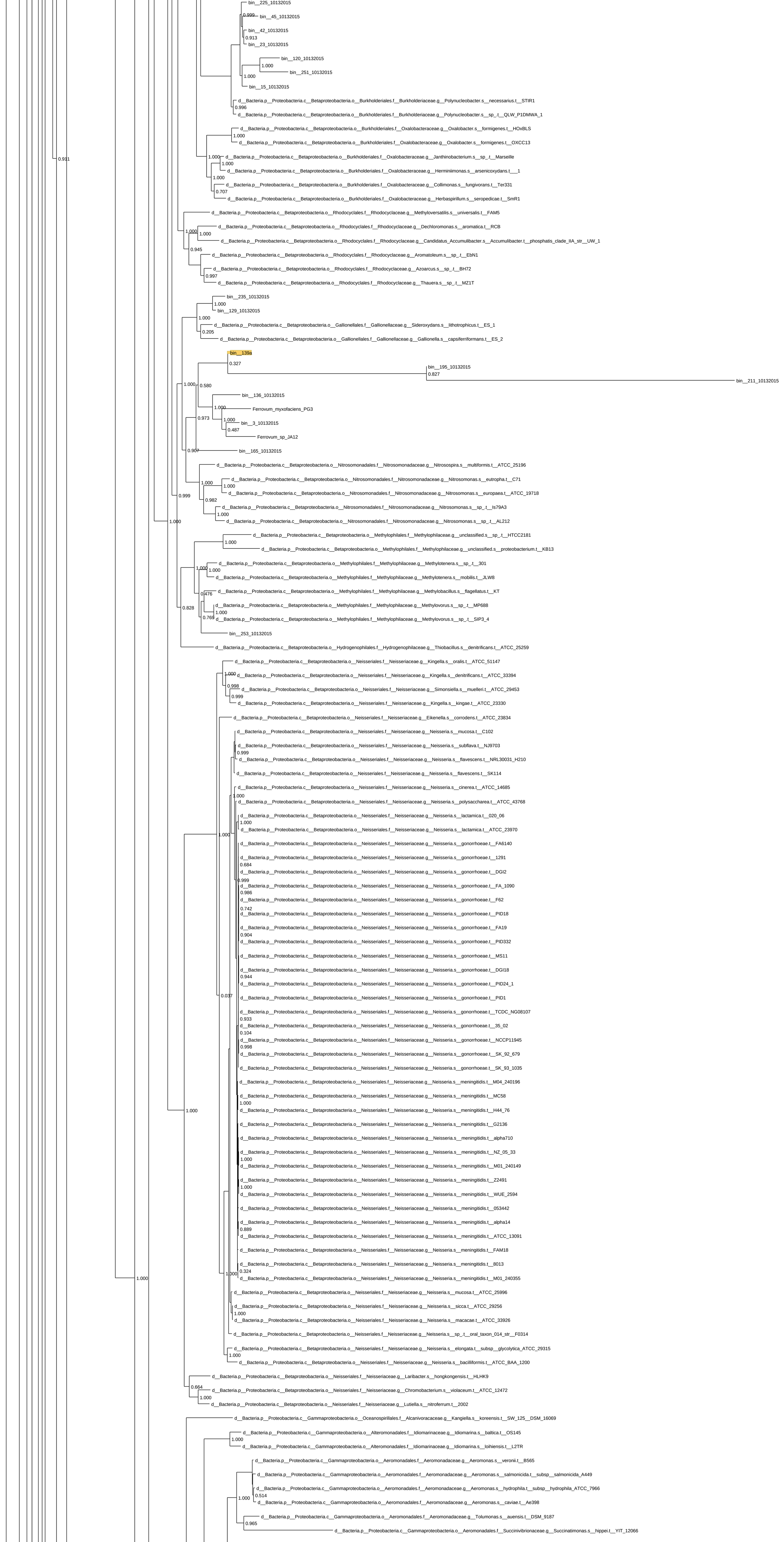
bin__225_10132015
bin__45_10132015
bin__42_10132015
bin__23_10132015
bin__120_10132015
bin__251_10132015
bin__15_10132015
d__Bacteria.p__Proteobacteria.c__Betaproteobacteria.o__Burkholderiales.f__Burkholderiaceae.g__Polynucleobacter.s__necessarius.t__STIR1
d__Bacteria.p__Proteobacteria.c__Betaproteobacteria.o__Burkholderiales.f__Burkholderiaceae.g__Polynucleobacter.s__sp_.t__QLW_P1DMWA_1
d__Bacteria.p__Proteobacteria.c__Betaproteobacteria.o__Burkholderiales.f__Oxalobacteraceae.g__Oxalobacter.s__formigenes.t__HOxBLS
d__Bacteria.p__Proteobacteria.c__Betaproteobacteria.o__Burkholderiales.f__Oxalobacteraceae.g__Oxalobacter.s__formigenes.t__OXCC13
d__Bacteria.p__Proteobacteria.c__Betaproteobacteria.o__Burkholderiales.f__Oxalobacteraceae.g__Janthinobacterium.s__sp_.t__Marseille
d__Bacteria.p__Proteobacteria.c__Betaproteobacteria.o__Burkholderiales.f__Oxalobacteraceae.g__Herminiimonas.s__arsenicoxydans.t___1
d__Bacteria.p__Proteobacteria.c__Betaproteobacteria.o__Burkholderiales.f__Oxalobacteraceae.g__Collimonas.s__fungivorans.t__Ter331
d__Bacteria.p__Proteobacteria.c__Betaproteobacteria.o__Burkholderiales.f__Oxalobacteraceae.g__Herbaspirillum.s__seropedicae.t__SmR1
d__Bacteria.p__Proteobacteria.c__Betaproteobacteria.o__Rhodocyclales.f__Rhodocyclaceae.g__Methyloversatilis.s__universalis.t__FAM5
d__Bacteria.p__Proteobacteria.c__Betaproteobacteria.o__Rhodocyclales.f__Rhodocyclaceae.g__Dechloromonas.s__aromatica.t__RCB
d__Bacteria.p__Proteobacteria.c__Betaproteobacteria.o__Rhodocyclales.f__Rhodocyclaceae.g__Candidatus_Accumulibacter.s__Accumulibacter.t__phosphatis_clade_IIA_str__UW_1
d__Bacteria.p__Proteobacteria.c__Betaproteobacteria.o__Rhodocyclales.f__Rhodocyclaceae.g__Aromatoleum.s__sp_.t__EbN1
d__Bacteria.p__Proteobacteria.c__Betaproteobacteria.o__Rhodocyclales.f__Rhodocyclaceae.g__Azoarcus.s__sp_.t__BH72
d__Bacteria.p__Proteobacteria.c__Betaproteobacteria.o__Rhodocyclales.f__Rhodocyclaceae.g__Thauera.s__sp_.t__MZ1T
bin__235_10132015
bin__129_10132015
d__Bacteria.p__Proteobacteria.c__Betaproteobacteria.o__Gallionellales.f__Gallionellaceae.g__Sideroxydans.s__lithotrophicus.t__ES_1
d__Bacteria.p__Proteobacteria.c__Betaproteobacteria.o__Gallionellales.f__Gallionellaceae.g__Gallionella.s__capsiferriformans.t__ES_2
bin__139a
bin__195_10132015
bin__211_10132015
bin__136_10132015
Ferrovum_myxofaciens_PG3
bin__3_10132015
Ferrovum_sp_JA12
bin__165_10132015
d__Bacteria.p__Proteobacteria.c__Betaproteobacteria.o__Nitrosomonadales.f__Nitrosomonadaceae.g__Nitrosospira.s__multiformis.t__ATCC_25196
d__Bacteria.p__Proteobacteria.c__Betaproteobacteria.o__Nitrosomonadales.f__Nitrosomonadaceae.g__Nitrosomonas.s__eutropha.t__C71
d__Bacteria.p__Proteobacteria.c__Betaproteobacteria.o__Nitrosomonadales.f__Nitrosomonadaceae.g__Nitrosomonas.s__europaea.t__ATCC_19718
d__Bacteria.p__Proteobacteria.c__Betaproteobacteria.o__Nitrosomonadales.f__Nitrosomonadaceae.g__Nitrosomonas.s__sp_.t__Is79A3
d__Bacteria.p__Proteobacteria.c__Betaproteobacteria.o__Nitrosomonadales.f__Nitrosomonadaceae.g__Nitrosomonas.s__sp_.t__AL212
d__Bacteria.p__Proteobacteria.c__Betaproteobacteria.o__Methylophilales.f__Methylophilaceae.g__unclassified.s__sp_.t__HTCC2181
d__Bacteria.p__Proteobacteria.c__Betaproteobacteria.o__Methylophilales.f__Methylophilaceae.g__unclassified.s__proteobacterium.t__KB13
d__Bacteria.p__Proteobacteria.c__Betaproteobacteria.o__Methylophilales.f__Methylophilaceae.g__Methylotenera.s__sp_.t__301
d__Bacteria.p__Proteobacteria.c__Betaproteobacteria.o__Methylophilales.f__Methylophilaceae.g__Methylotenera.s__mobilis.t__JLW8
d__Bacteria.p__Proteobacteria.c__Betaproteobacteria.o__Methylophilales.f__Methylophilaceae.g__Methylobacillus.s__flagellatus.t__KT
d__Bacteria.p__Proteobacteria.c__Betaproteobacteria.o__Methylophilales.f__Methylophilaceae.g__Methylovorus.s__sp_.t__MP688
d__Bacteria.p__Proteobacteria.c__Betaproteobacteria.o__Methylophilales.f__Methylophilaceae.g__Methylovorus.s__sp_.t__SIP3_4
bin__253_10132015
d__Bacteria.p__Proteobacteria.c__Betaproteobacteria.o__Hydrogenophilales.f__Hydrogenophilaceae.g__Thiobacillus.s__denitrificans.t__ATCC_25259
d__Bacteria.p__Proteobacteria.c__Betaproteobacteria.o__Neisseriales.f__Neisseriaceae.g__Kingella.s__oralis.t__ATCC_51147
d__Bacteria.p__Proteobacteria.c__Betaproteobacteria.o__Neisseriales.f__Neisseriaceae.g__Kingella.s__denitrificans.t__ATCC_33394
d__Bacteria.p__Proteobacteria.c__Betaproteobacteria.o__Neisseriales.f__Neisseriaceae.g__Simonsiella.s__muelleri.t__ATCC_29453
d__Bacteria.p__Proteobacteria.c__Betaproteobacteria.o__Neisseriales.f__Neisseriaceae.g__Kingella.s__kingae.t__ATCC_23330
d__Bacteria.p__Proteobacteria.c__Betaproteobacteria.o__Neisseriales.f__Neisseriaceae.g__Eikenella.s__corrodens.t__ATCC_23834
d__Bacteria.p__Proteobacteria.c__Betaproteobacteria.o__Neisseriales.f__Neisseriaceae.g__Neisseria.s__mucosa.t__C102
d__Bacteria.p__Proteobacteria.c__Betaproteobacteria.o__Neisseriales.f__Neisseriaceae.g__Neisseria.s__subflava.t__NJ9703
d__Bacteria.p__Proteobacteria.c__Betaproteobacteria.o__Neisseriales.f__Neisseriaceae.g__Neisseria.s__flavescens.t__NRL30031_H210
d__Bacteria.p__Proteobacteria.c__Betaproteobacteria.o__Neisseriales.f__Neisseriaceae.g__Neisseria.s__flavescens.t__SK114
d__Bacteria.p__Proteobacteria.c__Betaproteobacteria.o__Neisseriales.f__Neisseriaceae.g__Neisseria.s__cinerea.t__ATCC_14685
d__Bacteria.p__Proteobacteria.c__Betaproteobacteria.o__Neisseriales.f__Neisseriaceae.g__Neisseria.s__polysaccharea.t__ATCC_43768
d__Bacteria.p__Proteobacteria.c__Betaproteobacteria.o__Neisseriales.f__Neisseriaceae.g__Neisseria.s__lactamica.t__020_06
d__Bacteria.p__Proteobacteria.c__Betaproteobacteria.o__Neisseriales.f__Neisseriaceae.g__Neisseria.s__lactamica.t__ATCC_23970
d__Bacteria.p__Proteobacteria.c__Betaproteobacteria.o__Neisseriales.f__Neisseriaceae.g__Neisseria.s__gonorrhoeae.t__FA6140
d__Bacteria.p__Proteobacteria.c__Betaproteobacteria.o__Neisseriales.f__Neisseriaceae.g__Neisseria.s__gonorrhoeae.t__1291
d__Bacteria.p__Proteobacteria.c__Betaproteobacteria.o__Neisseriales.f__Neisseriaceae.g__Neisseria.s__gonorrhoeae.t__DGI2
d__Bacteria.p__Proteobacteria.c__Betaproteobacteria.o__Neisseriales.f__Neisseriaceae.g__Neisseria.s__gonorrhoeae.t__FA_1090
d__Bacteria.p__Proteobacteria.c__Betaproteobacteria.o__Neisseriales.f__Neisseriaceae.g__Neisseria.s__gonorrhoeae.t__F62
d__Bacteria.p__Proteobacteria.c__Betaproteobacteria.o__Neisseriales.f__Neisseriaceae.g__Neisseria.s__gonorrhoeae.t__PID18
d__Bacteria.p__Proteobacteria.c__Betaproteobacteria.o__Neisseriales.f__Neisseriaceae.g__Neisseria.s__gonorrhoeae.t__FA19
d__Bacteria.p__Proteobacteria.c__Betaproteobacteria.o__Neisseriales.f__Neisseriaceae.g__Neisseria.s__gonorrhoeae.t__PID332
d__Bacteria.p__Proteobacteria.c__Betaproteobacteria.o__Neisseriales.f__Neisseriaceae.g__Neisseria.s__gonorrhoeae.t__MS11
d__Bacteria.p__Proteobacteria.c__Betaproteobacteria.o__Neisseriales.f__Neisseriaceae.g__Neisseria.s__gonorrhoeae.t__DGI18
d__Bacteria.p__Proteobacteria.c__Betaproteobacteria.o__Neisseriales.f__Neisseriaceae.g__Neisseria.s__gonorrhoeae.t__PID24_1
d__Bacteria.p__Proteobacteria.c__Betaproteobacteria.o__Neisseriales.f__Neisseriaceae.g__Neisseria.s__gonorrhoeae.t__PID1
d__Bacteria.p__Proteobacteria.c__Betaproteobacteria.o__Neisseriales.f__Neisseriaceae.g__Neisseria.s__gonorrhoeae.t__TCDC_NG08107
d__Bacteria.p__Proteobacteria.c__Betaproteobacteria.o__Neisseriales.f__Neisseriaceae.g__Neisseria.s__gonorrhoeae.t__35_02
d__Bacteria.p__Proteobacteria.c__Betaproteobacteria.o__Neisseriales.f__Neisseriaceae.g__Neisseria.s__gonorrhoeae.t__NCCP11945
d__Bacteria.p__Proteobacteria.c__Betaproteobacteria.o__Neisseriales.f__Neisseriaceae.g__Neisseria.s__gonorrhoeae.t__SK_92_679
d__Bacteria.p__Proteobacteria.c__Betaproteobacteria.o__Neisseriales.f__Neisseriaceae.g__Neisseria.s__gonorrhoeae.t__SK_93_1035
d__Bacteria.p__Proteobacteria.c__Betaproteobacteria.o__Neisseriales.f__Neisseriaceae.g__Neisseria.s__meningitidis.t__M04_240196
d__Bacteria.p__Proteobacteria.c__Betaproteobacteria.o__Neisseriales.f__Neisseriaceae.g__Neisseria.s__meningitidis.t__MC58
d__Bacteria.p__Proteobacteria.c__Betaproteobacteria.o__Neisseriales.f__Neisseriaceae.g__Neisseria.s__meningitidis.t__H44_76
d__Bacteria.p__Proteobacteria.c__Betaproteobacteria.o__Neisseriales.f__Neisseriaceae.g__Neisseria.s__meningitidis.t__G2136
d__Bacteria.p__Proteobacteria.c__Betaproteobacteria.o__Neisseriales.f__Neisseriaceae.g__Neisseria.s__meningitidis.t__alpha710
d__Bacteria.p__Proteobacteria.c__Betaproteobacteria.o__Neisseriales.f__Neisseriaceae.g__Neisseria.s__meningitidis.t__NZ_05_33
d__Bacteria.p__Proteobacteria.c__Betaproteobacteria.o__Neisseriales.f__Neisseriaceae.g__Neisseria.s__meningitidis.t__M01_240149
d__Bacteria.p__Proteobacteria.c__Betaproteobacteria.o__Neisseriales.f__Neisseriaceae.g__Neisseria.s__meningitidis.t__Z2491
d__Bacteria.p__Proteobacteria.c__Betaproteobacteria.o__Neisseriales.f__Neisseriaceae.g__Neisseria.s__meningitidis.t__WUE_2594
d__Bacteria.p__Proteobacteria.c__Betaproteobacteria.o__Neisseriales.f__Neisseriaceae.g__Neisseria.s__meningitidis.t__053442
d__Bacteria.p__Proteobacteria.c__Betaproteobacteria.o__Neisseriales.f__Neisseriaceae.g__Neisseria.s__meningitidis.t__alpha14
d__Bacteria.p__Proteobacteria.c__Betaproteobacteria.o__Neisseriales.f__Neisseriaceae.g__Neisseria.s__meningitidis.t__ATCC_13091
d__Bacteria.p__Proteobacteria.c__Betaproteobacteria.o__Neisseriales.f__Neisseriaceae.g__Neisseria.s__meningitidis.t__FAM18
d__Bacteria.p__Proteobacteria.c__Betaproteobacteria.o__Neisseriales.f__Neisseriaceae.g__Neisseria.s__meningitidis.t__8013
d__Bacteria.p__Proteobacteria.c__Betaproteobacteria.o__Neisseriales.f__Neisseriaceae.g__Neisseria.s__meningitidis.t__M01_240355
d__Bacteria.p__Proteobacteria.c__Betaproteobacteria.o__Neisseriales.f__Neisseriaceae.g__Neisseria.s__mucosa.t__ATCC_25996
d__Bacteria.p__Proteobacteria.c__Betaproteobacteria.o__Neisseriales.f__Neisseriaceae.g__Neisseria.s__sicca.t__ATCC_29256
d__Bacteria.p__Proteobacteria.c__Betaproteobacteria.o__Neisseriales.f__Neisseriaceae.g__Neisseria.s__macacae.t__ATCC_33926
d__Bacteria.p__Proteobacteria.c__Betaproteobacteria.o__Neisseriales.f__Neisseriaceae.g__Neisseria.s__sp_.t__oral_taxon_014_str__F0314
d__Bacteria.p__Proteobacteria.c__Betaproteobacteria.o__Neisseriales.f__Neisseriaceae.g__Neisseria.s__elongata.t__subsp__glycolytica_ATCC_29315
d__Bacteria.p__Proteobacteria.c__Betaproteobacteria.o__Neisseriales.f__Neisseriaceae.g__Neisseria.s__bacilliformis.t__ATCC_BAA_1200
d__Bacteria.p__Proteobacteria.c__Betaproteobacteria.o__Neisseriales.f__Neisseriaceae.g__Laribacter.s__hongkongensis.t__HLHK9
d__Bacteria.p__Proteobacteria.c__Betaproteobacteria.o__Neisseriales.f__Neisseriaceae.g__Chromobacterium.s__violaceum.t__ATCC_12472
d__Bacteria.p__Proteobacteria.c__Betaproteobacteria.o__Neisseriales.f__Neisseriaceae.g__Lutiella.s__nitroferrum.t__2002
d__Bacteria.p__Proteobacteria.c__Gammaproteobacteria.o__Oceanospirillales.f__Alcanivoracaceae.g__Kangiella.s__koreensis.t__SW_125__DSM_16069
d__Bacteria.p__Proteobacteria.c__Gammaproteobacteria.o__Alteromonadales.f__Idiomarinaceae.g__Idiomarina.s__baltica.t__OS145
d__Bacteria.p__Proteobacteria.c__Gammaproteobacteria.o__Alteromonadales.f__Idiomarinaceae.g__Idiomarina.s__loihiensis.t__L2TR
d__Bacteria.p__Proteobacteria.c__Gammaproteobacteria.o__Aeromonadales.f__Aeromonadaceae.g__Aeromonas.s__veronii.t__B565
d__Bacteria.p__Proteobacteria.c__Gammaproteobacteria.o__Aeromonadales.f__Aeromonadaceae.g__Aeromonas.s__salmonicida.t__subsp__salmonicida_A449
d__Bacteria.p__Proteobacteria.c__Gammaproteobacteria.o__Aeromonadales.f__Aeromonadaceae.g__Aeromonas.s__hydrophila.t__subsp__hydrophila_ATCC_7966
d__Bacteria.p__Proteobacteria.c__Gammaproteobacteria.o__Aeromonadales.f__Aeromonadaceae.g__Aeromonas.s__caviae.t__Ae398
d__Bacteria.p__Proteobacteria.c__Gammaproteobacteria.o__Aeromonadales.f__Aeromonadaceae.g__Tolumonas.s__auensis.t__DSM_9187
d__Bacteria.p__Proteobacteria.c__Gammaproteobacteria.o__Aeromonadales.f__Succinivibrionaceae.g__Succinatimonas.s__hippei.t__YIT_12066

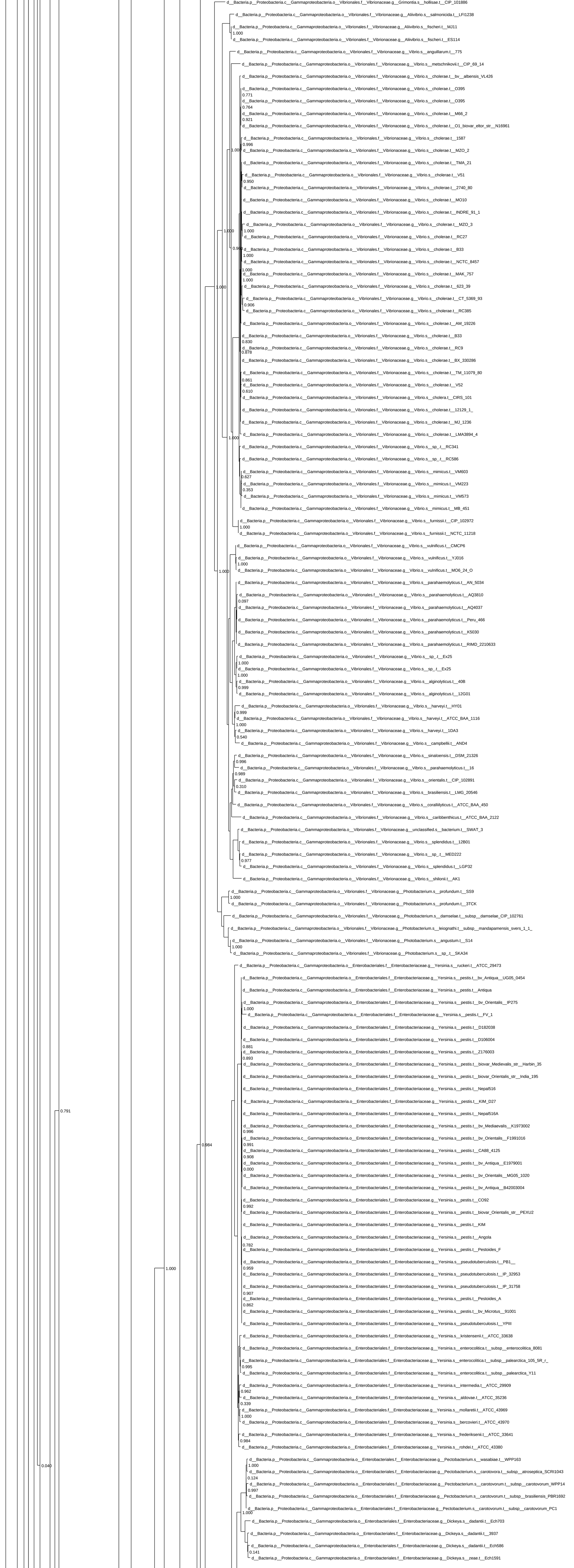
d__Bacteria.p__Proteobacteria.c__Gammaproteobacteria.o__Vibrionales.f__Vibrionaceae.g__Grimontia.s__hollisae.t__CIP_101886
d__Bacteria.p__Proteobacteria.c__Gammaproteobacteria.o__Vibrionales.f__Vibrionaceae.g__Aliivibrio.s__salmonicida.t__LFI1238
d__Bacteria.p__Proteobacteria.c__Gammaproteobacteria.o__Vibrionales.f__Vibrionaceae.g__Aliivibrio.s__fischeri.t__MJ11
1.000
d__Bacteria.p__Proteobacteria.c__Gammaproteobacteria.o__Vibrionales.f__Vibrionaceae.g__Aliivibrio.s__fischeri.t__ES114
d__Bacteria.p__Proteobacteria.c__Gammaproteobacteria.o__Vibrionales.f__Vibrionaceae.g__Vibrio.s__anguillarum.t__775
d__Bacteria.p__Proteobacteria.c__Gammaproteobacteria.o__Vibrionales.f__Vibrionaceae.g__Vibrio.s__metschnikovii.t__CIP_69_14
d__Bacteria.p__Proteobacteria.c__Gammaproteobacteria.o__Vibrionales.f__Vibrionaceae.g__Vibrio.s__cholerae.t__bv__albensis_VL426
d__Bacteria.p__Proteobacteria.c__Gammaproteobacteria.o__Vibrionales.f__Vibrionaceae.g__Vibrio.s__cholerae.t__O395
0.771
d__Bacteria.p__Proteobacteria.c__Gammaproteobacteria.o__Vibrionales.f__Vibrionaceae.g__Vibrio.s__cholerae.t__O395
0.764
d__Bacteria.p__Proteobacteria.c__Gammaproteobacteria.o__Vibrionales.f__Vibrionaceae.g__Vibrio.s__cholerae.t__M66_2
0.921
d__Bacteria.p__Proteobacteria.c__Gammaproteobacteria.o__Vibrionales.f__Vibrionaceae.g__Vibrio.s__cholerae.t__O1_biovar_eltor_str__N16961
d__Bacteria.p__Proteobacteria.c__Gammaproteobacteria.o__Vibrionales.f__Vibrionaceae.g__Vibrio.s__cholerae.t__1587
0.996
1.000
d__Bacteria.p__Proteobacteria.c__Gammaproteobacteria.o__Vibrionales.f__Vibrionaceae.g__Vibrio.s__cholerae.t__MZO_2
d__Bacteria.p__Proteobacteria.c__Gammaproteobacteria.o__Vibrionales.f__Vibrionaceae.g__Vibrio.s__cholerae.t__TMA_21
d__Bacteria.p__Proteobacteria.c__Gammaproteobacteria.o__Vibrionales.f__Vibrionaceae.g__Vibrio.s__cholerae.t__V51
0.950
d__Bacteria.p__Proteobacteria.c__Gammaproteobacteria.o__Vibrionales.f__Vibrionaceae.g__Vibrio.s__cholerae.t__2740_80
d__Bacteria.p__Proteobacteria.c__Gammaproteobacteria.o__Vibrionales.f__Vibrionaceae.g__Vibrio.s__cholerae.t__MO10
d__Bacteria.p__Proteobacteria.c__Gammaproteobacteria.o__Vibrionales.f__Vibrionaceae.g__Vibrio.s__cholerae.t__INDRE_91_1
d__Bacteria.p__Proteobacteria.c__Gammaproteobacteria.o__Vibrionales.f__Vibrionaceae.g__Vibrio.s__cholerae.t__MZO_3
1.000
1.000
d__Bacteria.p__Proteobacteria.c__Gammaproteobacteria.o__Vibrionales.f__Vibrionaceae.g__Vibrio.s__cholerae.t__RC27
d__Bacteria.p__Proteobacteria.c__Gammaproteobacteria.o__Vibrionales.f__Vibrionaceae.g__Vibrio.s__cholerae.t__B33
1.000
d__Bacteria.p__Proteobacteria.c__Gammaproteobacteria.o__Vibrionales.f__Vibrionaceae.g__Vibrio.s__cholerae.t__NCTC_8457
1.000
d__Bacteria.p__Proteobacteria.c__Gammaproteobacteria.o__Vibrionales.f__Vibrionaceae.g__Vibrio.s__cholerae.t__MAK_757
1.000
d__Bacteria.p__Proteobacteria.c__Gammaproteobacteria.o__Vibrionales.f__Vibrionaceae.g__Vibrio.s__cholerae.t__623_39
1.000
d__Bacteria.p__Proteobacteria.c__Gammaproteobacteria.o__Vibrionales.f__Vibrionaceae.g__Vibrio.s__cholerae.t__CT_5369_93
0.906
d__Bacteria.p__Proteobacteria.c__Gammaproteobacteria.o__Vibrionales.f__Vibrionaceae.g__Vibrio.s__cholerae.t__RC385
d__Bacteria.p__Proteobacteria.c__Gammaproteobacteria.o__Vibrionales.f__Vibrionaceae.g__Vibrio.s__cholerae.t__AM_19226
d__Bacteria.p__Proteobacteria.c__Gammaproteobacteria.o__Vibrionales.f__Vibrionaceae.g__Vibrio.s__cholerae.t__B33
0.830
d__Bacteria.p__Proteobacteria.c__Gammaproteobacteria.o__Vibrionales.f__Vibrionaceae.g__Vibrio.s__cholerae.t__RC9
0.878
d__Bacteria.p__Proteobacteria.c__Gammaproteobacteria.o__Vibrionales.f__Vibrionaceae.g__Vibrio.s__cholerae.t__BX_330286
d__Bacteria.p__Proteobacteria.c__Gammaproteobacteria.o__Vibrionales.f__Vibrionaceae.g__Vibrio.s__cholerae.t__TM_11079_80
0.861
d__Bacteria.p__Proteobacteria.c__Gammaproteobacteria.o__Vibrionales.f__Vibrionaceae.g__Vibrio.s__cholerae.t__V52
0.610
d__Bacteria.p__Proteobacteria.c__Gammaproteobacteria.o__Vibrionales.f__Vibrionaceae.g__Vibrio.s__cholera.t__CIRS_101
d__Bacteria.p__Proteobacteria.c__Gammaproteobacteria.o__Vibrionales.f__Vibrionaceae.g__Vibrio.s__cholerae.t__12129_1_
d__Bacteria.p__Proteobacteria.c__Gammaproteobacteria.o__Vibrionales.f__Vibrionaceae.g__Vibrio.s__cholerae.t__MJ_1236
d__Bacteria.p__Proteobacteria.c__Gammaproteobacteria.o__Vibrionales.f__Vibrionaceae.g__Vibrio.s__cholerae.t__LMA3894_4
1.000
d__Bacteria.p__Proteobacteria.c__Gammaproteobacteria.o__Vibrionales.f__Vibrionaceae.g__Vibrio.s__sp_.t__RC341
d__Bacteria.p__Proteobacteria.c__Gammaproteobacteria.o__Vibrionales.f__Vibrionaceae.g__Vibrio.s__sp_.t__RC586
d__Bacteria.p__Proteobacteria.c__Gammaproteobacteria.o__Vibrionales.f__Vibrionaceae.g__Vibrio.s__mimicus.t__VM603
0.627
d__Bacteria.p__Proteobacteria.c__Gammaproteobacteria.o__Vibrionales.f__Vibrionaceae.g__Vibrio.s__mimicus.t__VM223
0.353
d__Bacteria.p__Proteobacteria.c__Gammaproteobacteria.o__Vibrionales.f__Vibrionaceae.g__Vibrio.s__mimicus.t__VM573
d__Bacteria.p__Proteobacteria.c__Gammaproteobacteria.o__Vibrionales.f__Vibrionaceae.g__Vibrio.s__mimicus.t__MB_451
d__Bacteria.p__Proteobacteria.c__Gammaproteobacteria.o__Vibrionales.f__Vibrionaceae.g__Vibrio.s__furnissii.t__CIP_102972
1.000
d__Bacteria.p__Proteobacteria.c__Gammaproteobacteria.o__Vibrionales.f__Vibrionaceae.g__Vibrio.s__furnissii.t__NCTC_11218
d__Bacteria.p__Proteobacteria.c__Gammaproteobacteria.o__Vibrionales.f__Vibrionaceae.g__Vibrio.s__vulnificus.t__CMCP6
d__Bacteria.p__Proteobacteria.c__Gammaproteobacteria.o__Vibrionales.f__Vibrionaceae.g__Vibrio.s__vulnificus.t__YJ016
1.000
1.000
d__Bacteria.p__Proteobacteria.c__Gammaproteobacteria.o__Vibrionales.f__Vibrionaceae.g__Vibrio.s__vulnificus.t__MO6_24_O
d__Bacteria.p__Proteobacteria.c__Gammaproteobacteria.o__Vibrionales.f__Vibrionaceae.g__Vibrio.s__parahaemolyticus.t__AN_5034
d__Bacteria.p__Proteobacteria.c__Gammaproteobacteria.o__Vibrionales.f__Vibrionaceae.g__Vibrio.s__parahaemolyticus.t__AQ3810
0.097
d__Bacteria.p__Proteobacteria.c__Gammaproteobacteria.o__Vibrionales.f__Vibrionaceae.g__Vibrio.s__parahaemolyticus.t__AQ4037
d__Bacteria.p__Proteobacteria.c__Gammaproteobacteria.o__Vibrionales.f__Vibrionaceae.g__Vibrio.s__parahaemolyticus.t__Peru_466
d__Bacteria.p__Proteobacteria.c__Gammaproteobacteria.o__Vibrionales.f__Vibrionaceae.g__Vibrio.s__parahaemolyticus.t__K5030
d__Bacteria.p__Proteobacteria.c__Gammaproteobacteria.o__Vibrionales.f__Vibrionaceae.g__Vibrio.s__parahaemolyticus.t__RIMD_2210633
d__Bacteria.p__Proteobacteria.c__Gammaproteobacteria.o__Vibrionales.f__Vibrionaceae.g__Vibrio.s__sp_.t__Ex25
1.000
d__Bacteria.p__Proteobacteria.c__Gammaproteobacteria.o__Vibrionales.f__Vibrionaceae.g__Vibrio.s__sp_.t__Ex25
1.000
d__Bacteria.p__Proteobacteria.c__Gammaproteobacteria.o__Vibrionales.f__Vibrionaceae.g__Vibrio.s__alginolyticus.t__40B
0.999
d__Bacteria.p__Proteobacteria.c__Gammaproteobacteria.o__Vibrionales.f__Vibrionaceae.g__Vibrio.s__alginolyticus.t__12G01
d__Bacteria.p__Proteobacteria.c__Gammaproteobacteria.o__Vibrionales.f__Vibrionaceae.g__Vibrio.s__harveyi.t__HY01
0.999
d__Bacteria.p__Proteobacteria.c__Gammaproteobacteria.o__Vibrionales.f__Vibrionaceae.g__Vibrio.s__harveyi.t__ATCC_BAA_1116
1.000
d__Bacteria.p__Proteobacteria.c__Gammaproteobacteria.o__Vibrionales.f__Vibrionaceae.g__Vibrio.s__harveyi.t__1DA3
0.540
d__Bacteria.p__Proteobacteria.c__Gammaproteobacteria.o__Vibrionales.f__Vibrionaceae.g__Vibrio.s__campbellii.t__AND4
d__Bacteria.p__Proteobacteria.c__Gammaproteobacteria.o__Vibrionales.f__Vibrionaceae.g__Vibrio.s__sinaloensis.t__DSM_21326
0.996
d__Bacteria.p__Proteobacteria.c__Gammaproteobacteria.o__Vibrionales.f__Vibrionaceae.g__Vibrio.s__parahaemolyticus.t__16
0.989
d__Bacteria.p__Proteobacteria.c__Gammaproteobacteria.o__Vibrionales.f__Vibrionaceae.g__Vibrio.s__orientalis.t__CIP_102891
0.310
d__Bacteria.p__Proteobacteria.c__Gammaproteobacteria.o__Vibrionales.f__Vibrionaceae.g__Vibrio.s__brasiliensis.t__LMG_20546
d__Bacteria.p__Proteobacteria.c__Gammaproteobacteria.o__Vibrionales.f__Vibrionaceae.g__Vibrio.s__coralliilyticus.t__ATCC_BAA_450
d__Bacteria.p__Proteobacteria.c__Gammaproteobacteria.o__Vibrionales.f__Vibrionaceae.g__Vibrio.s__caribbenthicus.t__ATCC_BAA_2122
d__Bacteria.p__Proteobacteria.c__Gammaproteobacteria.o__Vibrionales.f__Vibrionaceae.g__unclassified.s__bacterium.t__SWAT_3
d__Bacteria.p__Proteobacteria.c__Gammaproteobacteria.o__Vibrionales.f__Vibrionaceae.g__Vibrio.s__splendidus.t__12B01
d__Bacteria.p__Proteobacteria.c__Gammaproteobacteria.o__Vibrionales.f__Vibrionaceae.g__Vibrio.s__sp_.t__MED222
0.977
d__Bacteria.p__Proteobacteria.c__Gammaproteobacteria.o__Vibrionales.f__Vibrionaceae.g__Vibrio.s__splendidus.t__LGP32
d__Bacteria.p__Proteobacteria.c__Gammaproteobacteria.o__Vibrionales.f__Vibrionaceae.g__Vibrio.s__shilonii.t__AK1
d__Bacteria.p__Proteobacteria.c__Gammaproteobacteria.o__Vibrionales.f__Vibrionaceae.g__Photobacterium.s__profundum.t__SS9
1.000
d__Bacteria.p__Proteobacteria.c__Gammaproteobacteria.o__Vibrionales.f__Vibrionaceae.g__Photobacterium.s__profundum.t__3TCK
d__Bacteria.p__Proteobacteria.c__Gammaproteobacteria.o__Vibrionales.f__Vibrionaceae.g__Photobacterium.s__damselae.t__subsp__damselae_CIP_102761
d__Bacteria.p__Proteobacteria.c__Gammaproteobacteria.o__Vibrionales.f__Vibrionaceae.g__Photobacterium.s__leiognathi.t__subsp__mandapamensis_svers_1_1_
d__Bacteria.p__Proteobacteria.c__Gammaproteobacteria.o__Vibrionales.f__Vibrionaceae.g__Photobacterium.s__angustum.t__S14
1.000
d__Bacteria.p__Proteobacteria.c__Gammaproteobacteria.o__Vibrionales.f__Vibrionaceae.g__Photobacterium.s__sp_.t__SKA34
d__Bacteria.p__Proteobacteria.c__Gammaproteobacteria.o__Enterobacteriales.f__Enterobacteriaceae.g__Yersinia.s__ruckeri.t__ATCC_29473
d__Bacteria.p__Proteobacteria.c__Gammaproteobacteria.o__Enterobacteriales.f__Enterobacteriaceae.g__Yersinia.s__pestis.t__bv_Antiqua__UG05_0454
d__Bacteria.p__Proteobacteria.c__Gammaproteobacteria.o__Enterobacteriales.f__Enterobacteriaceae.g__Yersinia.s__pestis.t__Antiqua
d__Bacteria.p__Proteobacteria.c__Gammaproteobacteria.o__Enterobacteriales.f__Enterobacteriaceae.g__Yersinia.s__pestis.t__bv_Orientalis__IP275
1.000
d__Bacteria.p__Proteobacteria.c__Gammaproteobacteria.o__Enterobacteriales.f__Enterobacteriaceae.g__Yersinia.s__pestis.t__FV_1
d__Bacteria.p__Proteobacteria.c__Gammaproteobacteria.o__Enterobacteriales.f__Enterobacteriaceae.g__Yersinia.s__pestis.t__D182038
d__Bacteria.p__Proteobacteria.c__Gammaproteobacteria.o__Enterobacteriales.f__Enterobacteriaceae.g__Yersinia.s__pestis.t__D106004
0.881
d__Bacteria.p__Proteobacteria.c__Gammaproteobacteria.o__Enterobacteriales.f__Enterobacteriaceae.g__Yersinia.s__pestis.t__Z176003
0.893
d__Bacteria.p__Proteobacteria.c__Gammaproteobacteria.o__Enterobacteriales.f__Enterobacteriaceae.g__Yersinia.s__pestis.t__biovar_Medievalis_str__Harbin_35
d__Bacteria.p__Proteobacteria.c__Gammaproteobacteria.o__Enterobacteriales.f__Enterobacteriaceae.g__Yersinia.s__pestis.t__biovar_Orientalis_str__India_195
d__Bacteria.p__Proteobacteria.c__Gammaproteobacteria.o__Enterobacteriales.f__Enterobacteriaceae.g__Yersinia.s__pestis.t__Nepal516
d__Bacteria.p__Proteobacteria.c__Gammaproteobacteria.o__Enterobacteriales.f__Enterobacteriaceae.g__Yersinia.s__pestis.t__KIM_D27
d__Bacteria.p__Proteobacteria.c__Gammaproteobacteria.o__Enterobacteriales.f__Enterobacteriaceae.g__Yersinia.s__pestis.t__Nepal516A
d__Bacteria.p__Proteobacteria.c__Gammaproteobacteria.o__Enterobacteriales.f__Enterobacteriaceae.g__Yersinia.s__pestis.t__bv_Mediaevalis__K1973002
0.996
d__Bacteria.p__Proteobacteria.c__Gammaproteobacteria.o__Enterobacteriales.f__Enterobacteriaceae.g__Yersinia.s__pestis.t__bv_Orientalis__F1991016
0.991
d__Bacteria.p__Proteobacteria.c__Gammaproteobacteria.o__Enterobacteriales.f__Enterobacteriaceae.g__Yersinia.s__pestis.t__CA88_4125
0.908
d__Bacteria.p__Proteobacteria.c__Gammaproteobacteria.o__Enterobacteriales.f__Enterobacteriaceae.g__Yersinia.s__pestis.t__bv_Antiqua__E1979001
0.000
d__Bacteria.p__Proteobacteria.c__Gammaproteobacteria.o__Enterobacteriales.f__Enterobacteriaceae.g__Yersinia.s__pestis.t__bv_Orientalis__MG05_1020
d__Bacteria.p__Proteobacteria.c__Gammaproteobacteria.o__Enterobacteriales.f__Enterobacteriaceae.g__Yersinia.s__pestis.t__bv_Antiqua__B42003004
d__Bacteria.p__Proteobacteria.c__Gammaproteobacteria.o__Enterobacteriales.f__Enterobacteriaceae.g__Yersinia.s__pestis.t__CO92
0.992
d__Bacteria.p__Proteobacteria.c__Gammaproteobacteria.o__Enterobacteriales.f__Enterobacteriaceae.g__Yersinia.s__pestis.t__biovar_Orientalis_str__PEXU2
d__Bacteria.p__Proteobacteria.c__Gammaproteobacteria.o__Enterobacteriales.f__Enterobacteriaceae.g__Yersinia.s__pestis.t__KIM
d__Bacteria.p__Proteobacteria.c__Gammaproteobacteria.o__Enterobacteriales.f__Enterobacteriaceae.g__Yersinia.s__pestis.t__Angola
0.782
d__Bacteria.p__Proteobacteria.c__Gammaproteobacteria.o__Enterobacteriales.f__Enterobacteriaceae.g__Yersinia.s__pestis.t__Pestoides_F
d__Bacteria.p__Proteobacteria.c__Gammaproteobacteria.o__Enterobacteriales.f__Enterobacteriaceae.g__Yersinia.s__pseudotuberculosis.t__PB1__
0.959
d__Bacteria.p__Proteobacteria.c__Gammaproteobacteria.o__Enterobacteriales.f__Enterobacteriaceae.g__Yersinia.s__pseudotuberculosis.t__IP_32953
d__Bacteria.p__Proteobacteria.c__Gammaproteobacteria.o__Enterobacteriales.f__Enterobacteriaceae.g__Yersinia.s__pseudotuberculosis.t__IP_31758
0.907
d__Bacteria.p__Proteobacteria.c__Gammaproteobacteria.o__Enterobacteriales.f__Enterobacteriaceae.g__Yersinia.s__pestis.t__Pestoides_A
0.862
d__Bacteria.p__Proteobacteria.c__Gammaproteobacteria.o__Enterobacteriales.f__Enterobacteriaceae.g__Yersinia.s__pestis.t__bv_Microtus__91001
d__Bacteria.p__Proteobacteria.c__Gammaproteobacteria.o__Enterobacteriales.f__Enterobacteriaceae.g__Yersinia.s__pseudotuberculosis.t__YPIII
d__Bacteria.p__Proteobacteria.c__Gammaproteobacteria.o__Enterobacteriales.f__Enterobacteriaceae.g__Yersinia.s__kristensenii.t__ATCC_33638
d__Bacteria.p__Proteobacteria.c__Gammaproteobacteria.o__Enterobacteriales.f__Enterobacteriaceae.g__Yersinia.s__enterocolitica.t__subsp__enterocolitica_8081
d__Bacteria.p__Proteobacteria.c__Gammaproteobacteria.o__Enterobacteriales.f__Enterobacteriaceae.g__Yersinia.s__enterocolitica.t__subsp__palearctica_105_5R_r_
0.995
d__Bacteria.p__Proteobacteria.c__Gammaproteobacteria.o__Enterobacteriales.f__Enterobacteriaceae.g__Yersinia.s__enterocolitica.t__subsp__palearctica_Y11
d__Bacteria.p__Proteobacteria.c__Gammaproteobacteria.o__Enterobacteriales.f__Enterobacteriaceae.g__Yersinia.s__intermedia.t__ATCC_29909
0.962
d__Bacteria.p__Proteobacteria.c__Gammaproteobacteria.o__Enterobacteriales.f__Enterobacteriaceae.g__Yersinia.s__aldovae.t__ATCC_35236
0.339
d__Bacteria.p__Proteobacteria.c__Gammaproteobacteria.o__Enterobacteriales.f__Enterobacteriaceae.g__Yersinia.s__mollaretii.t__ATCC_43969
1.000
d__Bacteria.p__Proteobacteria.c__Gammaproteobacteria.o__Enterobacteriales.f__Enterobacteriaceae.g__Yersinia.s__bercovieri.t__ATCC_43970
d__Bacteria.p__Proteobacteria.c__Gammaproteobacteria.o__Enterobacteriales.f__Enterobacteriaceae.g__Yersinia.s__frederiksenii.t__ATCC_33641
0.984
d__Bacteria.p__Proteobacteria.c__Gammaproteobacteria.o__Enterobacteriales.f__Enterobacteriaceae.g__Yersinia.s__rohdei.t__ATCC_43380
d__Bacteria.p__Proteobacteria.c__Gammaproteobacteria.o__Enterobacteriales.f__Enterobacteriaceae.g__Pectobacterium.s__wasabiae.t__WPP163
1.000
d__Bacteria.p__Proteobacteria.c__Gammaproteobacteria.o__Enterobacteriales.f__Enterobacteriaceae.g__Pectobacterium.s__carotovora.t__subsp__atroseptica_SCRI1043
0.124
d__Bacteria.p__Proteobacteria.c__Gammaproteobacteria.o__Enterobacteriales.f__Enterobacteriaceae.g__Pectobacterium.s__carotovorum.t__subsp__carotovorum_WPP14
0.997
d__Bacteria.p__Proteobacteria.c__Gammaproteobacteria.o__Enterobacteriales.f__Enterobacteriaceae.g__Pectobacterium.s__carotovorum.t__subsp__brasiliensis_PBR1692
d__Bacteria.p__Proteobacteria.c__Gammaproteobacteria.o__Enterobacteriales.f__Enterobacteriaceae.g__Pectobacterium.s__carotovorum.t__subsp__carotovorum_PC1
1.000
d__Bacteria.p__Proteobacteria.c__Gammaproteobacteria.o__Enterobacteriales.f__Enterobacteriaceae.g__Dickeya.s__dadantii.t__Ech703
d__Bacteria.p__Proteobacteria.c__Gammaproteobacteria.o__Enterobacteriales.f__Enterobacteriaceae.g__Dickeya.s__dadantii.t__3937
d__Bacteria.p__Proteobacteria.c__Gammaproteobacteria.o__Enterobacteriales.f__Enterobacteriaceae.g__Dickeya.s__dadantii.t__Ech586
0.141
d__Bacteria.p__Proteobacteria.c__Gammaproteobacteria.o__Enterobacteriales.f__Enterobacteriaceae.g__Dickeya.s__zeae.t__Ech1591
1.000
1.000
1.000
0.984
1.000
0.791
0.040

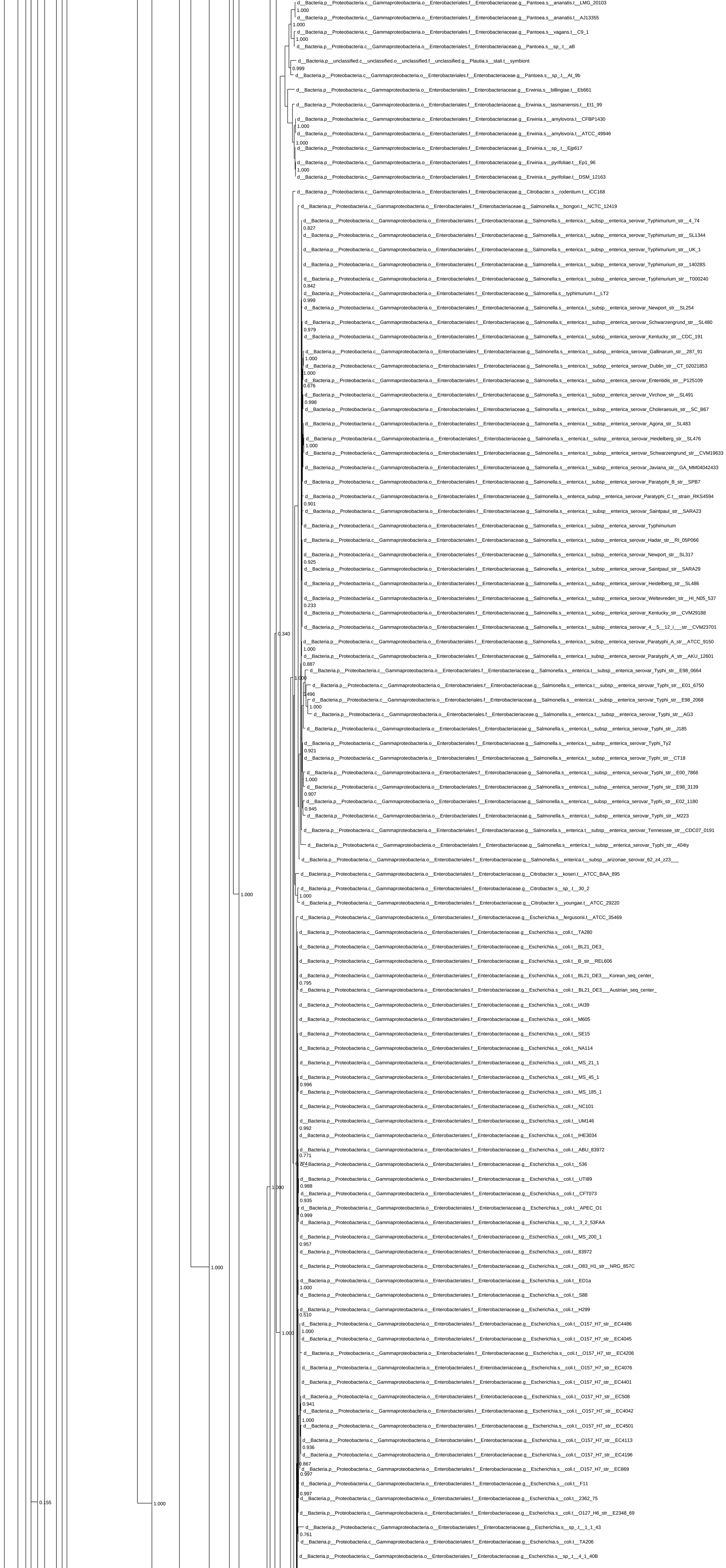

d__Bacteria.p__Proteobacteria.c__Gammaproteobacteria.o__Enterobacteriales.f__Enterobacteriaceae.g__Pantoea.s__ananatis.t__LMG_20103
1.000
d__Bacteria.p__Proteobacteria.c__Gammaproteobacteria.o__Enterobacteriales.f__Enterobacteriaceae.g__Pantoea.s__ananatis.t__AJ13355
1.000
d__Bacteria.p__Proteobacteria.c__Gammaproteobacteria.o__Enterobacteriales.f__Enterobacteriaceae.g__Pantoea.s__vagans.t__C9_1
1.000
d__Bacteria.p__Proteobacteria.c__Gammaproteobacteria.o__Enterobacteriales.f__Enterobacteriaceae.g__Pantoea.s__sp_.t__aB
d__Bacteria.p__unclassified.c__unclassified.o__unclassified.f__unclassified.g__Plautia.s__stali.t__symbiont
0.999
d__Bacteria.p__Proteobacteria.c__Gammaproteobacteria.o__Enterobacteriales.f__Enterobacteriaceae.g__Pantoea.s__sp_.t__At_9b
d__Bacteria.p__Proteobacteria.c__Gammaproteobacteria.o__Enterobacteriales.f__Enterobacteriaceae.g__Erwinia.s__billingiae.t__Eb661
d__Bacteria.p__Proteobacteria.c__Gammaproteobacteria.o__Enterobacteriales.f__Enterobacteriaceae.g__Erwinia.s__tasmaniensis.t__Et1_99
d__Bacteria.p__Proteobacteria.c__Gammaproteobacteria.o__Enterobacteriales.f__Enterobacteriaceae.g__Erwinia.s__amylovora.t__CFBP1430
1.000
d__Bacteria.p__Proteobacteria.c__Gammaproteobacteria.o__Enterobacteriales.f__Enterobacteriaceae.g__Erwinia.s__amylovora.t__ATCC_49946
1.000
d__Bacteria.p__Proteobacteria.c__Gammaproteobacteria.o__Enterobacteriales.f__Enterobacteriaceae.g__Erwinia.s__sp_.t__Ejp617
d__Bacteria.p__Proteobacteria.c__Gammaproteobacteria.o__Enterobacteriales.f__Enterobacteriaceae.g__Erwinia.s__pyrifoliae.t__Ep1_96
1.000
d__Bacteria.p__Proteobacteria.c__Gammaproteobacteria.o__Enterobacteriales.f__Enterobacteriaceae.g__Erwinia.s__pyrifoliae.t__DSM_12163
d__Bacteria.p__Proteobacteria.c__Gammaproteobacteria.o__Enterobacteriales.f__Enterobacteriaceae.g__Citrobacter.s__rodentium.t__ICC168
d__Bacteria.p__Proteobacteria.c__Gammaproteobacteria.o__Enterobacteriales.f__Enterobacteriaceae.g__Salmonella.s__bongori.t__NCTC_12419
d__Bacteria.p__Proteobacteria.c__Gammaproteobacteria.o__Enterobacteriales.f__Enterobacteriaceae.g__Salmonella.s__enterica.t__subsp__enterica_serovar_Typhimurium_str__4_74
0.827
d__Bacteria.p__Proteobacteria.c__Gammaproteobacteria.o__Enterobacteriales.f__Enterobacteriaceae.g__Salmonella.s__enterica.t__subsp__enterica_serovar_Typhimurium_str__SL1344
d__Bacteria.p__Proteobacteria.c__Gammaproteobacteria.o__Enterobacteriales.f__Enterobacteriaceae.g__Salmonella.s__enterica.t__subsp__enterica_serovar_Typhimurium_str__UK_1
d__Bacteria.p__Proteobacteria.c__Gammaproteobacteria.o__Enterobacteriales.f__Enterobacteriaceae.g__Salmonella.s__enterica.t__subsp__enterica_serovar_Typhimurium_str__14028S
d__Bacteria.p__Proteobacteria.c__Gammaproteobacteria.o__Enterobacteriales.f__Enterobacteriaceae.g__Salmonella.s__enterica.t__subsp__enterica_serovar_Typhimurium_str__T000240
0.842
d__Bacteria.p__Proteobacteria.c__Gammaproteobacteria.o__Enterobacteriales.f__Enterobacteriaceae.g__Salmonella.s__typhimurium.t__LT2
0.999
d__Bacteria.p__Proteobacteria.c__Gammaproteobacteria.o__Enterobacteriales.f__Enterobacteriaceae.g__Salmonella.s__enterica.t__subsp__enterica_serovar_Newport_str__SL254
d__Bacteria.p__Proteobacteria.c__Gammaproteobacteria.o__Enterobacteriales.f__Enterobacteriaceae.g__Salmonella.s__enterica.t__subsp__enterica_serovar_Schwarzengrund_str__SL480
0.979
d__Bacteria.p__Proteobacteria.c__Gammaproteobacteria.o__Enterobacteriales.f__Enterobacteriaceae.g__Salmonella.s__enterica.t__subsp__enterica_serovar_Kentucky_str__CDC_191
d__Bacteria.p__Proteobacteria.c__Gammaproteobacteria.o__Enterobacteriales.f__Enterobacteriaceae.g__Salmonella.s__enterica.t__subsp__enterica_serovar_Gallinarum_str__287_91
1.000
d__Bacteria.p__Proteobacteria.c__Gammaproteobacteria.o__Enterobacteriales.f__Enterobacteriaceae.g__Salmonella.s__enterica.t__subsp__enterica_serovar_Dublin_str__CT_02021853
1.000
d__Bacteria.p__Proteobacteria.c__Gammaproteobacteria.o__Enterobacteriales.f__Enterobacteriaceae.g__Salmonella.s__enterica.t__subsp__enterica_serovar_Enteritidis_str__P125109
0.676
d__Bacteria.p__Proteobacteria.c__Gammaproteobacteria.o__Enterobacteriales.f__Enterobacteriaceae.g__Salmonella.s__enterica.t__subsp__enterica_serovar_Virchow_str__SL491
0.998
d__Bacteria.p__Proteobacteria.c__Gammaproteobacteria.o__Enterobacteriales.f__Enterobacteriaceae.g__Salmonella.s__enterica.t__subsp__enterica_serovar_Choleraesuis_str__SC_B67
d__Bacteria.p__Proteobacteria.c__Gammaproteobacteria.o__Enterobacteriales.f__Enterobacteriaceae.g__Salmonella.s__enterica.t__subsp__enterica_serovar_Agona_str__SL483
d__Bacteria.p__Proteobacteria.c__Gammaproteobacteria.o__Enterobacteriales.f__Enterobacteriaceae.g__Salmonella.s__enterica.t__subsp__enterica_serovar_Heidelberg_str__SL476
1.000
d__Bacteria.p__Proteobacteria.c__Gammaproteobacteria.o__Enterobacteriales.f__Enterobacteriaceae.g__Salmonella.s__enterica.t__subsp__enterica_serovar_Schwarzengrund_str__CVM19633
d__Bacteria.p__Proteobacteria.c__Gammaproteobacteria.o__Enterobacteriales.f__Enterobacteriaceae.g__Salmonella.s__enterica.t__subsp__enterica_serovar_Javiana_str__GA_MM04042433
d__Bacteria.p__Proteobacteria.c__Gammaproteobacteria.o__Enterobacteriales.f__Enterobacteriaceae.g__Salmonella.s__enterica.t__subsp__enterica_serovar_Paratyphi_B_str__SPB7
d__Bacteria.p__Proteobacteria.c__Gammaproteobacteria.o__Enterobacteriales.f__Enterobacteriaceae.g__Salmonella.s__enterica_subsp__enterica_serovar_Paratyphi_C.t__strain_RKS4594
0.901
d__Bacteria.p__Proteobacteria.c__Gammaproteobacteria.o__Enterobacteriales.f__Enterobacteriaceae.g__Salmonella.s__enterica.t__subsp__enterica_serovar_Saintpaul_str__SARA23
d__Bacteria.p__Proteobacteria.c__Gammaproteobacteria.o__Enterobacteriales.f__Enterobacteriaceae.g__Salmonella.s__enterica.t__subsp__enterica_serovar_Typhimurium
d__Bacteria.p__Proteobacteria.c__Gammaproteobacteria.o__Enterobacteriales.f__Enterobacteriaceae.g__Salmonella.s__enterica.t__subsp__enterica_serovar_Hadar_str__RI_05P066
d__Bacteria.p__Proteobacteria.c__Gammaproteobacteria.o__Enterobacteriales.f__Enterobacteriaceae.g__Salmonella.s__enterica.t__subsp__enterica_serovar_Newport_str__SL317
0.925
d__Bacteria.p__Proteobacteria.c__Gammaproteobacteria.o__Enterobacteriales.f__Enterobacteriaceae.g__Salmonella.s__enterica.t__subsp__enterica_serovar_Saintpaul_str__SARA29
d__Bacteria.p__Proteobacteria.c__Gammaproteobacteria.o__Enterobacteriales.f__Enterobacteriaceae.g__Salmonella.s__enterica.t__subsp__enterica_serovar_Heidelberg_str__SL486
d__Bacteria.p__Proteobacteria.c__Gammaproteobacteria.o__Enterobacteriales.f__Enterobacteriaceae.g__Salmonella.s__enterica.t__subsp__enterica_serovar_Weltevreden_str__HI_N05_537
0.233
d__Bacteria.p__Proteobacteria.c__Gammaproteobacteria.o__Enterobacteriales.f__Enterobacteriaceae.g__Salmonella.s__enterica.t__subsp__enterica_serovar_Kentucky_str__CVM29188
0.340
d__Bacteria.p__Proteobacteria.c__Gammaproteobacteria.o__Enterobacteriales.f__Enterobacteriaceae.g__Salmonella.s__enterica.t__subsp__enterica_serovar_4__5__12_i___str__CVM23701
d__Bacteria.p__Proteobacteria.c__Gammaproteobacteria.o__Enterobacteriales.f__Enterobacteriaceae.g__Salmonella.s__enterica.t__subsp__enterica_serovar_Paratyphi_A_str__ATCC_9150
1.000
d__Bacteria.p__Proteobacteria.c__Gammaproteobacteria.o__Enterobacteriales.f__Enterobacteriaceae.g__Salmonella.s__enterica.t__subsp__enterica_serovar_Paratyphi_A_str__AKU_12601
0.887
d__Bacteria.p__Proteobacteria.c__Gammaproteobacteria.o__Enterobacteriales.f__Enterobacteriaceae.g__Salmonella.s__enterica.t__subsp__enterica_serovar_Typhi_str__E98_0664
1.000
d__Bacteria.p__Proteobacteria.c__Gammaproteobacteria.o__Enterobacteriales.f__Enterobacteriaceae.g__Salmonella.s__enterica.t__subsp__enterica_serovar_Typhi_str__E01_6750
0.496
d__Bacteria.p__Proteobacteria.c__Gammaproteobacteria.o__Enterobacteriales.f__Enterobacteriaceae.g__Salmonella.s__enterica.t__subsp__enterica_serovar_Typhi_str__E98_2068
1.000
d__Bacteria.p__Proteobacteria.c__Gammaproteobacteria.o__Enterobacteriales.f__Enterobacteriaceae.g__Salmonella.s__enterica.t__subsp__enterica_serovar_Typhi_str__AG3
d__Bacteria.p__Proteobacteria.c__Gammaproteobacteria.o__Enterobacteriales.f__Enterobacteriaceae.g__Salmonella.s__enterica.t__subsp__enterica_serovar_Typhi_str__J185
d__Bacteria.p__Proteobacteria.c__Gammaproteobacteria.o__Enterobacteriales.f__Enterobacteriaceae.g__Salmonella.s__enterica.t__subsp__enterica_serovar_Typhi_Ty2
0.921
d__Bacteria.p__Proteobacteria.c__Gammaproteobacteria.o__Enterobacteriales.f__Enterobacteriaceae.g__Salmonella.s__enterica.t__subsp__enterica_serovar_Typhi_str__CT18
d__Bacteria.p__Proteobacteria.c__Gammaproteobacteria.o__Enterobacteriales.f__Enterobacteriaceae.g__Salmonella.s__enterica.t__subsp__enterica_serovar_Typhi_str__E00_7866
1.000
d__Bacteria.p__Proteobacteria.c__Gammaproteobacteria.o__Enterobacteriales.f__Enterobacteriaceae.g__Salmonella.s__enterica.t__subsp__enterica_serovar_Typhi_str__E98_3139
0.907
d__Bacteria.p__Proteobacteria.c__Gammaproteobacteria.o__Enterobacteriales.f__Enterobacteriaceae.g__Salmonella.s__enterica.t__subsp__enterica_serovar_Typhi_str__E02_1180
0.945
d__Bacteria.p__Proteobacteria.c__Gammaproteobacteria.o__Enterobacteriales.f__Enterobacteriaceae.g__Salmonella.s__enterica.t__subsp__enterica_serovar_Typhi_str__M223
d__Bacteria.p__Proteobacteria.c__Gammaproteobacteria.o__Enterobacteriales.f__Enterobacteriaceae.g__Salmonella.s__enterica.t__subsp__enterica_serovar_Tennessee_str__CDC07_0191
d__Bacteria.p__Proteobacteria.c__Gammaproteobacteria.o__Enterobacteriales.f__Enterobacteriaceae.g__Salmonella.s__enterica.t__subsp__enterica_serovar_Typhi_str__404ty
d__Bacteria.p__Proteobacteria.c__Gammaproteobacteria.o__Enterobacteriales.f__Enterobacteriaceae.g__Salmonella.s__enterica.t__subsp__arizonae_serovar_62_z4_z23___
d__Bacteria.p__Proteobacteria.c__Gammaproteobacteria.o__Enterobacteriales.f__Enterobacteriaceae.g__Citrobacter.s__koseri.t__ATCC_BAA_895
d__Bacteria.p__Proteobacteria.c__Gammaproteobacteria.o__Enterobacteriales.f__Enterobacteriaceae.g__Citrobacter.s__sp_.t__30_2
1.000
1.000
d__Bacteria.p__Proteobacteria.c__Gammaproteobacteria.o__Enterobacteriales.f__Enterobacteriaceae.g__Citrobacter.s__youngae.t__ATCC_29220
d__Bacteria.p__Proteobacteria.c__Gammaproteobacteria.o__Enterobacteriales.f__Enterobacteriaceae.g__Escherichia.s__fergusonii.t__ATCC_35469
d__Bacteria.p__Proteobacteria.c__Gammaproteobacteria.o__Enterobacteriales.f__Enterobacteriaceae.g__Escherichia.s__coli.t__TA280
d__Bacteria.p__Proteobacteria.c__Gammaproteobacteria.o__Enterobacteriales.f__Enterobacteriaceae.g__Escherichia.s__coli.t__BL21_DE3_
d__Bacteria.p__Proteobacteria.c__Gammaproteobacteria.o__Enterobacteriales.f__Enterobacteriaceae.g__Escherichia.s__coli.t__B_str__REL606
d__Bacteria.p__Proteobacteria.c__Gammaproteobacteria.o__Enterobacteriales.f__Enterobacteriaceae.g__Escherichia.s__coli.t__BL21_DE3___Korean_seq_center_
0.795
d__Bacteria.p__Proteobacteria.c__Gammaproteobacteria.o__Enterobacteriales.f__Enterobacteriaceae.g__Escherichia.s__coli.t__BL21_DE3___Austrian_seq_center_
d__Bacteria.p__Proteobacteria.c__Gammaproteobacteria.o__Enterobacteriales.f__Enterobacteriaceae.g__Escherichia.s__coli.t__IAI39
d__Bacteria.p__Proteobacteria.c__Gammaproteobacteria.o__Enterobacteriales.f__Enterobacteriaceae.g__Escherichia.s__coli.t__M605
d__Bacteria.p__Proteobacteria.c__Gammaproteobacteria.o__Enterobacteriales.f__Enterobacteriaceae.g__Escherichia.s__coli.t__SE15
d__Bacteria.p__Proteobacteria.c__Gammaproteobacteria.o__Enterobacteriales.f__Enterobacteriaceae.g__Escherichia.s__coli.t__NA114
d__Bacteria.p__Proteobacteria.c__Gammaproteobacteria.o__Enterobacteriales.f__Enterobacteriaceae.g__Escherichia.s__coli.t__MS_21_1
d__Bacteria.p__Proteobacteria.c__Gammaproteobacteria.o__Enterobacteriales.f__Enterobacteriaceae.g__Escherichia.s__coli.t__MS_45_1
0.996
d__Bacteria.p__Proteobacteria.c__Gammaproteobacteria.o__Enterobacteriales.f__Enterobacteriaceae.g__Escherichia.s__coli.t__MS_185_1
d__Bacteria.p__Proteobacteria.c__Gammaproteobacteria.o__Enterobacteriales.f__Enterobacteriaceae.g__Escherichia.s__coli.t__NC101
d__Bacteria.p__Proteobacteria.c__Gammaproteobacteria.o__Enterobacteriales.f__Enterobacteriaceae.g__Escherichia.s__coli.t__UM146
0.992
d__Bacteria.p__Proteobacteria.c__Gammaproteobacteria.o__Enterobacteriales.f__Enterobacteriaceae.g__Escherichia.s__coli.t__IHE3034
d__Bacteria.p__Proteobacteria.c__Gammaproteobacteria.o__Enterobacteriales.f__Enterobacteriaceae.g__Escherichia.s__coli.t__ABU_83972
0.771
0.774
d__Bacteria.p__Proteobacteria.c__Gammaproteobacteria.o__Enterobacteriales.f__Enterobacteriaceae.g__Escherichia.s__coli.t__536
d__Bacteria.p__Proteobacteria.c__Gammaproteobacteria.o__Enterobacteriales.f__Enterobacteriaceae.g__Escherichia.s__coli.t__UTI89
0.988
1.000
d__Bacteria.p__Proteobacteria.c__Gammaproteobacteria.o__Enterobacteriales.f__Enterobacteriaceae.g__Escherichia.s__coli.t__CFT073
0.935
d__Bacteria.p__Proteobacteria.c__Gammaproteobacteria.o__Enterobacteriales.f__Enterobacteriaceae.g__Escherichia.s__coli.t__APEC_O1
0.999
d__Bacteria.p__Proteobacteria.c__Gammaproteobacteria.o__Enterobacteriales.f__Enterobacteriaceae.g__Escherichia.s__sp_.t__3_2_53FAA
d__Bacteria.p__Proteobacteria.c__Gammaproteobacteria.o__Enterobacteriales.f__Enterobacteriaceae.g__Escherichia.s__coli.t__MS_200_1
0.957
d__Bacteria.p__Proteobacteria.c__Gammaproteobacteria.o__Enterobacteriales.f__Enterobacteriaceae.g__Escherichia.s__coli.t__83972
1.000
d__Bacteria.p__Proteobacteria.c__Gammaproteobacteria.o__Enterobacteriales.f__Enterobacteriaceae.g__Escherichia.s__coli.t__O83_H1_str__NRG_857C
d__Bacteria.p__Proteobacteria.c__Gammaproteobacteria.o__Enterobacteriales.f__Enterobacteriaceae.g__Escherichia.s__coli.t__ED1a
1.000
d__Bacteria.p__Proteobacteria.c__Gammaproteobacteria.o__Enterobacteriales.f__Enterobacteriaceae.g__Escherichia.s__coli.t__S88
d__Bacteria.p__Proteobacteria.c__Gammaproteobacteria.o__Enterobacteriales.f__Enterobacteriaceae.g__Escherichia.s__coli.t__H299
0.510
d__Bacteria.p__Proteobacteria.c__Gammaproteobacteria.o__Enterobacteriales.f__Enterobacteriaceae.g__Escherichia.s__coli.t__O157_H7_str__EC4486
1.000
1.000
d__Bacteria.p__Proteobacteria.c__Gammaproteobacteria.o__Enterobacteriales.f__Enterobacteriaceae.g__Escherichia.s__coli.t__O157_H7_str__EC4045
d__Bacteria.p__Proteobacteria.c__Gammaproteobacteria.o__Enterobacteriales.f__Enterobacteriaceae.g__Escherichia.s__coli.t__O157_H7_str__EC4206
d__Bacteria.p__Proteobacteria.c__Gammaproteobacteria.o__Enterobacteriales.f__Enterobacteriaceae.g__Escherichia.s__coli.t__O157_H7_str__EC4076
d__Bacteria.p__Proteobacteria.c__Gammaproteobacteria.o__Enterobacteriales.f__Enterobacteriaceae.g__Escherichia.s__coli.t__O157_H7_str__EC4401
d__Bacteria.p__Proteobacteria.c__Gammaproteobacteria.o__Enterobacteriales.f__Enterobacteriaceae.g__Escherichia.s__coli.t__O157_H7_str__EC508
0.941
d__Bacteria.p__Proteobacteria.c__Gammaproteobacteria.o__Enterobacteriales.f__Enterobacteriaceae.g__Escherichia.s__coli.t__O157_H7_str__EC4042
1.000
d__Bacteria.p__Proteobacteria.c__Gammaproteobacteria.o__Enterobacteriales.f__Enterobacteriaceae.g__Escherichia.s__coli.t__O157_H7_str__EC4501
d__Bacteria.p__Proteobacteria.c__Gammaproteobacteria.o__Enterobacteriales.f__Enterobacteriaceae.g__Escherichia.s__coli.t__O157_H7_str__EC4113
0.936
d__Bacteria.p__Proteobacteria.c__Gammaproteobacteria.o__Enterobacteriales.f__Enterobacteriaceae.g__Escherichia.s__coli.t__O157_H7_str__EC4196
0.867
d__Bacteria.p__Proteobacteria.c__Gammaproteobacteria.o__Enterobacteriales.f__Enterobacteriaceae.g__Escherichia.s__coli.t__O157_H7_str__EC869
0.997
d__Bacteria.p__Proteobacteria.c__Gammaproteobacteria.o__Enterobacteriales.f__Enterobacteriaceae.g__Escherichia.s__coli.t__F11
0.997
0.155
1.000
d__Bacteria.p__Proteobacteria.c__Gammaproteobacteria.o__Enterobacteriales.f__Enterobacteriaceae.g__Escherichia.s__coli.t__2362_75
d__Bacteria.p__Proteobacteria.c__Gammaproteobacteria.o__Enterobacteriales.f__Enterobacteriaceae.g__Escherichia.s__coli.t__O127_H6_str__E2348_69
d__Bacteria.p__Proteobacteria.c__Gammaproteobacteria.o__Enterobacteriales.f__Enterobacteriaceae.g__Escherichia.s__sp_.t__1_1_43
0.761
d__Bacteria.p__Proteobacteria.c__Gammaproteobacteria.o__Enterobacteriales.f__Enterobacteriaceae.g__Escherichia.s__coli.t__TA206
d__Bacteria.p__Proteobacteria.c__Gammaproteobacteria.o__Enterobacteriales.f__Enterobacteriaceae.g__Escherichia.s__sp_.t__4_1_40B

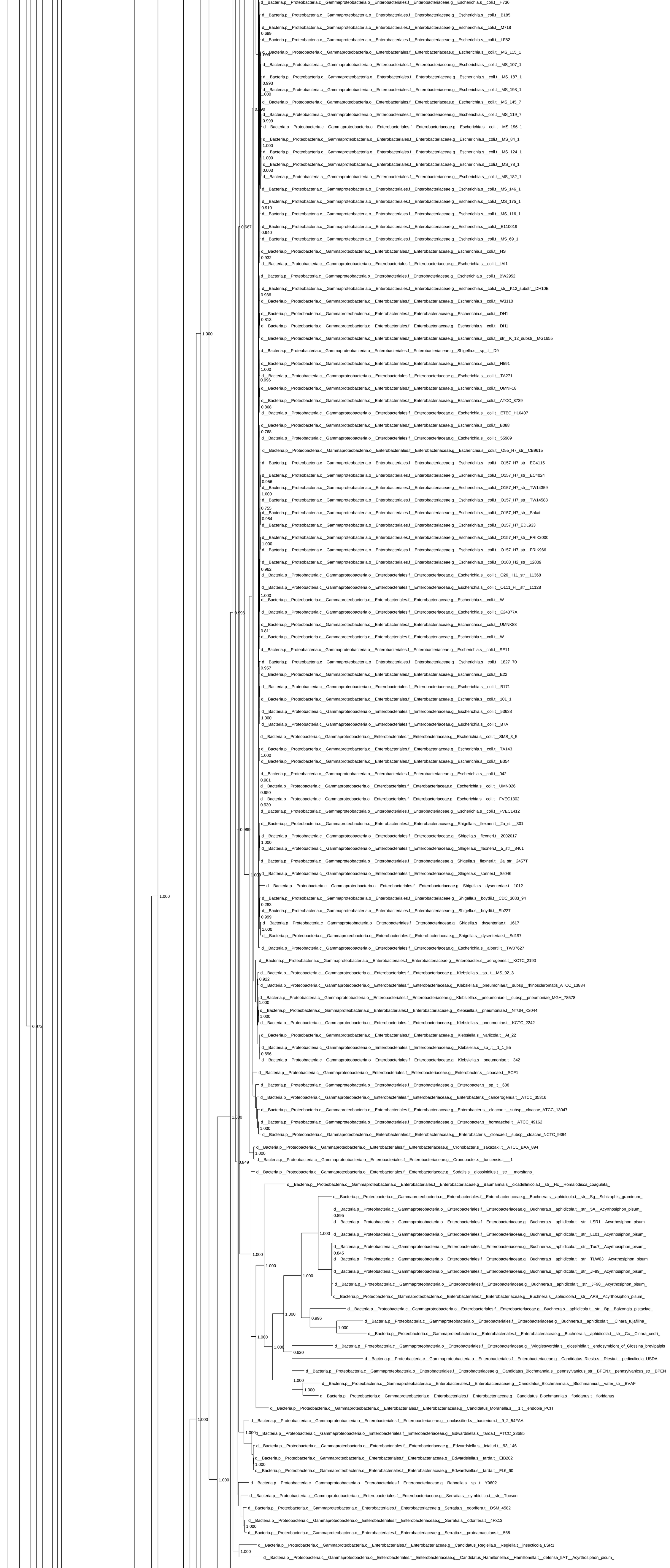
d__Bacteria.p__Proteobacteria.c__Gammaproteobacteria.o__Enterobacteriales.f__Enterobacteriaceae.g__Escherichia.s__coli.t__H736
d__Bacteria.p__Proteobacteria.c__Gammaproteobacteria.o__Enterobacteriales.f__Enterobacteriaceae.g__Escherichia.s__coli.t__B185
d__Bacteria.p__Proteobacteria.c__Gammaproteobacteria.o__Enterobacteriales.f__Enterobacteriaceae.g__Escherichia.s__coli.t__M718
0.689
d__Bacteria.p__Proteobacteria.c__Gammaproteobacteria.o__Enterobacteriales.f__Enterobacteriaceae.g__Escherichia.s__coli.t__LF82
d__Bacteria.p__Proteobacteria.c__Gammaproteobacteria.o__Enterobacteriales.f__Enterobacteriaceae.g__Escherichia.s__coli.t__MS_115_1
1.000
d__Bacteria.p__Proteobacteria.c__Gammaproteobacteria.o__Enterobacteriales.f__Enterobacteriaceae.g__Escherichia.s__coli.t__MS_107_1
d__Bacteria.p__Proteobacteria.c__Gammaproteobacteria.o__Enterobacteriales.f__Enterobacteriaceae.g__Escherichia.s__coli.t__MS_187_1
0.993
d__Bacteria.p__Proteobacteria.c__Gammaproteobacteria.o__Enterobacteriales.f__Enterobacteriaceae.g__Escherichia.s__coli.t__MS_198_1
1.000
d__Bacteria.p__Proteobacteria.c__Gammaproteobacteria.o__Enterobacteriales.f__Enterobacteriaceae.g__Escherichia.s__coli.t__MS_145_7
d__Bacteria.p__Proteobacteria.c__Gammaproteobacteria.o__Enterobacteriales.f__Enterobacteriaceae.g__Escherichia.s__coli.t__MS_119_7
0.999
d__Bacteria.p__Proteobacteria.c__Gammaproteobacteria.o__Enterobacteriales.f__Enterobacteriaceae.g__Escherichia.s__coli.t__MS_196_1
d__Bacteria.p__Proteobacteria.c__Gammaproteobacteria.o__Enterobacteriales.f__Enterobacteriaceae.g__Escherichia.s__coli.t__MS_84_1
1.000
d__Bacteria.p__Proteobacteria.c__Gammaproteobacteria.o__Enterobacteriales.f__Enterobacteriaceae.g__Escherichia.s__coli.t__MS_124_1
1.000
d__Bacteria.p__Proteobacteria.c__Gammaproteobacteria.o__Enterobacteriales.f__Enterobacteriaceae.g__Escherichia.s__coli.t__MS_78_1
0.603
d__Bacteria.p__Proteobacteria.c__Gammaproteobacteria.o__Enterobacteriales.f__Enterobacteriaceae.g__Escherichia.s__coli.t__MS_182_1
d__Bacteria.p__Proteobacteria.c__Gammaproteobacteria.o__Enterobacteriales.f__Enterobacteriaceae.g__Escherichia.s__coli.t__MS_146_1
d__Bacteria.p__Proteobacteria.c__Gammaproteobacteria.o__Enterobacteriales.f__Enterobacteriaceae.g__Escherichia.s__coli.t__MS_175_1
0.910
d__Bacteria.p__Proteobacteria.c__Gammaproteobacteria.o__Enterobacteriales.f__Enterobacteriaceae.g__Escherichia.s__coli.t__MS_116_1
0.667
d__Bacteria.p__Proteobacteria.c__Gammaproteobacteria.o__Enterobacteriales.f__Enterobacteriaceae.g__Escherichia.s__coli.t__E110019
0.940
d__Bacteria.p__Proteobacteria.c__Gammaproteobacteria.o__Enterobacteriales.f__Enterobacteriaceae.g__Escherichia.s__coli.t__MS_69_1
d__Bacteria.p__Proteobacteria.c__Gammaproteobacteria.o__Enterobacteriales.f__Enterobacteriaceae.g__Escherichia.s__coli.t__HS
0.932
d__Bacteria.p__Proteobacteria.c__Gammaproteobacteria.o__Enterobacteriales.f__Enterobacteriaceae.g__Escherichia.s__coli.t__IAI1
d__Bacteria.p__Proteobacteria.c__Gammaproteobacteria.o__Enterobacteriales.f__Enterobacteriaceae.g__Escherichia.s__coli.t__BW2952
d__Bacteria.p__Proteobacteria.c__Gammaproteobacteria.o__Enterobacteriales.f__Enterobacteriaceae.g__Escherichia.s__coli.t__str__K12_substr__DH10B
0.936
d__Bacteria.p__Proteobacteria.c__Gammaproteobacteria.o__Enterobacteriales.f__Enterobacteriaceae.g__Escherichia.s__coli.t__W3110
d__Bacteria.p__Proteobacteria.c__Gammaproteobacteria.o__Enterobacteriales.f__Enterobacteriaceae.g__Escherichia.s__coli.t__DH1
0.813
d__Bacteria.p__Proteobacteria.c__Gammaproteobacteria.o__Enterobacteriales.f__Enterobacteriaceae.g__Escherichia.s__coli.t__DH1
1.000
d__Bacteria.p__Proteobacteria.c__Gammaproteobacteria.o__Enterobacteriales.f__Enterobacteriaceae.g__Escherichia.s__coli.t__str__K_12_substr__MG1655
d__Bacteria.p__Proteobacteria.c__Gammaproteobacteria.o__Enterobacteriales.f__Enterobacteriaceae.g__Shigella.s__sp_.t__D9
d__Bacteria.p__Proteobacteria.c__Gammaproteobacteria.o__Enterobacteriales.f__Enterobacteriaceae.g__Escherichia.s__coli.t__H591
1.000
d__Bacteria.p__Proteobacteria.c__Gammaproteobacteria.o__Enterobacteriales.f__Enterobacteriaceae.g__Escherichia.s__coli.t__TA271
0.996
d__Bacteria.p__Proteobacteria.c__Gammaproteobacteria.o__Enterobacteriales.f__Enterobacteriaceae.g__Escherichia.s__coli.t__UMNF18
d__Bacteria.p__Proteobacteria.c__Gammaproteobacteria.o__Enterobacteriales.f__Enterobacteriaceae.g__Escherichia.s__coli.t__ATCC_8739
0.868
d__Bacteria.p__Proteobacteria.c__Gammaproteobacteria.o__Enterobacteriales.f__Enterobacteriaceae.g__Escherichia.s__coli.t__ETEC_H10407
d__Bacteria.p__Proteobacteria.c__Gammaproteobacteria.o__Enterobacteriales.f__Enterobacteriaceae.g__Escherichia.s__coli.t__B088
0.768
d__Bacteria.p__Proteobacteria.c__Gammaproteobacteria.o__Enterobacteriales.f__Enterobacteriaceae.g__Escherichia.s__coli.t__55989
d__Bacteria.p__Proteobacteria.c__Gammaproteobacteria.o__Enterobacteriales.f__Enterobacteriaceae.g__Escherichia.s__coli.t__O55_H7_str__CB9615
d__Bacteria.p__Proteobacteria.c__Gammaproteobacteria.o__Enterobacteriales.f__Enterobacteriaceae.g__Escherichia.s__coli.t__O157_H7_str__EC4115
d__Bacteria.p__Proteobacteria.c__Gammaproteobacteria.o__Enterobacteriales.f__Enterobacteriaceae.g__Escherichia.s__coli.t__O157_H7_str__EC4024
0.956
d__Bacteria.p__Proteobacteria.c__Gammaproteobacteria.o__Enterobacteriales.f__Enterobacteriaceae.g__Escherichia.s__coli.t__O157_H7_str__TW14359
1.000
d__Bacteria.p__Proteobacteria.c__Gammaproteobacteria.o__Enterobacteriales.f__Enterobacteriaceae.g__Escherichia.s__coli.t__O157_H7_str__TW14588
0.755
d__Bacteria.p__Proteobacteria.c__Gammaproteobacteria.o__Enterobacteriales.f__Enterobacteriaceae.g__Escherichia.s__coli.t__O157_H7_str__Sakai
0.984
d__Bacteria.p__Proteobacteria.c__Gammaproteobacteria.o__Enterobacteriales.f__Enterobacteriaceae.g__Escherichia.s__coli.t__O157_H7_EDL933
d__Bacteria.p__Proteobacteria.c__Gammaproteobacteria.o__Enterobacteriales.f__Enterobacteriaceae.g__Escherichia.s__coli.t__O157_H7_str__FRIK2000
1.000
d__Bacteria.p__Proteobacteria.c__Gammaproteobacteria.o__Enterobacteriales.f__Enterobacteriaceae.g__Escherichia.s__coli.t__O157_H7_str__FRIK966
d__Bacteria.p__Proteobacteria.c__Gammaproteobacteria.o__Enterobacteriales.f__Enterobacteriaceae.g__Escherichia.s__coli.t__O103_H2_str__12009
0.962
d__Bacteria.p__Proteobacteria.c__Gammaproteobacteria.o__Enterobacteriales.f__Enterobacteriaceae.g__Escherichia.s__coli.t__O26_H11_str__11368
d__Bacteria.p__Proteobacteria.c__Gammaproteobacteria.o__Enterobacteriales.f__Enterobacteriaceae.g__Escherichia.s__coli.t__O111_H__str__11128
1.000
d__Bacteria.p__Proteobacteria.c__Gammaproteobacteria.o__Enterobacteriales.f__Enterobacteriaceae.g__Escherichia.s__coli.t__W
0.998
d__Bacteria.p__Proteobacteria.c__Gammaproteobacteria.o__Enterobacteriales.f__Enterobacteriaceae.g__Escherichia.s__coli.t__E24377A
d__Bacteria.p__Proteobacteria.c__Gammaproteobacteria.o__Enterobacteriales.f__Enterobacteriaceae.g__Escherichia.s__coli.t__UMNK88
0.811
d__Bacteria.p__Proteobacteria.c__Gammaproteobacteria.o__Enterobacteriales.f__Enterobacteriaceae.g__Escherichia.s__coli.t__W
d__Bacteria.p__Proteobacteria.c__Gammaproteobacteria.o__Enterobacteriales.f__Enterobacteriaceae.g__Escherichia.s__coli.t__SE11
d__Bacteria.p__Proteobacteria.c__Gammaproteobacteria.o__Enterobacteriales.f__Enterobacteriaceae.g__Escherichia.s__coli.t__1827_70
0.957
d__Bacteria.p__Proteobacteria.c__Gammaproteobacteria.o__Enterobacteriales.f__Enterobacteriaceae.g__Escherichia.s__coli.t__E22
d__Bacteria.p__Proteobacteria.c__Gammaproteobacteria.o__Enterobacteriales.f__Enterobacteriaceae.g__Escherichia.s__coli.t__B171
d__Bacteria.p__Proteobacteria.c__Gammaproteobacteria.o__Enterobacteriales.f__Enterobacteriaceae.g__Escherichia.s__coli.t__101_1
d__Bacteria.p__Proteobacteria.c__Gammaproteobacteria.o__Enterobacteriales.f__Enterobacteriaceae.g__Escherichia.s__coli.t__53638
1.000
d__Bacteria.p__Proteobacteria.c__Gammaproteobacteria.o__Enterobacteriales.f__Enterobacteriaceae.g__Escherichia.s__coli.t__B7A
d__Bacteria.p__Proteobacteria.c__Gammaproteobacteria.o__Enterobacteriales.f__Enterobacteriaceae.g__Escherichia.s__coli.t__SMS_3_5
d__Bacteria.p__Proteobacteria.c__Gammaproteobacteria.o__Enterobacteriales.f__Enterobacteriaceae.g__Escherichia.s__coli.t__TA143
1.000
d__Bacteria.p__Proteobacteria.c__Gammaproteobacteria.o__Enterobacteriales.f__Enterobacteriaceae.g__Escherichia.s__coli.t__B354
d__Bacteria.p__Proteobacteria.c__Gammaproteobacteria.o__Enterobacteriales.f__Enterobacteriaceae.g__Escherichia.s__coli.t__042
0.981
d__Bacteria.p__Proteobacteria.c__Gammaproteobacteria.o__Enterobacteriales.f__Enterobacteriaceae.g__Escherichia.s__coli.t__UMN026
0.950
d__Bacteria.p__Proteobacteria.c__Gammaproteobacteria.o__Enterobacteriales.f__Enterobacteriaceae.g__Escherichia.s__coli.t__FVEC1302
0.930
d__Bacteria.p__Proteobacteria.c__Gammaproteobacteria.o__Enterobacteriales.f__Enterobacteriaceae.g__Escherichia.s__coli.t__FVEC1412
d__Bacteria.p__Proteobacteria.c__Gammaproteobacteria.o__Enterobacteriales.f__Enterobacteriaceae.g__Shigella.s__flexneri.t__2a_str__301
0.999
d__Bacteria.p__Proteobacteria.c__Gammaproteobacteria.o__Enterobacteriales.f__Enterobacteriaceae.g__Shigella.s__flexneri.t__2002017
1.000
d__Bacteria.p__Proteobacteria.c__Gammaproteobacteria.o__Enterobacteriales.f__Enterobacteriaceae.g__Shigella.s__flexneri.t__5_str__8401
d__Bacteria.p__Proteobacteria.c__Gammaproteobacteria.o__Enterobacteriales.f__Enterobacteriaceae.g__Shigella.s__flexneri.t__2a_str__2457T
d__Bacteria.p__Proteobacteria.c__Gammaproteobacteria.o__Enterobacteriales.f__Enterobacteriaceae.g__Shigella.s__sonnei.t__Ss046
1.000
d__Bacteria.p__Proteobacteria.c__Gammaproteobacteria.o__Enterobacteriales.f__Enterobacteriaceae.g__Shigella.s__dysenteriae.t__1012
1.000
d__Bacteria.p__Proteobacteria.c__Gammaproteobacteria.o__Enterobacteriales.f__Enterobacteriaceae.g__Shigella.s__boydii.t__CDC_3083_94
0.283
d__Bacteria.p__Proteobacteria.c__Gammaproteobacteria.o__Enterobacteriales.f__Enterobacteriaceae.g__Shigella.s__boydii.t__Sb227
0.999
d__Bacteria.p__Proteobacteria.c__Gammaproteobacteria.o__Enterobacteriales.f__Enterobacteriaceae.g__Shigella.s__dysenteriae.t__1617
1.000
d__Bacteria.p__Proteobacteria.c__Gammaproteobacteria.o__Enterobacteriales.f__Enterobacteriaceae.g__Shigella.s__dysenteriae.t__Sd197
d__Bacteria.p__Proteobacteria.c__Gammaproteobacteria.o__Enterobacteriales.f__Enterobacteriaceae.g__Escherichia.s__albertii.t__TW07627
d__Bacteria.p__Proteobacteria.c__Gammaproteobacteria.o__Enterobacteriales.f__Enterobacteriaceae.g__Enterobacter.s__aerogenes.t__KCTC_2190
d__Bacteria.p__Proteobacteria.c__Gammaproteobacteria.o__Enterobacteriales.f__Enterobacteriaceae.g__Klebsiella.s__sp_.t__MS_92_3
0.922
d__Bacteria.p__Proteobacteria.c__Gammaproteobacteria.o__Enterobacteriales.f__Enterobacteriaceae.g__Klebsiella.s__pneumoniae.t__subsp__rhinoscleromatis_ATCC_13884
d__Bacteria.p__Proteobacteria.c__Gammaproteobacteria.o__Enterobacteriales.f__Enterobacteriaceae.g__Klebsiella.s__pneumoniae.t__subsp__pneumoniae_MGH_78578
1.000
d__Bacteria.p__Proteobacteria.c__Gammaproteobacteria.o__Enterobacteriales.f__Enterobacteriaceae.g__Klebsiella.s__pneumoniae.t__NTUH_K2044
1.000
d__Bacteria.p__Proteobacteria.c__Gammaproteobacteria.o__Enterobacteriales.f__Enterobacteriaceae.g__Klebsiella.s__pneumoniae.t__KCTC_2242
0.972
d__Bacteria.p__Proteobacteria.c__Gammaproteobacteria.o__Enterobacteriales.f__Enterobacteriaceae.g__Klebsiella.s__variicola.t__At_22
d__Bacteria.p__Proteobacteria.c__Gammaproteobacteria.o__Enterobacteriales.f__Enterobacteriaceae.g__Klebsiella.s__sp_.t__1_1_55
0.696
d__Bacteria.p__Proteobacteria.c__Gammaproteobacteria.o__Enterobacteriales.f__Enterobacteriaceae.g__Klebsiella.s__pneumoniae.t__342
d__Bacteria.p__Proteobacteria.c__Gammaproteobacteria.o__Enterobacteriales.f__Enterobacteriaceae.g__Enterobacter.s__cloacae.t__SCF1
d__Bacteria.p__Proteobacteria.c__Gammaproteobacteria.o__Enterobacteriales.f__Enterobacteriaceae.g__Enterobacter.s__sp_.t__638
d__Bacteria.p__Proteobacteria.c__Gammaproteobacteria.o__Enterobacteriales.f__Enterobacteriaceae.g__Enterobacter.s__cancerogenus.t__ATCC_35316
d__Bacteria.p__Proteobacteria.c__Gammaproteobacteria.o__Enterobacteriales.f__Enterobacteriaceae.g__Enterobacter.s__cloacae.t__subsp__cloacae_ATCC_13047
1.000
d__Bacteria.p__Proteobacteria.c__Gammaproteobacteria.o__Enterobacteriales.f__Enterobacteriaceae.g__Enterobacter.s__hormaechei.t__ATCC_49162
1.000
d__Bacteria.p__Proteobacteria.c__Gammaproteobacteria.o__Enterobacteriales.f__Enterobacteriaceae.g__Enterobacter.s__cloacae.t__subsp__cloacae_NCTC_9394
d__Bacteria.p__Proteobacteria.c__Gammaproteobacteria.o__Enterobacteriales.f__Enterobacteriaceae.g__Cronobacter.s__sakazakii.t__ATCC_BAA_894
1.000
d__Bacteria.p__Proteobacteria.c__Gammaproteobacteria.o__Enterobacteriales.f__Enterobacteriaceae.g__Cronobacter.s__turicensis.t___1
0.849
d__Bacteria.p__Proteobacteria.c__Gammaproteobacteria.o__Enterobacteriales.f__Enterobacteriaceae.g__Sodalis.s__glossinidius.t__str___morsitans_
d__Bacteria.p__Proteobacteria.c__Gammaproteobacteria.o__Enterobacteriales.f__Enterobacteriaceae.g__Baumannia.s__cicadellinicola.t__str__Hc__Homalodisca_coagulata_
d__Bacteria.p__Proteobacteria.c__Gammaproteobacteria.o__Enterobacteriales.f__Enterobacteriaceae.g__Buchnera.s__aphidicola.t__str__Sg__Schizaphis_graminum_
d__Bacteria.p__Proteobacteria.c__Gammaproteobacteria.o__Enterobacteriales.f__Enterobacteriaceae.g__Buchnera.s__aphidicola.t__str__5A__Acyrthosiphon_pisum_
0.895
d__Bacteria.p__Proteobacteria.c__Gammaproteobacteria.o__Enterobacteriales.f__Enterobacteriaceae.g__Buchnera.s__aphidicola.t__str__LSR1__Acyrthosiphon_pisum_
1.000
d__Bacteria.p__Proteobacteria.c__Gammaproteobacteria.o__Enterobacteriales.f__Enterobacteriaceae.g__Buchnera.s__aphidicola.t__str__LL01__Acyrthosiphon_pisum_
d__Bacteria.p__Proteobacteria.c__Gammaproteobacteria.o__Enterobacteriales.f__Enterobacteriaceae.g__Buchnera.s__aphidicola.t__str__Tuc7__Acyrthosiphon_pisum_
0.845
1.000
d__Bacteria.p__Proteobacteria.c__Gammaproteobacteria.o__Enterobacteriales.f__Enterobacteriaceae.g__Buchnera.s__aphidicola.t__str__TLW03__Acyrthosiphon_pisum_
1.000
d__Bacteria.p__Proteobacteria.c__Gammaproteobacteria.o__Enterobacteriales.f__Enterobacteriaceae.g__Buchnera.s__aphidicola.t__str__JF99__Acyrthosiphon_pisum_
1.000
d__Bacteria.p__Proteobacteria.c__Gammaproteobacteria.o__Enterobacteriales.f__Enterobacteriaceae.g__Buchnera.s__aphidicola.t__str__JF98__Acyrthosiphon_pisum_
d__Bacteria.p__Proteobacteria.c__Gammaproteobacteria.o__Enterobacteriales.f__Enterobacteriaceae.g__Buchnera.s__aphidicola.t__str__APS__Acyrthosiphon_pisum_
d__Bacteria.p__Proteobacteria.c__Gammaproteobacteria.o__Enterobacteriales.f__Enterobacteriaceae.g__Buchnera.s__aphidicola.t__str__Bp__Baizongia_pistaciae_
1.000
0.996
d__Bacteria.p__Proteobacteria.c__Gammaproteobacteria.o__Enterobacteriales.f__Enterobacteriaceae.g__Buchnera.s__aphidicola.t___Cinara_tujafilina_
1.000
d__Bacteria.p__Proteobacteria.c__Gammaproteobacteria.o__Enterobacteriales.f__Enterobacteriaceae.g__Buchnera.s__aphidicola.t__str__Cc__Cinara_cedri_
1.000
1.000
d__Bacteria.p__Proteobacteria.c__Gammaproteobacteria.o__Enterobacteriales.f__Enterobacteriaceae.g__Wigglesworthia.s__glossinidia.t__endosymbiont_of_Glossina_brevipalpis
0.620
d__Bacteria.p__Proteobacteria.c__Gammaproteobacteria.o__Enterobacteriales.f__Enterobacteriaceae.g__Candidatus_Riesia.s__Riesia.t__pediculicola_USDA
d__Bacteria.p__Proteobacteria.c__Gammaproteobacteria.o__Enterobacteriales.f__Enterobacteriaceae.g__Candidatus_Blochmannia.s__pennsylvanicus_str__BPEN.t__pennsylvanicus_str__BPEN
1.000
d__Bacteria.p__Proteobacteria.c__Gammaproteobacteria.o__Enterobacteriales.f__Enterobacteriaceae.g__Candidatus_Blochmannia.s__Blochmannia.t__vafer_str__BVAF
1.000
d__Bacteria.p__Proteobacteria.c__Gammaproteobacteria.o__Enterobacteriales.f__Enterobacteriaceae.g__Candidatus_Blochmannia.s__floridanus.t__floridanus
d__Bacteria.p__Proteobacteria.c__Gammaproteobacteria.o__Enterobacteriales.f__Enterobacteriaceae.g__Candidatus_Moranella.s___1.t__endobia_PCIT
1.000
d__Bacteria.p__Proteobacteria.c__Gammaproteobacteria.o__Enterobacteriales.f__Enterobacteriaceae.g__unclassified.s__bacterium.t__9_2_54FAA
1.000
d__Bacteria.p__Proteobacteria.c__Gammaproteobacteria.o__Enterobacteriales.f__Enterobacteriaceae.g__Edwardsiella.s__tarda.t__ATCC_23685
d__Bacteria.p__Proteobacteria.c__Gammaproteobacteria.o__Enterobacteriales.f__Enterobacteriaceae.g__Edwardsiella.s__ictaluri.t__93_146
d__Bacteria.p__Proteobacteria.c__Gammaproteobacteria.o__Enterobacteriales.f__Enterobacteriaceae.g__Edwardsiella.s__tarda.t__EIB202
1.000
d__Bacteria.p__Proteobacteria.c__Gammaproteobacteria.o__Enterobacteriales.f__Enterobacteriaceae.g__Edwardsiella.s__tarda.t__FL6_60
1.000
d__Bacteria.p__Proteobacteria.c__Gammaproteobacteria.o__Enterobacteriales.f__Enterobacteriaceae.g__Rahnella.s__sp_.t__Y9602
d__Bacteria.p__Proteobacteria.c__Gammaproteobacteria.o__Enterobacteriales.f__Enterobacteriaceae.g__Serratia.s__symbiotica.t__str__Tucson
d__Bacteria.p__Proteobacteria.c__Gammaproteobacteria.o__Enterobacteriales.f__Enterobacteriaceae.g__Serratia.s__odorifera.t__DSM_4582
d__Bacteria.p__Proteobacteria.c__Gammaproteobacteria.o__Enterobacteriales.f__Enterobacteriaceae.g__Serratia.s__odorifera.t__4Rx13
1.000
d__Bacteria.p__Proteobacteria.c__Gammaproteobacteria.o__Enterobacteriales.f__Enterobacteriaceae.g__Serratia.s__proteamaculans.t__568
d__Bacteria.p__Proteobacteria.c__Gammaproteobacteria.o__Enterobacteriales.f__Enterobacteriaceae.g__Candidatus_Regiella.s__Regiella.t__insecticola_LSR1
1.000
d__Bacteria.p__Proteobacteria.c__Gammaproteobacteria.o__Enterobacteriales.f__Enterobacteriaceae.g__Candidatus_Hamiltonella.s__Hamiltonella.t__defensa_5AT__Acyrthosiphon_pisum_

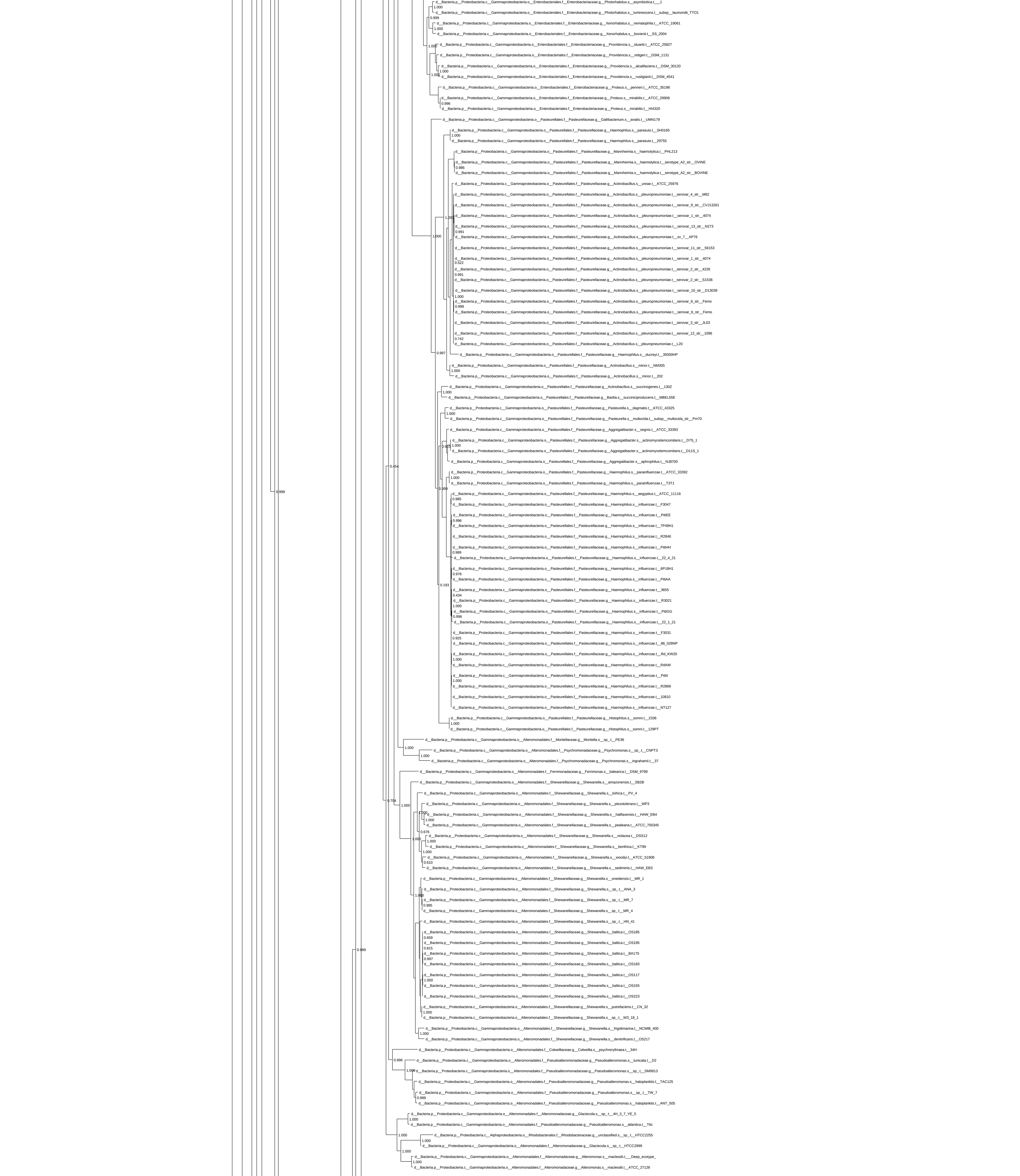

d__Bacteria.p__Proteobacteria.c__Gammaproteobacteria.o__Enterobacteriales.f__Enterobacteriaceae.g__Photorhabdus.s__asymbiotica.t___1
1.000
d__Bacteria.p__Proteobacteria.c__Gammaproteobacteria.o__Enterobacteriales.f__Enterobacteriaceae.g__Photorhabdus.s__luminescens.t__subsp__laumondii_TTO1
0.999
d__Bacteria.p__Proteobacteria.c__Gammaproteobacteria.o__Enterobacteriales.f__Enterobacteriaceae.g__Xenorhabdus.s__nematophila.t__ATCC_19061
1.000
d__Bacteria.p__Proteobacteria.c__Gammaproteobacteria.o__Enterobacteriales.f__Enterobacteriaceae.g__Xenorhabdus.s__bovienii.t__SS_2004
1.000
d__Bacteria.p__Proteobacteria.c__Gammaproteobacteria.o__Enterobacteriales.f__Enterobacteriaceae.g__Providencia.s__stuartii.t__ATCC_25827
d__Bacteria.p__Proteobacteria.c__Gammaproteobacteria.o__Enterobacteriales.f__Enterobacteriaceae.g__Providencia.s__rettgeri.t__DSM_1131
d__Bacteria.p__Proteobacteria.c__Gammaproteobacteria.o__Enterobacteriales.f__Enterobacteriaceae.g__Providencia.s__alcalifaciens.t__DSM_30120
1.000
1.000
d__Bacteria.p__Proteobacteria.c__Gammaproteobacteria.o__Enterobacteriales.f__Enterobacteriaceae.g__Providencia.s__rustigianii.t__DSM_4541
d__Bacteria.p__Proteobacteria.c__Gammaproteobacteria.o__Enterobacteriales.f__Enterobacteriaceae.g__Proteus.s__penneri.t__ATCC_35198
d__Bacteria.p__Proteobacteria.c__Gammaproteobacteria.o__Enterobacteriales.f__Enterobacteriaceae.g__Proteus.s__mirabilis.t__ATCC_29906
0.996
d__Bacteria.p__Proteobacteria.c__Gammaproteobacteria.o__Enterobacteriales.f__Enterobacteriaceae.g__Proteus.s__mirabilis.t__HI4320
d__Bacteria.p__Proteobacteria.c__Gammaproteobacteria.o__Pasteurellales.f__Pasteurellaceae.g__Gallibacterium.s__anatis.t__UMN179
d__Bacteria.p__Proteobacteria.c__Gammaproteobacteria.o__Pasteurellales.f__Pasteurellaceae.g__Haemophilus.s__parasuis.t__SH0165
1.000
d__Bacteria.p__Proteobacteria.c__Gammaproteobacteria.o__Pasteurellales.f__Pasteurellaceae.g__Haemophilus.s__parasuis.t__29755
d__Bacteria.p__Proteobacteria.c__Gammaproteobacteria.o__Pasteurellales.f__Pasteurellaceae.g__Mannheimia.s__haemolytica.t__PHL213
d__Bacteria.p__Proteobacteria.c__Gammaproteobacteria.o__Pasteurellales.f__Pasteurellaceae.g__Mannheimia.s__haemolytica.t__serotype_A2_str__OVINE
0.986
d__Bacteria.p__Proteobacteria.c__Gammaproteobacteria.o__Pasteurellales.f__Pasteurellaceae.g__Mannheimia.s__haemolytica.t__serotype_A2_str__BOVINE
d__Bacteria.p__Proteobacteria.c__Gammaproteobacteria.o__Pasteurellales.f__Pasteurellaceae.g__Actinobacillus.s__ureae.t__ATCC_25976
d__Bacteria.p__Proteobacteria.c__Gammaproteobacteria.o__Pasteurellales.f__Pasteurellaceae.g__Actinobacillus.s__pleuropneumoniae.t__serovar_4_str__M62
d__Bacteria.p__Proteobacteria.c__Gammaproteobacteria.o__Pasteurellales.f__Pasteurellaceae.g__Actinobacillus.s__pleuropneumoniae.t__serovar_9_str__CVJ13261
1.000
d__Bacteria.p__Proteobacteria.c__Gammaproteobacteria.o__Pasteurellales.f__Pasteurellaceae.g__Actinobacillus.s__pleuropneumoniae.t__serovar_1_str__4074
d__Bacteria.p__Proteobacteria.c__Gammaproteobacteria.o__Pasteurellales.f__Pasteurellaceae.g__Actinobacillus.s__pleuropneumoniae.t__serovar_13_str__N273
0.991
1.000
d__Bacteria.p__Proteobacteria.c__Gammaproteobacteria.o__Pasteurellales.f__Pasteurellaceae.g__Actinobacillus.s__pleuropneumoniae.t__sv_7__AP76
d__Bacteria.p__Proteobacteria.c__Gammaproteobacteria.o__Pasteurellales.f__Pasteurellaceae.g__Actinobacillus.s__pleuropneumoniae.t__serovar_11_str__56153
d__Bacteria.p__Proteobacteria.c__Gammaproteobacteria.o__Pasteurellales.f__Pasteurellaceae.g__Actinobacillus.s__pleuropneumoniae.t__serovar_1_str__4074
0.522
d__Bacteria.p__Proteobacteria.c__Gammaproteobacteria.o__Pasteurellales.f__Pasteurellaceae.g__Actinobacillus.s__pleuropneumoniae.t__serovar_2_str__4226
0.991
d__Bacteria.p__Proteobacteria.c__Gammaproteobacteria.o__Pasteurellales.f__Pasteurellaceae.g__Actinobacillus.s__pleuropneumoniae.t__serovar_2_str__S1536
d__Bacteria.p__Proteobacteria.c__Gammaproteobacteria.o__Pasteurellales.f__Pasteurellaceae.g__Actinobacillus.s__pleuropneumoniae.t__serovar_10_str__D13039
1.000
d__Bacteria.p__Proteobacteria.c__Gammaproteobacteria.o__Pasteurellales.f__Pasteurellaceae.g__Actinobacillus.s__pleuropneumoniae.t__serovar_6_str__Femo
0.999
d__Bacteria.p__Proteobacteria.c__Gammaproteobacteria.o__Pasteurellales.f__Pasteurellaceae.g__Actinobacillus.s__pleuropneumoniae.t__serovar_6_str__Femo
d__Bacteria.p__Proteobacteria.c__Gammaproteobacteria.o__Pasteurellales.f__Pasteurellaceae.g__Actinobacillus.s__pleuropneumoniae.t__serovar_3_str__JL03
d__Bacteria.p__Proteobacteria.c__Gammaproteobacteria.o__Pasteurellales.f__Pasteurellaceae.g__Actinobacillus.s__pleuropneumoniae.t__serovar_12_str__1096
0.742
d__Bacteria.p__Proteobacteria.c__Gammaproteobacteria.o__Pasteurellales.f__Pasteurellaceae.g__Actinobacillus.s__pleuropneumoniae.t__L20
d__Bacteria.p__Proteobacteria.c__Gammaproteobacteria.o__Pasteurellales.f__Pasteurellaceae.g__Haemophilus.s__ducreyi.t__35000HP
0.997
d__Bacteria.p__Proteobacteria.c__Gammaproteobacteria.o__Pasteurellales.f__Pasteurellaceae.g__Actinobacillus.s__minor.t__NM305
1.000
d__Bacteria.p__Proteobacteria.c__Gammaproteobacteria.o__Pasteurellales.f__Pasteurellaceae.g__Actinobacillus.s__minor.t__202
d__Bacteria.p__Proteobacteria.c__Gammaproteobacteria.o__Pasteurellales.f__Pasteurellaceae.g__Actinobacillus.s__succinogenes.t__130Z
1.000
d__Bacteria.p__Proteobacteria.c__Gammaproteobacteria.o__Pasteurellales.f__Pasteurellaceae.g__Basfia.s__succiniciproducens.t__MBEL55E
d__Bacteria.p__Proteobacteria.c__Gammaproteobacteria.o__Pasteurellales.f__Pasteurellaceae.g__Pasteurella.s__dagmatis.t__ATCC_43325
1.000
d__Bacteria.p__Proteobacteria.c__Gammaproteobacteria.o__Pasteurellales.f__Pasteurellaceae.g__Pasteurella.s__multocida.t__subsp__multocida_str__Pm70
d__Bacteria.p__Proteobacteria.c__Gammaproteobacteria.o__Pasteurellales.f__Pasteurellaceae.g__Aggregatibacter.s__segnis.t__ATCC_33393
d__Bacteria.p__Proteobacteria.c__Gammaproteobacteria.o__Pasteurellales.f__Pasteurellaceae.g__Aggregatibacter.s__actinomycetemcomitans.t__D7S_1
1.000
d__Bacteria.p__Proteobacteria.c__Gammaproteobacteria.o__Pasteurellales.f__Pasteurellaceae.g__Aggregatibacter.s__actinomycetemcomitans.t__D11S_1
d__Bacteria.p__Proteobacteria.c__Gammaproteobacteria.o__Pasteurellales.f__Pasteurellaceae.g__Aggregatibacter.s__aphrophilus.t__NJ8700
0.454
d__Bacteria.p__Proteobacteria.c__Gammaproteobacteria.o__Pasteurellales.f__Pasteurellaceae.g__Haemophilus.s__parainfluenzae.t__ATCC_33392
1.000
d__Bacteria.p__Proteobacteria.c__Gammaproteobacteria.o__Pasteurellales.f__Pasteurellaceae.g__Haemophilus.s__parainfluenzae.t__T3T1
0.999
0.999
d__Bacteria.p__Proteobacteria.c__Gammaproteobacteria.o__Pasteurellales.f__Pasteurellaceae.g__Haemophilus.s__aegyptius.t__ATCC_11116
0.985
d__Bacteria.p__Proteobacteria.c__Gammaproteobacteria.o__Pasteurellales.f__Pasteurellaceae.g__Haemophilus.s__influenzae.t__F3047
d__Bacteria.p__Proteobacteria.c__Gammaproteobacteria.o__Pasteurellales.f__Pasteurellaceae.g__Haemophilus.s__influenzae.t__PittEE
0.996
d__Bacteria.p__Proteobacteria.c__Gammaproteobacteria.o__Pasteurellales.f__Pasteurellaceae.g__Haemophilus.s__influenzae.t__7P49H1
d__Bacteria.p__Proteobacteria.c__Gammaproteobacteria.o__Pasteurellales.f__Pasteurellaceae.g__Haemophilus.s__influenzae.t__R2846
d__Bacteria.p__Proteobacteria.c__Gammaproteobacteria.o__Pasteurellales.f__Pasteurellaceae.g__Haemophilus.s__influenzae.t__PittHH
0.989
d__Bacteria.p__Proteobacteria.c__Gammaproteobacteria.o__Pasteurellales.f__Pasteurellaceae.g__Haemophilus.s__influenzae.t__22_4_21
d__Bacteria.p__Proteobacteria.c__Gammaproteobacteria.o__Pasteurellales.f__Pasteurellaceae.g__Haemophilus.s__influenzae.t__6P18H1
0.976
d__Bacteria.p__Proteobacteria.c__Gammaproteobacteria.o__Pasteurellales.f__Pasteurellaceae.g__Haemophilus.s__influenzae.t__PittAA
0.193
d__Bacteria.p__Proteobacteria.c__Gammaproteobacteria.o__Pasteurellales.f__Pasteurellaceae.g__Haemophilus.s__influenzae.t__3655
0.434
d__Bacteria.p__Proteobacteria.c__Gammaproteobacteria.o__Pasteurellales.f__Pasteurellaceae.g__Haemophilus.s__influenzae.t__R3021
1.000
d__Bacteria.p__Proteobacteria.c__Gammaproteobacteria.o__Pasteurellales.f__Pasteurellaceae.g__Haemophilus.s__influenzae.t__PittGG
0.996
d__Bacteria.p__Proteobacteria.c__Gammaproteobacteria.o__Pasteurellales.f__Pasteurellaceae.g__Haemophilus.s__influenzae.t__22_1_21
d__Bacteria.p__Proteobacteria.c__Gammaproteobacteria.o__Pasteurellales.f__Pasteurellaceae.g__Haemophilus.s__influenzae.t__F3031
0.925
d__Bacteria.p__Proteobacteria.c__Gammaproteobacteria.o__Pasteurellales.f__Pasteurellaceae.g__Haemophilus.s__influenzae.t__86_028NP
d__Bacteria.p__Proteobacteria.c__Gammaproteobacteria.o__Pasteurellales.f__Pasteurellaceae.g__Haemophilus.s__influenzae.t__Rd_KW20
1.000
d__Bacteria.p__Proteobacteria.c__Gammaproteobacteria.o__Pasteurellales.f__Pasteurellaceae.g__Haemophilus.s__influenzae.t__RdAW
d__Bacteria.p__Proteobacteria.c__Gammaproteobacteria.o__Pasteurellales.f__Pasteurellaceae.g__Haemophilus.s__influenzae.t__PittII
1.000
d__Bacteria.p__Proteobacteria.c__Gammaproteobacteria.o__Pasteurellales.f__Pasteurellaceae.g__Haemophilus.s__influenzae.t__R2866
d__Bacteria.p__Proteobacteria.c__Gammaproteobacteria.o__Pasteurellales.f__Pasteurellaceae.g__Haemophilus.s__influenzae.t__10810
d__Bacteria.p__Proteobacteria.c__Gammaproteobacteria.o__Pasteurellales.f__Pasteurellaceae.g__Haemophilus.s__influenzae.t__NT127
d__Bacteria.p__Proteobacteria.c__Gammaproteobacteria.o__Pasteurellales.f__Pasteurellaceae.g__Histophilus.s__somni.t__2336
1.000
d__Bacteria.p__Proteobacteria.c__Gammaproteobacteria.o__Pasteurellales.f__Pasteurellaceae.g__Histophilus.s__somni.t__129PT
d__Bacteria.p__Proteobacteria.c__Gammaproteobacteria.o__Alteromonadales.f__Moritellaceae.g__Moritella.s__sp_.t__PE36
1.000
d__Bacteria.p__Proteobacteria.c__Gammaproteobacteria.o__Alteromonadales.f__Psychromonadaceae.g__Psychromonas.s__sp_.t__CNPT3
1.000
d__Bacteria.p__Proteobacteria.c__Gammaproteobacteria.o__Alteromonadales.f__Psychromonadaceae.g__Psychromonas.s__ingrahamii.t__37
d__Bacteria.p__Proteobacteria.c__Gammaproteobacteria.o__Alteromonadales.f__Ferrimonadaceae.g__Ferrimonas.s__balearica.t__DSM_9799
d__Bacteria.p__Proteobacteria.c__Gammaproteobacteria.o__Alteromonadales.f__Shewanellaceae.g__Shewanella.s__amazonensis.t__SB2B
d__Bacteria.p__Proteobacteria.c__Gammaproteobacteria.o__Alteromonadales.f__Shewanellaceae.g__Shewanella.s__loihica.t__PV_4
0.704
1.000
d__Bacteria.p__Proteobacteria.c__Gammaproteobacteria.o__Alteromonadales.f__Shewanellaceae.g__Shewanella.s__piezotolerans.t__WP3
1.000
d__Bacteria.p__Proteobacteria.c__Gammaproteobacteria.o__Alteromonadales.f__Shewanellaceae.g__Shewanella.s__halifaxensis.t__HAW_EB4
1.000
d__Bacteria.p__Proteobacteria.c__Gammaproteobacteria.o__Alteromonadales.f__Shewanellaceae.g__Shewanella.s__pealeana.t__ATCC_700345
0.676
1.000
d__Bacteria.p__Proteobacteria.c__Gammaproteobacteria.o__Alteromonadales.f__Shewanellaceae.g__Shewanella.s__violacea.t__DSS12
1.000
d__Bacteria.p__Proteobacteria.c__Gammaproteobacteria.o__Alteromonadales.f__Shewanellaceae.g__Shewanella.s__benthica.t__KT99
1.000
d__Bacteria.p__Proteobacteria.c__Gammaproteobacteria.o__Alteromonadales.f__Shewanellaceae.g__Shewanella.s__woodyi.t__ATCC_51908
0.610
d__Bacteria.p__Proteobacteria.c__Gammaproteobacteria.o__Alteromonadales.f__Shewanellaceae.g__Shewanella.s__sediminis.t__HAW_EB3
d__Bacteria.p__Proteobacteria.c__Gammaproteobacteria.o__Alteromonadales.f__Shewanellaceae.g__Shewanella.s__oneidensis.t__MR_1
d__Bacteria.p__Proteobacteria.c__Gammaproteobacteria.o__Alteromonadales.f__Shewanellaceae.g__Shewanella.s__sp_.t__ANA_3
1.000
d__Bacteria.p__Proteobacteria.c__Gammaproteobacteria.o__Alteromonadales.f__Shewanellaceae.g__Shewanella.s__sp_.t__MR_7
0.985
d__Bacteria.p__Proteobacteria.c__Gammaproteobacteria.o__Alteromonadales.f__Shewanellaceae.g__Shewanella.s__sp_.t__MR_4
d__Bacteria.p__Proteobacteria.c__Gammaproteobacteria.o__Alteromonadales.f__Shewanellaceae.g__Shewanella.s__sp_.t__HN_41
d__Bacteria.p__Proteobacteria.c__Gammaproteobacteria.o__Alteromonadales.f__Shewanellaceae.g__Shewanella.s__baltica.t__OS185
0.659
d__Bacteria.p__Proteobacteria.c__Gammaproteobacteria.o__Alteromonadales.f__Shewanellaceae.g__Shewanella.s__baltica.t__OS195
0.815
0.989
d__Bacteria.p__Proteobacteria.c__Gammaproteobacteria.o__Alteromonadales.f__Shewanellaceae.g__Shewanella.s__baltica.t__BA175
0.997
d__Bacteria.p__Proteobacteria.c__Gammaproteobacteria.o__Alteromonadales.f__Shewanellaceae.g__Shewanella.s__baltica.t__OS183
d__Bacteria.p__Proteobacteria.c__Gammaproteobacteria.o__Alteromonadales.f__Shewanellaceae.g__Shewanella.s__baltica.t__OS117
1.000
d__Bacteria.p__Proteobacteria.c__Gammaproteobacteria.o__Alteromonadales.f__Shewanellaceae.g__Shewanella.s__baltica.t__OS155
d__Bacteria.p__Proteobacteria.c__Gammaproteobacteria.o__Alteromonadales.f__Shewanellaceae.g__Shewanella.s__baltica.t__OS223
d__Bacteria.p__Proteobacteria.c__Gammaproteobacteria.o__Alteromonadales.f__Shewanellaceae.g__Shewanella.s__putrefaciens.t__CN_32
1.000
d__Bacteria.p__Proteobacteria.c__Gammaproteobacteria.o__Alteromonadales.f__Shewanellaceae.g__Shewanella.s__sp_.t__W3_18_1
d__Bacteria.p__Proteobacteria.c__Gammaproteobacteria.o__Alteromonadales.f__Shewanellaceae.g__Shewanella.s__frigidimarina.t__NCIMB_400
1.000
d__Bacteria.p__Proteobacteria.c__Gammaproteobacteria.o__Alteromonadales.f__Shewanellaceae.g__Shewanella.s__denitrificans.t__OS217
d__Bacteria.p__Proteobacteria.c__Gammaproteobacteria.o__Alteromonadales.f__Colwelliaceae.g__Colwellia.s__psychrerythraea.t__34H
0.996
d__Bacteria.p__Proteobacteria.c__Gammaproteobacteria.o__Alteromonadales.f__Pseudoalteromonadaceae.g__Pseudoalteromonas.s__tunicata.t__D2
1.000
d__Bacteria.p__Proteobacteria.c__Gammaproteobacteria.o__Alteromonadales.f__Pseudoalteromonadaceae.g__Pseudoalteromonas.s__sp_.t__SM9913
d__Bacteria.p__Proteobacteria.c__Gammaproteobacteria.o__Alteromonadales.f__Pseudoalteromonadaceae.g__Pseudoalteromonas.s__haloplanktis.t__TAC125
d__Bacteria.p__Proteobacteria.c__Gammaproteobacteria.o__Alteromonadales.f__Pseudoalteromonadaceae.g__Pseudoalteromonas.s__sp_.t__TW_7
0.999
d__Bacteria.p__Proteobacteria.c__Gammaproteobacteria.o__Alteromonadales.f__Pseudoalteromonadaceae.g__Pseudoalteromonas.s__haloplanktis.t__ANT_505
d__Bacteria.p__Proteobacteria.c__Gammaproteobacteria.o__Alteromonadales.f__Alteromonadaceae.g__Glaciecola.s__sp_.t__4H_3_7_YE_5
1.000
d__Bacteria.p__Proteobacteria.c__Gammaproteobacteria.o__Alteromonadales.f__Pseudoalteromonadaceae.g__Pseudoalteromonas.s__atlantica.t__T6c
1.000
d__Bacteria.p__Proteobacteria.c__Alphaproteobacteria.o__Rhodobacterales.f__Rhodobacteraceae.g__unclassified.s__sp_.t__HTCC2255
1.000
d__Bacteria.p__Proteobacteria.c__Gammaproteobacteria.o__Alteromonadales.f__Alteromonadaceae.g__Glaciecola.s__sp_.t__HTCC2999
1.000
d__Bacteria.p__Proteobacteria.c__Gammaproteobacteria.o__Alteromonadales.f__Alteromonadaceae.g__Alteromonas.s__macleodii.t__Deep_ecotype_
1.000
d__Bacteria.p__Proteobacteria.c__Gammaproteobacteria.o__Alteromonadales.f__Alteromonadaceae.g__Alteromonas.s__macleodii.t__ATCC_27126

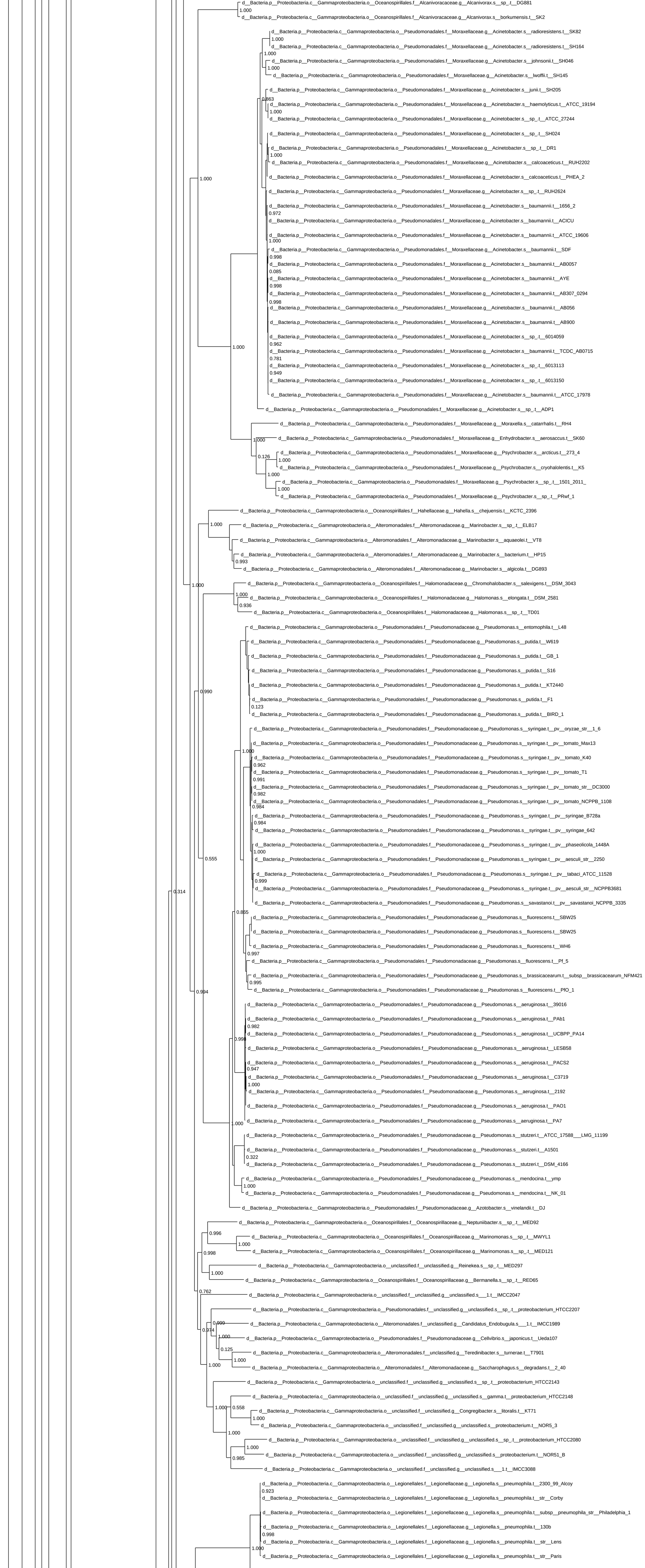

d__Bacteria.p__Proteobacteria.c__Gammaproteobacteria.o__Oceanospirillales.f__Alcanivoracaceae.g__Alcanivorax.s__sp_.t__DG881
1.000
d__Bacteria.p__Proteobacteria.c__Gammaproteobacteria.o__Oceanospirillales.f__Alcanivoracaceae.g__Alcanivorax.s__borkumensis.t__SK2
d__Bacteria.p__Proteobacteria.c__Gammaproteobacteria.o__Pseudomonadales.f__Moraxellaceae.g__Acinetobacter.s__radioresistens.t__SK82
1.000
d__Bacteria.p__Proteobacteria.c__Gammaproteobacteria.o__Pseudomonadales.f__Moraxellaceae.g__Acinetobacter.s__radioresistens.t__SH164
1.000
d__Bacteria.p__Proteobacteria.c__Gammaproteobacteria.o__Pseudomonadales.f__Moraxellaceae.g__Acinetobacter.s__johnsonii.t__SH046
1.000
d__Bacteria.p__Proteobacteria.c__Gammaproteobacteria.o__Pseudomonadales.f__Moraxellaceae.g__Acinetobacter.s__lwoffii.t__SH145
d__Bacteria.p__Proteobacteria.c__Gammaproteobacteria.o__Pseudomonadales.f__Moraxellaceae.g__Acinetobacter.s__junii.t__SH205
0.863
d__Bacteria.p__Proteobacteria.c__Gammaproteobacteria.o__Pseudomonadales.f__Moraxellaceae.g__Acinetobacter.s__haemolyticus.t__ATCC_19194
1.000
d__Bacteria.p__Proteobacteria.c__Gammaproteobacteria.o__Pseudomonadales.f__Moraxellaceae.g__Acinetobacter.s__sp_.t__ATCC_27244
d__Bacteria.p__Proteobacteria.c__Gammaproteobacteria.o__Pseudomonadales.f__Moraxellaceae.g__Acinetobacter.s__sp_.t__SH024
d__Bacteria.p__Proteobacteria.c__Gammaproteobacteria.o__Pseudomonadales.f__Moraxellaceae.g__Acinetobacter.s__sp_.t__DR1
1.000
d__Bacteria.p__Proteobacteria.c__Gammaproteobacteria.o__Pseudomonadales.f__Moraxellaceae.g__Acinetobacter.s__calcoaceticus.t__RUH2202
1.000
d__Bacteria.p__Proteobacteria.c__Gammaproteobacteria.o__Pseudomonadales.f__Moraxellaceae.g__Acinetobacter.s__calcoaceticus.t__PHEA_2
d__Bacteria.p__Proteobacteria.c__Gammaproteobacteria.o__Pseudomonadales.f__Moraxellaceae.g__Acinetobacter.s__sp_.t__RUH2624
d__Bacteria.p__Proteobacteria.c__Gammaproteobacteria.o__Pseudomonadales.f__Moraxellaceae.g__Acinetobacter.s__baumannii.t__1656_2
0.972
d__Bacteria.p__Proteobacteria.c__Gammaproteobacteria.o__Pseudomonadales.f__Moraxellaceae.g__Acinetobacter.s__baumannii.t__ACICU
d__Bacteria.p__Proteobacteria.c__Gammaproteobacteria.o__Pseudomonadales.f__Moraxellaceae.g__Acinetobacter.s__baumannii.t__ATCC_19606
1.000
d__Bacteria.p__Proteobacteria.c__Gammaproteobacteria.o__Pseudomonadales.f__Moraxellaceae.g__Acinetobacter.s__baumannii.t__SDF
0.998
d__Bacteria.p__Proteobacteria.c__Gammaproteobacteria.o__Pseudomonadales.f__Moraxellaceae.g__Acinetobacter.s__baumannii.t__AB0057
0.085
d__Bacteria.p__Proteobacteria.c__Gammaproteobacteria.o__Pseudomonadales.f__Moraxellaceae.g__Acinetobacter.s__baumannii.t__AYE
0.998
d__Bacteria.p__Proteobacteria.c__Gammaproteobacteria.o__Pseudomonadales.f__Moraxellaceae.g__Acinetobacter.s__baumannii.t__AB307_0294
0.998
d__Bacteria.p__Proteobacteria.c__Gammaproteobacteria.o__Pseudomonadales.f__Moraxellaceae.g__Acinetobacter.s__baumannii.t__AB056
d__Bacteria.p__Proteobacteria.c__Gammaproteobacteria.o__Pseudomonadales.f__Moraxellaceae.g__Acinetobacter.s__baumannii.t__AB900
d__Bacteria.p__Proteobacteria.c__Gammaproteobacteria.o__Pseudomonadales.f__Moraxellaceae.g__Acinetobacter.s__sp_.t__6014059
1.000
0.962
d__Bacteria.p__Proteobacteria.c__Gammaproteobacteria.o__Pseudomonadales.f__Moraxellaceae.g__Acinetobacter.s__baumannii.t__TCDC_AB0715
0.781
d__Bacteria.p__Proteobacteria.c__Gammaproteobacteria.o__Pseudomonadales.f__Moraxellaceae.g__Acinetobacter.s__sp_.t__6013113
0.949
d__Bacteria.p__Proteobacteria.c__Gammaproteobacteria.o__Pseudomonadales.f__Moraxellaceae.g__Acinetobacter.s__sp_.t__6013150
d__Bacteria.p__Proteobacteria.c__Gammaproteobacteria.o__Pseudomonadales.f__Moraxellaceae.g__Acinetobacter.s__baumannii.t__ATCC_17978
d__Bacteria.p__Proteobacteria.c__Gammaproteobacteria.o__Pseudomonadales.f__Moraxellaceae.g__Acinetobacter.s__sp_.t__ADP1
d__Bacteria.p__Proteobacteria.c__Gammaproteobacteria.o__Pseudomonadales.f__Moraxellaceae.g__Moraxella.s__catarrhalis.t__RH4
1.000
d__Bacteria.p__Proteobacteria.c__Gammaproteobacteria.o__Pseudomonadales.f__Moraxellaceae.g__Enhydrobacter.s__aerosaccus.t__SK60
d__Bacteria.p__Proteobacteria.c__Gammaproteobacteria.o__Pseudomonadales.f__Moraxellaceae.g__Psychrobacter.s__arcticus.t__273_4
0.126
1.000
d__Bacteria.p__Proteobacteria.c__Gammaproteobacteria.o__Pseudomonadales.f__Moraxellaceae.g__Psychrobacter.s__cryohalolentis.t__K5
1.000
d__Bacteria.p__Proteobacteria.c__Gammaproteobacteria.o__Pseudomonadales.f__Moraxellaceae.g__Psychrobacter.s__sp_.t__1501_2011_
1.000
d__Bacteria.p__Proteobacteria.c__Gammaproteobacteria.o__Pseudomonadales.f__Moraxellaceae.g__Psychrobacter.s__sp_.t__PRwf_1
d__Bacteria.p__Proteobacteria.c__Gammaproteobacteria.o__Oceanospirillales.f__Hahellaceae.g__Hahella.s__chejuensis.t__KCTC_2396
1.000
d__Bacteria.p__Proteobacteria.c__Gammaproteobacteria.o__Alteromonadales.f__Alteromonadaceae.g__Marinobacter.s__sp_.t__ELB17
d__Bacteria.p__Proteobacteria.c__Gammaproteobacteria.o__Alteromonadales.f__Alteromonadaceae.g__Marinobacter.s__aquaeolei.t__VT8
d__Bacteria.p__Proteobacteria.c__Gammaproteobacteria.o__Alteromonadales.f__Alteromonadaceae.g__Marinobacter.s__bacterium.t__HP15
0.993
d__Bacteria.p__Proteobacteria.c__Gammaproteobacteria.o__Alteromonadales.f__Alteromonadaceae.g__Marinobacter.s__algicola.t__DG893
1.000
d__Bacteria.p__Proteobacteria.c__Gammaproteobacteria.o__Oceanospirillales.f__Halomonadaceae.g__Chromohalobacter.s__salexigens.t__DSM_3043
1.000
d__Bacteria.p__Proteobacteria.c__Gammaproteobacteria.o__Oceanospirillales.f__Halomonadaceae.g__Halomonas.s__elongata.t__DSM_2581
0.936
d__Bacteria.p__Proteobacteria.c__Gammaproteobacteria.o__Oceanospirillales.f__Halomonadaceae.g__Halomonas.s__sp_.t__TD01
d__Bacteria.p__Proteobacteria.c__Gammaproteobacteria.o__Pseudomonadales.f__Pseudomonadaceae.g__Pseudomonas.s__entomophila.t__L48
d__Bacteria.p__Proteobacteria.c__Gammaproteobacteria.o__Pseudomonadales.f__Pseudomonadaceae.g__Pseudomonas.s__putida.t__W619
d__Bacteria.p__Proteobacteria.c__Gammaproteobacteria.o__Pseudomonadales.f__Pseudomonadaceae.g__Pseudomonas.s__putida.t__GB_1
d__Bacteria.p__Proteobacteria.c__Gammaproteobacteria.o__Pseudomonadales.f__Pseudomonadaceae.g__Pseudomonas.s__putida.t__S16
d__Bacteria.p__Proteobacteria.c__Gammaproteobacteria.o__Pseudomonadales.f__Pseudomonadaceae.g__Pseudomonas.s__putida.t__KT2440
0.990
d__Bacteria.p__Proteobacteria.c__Gammaproteobacteria.o__Pseudomonadales.f__Pseudomonadaceae.g__Pseudomonas.s__putida.t__F1
0.123
d__Bacteria.p__Proteobacteria.c__Gammaproteobacteria.o__Pseudomonadales.f__Pseudomonadaceae.g__Pseudomonas.s__putida.t__BIRD_1
d__Bacteria.p__Proteobacteria.c__Gammaproteobacteria.o__Pseudomonadales.f__Pseudomonadaceae.g__Pseudomonas.s__syringae.t__pv__oryzae_str__1_6
d__Bacteria.p__Proteobacteria.c__Gammaproteobacteria.o__Pseudomonadales.f__Pseudomonadaceae.g__Pseudomonas.s__syringae.t__pv__tomato_Max13
1.000
d__Bacteria.p__Proteobacteria.c__Gammaproteobacteria.o__Pseudomonadales.f__Pseudomonadaceae.g__Pseudomonas.s__syringae.t__pv__tomato_K40
0.962
d__Bacteria.p__Proteobacteria.c__Gammaproteobacteria.o__Pseudomonadales.f__Pseudomonadaceae.g__Pseudomonas.s__syringae.t__pv__tomato_T1
0.991
d__Bacteria.p__Proteobacteria.c__Gammaproteobacteria.o__Pseudomonadales.f__Pseudomonadaceae.g__Pseudomonas.s__syringae.t__pv__tomato_str__DC3000
0.982
d__Bacteria.p__Proteobacteria.c__Gammaproteobacteria.o__Pseudomonadales.f__Pseudomonadaceae.g__Pseudomonas.s__syringae.t__pv__tomato_NCPPB_1108
0.984
d__Bacteria.p__Proteobacteria.c__Gammaproteobacteria.o__Pseudomonadales.f__Pseudomonadaceae.g__Pseudomonas.s__syringae.t__pv__syringae_B728a
0.984
d__Bacteria.p__Proteobacteria.c__Gammaproteobacteria.o__Pseudomonadales.f__Pseudomonadaceae.g__Pseudomonas.s__syringae.t__pv__syringae_642
d__Bacteria.p__Proteobacteria.c__Gammaproteobacteria.o__Pseudomonadales.f__Pseudomonadaceae.g__Pseudomonas.s__syringae.t__pv__phaseolicola_1448A
1.000
0.555
d__Bacteria.p__Proteobacteria.c__Gammaproteobacteria.o__Pseudomonadales.f__Pseudomonadaceae.g__Pseudomonas.s__syringae.t__pv__aesculi_str__2250
d__Bacteria.p__Proteobacteria.c__Gammaproteobacteria.o__Pseudomonadales.f__Pseudomonadaceae.g__Pseudomonas.s__syringae.t__pv__tabaci_ATCC_11528
0.999
0.314
d__Bacteria.p__Proteobacteria.c__Gammaproteobacteria.o__Pseudomonadales.f__Pseudomonadaceae.g__Pseudomonas.s__syringae.t__pv__aesculi_str__NCPPB3681
d__Bacteria.p__Proteobacteria.c__Gammaproteobacteria.o__Pseudomonadales.f__Pseudomonadaceae.g__Pseudomonas.s__savastanoi.t__pv__savastanoi_NCPPB_3335
0.865
d__Bacteria.p__Proteobacteria.c__Gammaproteobacteria.o__Pseudomonadales.f__Pseudomonadaceae.g__Pseudomonas.s__fluorescens.t__SBW25
d__Bacteria.p__Proteobacteria.c__Gammaproteobacteria.o__Pseudomonadales.f__Pseudomonadaceae.g__Pseudomonas.s__fluorescens.t__SBW25
d__Bacteria.p__Proteobacteria.c__Gammaproteobacteria.o__Pseudomonadales.f__Pseudomonadaceae.g__Pseudomonas.s__fluorescens.t__WH6
0.997
d__Bacteria.p__Proteobacteria.c__Gammaproteobacteria.o__Pseudomonadales.f__Pseudomonadaceae.g__Pseudomonas.s__fluorescens.t__Pf_5
d__Bacteria.p__Proteobacteria.c__Gammaproteobacteria.o__Pseudomonadales.f__Pseudomonadaceae.g__Pseudomonas.s__brassicacearum.t__subsp__brassicacearum_NFM421
0.995
d__Bacteria.p__Proteobacteria.c__Gammaproteobacteria.o__Pseudomonadales.f__Pseudomonadaceae.g__Pseudomonas.s__fluorescens.t__PfO_1
0.994
d__Bacteria.p__Proteobacteria.c__Gammaproteobacteria.o__Pseudomonadales.f__Pseudomonadaceae.g__Pseudomonas.s__aeruginosa.t__39016
d__Bacteria.p__Proteobacteria.c__Gammaproteobacteria.o__Pseudomonadales.f__Pseudomonadaceae.g__Pseudomonas.s__aeruginosa.t__PAb1
0.982
d__Bacteria.p__Proteobacteria.c__Gammaproteobacteria.o__Pseudomonadales.f__Pseudomonadaceae.g__Pseudomonas.s__aeruginosa.t__UCBPP_PA14
0.998
d__Bacteria.p__Proteobacteria.c__Gammaproteobacteria.o__Pseudomonadales.f__Pseudomonadaceae.g__Pseudomonas.s__aeruginosa.t__LESB58
d__Bacteria.p__Proteobacteria.c__Gammaproteobacteria.o__Pseudomonadales.f__Pseudomonadaceae.g__Pseudomonas.s__aeruginosa.t__PACS2
0.947
d__Bacteria.p__Proteobacteria.c__Gammaproteobacteria.o__Pseudomonadales.f__Pseudomonadaceae.g__Pseudomonas.s__aeruginosa.t__C3719
1.000
d__Bacteria.p__Proteobacteria.c__Gammaproteobacteria.o__Pseudomonadales.f__Pseudomonadaceae.g__Pseudomonas.s__aeruginosa.t__2192
d__Bacteria.p__Proteobacteria.c__Gammaproteobacteria.o__Pseudomonadales.f__Pseudomonadaceae.g__Pseudomonas.s__aeruginosa.t__PAO1
d__Bacteria.p__Proteobacteria.c__Gammaproteobacteria.o__Pseudomonadales.f__Pseudomonadaceae.g__Pseudomonas.s__aeruginosa.t__PA7
1.000
d__Bacteria.p__Proteobacteria.c__Gammaproteobacteria.o__Pseudomonadales.f__Pseudomonadaceae.g__Pseudomonas.s__stutzeri.t__ATCC_17588___LMG_11199
d__Bacteria.p__Proteobacteria.c__Gammaproteobacteria.o__Pseudomonadales.f__Pseudomonadaceae.g__Pseudomonas.s__stutzeri.t__A1501
0.322
d__Bacteria.p__Proteobacteria.c__Gammaproteobacteria.o__Pseudomonadales.f__Pseudomonadaceae.g__Pseudomonas.s__stutzeri.t__DSM_4166
d__Bacteria.p__Proteobacteria.c__Gammaproteobacteria.o__Pseudomonadales.f__Pseudomonadaceae.g__Pseudomonas.s__mendocina.t__ymp
1.000
d__Bacteria.p__Proteobacteria.c__Gammaproteobacteria.o__Pseudomonadales.f__Pseudomonadaceae.g__Pseudomonas.s__mendocina.t__NK_01
d__Bacteria.p__Proteobacteria.c__Gammaproteobacteria.o__Pseudomonadales.f__Pseudomonadaceae.g__Azotobacter.s__vinelandii.t__DJ
d__Bacteria.p__Proteobacteria.c__Gammaproteobacteria.o__Oceanospirillales.f__Oceanospirillaceae.g__Neptuniibacter.s__sp_.t__MED92
0.996
d__Bacteria.p__Proteobacteria.c__Gammaproteobacteria.o__Oceanospirillales.f__Oceanospirillaceae.g__Marinomonas.s__sp_.t__MWYL1
1.000
d__Bacteria.p__Proteobacteria.c__Gammaproteobacteria.o__Oceanospirillales.f__Oceanospirillaceae.g__Marinomonas.s__sp_.t__MED121
0.998
d__Bacteria.p__Proteobacteria.c__Gammaproteobacteria.o__unclassified.f__unclassified.g__Reinekea.s__sp_.t__MED297
1.000
d__Bacteria.p__Proteobacteria.c__Gammaproteobacteria.o__Oceanospirillales.f__Oceanospirillaceae.g__Bermanella.s__sp_.t__RED65
0.762
d__Bacteria.p__Proteobacteria.c__Gammaproteobacteria.o__unclassified.f__unclassified.g__unclassified.s___1.t__IMCC2047
d__Bacteria.p__Proteobacteria.c__Gammaproteobacteria.o__Pseudomonadales.f__unclassified.g__unclassified.s__sp_.t__proteobacterium_HTCC2207
0.999
d__Bacteria.p__Proteobacteria.c__Gammaproteobacteria.o__Alteromonadales.f__unclassified.g__Candidatus_Endobugula.s___1.t__IMCC1989
0.974
1.000
d__Bacteria.p__Proteobacteria.c__Gammaproteobacteria.o__Pseudomonadales.f__Pseudomonadaceae.g__Cellvibrio.s__japonicus.t__Ueda107
0.125
d__Bacteria.p__Proteobacteria.c__Gammaproteobacteria.o__Alteromonadales.f__unclassified.g__Teredinibacter.s__turnerae.t__T7901
1.000
1.000
d__Bacteria.p__Proteobacteria.c__Gammaproteobacteria.o__Alteromonadales.f__Alteromonadaceae.g__Saccharophagus.s__degradans.t__2_40
d__Bacteria.p__Proteobacteria.c__Gammaproteobacteria.o__unclassified.f__unclassified.g__unclassified.s__sp_.t__proteobacterium_HTCC2143
d__Bacteria.p__Proteobacteria.c__Gammaproteobacteria.o__unclassified.f__unclassified.g__unclassified.s__gamma.t__proteobacterium_HTCC2148
1.000
0.558
d__Bacteria.p__Proteobacteria.c__Gammaproteobacteria.o__unclassified.f__unclassified.g__Congregibacter.s__litoralis.t__KT71
1.000
d__Bacteria.p__Proteobacteria.c__Gammaproteobacteria.o__unclassified.f__unclassified.g__unclassified.s__proteobacterium.t__NOR5_3
1.000
d__Bacteria.p__Proteobacteria.c__Gammaproteobacteria.o__unclassified.f__unclassified.g__unclassified.s__sp_.t__proteobacterium_HTCC2080
1.000
d__Bacteria.p__Proteobacteria.c__Gammaproteobacteria.o__unclassified.f__unclassified.g__unclassified.s__proteobacterium.t__NOR51_B
0.985
d__Bacteria.p__Proteobacteria.c__Gammaproteobacteria.o__unclassified.f__unclassified.g__unclassified.s___1.t__IMCC3088
d__Bacteria.p__Proteobacteria.c__Gammaproteobacteria.o__Legionellales.f__Legionellaceae.g__Legionella.s__pneumophila.t__2300_99_Alcoy
0.923
d__Bacteria.p__Proteobacteria.c__Gammaproteobacteria.o__Legionellales.f__Legionellaceae.g__Legionella.s__pneumophila.t__str__Corby
d__Bacteria.p__Proteobacteria.c__Gammaproteobacteria.o__Legionellales.f__Legionellaceae.g__Legionella.s__pneumophila.t__subsp__pneumophila_str__Philadelphia_1
d__Bacteria.p__Proteobacteria.c__Gammaproteobacteria.o__Legionellales.f__Legionellaceae.g__Legionella.s__pneumophila.t__130b
0.998
d__Bacteria.p__Proteobacteria.c__Gammaproteobacteria.o__Legionellales.f__Legionellaceae.g__Legionella.s__pneumophila.t__str__Lens
1.000
d__Bacteria.p__Proteobacteria.c__Gammaproteobacteria.o__Legionellales.f__Legionellaceae.g__Legionella.s__pneumophila.t__str__Paris

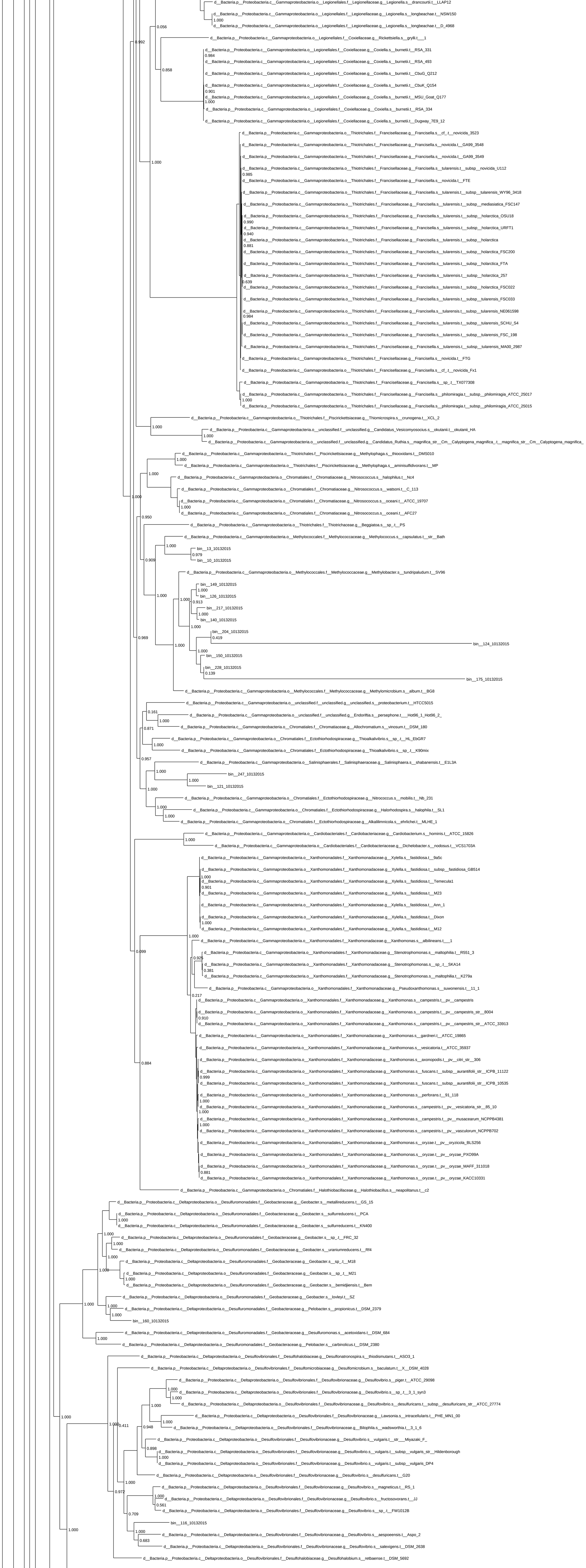
d__Bacteria.p__Proteobacteria.c__Gammaproteobacteria.o__Legionellales.f__Legionellaceae.g__Legionella.s__drancourtii.t__LLAP12
d__Bacteria.p__Proteobacteria.c__Gammaproteobacteria.o__Legionellales.f__Legionellaceae.g__Legionella.s__longbeachae.t__NSW150
d__Bacteria.p__Proteobacteria.c__Gammaproteobacteria.o__Legionellales.f__Legionellaceae.g__Legionella.s__longbeachae.t__D_4968
d__Bacteria.p__Proteobacteria.c__Gammaproteobacteria.o__Legionellales.f__Coxiellaceae.g__Rickettsiella.s__grylli.t___1
d__Bacteria.p__Proteobacteria.c__Gammaproteobacteria.o__Legionellales.f__Coxiellaceae.g__Coxiella.s__burnetii.t__RSA_331
d__Bacteria.p__Proteobacteria.c__Gammaproteobacteria.o__Legionellales.f__Coxiellaceae.g__Coxiella.s__burnetii.t__RSA_493
d__Bacteria.p__Proteobacteria.c__Gammaproteobacteria.o__Legionellales.f__Coxiellaceae.g__Coxiella.s__burnetii.t__CbuG_Q212
d__Bacteria.p__Proteobacteria.c__Gammaproteobacteria.o__Legionellales.f__Coxiellaceae.g__Coxiella.s__burnetii.t__CbuK_Q154
d__Bacteria.p__Proteobacteria.c__Gammaproteobacteria.o__Legionellales.f__Coxiellaceae.g__Coxiella.s__burnetii.t__MSU_Goat_Q177
d__Bacteria.p__Proteobacteria.c__Gammaproteobacteria.o__Legionellales.f__Coxiellaceae.g__Coxiella.s__burnetii.t__RSA_334
d__Bacteria.p__Proteobacteria.c__Gammaproteobacteria.o__Legionellales.f__Coxiellaceae.g__Coxiella.s__burnetii.t__Dugway_7E9_12
d__Bacteria.p__Proteobacteria.c__Gammaproteobacteria.o__Thiotrichales.f__Francisellaceae.g__Francisella.s__cf._t__novicida_3523
d__Bacteria.p__Proteobacteria.c__Gammaproteobacteria.o__Thiotrichales.f__Francisellaceae.g__Francisella.s__novicida.t__GA99_3548
d__Bacteria.p__Proteobacteria.c__Gammaproteobacteria.o__Thiotrichales.f__Francisellaceae.g__Francisella.s__novicida.t__GA99_3549
d__Bacteria.p__Proteobacteria.c__Gammaproteobacteria.o__Thiotrichales.f__Francisellaceae.g__Francisella.s__tularensis.t__subsp__novicida_U112
d__Bacteria.p__Proteobacteria.c__Gammaproteobacteria.o__Thiotrichales.f__Francisellaceae.g__Francisella.s__novicida.t__FTE
d__Bacteria.p__Proteobacteria.c__Gammaproteobacteria.o__Thiotrichales.f__Francisellaceae.g__Francisella.s__tularensis.t__subsp__tularensis_WY96_3418
d__Bacteria.p__Proteobacteria.c__Gammaproteobacteria.o__Thiotrichales.f__Francisellaceae.g__Francisella.s__tularensis.t__subsp__mediasiatica_FSC147
d__Bacteria.p__Proteobacteria.c__Gammaproteobacteria.o__Thiotrichales.f__Francisellaceae.g__Francisella.s__tularensis.t__subsp__holarctica_OSU18
d__Bacteria.p__Proteobacteria.c__Gammaproteobacteria.o__Thiotrichales.f__Francisellaceae.g__Francisella.s__tularensis.t__subsp__holarctica_URFT1
d__Bacteria.p__Proteobacteria.c__Gammaproteobacteria.o__Thiotrichales.f__Francisellaceae.g__Francisella.s__tularensis.t__subsp__holarctica
d__Bacteria.p__Proteobacteria.c__Gammaproteobacteria.o__Thiotrichales.f__Francisellaceae.g__Francisella.s__tularensis.t__subsp__holarctica_FSC200
d__Bacteria.p__Proteobacteria.c__Gammaproteobacteria.o__Thiotrichales.f__Francisellaceae.g__Francisella.s__tularensis.t__subsp__holarctica_FTA
d__Bacteria.p__Proteobacteria.c__Gammaproteobacteria.o__Thiotrichales.f__Francisellaceae.g__Francisella.s__tularensis.t__subsp__holarctica_257
d__Bacteria.p__Proteobacteria.c__Gammaproteobacteria.o__Thiotrichales.f__Francisellaceae.g__Francisella.s__tularensis.t__subsp__holarctica_FSC022
d__Bacteria.p__Proteobacteria.c__Gammaproteobacteria.o__Thiotrichales.f__Francisellaceae.g__Francisella.s__tularensis.t__subsp__tularensis_FSC033
d__Bacteria.p__Proteobacteria.c__Gammaproteobacteria.o__Thiotrichales.f__Francisellaceae.g__Francisella.s__tularensis.t__subsp__tularensis_NE061598
d__Bacteria.p__Proteobacteria.c__Gammaproteobacteria.o__Thiotrichales.f__Francisellaceae.g__Francisella.s__tularensis.t__subsp__tularensis_SCHU_S4
d__Bacteria.p__Proteobacteria.c__Gammaproteobacteria.o__Thiotrichales.f__Francisellaceae.g__Francisella.s__tularensis.t__subsp__tularensis_FSC_198
d__Bacteria.p__Proteobacteria.c__Gammaproteobacteria.o__Thiotrichales.f__Francisellaceae.g__Francisella.s__tularensis.t__subsp__tularensis_MA00_2987
d__Bacteria.p__Proteobacteria.c__Gammaproteobacteria.o__Thiotrichales.f__Francisellaceae.g__Francisella.s__novicida.t__FTG
d__Bacteria.p__Proteobacteria.c__Gammaproteobacteria.o__Thiotrichales.f__Francisellaceae.g__Francisella.s__cf._t__novicida_Fx1
d__Bacteria.p__Proteobacteria.c__Gammaproteobacteria.o__Thiotrichales.f__Francisellaceae.g__Francisella.s__sp_t__TX077308
d__Bacteria.p__Proteobacteria.c__Gammaproteobacteria.o__Thiotrichales.f__Francisellaceae.g__Francisella.s__philomiragia.t__subsp__philomiragia_ATCC_25017
d__Bacteria.p__Proteobacteria.c__Gammaproteobacteria.o__Thiotrichales.f__Francisellaceae.g__Francisella.s__philomiragia.t__subsp__philomiragia_ATCC_25015
d__Bacteria.p__Proteobacteria.c__Gammaproteobacteria.o__Thiotrichales.f__Piscirickettsiaceae.g__Thiomicrospira.s__crunogena.t__XCL_2
d__Bacteria.p__Proteobacteria.c__Gammaproteobacteria.o__unclassified.f__unclassified.g__Candidatus_Vesicomyosocius.s__okutanii.t__okutanii_HA
d__Bacteria.p__Proteobacteria.c__Gammaproteobacteria.o__unclassified.f__unclassified.g__Candidatus_Ruthia.s__magnifica_str__Cm__Calyptogena_magnifica_.t__magnifica_str__Cm__Calyptogena_magnifica_
d__Bacteria.p__Proteobacteria.c__Gammaproteobacteria.o__Thiotrichales.f__Piscirickettsiaceae.g__Methylophaga.s__thiooxidans.t__DMS010
d__Bacteria.p__Proteobacteria.c__Gammaproteobacteria.o__Thiotrichales.f__Piscirickettsiaceae.g__Methylophaga.s__aminisulfidivorans.t__MP
d__Bacteria.p__Proteobacteria.c__Gammaproteobacteria.o__Chromatiales.f__Chromatiaceae.g__Nitrosococcus.s__halophilus.t__Nc4
d__Bacteria.p__Proteobacteria.c__Gammaproteobacteria.o__Chromatiales.f__Chromatiaceae.g__Nitrosococcus.s__watsoni.t__C_113
d__Bacteria.p__Proteobacteria.c__Gammaproteobacteria.o__Chromatiales.f__Chromatiaceae.g__Nitrosococcus.s__oceani.t__ATCC_19707
d__Bacteria.p__Proteobacteria.c__Gammaproteobacteria.o__Chromatiales.f__Chromatiaceae.g__Nitrosococcus.s__oceani.t__AFC27
d__Bacteria.p__Proteobacteria.c__Gammaproteobacteria.o__Thiotrichales.f__Thiotrichaceae.g__Beggiatoa.s__sp_t__PS
d__Bacteria.p__Proteobacteria.c__Gammaproteobacteria.o__Methylococcales.f__Methylococcaceae.g__Methylococcus.s__capsulatus.t__str__Bath
bin__13_10132015
bin__10_10132015
d__Bacteria.p__Proteobacteria.c__Gammaproteobacteria.o__Methylococcales.f__Methylococcaceae.g__Methylobacter.s__tundripaludum.t__SV96
bin__149_10132015
bin__126_10132015
bin__217_10132015
bin__140_10132015
bin__204_10132015
bin__124_10132015
bin__150_10132015
bin__228_10132015
bin__175_10132015
d__Bacteria.p__Proteobacteria.c__Gammaproteobacteria.o__Methylococcales.f__Methylococcaceae.g__Methylomicrobium.s__album.t__BG8
d__Bacteria.p__Proteobacteria.c__Gammaproteobacteria.o__unclassified.f__unclassified.g__unclassified.s__proteobacterium.t__HTCC5015
d__Bacteria.p__Proteobacteria.c__Gammaproteobacteria.o__unclassified.f__unclassified.g__Endoriftia.s__persephone.t___Hot96_1_Hot96_2_
d__Bacteria.p__Proteobacteria.c__Gammaproteobacteria.o__Chromatiales.f__Chromatiaceae.g__Allochromatium.s__vinosum.t__DSM_180
d__Bacteria.p__Proteobacteria.c__Gammaproteobacteria.o__Chromatiales.f__Ectothiorhodospiraceae.g__Thioalkalivibrio.s__sp_t__HL_EbGR7
d__Bacteria.p__Proteobacteria.c__Gammaproteobacteria.o__Chromatiales.f__Ectothiorhodospiraceae.g__Thioalkalivibrio.s__sp_t__K90mix
d__Bacteria.p__Proteobacteria.c__Gammaproteobacteria.o__Salinisphaerales.f__Salinisphaeraceae.g__Salinisphaera.s__shabanensis.t__E1L3A
bin__247_10132015
bin__121_10132015
d__Bacteria.p__Proteobacteria.c__Gammaproteobacteria.o__Chromatiales.f__Ectothiorhodospiraceae.g__Nitrococcus.s__mobilis.t__Nb_231
d__Bacteria.p__Proteobacteria.c__Gammaproteobacteria.o__Chromatiales.f__Ectothiorhodospiraceae.g__Halorhodospira.s__halophila.t__SL1
d__Bacteria.p__Proteobacteria.c__Gammaproteobacteria.o__Chromatiales.f__Ectothiorhodospiraceae.g__Alkalilimnicola.s__ehrlichei.t__MLHE_1
d__Bacteria.p__Proteobacteria.c__Gammaproteobacteria.o__Cardiobacteriales.f__Cardiobacteriaceae.g__Cardiobacterium.s__hominis.t__ATCC_15826
d__Bacteria.p__Proteobacteria.c__Gammaproteobacteria.o__Cardiobacteriales.f__Cardiobacteriaceae.g__Dichelobacter.s__nodosus.t__VCS1703A
d__Bacteria.p__Proteobacteria.c__Gammaproteobacteria.o__Xanthomonadales.f__Xanthomonadaceae.g__Xylella.s__fastidiosa.t__9a5c
d__Bacteria.p__Proteobacteria.c__Gammaproteobacteria.o__Xanthomonadales.f__Xanthomonadaceae.g__Xylella.s__fastidiosa.t__subsp__fastidiosa_GB514
d__Bacteria.p__Proteobacteria.c__Gammaproteobacteria.o__Xanthomonadales.f__Xanthomonadaceae.g__Xylella.s__fastidiosa.t__Temecula1
d__Bacteria.p__Proteobacteria.c__Gammaproteobacteria.o__Xanthomonadales.f__Xanthomonadaceae.g__Xylella.s__fastidiosa.t__M23
d__Bacteria.p__Proteobacteria.c__Gammaproteobacteria.o__Xanthomonadales.f__Xanthomonadaceae.g__Xylella.s__fastidiosa.t__Ann_1
d__Bacteria.p__Proteobacteria.c__Gammaproteobacteria.o__Xanthomonadales.f__Xanthomonadaceae.g__Xylella.s__fastidiosa.t__Dixon
d__Bacteria.p__Proteobacteria.c__Gammaproteobacteria.o__Xanthomonadales.f__Xanthomonadaceae.g__Xylella.s__fastidiosa.t__M12
d__Bacteria.p__Proteobacteria.c__Gammaproteobacteria.o__Xanthomonadales.f__Xanthomonadaceae.g__Xanthomonas.s__albilineans.t___1
d__Bacteria.p__Proteobacteria.c__Gammaproteobacteria.o__Xanthomonadales.f__Xanthomonadaceae.g__Stenotrophomonas.s__maltophilia.t__R551_3
d__Bacteria.p__Proteobacteria.c__Gammaproteobacteria.o__Xanthomonadales.f__Xanthomonadaceae.g__Stenotrophomonas.s__sp_t__SKA14
d__Bacteria.p__Proteobacteria.c__Gammaproteobacteria.o__Xanthomonadales.f__Xanthomonadaceae.g__Stenotrophomonas.s__maltophilia.t__K279a
d__Bacteria.p__Proteobacteria.c__Gammaproteobacteria.o__Xanthomonadales.f__Xanthomonadaceae.g__Pseudoxanthomonas.s__suwonensis.t__11_1
d__Bacteria.p__Proteobacteria.c__Gammaproteobacteria.o__Xanthomonadales.f__Xanthomonadaceae.g__Xanthomonas.s__campestris.t__pv__campestris
d__Bacteria.p__Proteobacteria.c__Gammaproteobacteria.o__Xanthomonadales.f__Xanthomonadaceae.g__Xanthomonas.s__campestris.t__pv__campestris_str__8004
d__Bacteria.p__Proteobacteria.c__Gammaproteobacteria.o__Xanthomonadales.f__Xanthomonadaceae.g__Xanthomonas.s__campestris.t__pv__campestris_str__ATCC_33913
d__Bacteria.p__Proteobacteria.c__Gammaproteobacteria.o__Xanthomonadales.f__Xanthomonadaceae.g__Xanthomonas.s__gardneri.t__ATCC_19865
d__Bacteria.p__Proteobacteria.c__Gammaproteobacteria.o__Xanthomonadales.f__Xanthomonadaceae.g__Xanthomonas.s__vesicatoria.t__ATCC_35937
d__Bacteria.p__Proteobacteria.c__Gammaproteobacteria.o__Xanthomonadales.f__Xanthomonadaceae.g__Xanthomonas.s__axonopodis.t__pv__citri_str__306
d__Bacteria.p__Proteobacteria.c__Gammaproteobacteria.o__Xanthomonadales.f__Xanthomonadaceae.g__Xanthomonas.s__fuscans.t__subsp__aurantifolii_str__ICPB_11122
d__Bacteria.p__Proteobacteria.c__Gammaproteobacteria.o__Xanthomonadales.f__Xanthomonadaceae.g__Xanthomonas.s__fuscans.t__subsp__aurantifolii_str__ICPB_10535
d__Bacteria.p__Proteobacteria.c__Gammaproteobacteria.o__Xanthomonadales.f__Xanthomonadaceae.g__Xanthomonas.s__perforans.t__91_118
d__Bacteria.p__Proteobacteria.c__Gammaproteobacteria.o__Xanthomonadales.f__Xanthomonadaceae.g__Xanthomonas.s__campestris.t__pv__vesicatoria_str__85_10
d__Bacteria.p__Proteobacteria.c__Gammaproteobacteria.o__Xanthomonadales.f__Xanthomonadaceae.g__Xanthomonas.s__campestris.t__pv__musacearum_NCPPB4381
d__Bacteria.p__Proteobacteria.c__Gammaproteobacteria.o__Xanthomonadales.f__Xanthomonadaceae.g__Xanthomonas.s__campestris.t__pv__vasculorum_NCPPB702
d__Bacteria.p__Proteobacteria.c__Gammaproteobacteria.o__Xanthomonadales.f__Xanthomonadaceae.g__Xanthomonas.s__oryzae.t__pv__oryzicola_BLS256
d__Bacteria.p__Proteobacteria.c__Gammaproteobacteria.o__Xanthomonadales.f__Xanthomonadaceae.g__Xanthomonas.s__oryzae.t__pv__oryzae_PXO99A
d__Bacteria.p__Proteobacteria.c__Gammaproteobacteria.o__Xanthomonadales.f__Xanthomonadaceae.g__Xanthomonas.s__oryzae.t__pv__oryzae_MAFF_311018
d__Bacteria.p__Proteobacteria.c__Gammaproteobacteria.o__Xanthomonadales.f__Xanthomonadaceae.g__Xanthomonas.s__oryzae.t__pv__oryzae_KACC10331
d__Bacteria.p__Proteobacteria.c__Gammaproteobacteria.o__Chromatiales.f__Halothiobacillaceae.g__Halothiobacillus.s__neapolitanus.t__c2
d__Bacteria.p__Proteobacteria.c__Deltaproteobacteria.o__Desulfuromonadales.f__Geobacteraceae.g__Geobacter.s__metallireducens.t__GS_15
d__Bacteria.p__Proteobacteria.c__Deltaproteobacteria.o__Desulfuromonadales.f__Geobacteraceae.g__Geobacter.s__sulfurreducens.t__PCA
d__Bacteria.p__Proteobacteria.c__Deltaproteobacteria.o__Desulfuromonadales.f__Geobacteraceae.g__Geobacter.s__sulfurreducens.t__KN400
d__Bacteria.p__Proteobacteria.c__Deltaproteobacteria.o__Desulfuromonadales.f__Geobacteraceae.g__Geobacter.s__sp_t__FRC_32
d__Bacteria.p__Proteobacteria.c__Deltaproteobacteria.o__Desulfuromonadales.f__Geobacteraceae.g__Geobacter.s__uraniumreducens.t__Rf4
d__Bacteria.p__Proteobacteria.c__Deltaproteobacteria.o__Desulfuromonadales.f__Geobacteraceae.g__Geobacter.s__sp_t__M18
d__Bacteria.p__Proteobacteria.c__Deltaproteobacteria.o__Desulfuromonadales.f__Geobacteraceae.g__Geobacter.s__sp_t__M21
d__Bacteria.p__Proteobacteria.c__Deltaproteobacteria.o__Desulfuromonadales.f__Geobacteraceae.g__Geobacter.s__bemidjiensis.t__Bem
d__Bacteria.p__Proteobacteria.c__Deltaproteobacteria.o__Desulfuromonadales.f__Geobacteraceae.g__Geobacter.s__lovleyi.t__SZ
d__Bacteria.p__Proteobacteria.c__Deltaproteobacteria.o__Desulfuromonadales.f__Geobacteraceae.g__Pelobacter.s__propionicus.t__DSM_2379
bin__160_10132015
d__Bacteria.p__Proteobacteria.c__Deltaproteobacteria.o__Desulfuromonadales.f__Geobacteraceae.g__Desulfuromonas.s__acetoxidans.t__DSM_684
d__Bacteria.p__Proteobacteria.c__Deltaproteobacteria.o__Desulfuromonadales.f__Geobacteraceae.g__Pelobacter.s__carbinolicus.t__DSM_2380
d__Bacteria.p__Proteobacteria.c__Deltaproteobacteria.o__Desulfovibrionales.f__Desulfohalobiaceae.g__Desulfonatronospira.s__thiodismutans.t__ASO3_1
d__Bacteria.p__Proteobacteria.c__Deltaproteobacteria.o__Desulfovibrionales.f__Desulfomicrobiaceae.g__Desulfomicrobium.s__baculatum.t__X__DSM_4028
d__Bacteria.p__Proteobacteria.c__Deltaproteobacteria.o__Desulfovibrionales.f__Desulfovibrionaceae.g__Desulfovibrio.s__piger.t__ATCC_29098
d__Bacteria.p__Proteobacteria.c__Deltaproteobacteria.o__Desulfovibrionales.f__Desulfovibrionaceae.g__Desulfovibrio.s__sp_t__3_1_syn3
d__Bacteria.p__Proteobacteria.c__Deltaproteobacteria.o__Desulfovibrionales.f__Desulfovibrionaceae.g__Desulfovibrio.s__desulfuricans.t__subsp__desulfuricans_str__ATCC_27774
d__Bacteria.p__Proteobacteria.c__Deltaproteobacteria.o__Desulfovibrionales.f__Desulfovibrionaceae.g__Lawsonia.s__intracellularis.t__PHE_MN1_00
d__Bacteria.p__Proteobacteria.c__Deltaproteobacteria.o__Desulfovibrionales.f__Desulfovibrionaceae.g__Bilophila.s__wadsworthia.t__3_1_6
d__Bacteria.p__Proteobacteria.c__Deltaproteobacteria.o__Desulfovibrionales.f__Desulfovibrionaceae.g__Desulfovibrio.s__vulgaris.t__str___Miyazaki_F_
d__Bacteria.p__Proteobacteria.c__Deltaproteobacteria.o__Desulfovibrionales.f__Desulfovibrionaceae.g__Desulfovibrio.s__vulgaris.t__subsp__vulgaris_str__Hildenborough
d__Bacteria.p__Proteobacteria.c__Deltaproteobacteria.o__Desulfovibrionales.f__Desulfovibrionaceae.g__Desulfovibrio.s__vulgaris.t__subsp__vulgaris_DP4
d__Bacteria.p__Proteobacteria.c__Deltaproteobacteria.o__Desulfovibrionales.f__Desulfovibrionaceae.g__Desulfovibrio.s__desulfuricans.t__G20
d__Bacteria.p__Proteobacteria.c__Deltaproteobacteria.o__Desulfovibrionales.f__Desulfovibrionaceae.g__Desulfovibrio.s__magneticus.t__RS_1
d__Bacteria.p__Proteobacteria.c__Deltaproteobacteria.o__Desulfovibrionales.f__Desulfovibrionaceae.g__Desulfovibrio.s__fructosovorans.t__JJ
d__Bacteria.p__Proteobacteria.c__Deltaproteobacteria.o__Desulfovibrionales.f__Desulfovibrionaceae.g__Desulfovibrio.s__sp_t__FW1012B
bin__116_10132015
d__Bacteria.p__Proteobacteria.c__Deltaproteobacteria.o__Desulfovibrionales.f__Desulfovibrionaceae.g__Desulfovibrio.s__aespoeensis.t__Aspo_2
d__Bacteria.p__Proteobacteria.c__Deltaproteobacteria.o__Desulfovibrionales.f__Desulfovibrionaceae.g__Desulfovibrio.s__salexigens.t__DSM_2638
d__Bacteria.p__Proteobacteria.c__Deltaproteobacteria.o__Desulfovibrionales.f__Desulfohalobiaceae.g__Desulfohalobium.s__retbaense.t__DSM_5692

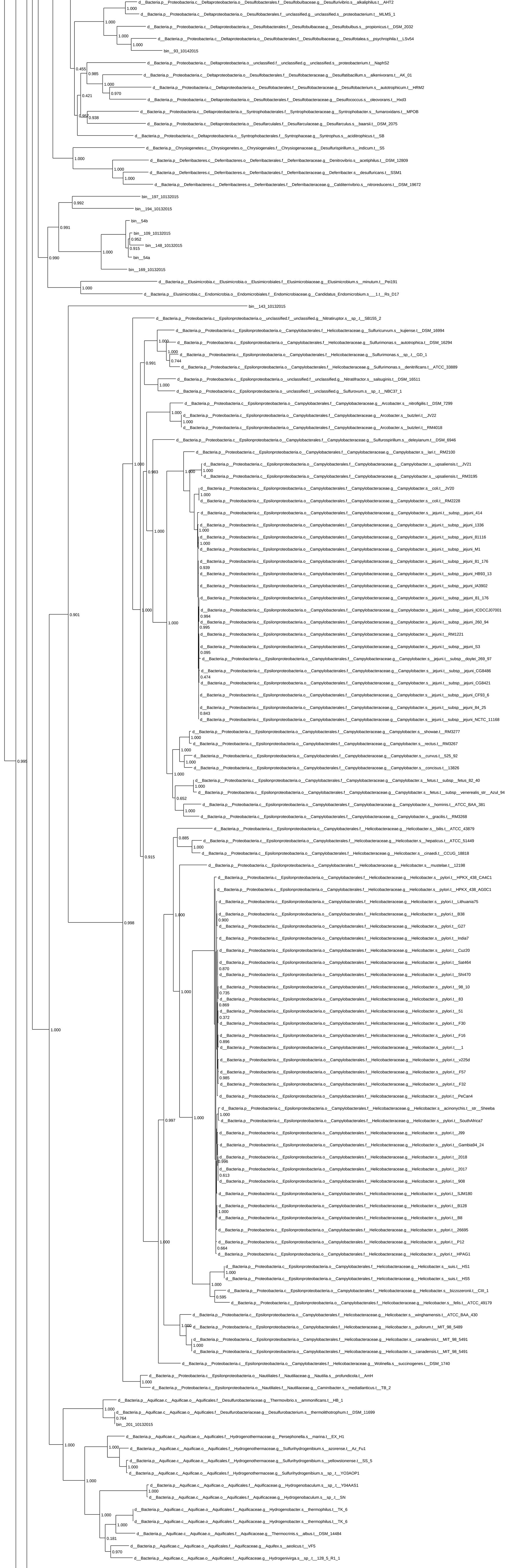
d__Bacteria.p__Proteobacteria.c__Deltaproteobacteria.o__Desulfobacterales.f__Desulfobulbaceae.g__Desulfurivibrio.s__alkaliphilus.t__AHT2
d__Bacteria.p__Proteobacteria.c__Deltaproteobacteria.o__Desulfobacterales.f__unclassified.g__unclassified.s__proteobacterium.t__MLMS_1
d__Bacteria.p__Proteobacteria.c__Deltaproteobacteria.o__Desulfobacterales.f__Desulfobulbaceae.g__Desulfobulbus.s__propionicus.t__DSM_2032
d__Bacteria.p__Proteobacteria.c__Deltaproteobacteria.o__Desulfobacterales.f__Desulfobulbaceae.g__Desulfotalea.s__psychrophila.t__LSv54
bin__93_10142015
d__Bacteria.p__Proteobacteria.c__Deltaproteobacteria.o__unclassified.f__unclassified.g__unclassified.s__proteobacterium.t__NaphS2
d__Bacteria.p__Proteobacteria.c__Deltaproteobacteria.o__Desulfobacterales.f__Desulfobacteraceae.g__Desulfatibacillum.s__alkenivorans.t__AK_01
d__Bacteria.p__Proteobacteria.c__Deltaproteobacteria.o__Desulfobacterales.f__Desulfobacteraceae.g__Desulfobacterium.s__autotrophicum.t__HRM2
d__Bacteria.p__Proteobacteria.c__Deltaproteobacteria.o__Desulfobacterales.f__Desulfobacteraceae.g__Desulfococcus.s__oleovorans.t__Hxd3
d__Bacteria.p__Proteobacteria.c__Deltaproteobacteria.o__Syntrophobacterales.f__Syntrophobacteraceae.g__Syntrophobacter.s__fumaroxidans.t__MPOB
d__Bacteria.p__Proteobacteria.c__Deltaproteobacteria.o__Desulfarculales.f__Desulfarculaceae.g__Desulfarculus.s__baarsii.t__DSM_2075
d__Bacteria.p__Proteobacteria.c__Deltaproteobacteria.o__Syntrophobacterales.f__Syntrophaceae.g__Syntrophus.s__aciditrophicus.t__SB
d__Bacteria.p__Chrysiogenetes.c__Chrysiogenetes.o__Chrysiogenales.f__Chrysiogenaceae.g__Desulfurispirillum.s__indicum.t__S5
d__Bacteria.p__Deferribacteres.c__Deferribacteres.o__Deferribacterales.f__Deferribacteraceae.g__Denitrovibrio.s__acetiphilus.t__DSM_12809
d__Bacteria.p__Deferribacteres.c__Deferribacteres.o__Deferribacterales.f__Deferribacteraceae.g__Deferribacter.s__desulfuricans.t__SSM1
d__Bacteria.p__Deferribacteres.c__Deferribacteres.o__Deferribacterales.f__Deferribacteraceae.g__Calditerrivibrio.s__nitroreducens.t__DSM_19672
bin__197_10132015
bin__194_10132015
bin__54b
bin__109_10132015
bin__148_10132015
bin__54a
bin__169_10132015
d__Bacteria.p__Elusimicrobia.c__Elusimicrobia.o__Elusimicrobiales.f__Elusimicrobiaceae.g__Elusimicrobium.s__minutum.t__Pei191
d__Bacteria.p__Elusimicrobia.c__Endomicrobia.o__Endomicrobiales.f__Endomicrobiaceae.g__Candidatus_Endomicrobium.s___1.t__Rs_D17
bin__143_10132015
d__Bacteria.p__Proteobacteria.c__Epsilonproteobacteria.o__unclassified.f__unclassified.g__Nitratiruptor.s__sp_.t__SB155_2
d__Bacteria.p__Proteobacteria.c__Epsilonproteobacteria.o__Campylobacterales.f__Helicobacteraceae.g__Sulfuricurvum.s__kujiense.t__DSM_16994
d__Bacteria.p__Proteobacteria.c__Epsilonproteobacteria.o__Campylobacterales.f__Helicobacteraceae.g__Sulfurimonas.s__autotrophica.t__DSM_16294
d__Bacteria.p__Proteobacteria.c__Epsilonproteobacteria.o__Campylobacterales.f__Helicobacteraceae.g__Sulfurimonas.s__sp_.t__GD_1
d__Bacteria.p__Proteobacteria.c__Epsilonproteobacteria.o__Campylobacterales.f__Helicobacteraceae.g__Sulfurimonas.s__denitrificans.t__ATCC_33889
d__Bacteria.p__Proteobacteria.c__Epsilonproteobacteria.o__unclassified.f__unclassified.g__Nitratifractor.s__salsuginis.t__DSM_16511
d__Bacteria.p__Proteobacteria.c__Epsilonproteobacteria.o__unclassified.f__unclassified.g__Sulfurovum.s__sp_.t__NBC37_1
d__Bacteria.p__Proteobacteria.c__Epsilonproteobacteria.o__Campylobacterales.f__Campylobacteraceae.g__Arcobacter.s__nitrofigilis.t__DSM_7299
d__Bacteria.p__Proteobacteria.c__Epsilonproteobacteria.o__Campylobacterales.f__Campylobacteraceae.g__Arcobacter.s__butzleri.t__JV22
d__Bacteria.p__Proteobacteria.c__Epsilonproteobacteria.o__Campylobacterales.f__Campylobacteraceae.g__Arcobacter.s__butzleri.t__RM4018
d__Bacteria.p__Proteobacteria.c__Epsilonproteobacteria.o__Campylobacterales.f__Campylobacteraceae.g__Sulfurospirillum.s__deleyianum.t__DSM_6946
d__Bacteria.p__Proteobacteria.c__Epsilonproteobacteria.o__Campylobacterales.f__Campylobacteraceae.g__Campylobacter.s__lari.t__RM2100
d__Bacteria.p__Proteobacteria.c__Epsilonproteobacteria.o__Campylobacterales.f__Campylobacteraceae.g__Campylobacter.s__upsaliensis.t__JV21
d__Bacteria.p__Proteobacteria.c__Epsilonproteobacteria.o__Campylobacterales.f__Campylobacteraceae.g__Campylobacter.s__upsaliensis.t__RM3195
d__Bacteria.p__Proteobacteria.c__Epsilonproteobacteria.o__Campylobacterales.f__Campylobacteraceae.g__Campylobacter.s__coli.t__JV20
d__Bacteria.p__Proteobacteria.c__Epsilonproteobacteria.o__Campylobacterales.f__Campylobacteraceae.g__Campylobacter.s__coli.t__RM2228
d__Bacteria.p__Proteobacteria.c__Epsilonproteobacteria.o__Campylobacterales.f__Campylobacteraceae.g__Campylobacter.s__jejuni.t__subsp__jejuni_414
d__Bacteria.p__Proteobacteria.c__Epsilonproteobacteria.o__Campylobacterales.f__Campylobacteraceae.g__Campylobacter.s__jejuni.t__subsp__jejuni_1336
d__Bacteria.p__Proteobacteria.c__Epsilonproteobacteria.o__Campylobacterales.f__Campylobacteraceae.g__Campylobacter.s__jejuni.t__subsp__jejuni_81116
d__Bacteria.p__Proteobacteria.c__Epsilonproteobacteria.o__Campylobacterales.f__Campylobacteraceae.g__Campylobacter.s__jejuni.t__subsp__jejuni_M1
d__Bacteria.p__Proteobacteria.c__Epsilonproteobacteria.o__Campylobacterales.f__Campylobacteraceae.g__Campylobacter.s__jejuni.t__subsp__jejuni_81_176
d__Bacteria.p__Proteobacteria.c__Epsilonproteobacteria.o__Campylobacterales.f__Campylobacteraceae.g__Campylobacter.s__jejuni.t__subsp__jejuni_HB93_13
d__Bacteria.p__Proteobacteria.c__Epsilonproteobacteria.o__Campylobacterales.f__Campylobacteraceae.g__Campylobacter.s__jejuni.t__subsp__jejuni_IA3902
d__Bacteria.p__Proteobacteria.c__Epsilonproteobacteria.o__Campylobacterales.f__Campylobacteraceae.g__Campylobacter.s__jejuni.t__subsp__jejuni_81_176
d__Bacteria.p__Proteobacteria.c__Epsilonproteobacteria.o__Campylobacterales.f__Campylobacteraceae.g__Campylobacter.s__jejuni.t__subsp__jejuni_ICDCCJ07001
d__Bacteria.p__Proteobacteria.c__Epsilonproteobacteria.o__Campylobacterales.f__Campylobacteraceae.g__Campylobacter.s__jejuni.t__subsp__jejuni_260_94
d__Bacteria.p__Proteobacteria.c__Epsilonproteobacteria.o__Campylobacterales.f__Campylobacteraceae.g__Campylobacter.s__jejuni.t__RM1221
d__Bacteria.p__Proteobacteria.c__Epsilonproteobacteria.o__Campylobacterales.f__Campylobacteraceae.g__Campylobacter.s__jejuni.t__subsp__jejuni_S3
d__Bacteria.p__Proteobacteria.c__Epsilonproteobacteria.o__Campylobacterales.f__Campylobacteraceae.g__Campylobacter.s__jejuni.t__subsp__doylei_269_97
d__Bacteria.p__Proteobacteria.c__Epsilonproteobacteria.o__Campylobacterales.f__Campylobacteraceae.g__Campylobacter.s__jejuni.t__subsp__jejuni_CG8486
d__Bacteria.p__Proteobacteria.c__Epsilonproteobacteria.o__Campylobacterales.f__Campylobacteraceae.g__Campylobacter.s__jejuni.t__subsp__jejuni_CG8421
d__Bacteria.p__Proteobacteria.c__Epsilonproteobacteria.o__Campylobacterales.f__Campylobacteraceae.g__Campylobacter.s__jejuni.t__subsp__jejuni_CF93_6
d__Bacteria.p__Proteobacteria.c__Epsilonproteobacteria.o__Campylobacterales.f__Campylobacteraceae.g__Campylobacter.s__jejuni.t__subsp__jejuni_84_25
d__Bacteria.p__Proteobacteria.c__Epsilonproteobacteria.o__Campylobacterales.f__Campylobacteraceae.g__Campylobacter.s__jejuni.t__subsp__jejuni_NCTC_11168
d__Bacteria.p__Proteobacteria.c__Epsilonproteobacteria.o__Campylobacterales.f__Campylobacteraceae.g__Campylobacter.s__showae.t__RM3277
d__Bacteria.p__Proteobacteria.c__Epsilonproteobacteria.o__Campylobacterales.f__Campylobacteraceae.g__Campylobacter.s__rectus.t__RM3267
d__Bacteria.p__Proteobacteria.c__Epsilonproteobacteria.o__Campylobacterales.f__Campylobacteraceae.g__Campylobacter.s__curvus.t__525_92
d__Bacteria.p__Proteobacteria.c__Epsilonproteobacteria.o__Campylobacterales.f__Campylobacteraceae.g__Campylobacter.s__concisus.t__13826
d__Bacteria.p__Proteobacteria.c__Epsilonproteobacteria.o__Campylobacterales.f__Campylobacteraceae.g__Campylobacter.s__fetus.t__subsp__fetus_82_40
d__Bacteria.p__Proteobacteria.c__Epsilonproteobacteria.o__Campylobacterales.f__Campylobacteraceae.g__Campylobacter.s__fetus.t__subsp__venerealis_str__Azul_94
d__Bacteria.p__Proteobacteria.c__Epsilonproteobacteria.o__Campylobacterales.f__Campylobacteraceae.g__Campylobacter.s__hominis.t__ATCC_BAA_381
d__Bacteria.p__Proteobacteria.c__Epsilonproteobacteria.o__Campylobacterales.f__Campylobacteraceae.g__Campylobacter.s__gracilis.t__RM3268
d__Bacteria.p__Proteobacteria.c__Epsilonproteobacteria.o__Campylobacterales.f__Helicobacteraceae.g__Helicobacter.s__bilis.t__ATCC_43879
d__Bacteria.p__Proteobacteria.c__Epsilonproteobacteria.o__Campylobacterales.f__Helicobacteraceae.g__Helicobacter.s__hepaticus.t__ATCC_51449
d__Bacteria.p__Proteobacteria.c__Epsilonproteobacteria.o__Campylobacterales.f__Helicobacteraceae.g__Helicobacter.s__cinaedi.t__CCUG_18818
d__Bacteria.p__Proteobacteria.c__Epsilonproteobacteria.o__Campylobacterales.f__Helicobacteraceae.g__Helicobacter.s__mustelae.t__12198
d__Bacteria.p__Proteobacteria.c__Epsilonproteobacteria.o__Campylobacterales.f__Helicobacteraceae.g__Helicobacter.s__pylori.t__HPKX_438_CA4C1
d__Bacteria.p__Proteobacteria.c__Epsilonproteobacteria.o__Campylobacterales.f__Helicobacteraceae.g__Helicobacter.s__pylori.t__HPKX_438_AG0C1
d__Bacteria.p__Proteobacteria.c__Epsilonproteobacteria.o__Campylobacterales.f__Helicobacteraceae.g__Helicobacter.s__pylori.t__Lithuania75
d__Bacteria.p__Proteobacteria.c__Epsilonproteobacteria.o__Campylobacterales.f__Helicobacteraceae.g__Helicobacter.s__pylori.t__B38
d__Bacteria.p__Proteobacteria.c__Epsilonproteobacteria.o__Campylobacterales.f__Helicobacteraceae.g__Helicobacter.s__pylori.t__G27
d__Bacteria.p__Proteobacteria.c__Epsilonproteobacteria.o__Campylobacterales.f__Helicobacteraceae.g__Helicobacter.s__pylori.t__India7
d__Bacteria.p__Proteobacteria.c__Epsilonproteobacteria.o__Campylobacterales.f__Helicobacteraceae.g__Helicobacter.s__pylori.t__Cuz20
d__Bacteria.p__Proteobacteria.c__Epsilonproteobacteria.o__Campylobacterales.f__Helicobacteraceae.g__Helicobacter.s__pylori.t__Sat464
d__Bacteria.p__Proteobacteria.c__Epsilonproteobacteria.o__Campylobacterales.f__Helicobacteraceae.g__Helicobacter.s__pylori.t__Shi470
d__Bacteria.p__Proteobacteria.c__Epsilonproteobacteria.o__Campylobacterales.f__Helicobacteraceae.g__Helicobacter.s__pylori.t__98_10
d__Bacteria.p__Proteobacteria.c__Epsilonproteobacteria.o__Campylobacterales.f__Helicobacteraceae.g__Helicobacter.s__pylori.t__83
d__Bacteria.p__Proteobacteria.c__Epsilonproteobacteria.o__Campylobacterales.f__Helicobacteraceae.g__Helicobacter.s__pylori.t__51
d__Bacteria.p__Proteobacteria.c__Epsilonproteobacteria.o__Campylobacterales.f__Helicobacteraceae.g__Helicobacter.s__pylori.t__F30
d__Bacteria.p__Proteobacteria.c__Epsilonproteobacteria.o__Campylobacterales.f__Helicobacteraceae.g__Helicobacter.s__pylori.t__F16
d__Bacteria.p__Proteobacteria.c__Epsilonproteobacteria.o__Campylobacterales.f__Helicobacteraceae.g__Helicobacter.s__pylori.t___1
d__Bacteria.p__Proteobacteria.c__Epsilonproteobacteria.o__Campylobacterales.f__Helicobacteraceae.g__Helicobacter.s__pylori.t__v225d
d__Bacteria.p__Proteobacteria.c__Epsilonproteobacteria.o__Campylobacterales.f__Helicobacteraceae.g__Helicobacter.s__pylori.t__F57
d__Bacteria.p__Proteobacteria.c__Epsilonproteobacteria.o__Campylobacterales.f__Helicobacteraceae.g__Helicobacter.s__pylori.t__F32
d__Bacteria.p__Proteobacteria.c__Epsilonproteobacteria.o__Campylobacterales.f__Helicobacteraceae.g__Helicobacter.s__pylori.t__PeCan4
d__Bacteria.p__Proteobacteria.c__Epsilonproteobacteria.o__Campylobacterales.f__Helicobacteraceae.g__Helicobacter.s__acinonychis.t__str__Sheeba
d__Bacteria.p__Proteobacteria.c__Epsilonproteobacteria.o__Campylobacterales.f__Helicobacteraceae.g__Helicobacter.s__pylori.t__SouthAfrica7
d__Bacteria.p__Proteobacteria.c__Epsilonproteobacteria.o__Campylobacterales.f__Helicobacteraceae.g__Helicobacter.s__pylori.t__J99
d__Bacteria.p__Proteobacteria.c__Epsilonproteobacteria.o__Campylobacterales.f__Helicobacteraceae.g__Helicobacter.s__pylori.t__Gambia94_24
d__Bacteria.p__Proteobacteria.c__Epsilonproteobacteria.o__Campylobacterales.f__Helicobacteraceae.g__Helicobacter.s__pylori.t__2018
d__Bacteria.p__Proteobacteria.c__Epsilonproteobacteria.o__Campylobacterales.f__Helicobacteraceae.g__Helicobacter.s__pylori.t__2017
d__Bacteria.p__Proteobacteria.c__Epsilonproteobacteria.o__Campylobacterales.f__Helicobacteraceae.g__Helicobacter.s__pylori.t__908
d__Bacteria.p__Proteobacteria.c__Epsilonproteobacteria.o__Campylobacterales.f__Helicobacteraceae.g__Helicobacter.s__pylori.t__SJM180
d__Bacteria.p__Proteobacteria.c__Epsilonproteobacteria.o__Campylobacterales.f__Helicobacteraceae.g__Helicobacter.s__pylori.t__B128
d__Bacteria.p__Proteobacteria.c__Epsilonproteobacteria.o__Campylobacterales.f__Helicobacteraceae.g__Helicobacter.s__pylori.t__B8
d__Bacteria.p__Proteobacteria.c__Epsilonproteobacteria.o__Campylobacterales.f__Helicobacteraceae.g__Helicobacter.s__pylori.t__26695
d__Bacteria.p__Proteobacteria.c__Epsilonproteobacteria.o__Campylobacterales.f__Helicobacteraceae.g__Helicobacter.s__pylori.t__P12
d__Bacteria.p__Proteobacteria.c__Epsilonproteobacteria.o__Campylobacterales.f__Helicobacteraceae.g__Helicobacter.s__pylori.t__HPAG1
d__Bacteria.p__Proteobacteria.c__Epsilonproteobacteria.o__Campylobacterales.f__Helicobacteraceae.g__Helicobacter.s__suis.t__HS1
d__Bacteria.p__Proteobacteria.c__Epsilonproteobacteria.o__Campylobacterales.f__Helicobacteraceae.g__Helicobacter.s__suis.t__HS5
d__Bacteria.p__Proteobacteria.c__Epsilonproteobacteria.o__Campylobacterales.f__Helicobacteraceae.g__Helicobacter.s__bizzozeronii.t__CIII_1
d__Bacteria.p__Proteobacteria.c__Epsilonproteobacteria.o__Campylobacterales.f__Helicobacteraceae.g__Helicobacter.s__felis.t__ATCC_49179
d__Bacteria.p__Proteobacteria.c__Epsilonproteobacteria.o__Campylobacterales.f__Helicobacteraceae.g__Helicobacter.s__winghamensis.t__ATCC_BAA_430
d__Bacteria.p__Proteobacteria.c__Epsilonproteobacteria.o__Campylobacterales.f__Helicobacteraceae.g__Helicobacter.s__pullorum.t__MIT_98_5489
d__Bacteria.p__Proteobacteria.c__Epsilonproteobacteria.o__Campylobacterales.f__Helicobacteraceae.g__Helicobacter.s__canadensis.t__MIT_98_5491
d__Bacteria.p__Proteobacteria.c__Epsilonproteobacteria.o__Campylobacterales.f__Helicobacteraceae.g__Helicobacter.s__canadensis.t__MIT_98_5491
d__Bacteria.p__Proteobacteria.c__Epsilonproteobacteria.o__Campylobacterales.f__Helicobacteraceae.g__Wolinella.s__succinogenes.t__DSM_1740
d__Bacteria.p__Proteobacteria.c__Epsilonproteobacteria.o__Nautiliales.f__Nautiliaceae.g__Nautilia.s__profundicola.t__AmH
d__Bacteria.p__Proteobacteria.c__Epsilonproteobacteria.o__Nautiliales.f__Nautiliaceae.g__Caminibacter.s__mediatlanticus.t__TB_2
d__Bacteria.p__Aquificae.c__Aquificae.o__Aquificales.f__Desulfurobacteriaceae.g__Thermovibrio.s__ammonificans.t__HB_1
d__Bacteria.p__Aquificae.c__Aquificae.o__Aquificales.f__Desulfurobacteriaceae.g__Desulfurobacterium.s__thermolithotrophum.t__DSM_11699
bin__201_10132015
d__Bacteria.p__Aquificae.c__Aquificae.o__Aquificales.f__Hydrogenothermaceae.g__Persephonella.s__marina.t__EX_H1
d__Bacteria.p__Aquificae.c__Aquificae.o__Aquificales.f__Hydrogenothermaceae.g__Sulfurihydrogenibium.s__azorense.t__Az_Fu1
d__Bacteria.p__Aquificae.c__Aquificae.o__Aquificales.f__Hydrogenothermaceae.g__Sulfurihydrogenibium.s__yellowstonense.t__SS_5
d__Bacteria.p__Aquificae.c__Aquificae.o__Aquificales.f__Hydrogenothermaceae.g__Sulfurihydrogenibium.s__sp_.t__YO3AOP1
d__Bacteria.p__Aquificae.c__Aquificae.o__Aquificales.f__Aquificaceae.g__Hydrogenobaculum.s__sp_.t__Y04AAS1
d__Bacteria.p__Aquificae.c__Aquificae.o__Aquificales.f__Aquificaceae.g__Hydrogenobaculum.s__sp_.t__SN
d__Bacteria.p__Aquificae.c__Aquificae.o__Aquificales.f__Aquificaceae.g__Hydrogenobacter.s__thermophilus.t__TK_6
d__Bacteria.p__Aquificae.c__Aquificae.o__Aquificales.f__Aquificaceae.g__Hydrogenobacter.s__thermophilus.t__TK_6
d__Bacteria.p__Aquificae.c__Aquificae.o__Aquificales.f__Aquificaceae.g__Thermocrinis.s__albus.t__DSM_14484
d__Bacteria.p__Aquificae.c__Aquificae.o__Aquificales.f__Aquificaceae.g__Aquifex.s__aeolicus.t__VF5
d__Bacteria.p__Aquificae.c__Aquificae.o__Aquificales.f__Aquificaceae.g__Hydrogenivirga.s__sp_.t__128_5_R1_1

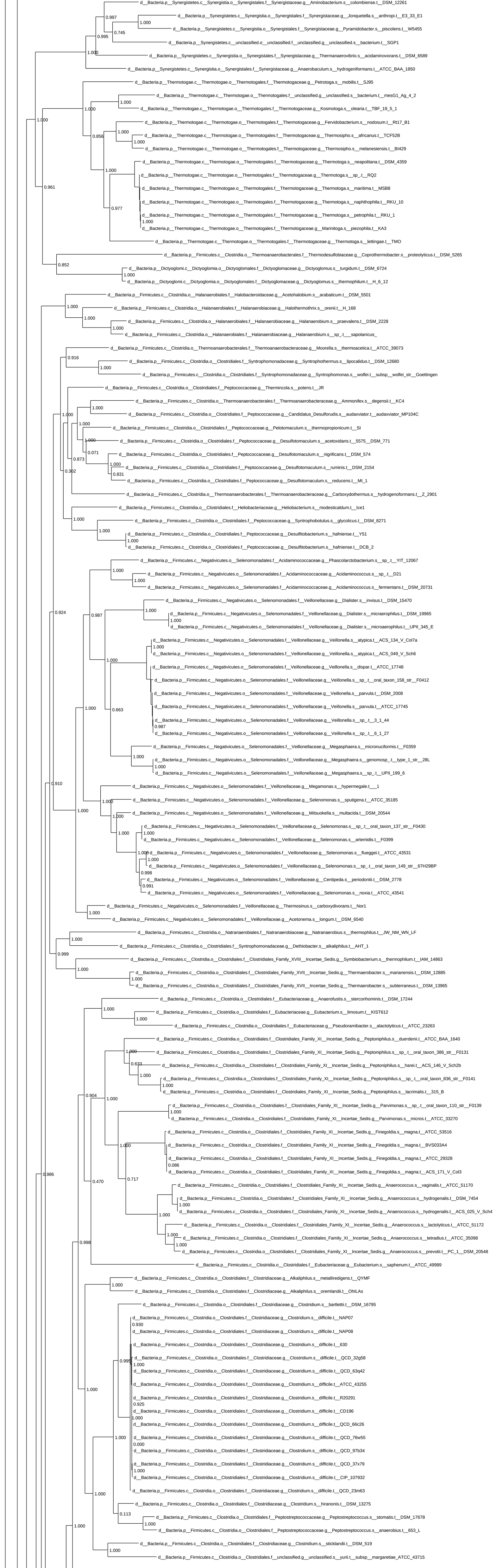
d__Bacteria.p__Synergistetes.c__Synergistia.o__Synergistales.f__Synergistaceae.g__Aminobacterium.s__colombiense.t__DSM_12261
d__Bacteria.p__Synergistetes.c__Synergistia.o__Synergistales.f__Synergistaceae.g__Jonquetella.s__anthropi.t__E3_33_E1
d__Bacteria.p__Synergistetes.c__Synergistia.o__Synergistales.f__Synergistaceae.g__Pyramidobacter.s__piscolens.t__W5455
d__Bacteria.p__Synergistetes.c__unclassified.o__unclassified.f__unclassified.g__unclassified.s__bacterium.t__SGP1
d__Bacteria.p__Synergistetes.c__Synergistia.o__Synergistales.f__Synergistaceae.g__Thermanaerovibrio.s__acidaminovorans.t__DSM_6589
d__Bacteria.p__Synergistetes.c__Synergistia.o__Synergistales.f__Synergistaceae.g__Anaerobaculum.s__hydrogeniformans.t__ATCC_BAA_1850
d__Bacteria.p__Thermotogae.c__Thermotogae.o__Thermotogales.f__Thermotogaceae.g__Petrotoga.s__mobilis.t__SJ95
d__Bacteria.p__Thermotogae.c__Thermotogae.o__Thermotogales.f__unclassified.g__unclassified.s__bacterium.t__mesG1_Ag_4_2
d__Bacteria.p__Thermotogae.c__Thermotogae.o__Thermotogales.f__Thermotogaceae.g__Kosmotoga.s__olearia.t__TBF_19_5_1
d__Bacteria.p__Thermotogae.c__Thermotogae.o__Thermotogales.f__Thermotogaceae.g__Fervidobacterium.s__nodosum.t__Rt17_B1
d__Bacteria.p__Thermotogae.c__Thermotogae.o__Thermotogales.f__Thermotogaceae.g__Thermosipho.s__africanus.t__TCF52B
d__Bacteria.p__Thermotogae.c__Thermotogae.o__Thermotogales.f__Thermotogaceae.g__Thermosipho.s__melanesiensis.t__BI429
d__Bacteria.p__Thermotogae.c__Thermotogae.o__Thermotogales.f__Thermotogaceae.g__Thermotoga.s__neapolitana.t__DSM_4359
d__Bacteria.p__Thermotogae.c__Thermotogae.o__Thermotogales.f__Thermotogaceae.g__Thermotoga.s__sp_.t__RQ2
d__Bacteria.p__Thermotogae.c__Thermotogae.o__Thermotogales.f__Thermotogaceae.g__Thermotoga.s__maritima.t__MSB8
d__Bacteria.p__Thermotogae.c__Thermotogae.o__Thermotogales.f__Thermotogaceae.g__Thermotoga.s__naphthophila.t__RKU_10
d__Bacteria.p__Thermotogae.c__Thermotogae.o__Thermotogales.f__Thermotogaceae.g__Thermotoga.s__petrophila.t__RKU_1
d__Bacteria.p__Thermotogae.c__Thermotogae.o__Thermotogales.f__Thermotogaceae.g__Marinitoga.s__piezophila.t__KA3
d__Bacteria.p__Thermotogae.c__Thermotogae.o__Thermotogales.f__Thermotogaceae.g__Thermotoga.s__lettingae.t__TMO
d__Bacteria.p__Firmicutes.c__Clostridia.o__Thermoanaerobacterales.f__Thermodesulfobiaceae.g__Coprothermobacter.s__proteolyticus.t__DSM_5265
d__Bacteria.p__Dictyoglomi.c__Dictyoglomia.o__Dictyoglomales.f__Dictyoglomaceae.g__Dictyoglomus.s__turgidum.t__DSM_6724
d__Bacteria.p__Dictyoglomi.c__Dictyoglomia.o__Dictyoglomales.f__Dictyoglomaceae.g__Dictyoglomus.s__thermophilum.t__H_6_12
d__Bacteria.p__Firmicutes.c__Clostridia.o__Halanaerobiales.f__Halobacteroidaceae.g__Acetohalobium.s__arabaticum.t__DSM_5501
d__Bacteria.p__Firmicutes.c__Clostridia.o__Halanaerobiales.f__Halanaerobiaceae.g__Halothermothrix.s__orenii.t__H_168
d__Bacteria.p__Firmicutes.c__Clostridia.o__Halanaerobiales.f__Halanaerobiaceae.g__Halanaerobium.s__praevalens.t__DSM_2228
d__Bacteria.p__Firmicutes.c__Clostridia.o__Halanaerobiales.f__Halanaerobiaceae.g__Halanaerobium.s__sp_.t__sapolanicus_
d__Bacteria.p__Firmicutes.c__Clostridia.o__Thermoanaerobacterales.f__Thermoanaerobacteraceae.g__Moorella.s__thermoacetica.t__ATCC_39073
d__Bacteria.p__Firmicutes.c__Clostridia.o__Clostridiales.f__Syntrophomonadaceae.g__Syntrophothermus.s__lipocalidus.t__DSM_12680
d__Bacteria.p__Firmicutes.c__Clostridia.o__Clostridiales.f__Syntrophomonadaceae.g__Syntrophomonas.s__wolfei.t__subsp__wolfei_str__Goettingen
d__Bacteria.p__Firmicutes.c__Clostridia.o__Clostridiales.f__Peptococcaceae.g__Thermincola.s__potens.t__JR
d__Bacteria.p__Firmicutes.c__Clostridia.o__Thermoanaerobacterales.f__Thermoanaerobacteraceae.g__Ammonifex.s__degensii.t__KC4
d__Bacteria.p__Firmicutes.c__Clostridia.o__Clostridiales.f__Peptococcaceae.g__Candidatus_Desulforudis.s__audaxviator.t__audaxviator_MP104C
d__Bacteria.p__Firmicutes.c__Clostridia.o__Clostridiales.f__Peptococcaceae.g__Pelotomaculum.s__thermopropionicum.t__SI
d__Bacteria.p__Firmicutes.c__Clostridia.o__Clostridiales.f__Peptococcaceae.g__Desulfotomaculum.s__acetoxidans.t__5575_DSM_771
d__Bacteria.p__Firmicutes.c__Clostridia.o__Clostridiales.f__Peptococcaceae.g__Desulfotomaculum.s__nigrificans.t__DSM_574
d__Bacteria.p__Firmicutes.c__Clostridia.o__Clostridiales.f__Peptococcaceae.g__Desulfotomaculum.s__ruminis.t__DSM_2154
d__Bacteria.p__Firmicutes.c__Clostridia.o__Clostridiales.f__Peptococcaceae.g__Desulfotomaculum.s__reducens.t__MI_1
d__Bacteria.p__Firmicutes.c__Clostridia.o__Thermoanaerobacterales.f__Thermoanaerobacteraceae.g__Carboxydothermus.s__hydrogenoformans.t__Z_2901
d__Bacteria.p__Firmicutes.c__Clostridia.o__Clostridiales.f__Heliobacteriaceae.g__Heliobacterium.s__modesticaldum.t__Ice1
d__Bacteria.p__Firmicutes.c__Clostridia.o__Clostridiales.f__Peptococcaceae.g__Syntrophobotulus.s__glycolicus.t__DSM_8271
d__Bacteria.p__Firmicutes.c__Clostridia.o__Clostridiales.f__Peptococcaceae.g__Desulfitobacterium.s__hafniense.t__Y51
d__Bacteria.p__Firmicutes.c__Clostridia.o__Clostridiales.f__Peptococcaceae.g__Desulfitobacterium.s__hafniense.t__DCB_2
d__Bacteria.p__Firmicutes.c__Negativicutes.o__Selenomonadales.f__Acidaminococcaceae.g__Phascolarctobacterium.s__sp_.t__YIT_12067
d__Bacteria.p__Firmicutes.c__Negativicutes.o__Selenomonadales.f__Acidaminococcaceae.g__Acidaminococcus.s__sp_.t__D21
d__Bacteria.p__Firmicutes.c__Negativicutes.o__Selenomonadales.f__Acidaminococcaceae.g__Acidaminococcus.s__fermentans.t__DSM_20731
d__Bacteria.p__Firmicutes.c__Negativicutes.o__Selenomonadales.f__Veillonellaceae.g__Dialister.s__invisus.t__DSM_15470
d__Bacteria.p__Firmicutes.c__Negativicutes.o__Selenomonadales.f__Veillonellaceae.g__Dialister.s__micraerophilus.t__DSM_19965
d__Bacteria.p__Firmicutes.c__Negativicutes.o__Selenomonadales.f__Veillonellaceae.g__Dialister.s__microaerophilus.t__UPII_345_E
d__Bacteria.p__Firmicutes.c__Negativicutes.o__Selenomonadales.f__Veillonellaceae.g__Veillonella.s__atypica.t__ACS_134_V_Col7a
d__Bacteria.p__Firmicutes.c__Negativicutes.o__Selenomonadales.f__Veillonellaceae.g__Veillonella.s__atypica.t__ACS_049_V_Sch6
d__Bacteria.p__Firmicutes.c__Negativicutes.o__Selenomonadales.f__Veillonellaceae.g__Veillonella.s__dispar.t__ATCC_17748
d__Bacteria.p__Firmicutes.c__Negativicutes.o__Selenomonadales.f__Veillonellaceae.g__Veillonella.s__sp_.t__oral_taxon_158_str__F0412
d__Bacteria.p__Firmicutes.c__Negativicutes.o__Selenomonadales.f__Veillonellaceae.g__Veillonella.s__parvula.t__DSM_2008
d__Bacteria.p__Firmicutes.c__Negativicutes.o__Selenomonadales.f__Veillonellaceae.g__Veillonella.s__parvula.t__ATCC_17745
d__Bacteria.p__Firmicutes.c__Negativicutes.o__Selenomonadales.f__Veillonellaceae.g__Veillonella.s__sp_.t__3_1_44
d__Bacteria.p__Firmicutes.c__Negativicutes.o__Selenomonadales.f__Veillonellaceae.g__Veillonella.s__sp_.t__6_1_27
d__Bacteria.p__Firmicutes.c__Negativicutes.o__Selenomonadales.f__Veillonellaceae.g__Megasphaera.s__micronuciformis.t__F0359
d__Bacteria.p__Firmicutes.c__Negativicutes.o__Selenomonadales.f__Veillonellaceae.g__Megasphaera.s__genomosp_.t__type_1_str__28L
d__Bacteria.p__Firmicutes.c__Negativicutes.o__Selenomonadales.f__Veillonellaceae.g__Megasphaera.s__sp_.t__UPII_199_6
d__Bacteria.p__Firmicutes.c__Negativicutes.o__Selenomonadales.f__Veillonellaceae.g__Megamonas.s__hypermegale.t__1
d__Bacteria.p__Firmicutes.c__Negativicutes.o__Selenomonadales.f__Veillonellaceae.g__Selenomonas.s__sputigena.t__ATCC_35185
d__Bacteria.p__Firmicutes.c__Negativicutes.o__Selenomonadales.f__Veillonellaceae.g__Mitsuokella.s__multacida.t__DSM_20544
d__Bacteria.p__Firmicutes.c__Negativicutes.o__Selenomonadales.f__Veillonellaceae.g__Selenomonas.s__sp_.t__oral_taxon_137_str__F0430
d__Bacteria.p__Firmicutes.c__Negativicutes.o__Selenomonadales.f__Veillonellaceae.g__Selenomonas.s__artemidis.t__F0399
d__Bacteria.p__Firmicutes.c__Negativicutes.o__Selenomonadales.f__Veillonellaceae.g__Selenomonas.s__flueggei.t__ATCC_43531
d__Bacteria.p__Firmicutes.c__Negativicutes.o__Selenomonadales.f__Veillonellaceae.g__Selenomonas.s__sp_.t__oral_taxon_149_str__67H29BP
d__Bacteria.p__Firmicutes.c__Negativicutes.o__Selenomonadales.f__Veillonellaceae.g__Centipeda.s__periodontii.t__DSM_2778
d__Bacteria.p__Firmicutes.c__Negativicutes.o__Selenomonadales.f__Veillonellaceae.g__Selenomonas.s__noxia.t__ATCC_43541
d__Bacteria.p__Firmicutes.c__Negativicutes.o__Selenomonadales.f__Veillonellaceae.g__Thermosinus.s__carboxydivorans.t__Nor1
d__Bacteria.p__Firmicutes.c__Negativicutes.o__Selenomonadales.f__Veillonellaceae.g__Acetonema.s__longum.t__DSM_6540
d__Bacteria.p__Firmicutes.c__Clostridia.o__Natranaerobiales.f__Natranaerobiaceae.g__Natranaerobius.s__thermophilus.t__JW_NM_WN_LF
d__Bacteria.p__Firmicutes.c__Clostridia.o__Clostridiales.f__Syntrophomonadaceae.g__Dethiobacter.s__alkaliphilus.t__AHT_1
d__Bacteria.p__Firmicutes.c__Clostridia.o__Clostridiales.f__Clostridiales_Family_XVIII__Incertae_Sedis.g__Symbiobacterium.s__thermophilum.t__IAM_14863
d__Bacteria.p__Firmicutes.c__Clostridia.o__Clostridiales.f__Clostridiales_Family_XVII__Incertae_Sedis.g__Thermaerobacter.s__marianensis.t__DSM_12885
d__Bacteria.p__Firmicutes.c__Clostridia.o__Clostridiales.f__Clostridiales_Family_XVII__Incertae_Sedis.g__Thermaerobacter.s__subterraneus.t__DSM_13965
d__Bacteria.p__Firmicutes.c__Clostridia.o__Clostridiales.f__Eubacteriaceae.g__Anaerofustis.s__stercorihominis.t__DSM_17244
d__Bacteria.p__Firmicutes.c__Clostridia.o__Clostridiales.f__Eubacteriaceae.g__Eubacterium.s__limosum.t__KIST612
d__Bacteria.p__Firmicutes.c__Clostridia.o__Clostridiales.f__Eubacteriaceae.g__Pseudoramibacter.s__alactolyticus.t__ATCC_23263
d__Bacteria.p__Firmicutes.c__Clostridia.o__Clostridiales.f__Clostridiales_Family_XI__Incertae_Sedis.g__Peptoniphilus.s__duerdenii.t__ATCC_BAA_1640
d__Bacteria.p__Firmicutes.c__Clostridia.o__Clostridiales.f__Clostridiales_Family_XI__Incertae_Sedis.g__Peptoniphilus.s__sp_.t__oral_taxon_386_str__F0131
d__Bacteria.p__Firmicutes.c__Clostridia.o__Clostridiales.f__Clostridiales_Family_XI__Incertae_Sedis.g__Peptoniphilus.s__harei.t__ACS_146_V_Sch2b
d__Bacteria.p__Firmicutes.c__Clostridia.o__Clostridiales.f__Clostridiales_Family_XI__Incertae_Sedis.g__Peptoniphilus.s__sp_.t__oral_taxon_836_str__F0141
d__Bacteria.p__Firmicutes.c__Clostridia.o__Clostridiales.f__Clostridiales_Family_XI__Incertae_Sedis.g__Peptoniphilus.s__lacrimalis.t__315_B
d__Bacteria.p__Firmicutes.c__Clostridia.o__Clostridiales.f__Clostridiales_Family_XI__Incertae_Sedis.g__Parvimonas.s__sp_.t__oral_taxon_110_str__F0139
d__Bacteria.p__Firmicutes.c__Clostridia.o__Clostridiales.f__Clostridiales_Family_XI__Incertae_Sedis.g__Parvimonas.s__micros.t__ATCC_33270
d__Bacteria.p__Firmicutes.c__Clostridia.o__Clostridiales.f__Clostridiales_Family_XI__Incertae_Sedis.g__Finegoldia.s__magna.t__ATCC_53516
d__Bacteria.p__Firmicutes.c__Clostridia.o__Clostridiales.f__Clostridiales_Family_XI__Incertae_Sedis.g__Finegoldia.s__magna.t__BVS033A4
d__Bacteria.p__Firmicutes.c__Clostridia.o__Clostridiales.f__Clostridiales_Family_XI__Incertae_Sedis.g__Finegoldia.s__magna.t__ATCC_29328
d__Bacteria.p__Firmicutes.c__Clostridia.o__Clostridiales.f__Clostridiales_Family_XI__Incertae_Sedis.g__Finegoldia.s__magna.t__ACS_171_V_Col3
d__Bacteria.p__Firmicutes.c__Clostridia.o__Clostridiales.f__Clostridiales_Family_XI__Incertae_Sedis.g__Anaerococcus.s__vaginalis.t__ATCC_51170
d__Bacteria.p__Firmicutes.c__Clostridia.o__Clostridiales.f__Clostridiales_Family_XI__Incertae_Sedis.g__Anaerococcus.s__hydrogenalis.t__DSM_7454
d__Bacteria.p__Firmicutes.c__Clostridia.o__Clostridiales.f__Clostridiales_Family_XI__Incertae_Sedis.g__Anaerococcus.s__hydrogenalis.t__ACS_025_V_Sch4
d__Bacteria.p__Firmicutes.c__Clostridia.o__Clostridiales.f__Clostridiales_Family_XI__Incertae_Sedis.g__Anaerococcus.s__lactolyticus.t__ATCC_51172
d__Bacteria.p__Firmicutes.c__Clostridia.o__Clostridiales.f__Clostridiales_Family_XI__Incertae_Sedis.g__Anaerococcus.s__tetradius.t__ATCC_35098
d__Bacteria.p__Firmicutes.c__Clostridia.o__Clostridiales.f__Clostridiales_Family_XI__Incertae_Sedis.g__Anaerococcus.s__prevotii.t__PC_1__DSM_20548
d__Bacteria.p__Firmicutes.c__Clostridia.o__Clostridiales.f__Eubacteriaceae.g__Eubacterium.s__saphenum.t__ATCC_49989
d__Bacteria.p__Firmicutes.c__Clostridia.o__Clostridiales.f__Clostridiaceae.g__Alkaliphilus.s__metalliredigens.t__QYMF
d__Bacteria.p__Firmicutes.c__Clostridia.o__Clostridiales.f__Clostridiaceae.g__Alkaliphilus.s__oremlandii.t__OhILAs
d__Bacteria.p__Firmicutes.c__Clostridia.o__Clostridiales.f__Clostridiaceae.g__Clostridium.s__bartlettii.t__DSM_16795
d__Bacteria.p__Firmicutes.c__Clostridia.o__Clostridiales.f__Clostridiaceae.g__Clostridium.s__difficile.t__NAP07
d__Bacteria.p__Firmicutes.c__Clostridia.o__Clostridiales.f__Clostridiaceae.g__Clostridium.s__difficile.t__NAP08
d__Bacteria.p__Firmicutes.c__Clostridia.o__Clostridiales.f__Clostridiaceae.g__Clostridium.s__difficile.t__630
d__Bacteria.p__Firmicutes.c__Clostridia.o__Clostridiales.f__Clostridiaceae.g__Clostridium.s__difficile.t__QCD_32g58
d__Bacteria.p__Firmicutes.c__Clostridia.o__Clostridiales.f__Clostridiaceae.g__Clostridium.s__difficile.t__QCD_63q42
d__Bacteria.p__Firmicutes.c__Clostridia.o__Clostridiales.f__Clostridiaceae.g__Clostridium.s__difficile.t__ATCC_43255
d__Bacteria.p__Firmicutes.c__Clostridia.o__Clostridiales.f__Clostridiaceae.g__Clostridium.s__difficile.t__R20291
d__Bacteria.p__Firmicutes.c__Clostridia.o__Clostridiales.f__Clostridiaceae.g__Clostridium.s__difficile.t__CD196
d__Bacteria.p__Firmicutes.c__Clostridia.o__Clostridiales.f__Clostridiaceae.g__Clostridium.s__difficile.t__QCD_66c26
d__Bacteria.p__Firmicutes.c__Clostridia.o__Clostridiales.f__Clostridiaceae.g__Clostridium.s__difficile.t__QCD_76w55
d__Bacteria.p__Firmicutes.c__Clostridia.o__Clostridiales.f__Clostridiaceae.g__Clostridium.s__difficile.t__QCD_97b34
d__Bacteria.p__Firmicutes.c__Clostridia.o__Clostridiales.f__Clostridiaceae.g__Clostridium.s__difficile.t__QCD_37x79
d__Bacteria.p__Firmicutes.c__Clostridia.o__Clostridiales.f__Clostridiaceae.g__Clostridium.s__difficile.t__CIP_107932
d__Bacteria.p__Firmicutes.c__Clostridia.o__Clostridiales.f__Clostridiaceae.g__Clostridium.s__difficile.t__QCD_23m63
d__Bacteria.p__Firmicutes.c__Clostridia.o__Clostridiales.f__Clostridiaceae.g__Clostridium.s__hiranonis.t__DSM_13275
d__Bacteria.p__Firmicutes.c__Clostridia.o__Clostridiales.f__Peptostreptococcaceae.g__Peptostreptococcus.s__stomatis.t__DSM_17678
d__Bacteria.p__Firmicutes.c__Clostridia.o__Clostridiales.f__Peptostreptococcaceae.g__Peptostreptococcus.s__anaerobius.t__653_L
d__Bacteria.p__Firmicutes.c__Clostridia.o__Clostridiales.f__Clostridiaceae.g__Clostridium.s__sticklandii.t__DSM_519
d__Bacteria.p__Firmicutes.c__Clostridia.o__Clostridiales.f__unclassified.g__unclassified.s__yurii.t__subsp__margaretiae_ATCC_43715

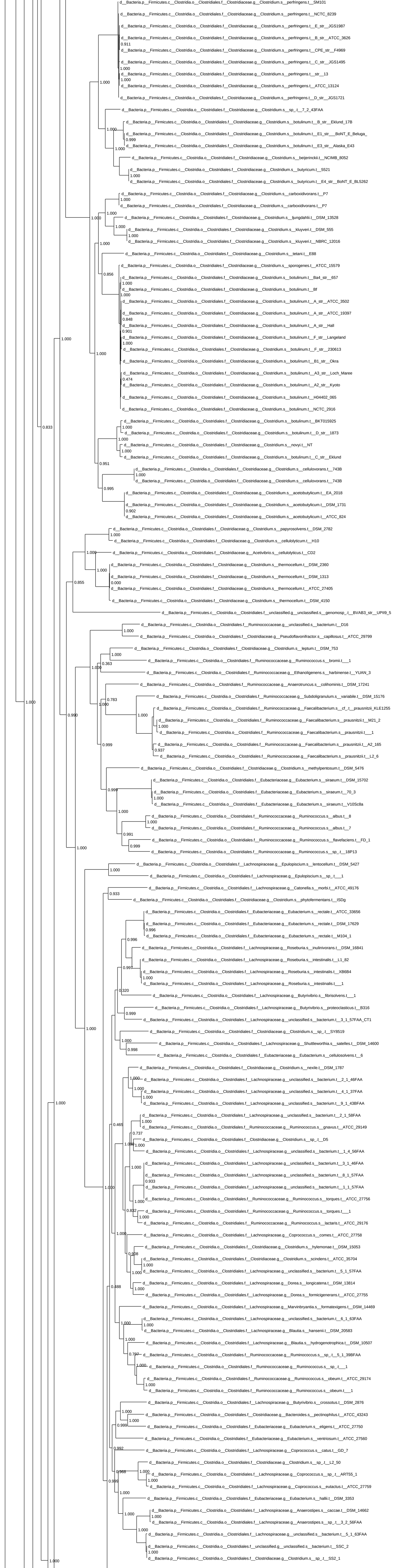

d__Bacteria.p__Firmicutes.c__Clostridia.o__Clostridiales.f__Clostridiaceae.g__Clostridium.s__perfringens.t__SM101
d__Bacteria.p__Firmicutes.c__Clostridia.o__Clostridiales.f__Clostridiaceae.g__Clostridium.s__perfringens.t__NCTC_8239
d__Bacteria.p__Firmicutes.c__Clostridia.o__Clostridiales.f__Clostridiaceae.g__Clostridium.s__perfringens.t__E_str__JGS1987
d__Bacteria.p__Firmicutes.c__Clostridia.o__Clostridiales.f__Clostridiaceae.g__Clostridium.s__perfringens.t__B_str__ATCC_3626
d__Bacteria.p__Firmicutes.c__Clostridia.o__Clostridiales.f__Clostridiaceae.g__Clostridium.s__perfringens.t__CPE_str__F4969
d__Bacteria.p__Firmicutes.c__Clostridia.o__Clostridiales.f__Clostridiaceae.g__Clostridium.s__perfringens.t__C_str__JGS1495
d__Bacteria.p__Firmicutes.c__Clostridia.o__Clostridiales.f__Clostridiaceae.g__Clostridium.s__perfringens.t__str__13
d__Bacteria.p__Firmicutes.c__Clostridia.o__Clostridiales.f__Clostridiaceae.g__Clostridium.s__perfringens.t__ATCC_13124
d__Bacteria.p__Firmicutes.c__Clostridia.o__Clostridiales.f__Clostridiaceae.g__Clostridium.s__perfringens.t__D_str__JGS1721
d__Bacteria.p__Firmicutes.c__Clostridia.o__Clostridiales.f__Clostridiaceae.g__Clostridium.s__sp_t__7_2_43FAA
d__Bacteria.p__Firmicutes.c__Clostridia.o__Clostridiales.f__Clostridiaceae.g__Clostridium.s__botulinum.t__B_str__Eklund_17B
d__Bacteria.p__Firmicutes.c__Clostridia.o__Clostridiales.f__Clostridiaceae.g__Clostridium.s__botulinum.t__E1_str___BoNT_E_Beluga_
d__Bacteria.p__Firmicutes.c__Clostridia.o__Clostridiales.f__Clostridiaceae.g__Clostridium.s__botulinum.t__E3_str__Alaska_E43
d__Bacteria.p__Firmicutes.c__Clostridia.o__Clostridiales.f__Clostridiaceae.g__Clostridium.s__beijerinckii.t__NCIMB_8052
d__Bacteria.p__Firmicutes.c__Clostridia.o__Clostridiales.f__Clostridiaceae.g__Clostridium.s__butyricum.t__5521
d__Bacteria.p__Firmicutes.c__Clostridia.o__Clostridiales.f__Clostridiaceae.g__Clostridium.s__butyricum.t__E4_str__BoNT_E_BL5262
d__Bacteria.p__Firmicutes.c__Clostridia.o__Clostridiales.f__Clostridiaceae.g__Clostridium.s__carboxidivorans.t__P7
d__Bacteria.p__Firmicutes.c__Clostridia.o__Clostridiales.f__Clostridiaceae.g__Clostridium.s__carboxidivorans.t__P7
d__Bacteria.p__Firmicutes.c__Clostridia.o__Clostridiales.f__Clostridiaceae.g__Clostridium.s__ljungdahlii.t__DSM_13528
d__Bacteria.p__Firmicutes.c__Clostridia.o__Clostridiales.f__Clostridiaceae.g__Clostridium.s__kluyveri.t__DSM_555
d__Bacteria.p__Firmicutes.c__Clostridia.o__Clostridiales.f__Clostridiaceae.g__Clostridium.s__kluyveri.t__NBRC_12016
d__Bacteria.p__Firmicutes.c__Clostridia.o__Clostridiales.f__Clostridiaceae.g__Clostridium.s__tetani.t__E88
d__Bacteria.p__Firmicutes.c__Clostridia.o__Clostridiales.f__Clostridiaceae.g__Clostridium.s__sporogenes.t__ATCC_15579
d__Bacteria.p__Firmicutes.c__Clostridia.o__Clostridiales.f__Clostridiaceae.g__Clostridium.s__botulinum.t__Ba4_str__657
d__Bacteria.p__Firmicutes.c__Clostridia.o__Clostridiales.f__Clostridiaceae.g__Clostridium.s__botulinum.t__Bf
d__Bacteria.p__Firmicutes.c__Clostridia.o__Clostridiales.f__Clostridiaceae.g__Clostridium.s__botulinum.t__A_str__ATCC_3502
d__Bacteria.p__Firmicutes.c__Clostridia.o__Clostridiales.f__Clostridiaceae.g__Clostridium.s__botulinum.t__A_str__ATCC_19397
d__Bacteria.p__Firmicutes.c__Clostridia.o__Clostridiales.f__Clostridiaceae.g__Clostridium.s__botulinum.t__A_str__Hall
d__Bacteria.p__Firmicutes.c__Clostridia.o__Clostridiales.f__Clostridiaceae.g__Clostridium.s__botulinum.t__F_str__Langeland
d__Bacteria.p__Firmicutes.c__Clostridia.o__Clostridiales.f__Clostridiaceae.g__Clostridium.s__botulinum.t__F_str__230613
d__Bacteria.p__Firmicutes.c__Clostridia.o__Clostridiales.f__Clostridiaceae.g__Clostridium.s__botulinum.t__B1_str__Okra
d__Bacteria.p__Firmicutes.c__Clostridia.o__Clostridiales.f__Clostridiaceae.g__Clostridium.s__botulinum.t__A3_str__Loch_Maree
d__Bacteria.p__Firmicutes.c__Clostridia.o__Clostridiales.f__Clostridiaceae.g__Clostridium.s__botulinum.t__A2_str__Kyoto
d__Bacteria.p__Firmicutes.c__Clostridia.o__Clostridiales.f__Clostridiaceae.g__Clostridium.s__botulinum.t__H04402_065
d__Bacteria.p__Firmicutes.c__Clostridia.o__Clostridiales.f__Clostridiaceae.g__Clostridium.s__botulinum.t__NCTC_2916
d__Bacteria.p__Firmicutes.c__Clostridia.o__Clostridiales.f__Clostridiaceae.g__Clostridium.s__botulinum.t__BKT015925
d__Bacteria.p__Firmicutes.c__Clostridia.o__Clostridiales.f__Clostridiaceae.g__Clostridium.s__botulinum.t__D_str__1873
d__Bacteria.p__Firmicutes.c__Clostridia.o__Clostridiales.f__Clostridiaceae.g__Clostridium.s__novyi.t__NT
d__Bacteria.p__Firmicutes.c__Clostridia.o__Clostridiales.f__Clostridiaceae.g__Clostridium.s__botulinum.t__C_str__Eklund
d__Bacteria.p__Firmicutes.c__Clostridia.o__Clostridiales.f__Clostridiaceae.g__Clostridium.s__cellulovorans.t__743B
d__Bacteria.p__Firmicutes.c__Clostridia.o__Clostridiales.f__Clostridiaceae.g__Clostridium.s__cellulovorans.t__743B
d__Bacteria.p__Firmicutes.c__Clostridia.o__Clostridiales.f__Clostridiaceae.g__Clostridium.s__acetobutylicum.t__EA_2018
d__Bacteria.p__Firmicutes.c__Clostridia.o__Clostridiales.f__Clostridiaceae.g__Clostridium.s__acetobutylicum.t__DSM_1731
d__Bacteria.p__Firmicutes.c__Clostridia.o__Clostridiales.f__Clostridiaceae.g__Clostridium.s__acetobutylicum.t__ATCC_824
d__Bacteria.p__Firmicutes.c__Clostridia.o__Clostridiales.f__Clostridiaceae.g__Clostridium.s__papyrosolvens.t__DSM_2782
d__Bacteria.p__Firmicutes.c__Clostridia.o__Clostridiales.f__Clostridiaceae.g__Clostridium.s__cellulolyticum.t__H10
d__Bacteria.p__Firmicutes.c__Clostridia.o__Clostridiales.f__Clostridiaceae.g__Acetivibrio.s__cellulolyticus.t__CD2
d__Bacteria.p__Firmicutes.c__Clostridia.o__Clostridiales.f__Clostridiaceae.g__Clostridium.s__thermocellum.t__DSM_2360
d__Bacteria.p__Firmicutes.c__Clostridia.o__Clostridiales.f__Clostridiaceae.g__Clostridium.s__thermocellum.t__DSM_1313
d__Bacteria.p__Firmicutes.c__Clostridia.o__Clostridiales.f__Clostridiaceae.g__Clostridium.s__thermocellum.t__ATCC_27405
d__Bacteria.p__Firmicutes.c__Clostridia.o__Clostridiales.f__Clostridiaceae.g__Clostridium.s__thermocellum.t__DSM_4150
d__Bacteria.p__Firmicutes.c__Clostridia.o__Clostridiales.f__unclassified.g__unclassified.s__genomosp_t__BVAB3_str__UPII9_5
d__Bacteria.p__Firmicutes.c__Clostridia.o__Clostridiales.f__Ruminococcaceae.g__unclassified.s__bacterium.t__D16
d__Bacteria.p__Firmicutes.c__Clostridia.o__Clostridiales.f__Clostridiaceae.g__Pseudoflavonifractor.s__capillosus.t__ATCC_29799
d__Bacteria.p__Firmicutes.c__Clostridia.o__Clostridiales.f__Clostridiaceae.g__Clostridium.s__leptum.t__DSM_753
d__Bacteria.p__Firmicutes.c__Clostridia.o__Clostridiales.f__Ruminococcaceae.g__Ruminococcus.s__bromii.t___1
d__Bacteria.p__Firmicutes.c__Clostridia.o__Clostridiales.f__Ruminococcaceae.g__Ethanoligenens.s__harbinense.t__YUAN_3
d__Bacteria.p__Firmicutes.c__Clostridia.o__Clostridiales.f__Ruminococcaceae.g__Anaerotruncus.s__colihominis.t__DSM_17241
d__Bacteria.p__Firmicutes.c__Clostridia.o__Clostridiales.f__Ruminococcaceae.g__Subdoligranulum.s__variabile.t__DSM_15176
d__Bacteria.p__Firmicutes.c__Clostridia.o__Clostridiales.f__Ruminococcaceae.g__Faecalibacterium.s__cf_t__prausnitzii_KLE1255
d__Bacteria.p__Firmicutes.c__Clostridia.o__Clostridiales.f__Ruminococcaceae.g__Faecalibacterium.s__prausnitzii.t__M21_2
d__Bacteria.p__Firmicutes.c__Clostridia.o__Clostridiales.f__Ruminococcaceae.g__Faecalibacterium.s__prausnitzii.t___1
d__Bacteria.p__Firmicutes.c__Clostridia.o__Clostridiales.f__Ruminococcaceae.g__Faecalibacterium.s__prausnitzii.t__A2_165
d__Bacteria.p__Firmicutes.c__Clostridia.o__Clostridiales.f__Ruminococcaceae.g__Faecalibacterium.s__prausnitzii.t__L2_6
d__Bacteria.p__Firmicutes.c__Clostridia.o__Clostridiales.f__Clostridiaceae.g__Clostridium.s__methylpentosum.t__DSM_5476
d__Bacteria.p__Firmicutes.c__Clostridia.o__Clostridiales.f__Eubacteriaceae.g__Eubacterium.s__siraeum.t__DSM_15702
d__Bacteria.p__Firmicutes.c__Clostridia.o__Clostridiales.f__Eubacteriaceae.g__Eubacterium.s__siraeum.t__70_3
d__Bacteria.p__Firmicutes.c__Clostridia.o__Clostridiales.f__Eubacteriaceae.g__Eubacterium.s__siraeum.t__V10Sc8a
d__Bacteria.p__Firmicutes.c__Clostridia.o__Clostridiales.f__Ruminococcaceae.g__Ruminococcus.s__albus.t__8
d__Bacteria.p__Firmicutes.c__Clostridia.o__Clostridiales.f__Ruminococcaceae.g__Ruminococcus.s__albus.t__7
d__Bacteria.p__Firmicutes.c__Clostridia.o__Clostridiales.f__Ruminococcaceae.g__Ruminococcus.s__flavefaciens.t__FD_1
d__Bacteria.p__Firmicutes.c__Clostridia.o__Clostridiales.f__Ruminococcaceae.g__Ruminococcus.s__sp_t__18P13
d__Bacteria.p__Firmicutes.c__Clostridia.o__Clostridiales.f__Lachnospiraceae.g__Epulopiscium.s__lentocellum.t__DSM_5427
d__Bacteria.p__Firmicutes.c__Clostridia.o__Clostridiales.f__Lachnospiraceae.g__Epulopiscium.s__sp_t___1
d__Bacteria.p__Firmicutes.c__Clostridia.o__Clostridiales.f__Lachnospiraceae.g__Catonella.s__morbi.t__ATCC_49176
d__Bacteria.p__Firmicutes.c__Clostridia.o__Clostridiales.f__Clostridiaceae.g__Clostridium.s__phytofermentans.t__ISDg
d__Bacteria.p__Firmicutes.c__Clostridia.o__Clostridiales.f__Eubacteriaceae.g__Eubacterium.s__rectale.t__ATCC_33656
d__Bacteria.p__Firmicutes.c__Clostridia.o__Clostridiales.f__Eubacteriaceae.g__Eubacterium.s__rectale.t__DSM_17629
d__Bacteria.p__Firmicutes.c__Clostridia.o__Clostridiales.f__Eubacteriaceae.g__Eubacterium.s__rectale.t__M104_1
d__Bacteria.p__Firmicutes.c__Clostridia.o__Clostridiales.f__Lachnospiraceae.g__Roseburia.s__inulinivorans.t__DSM_16841
d__Bacteria.p__Firmicutes.c__Clostridia.o__Clostridiales.f__Lachnospiraceae.g__Roseburia.s__intestinalis.t__L1_82
d__Bacteria.p__Firmicutes.c__Clostridia.o__Clostridiales.f__Lachnospiraceae.g__Roseburia.s__intestinalis.t__XB6B4
d__Bacteria.p__Firmicutes.c__Clostridia.o__Clostridiales.f__Lachnospiraceae.g__Roseburia.s__intestinalis.t___1
d__Bacteria.p__Firmicutes.c__Clostridia.o__Clostridiales.f__Lachnospiraceae.g__Butyrivibrio.s__fibrisolvens.t___1
d__Bacteria.p__Firmicutes.c__Clostridia.o__Clostridiales.f__Lachnospiraceae.g__Butyrivibrio.s__proteoclasticus.t__B316
d__Bacteria.p__Firmicutes.c__Clostridia.o__Clostridiales.f__Lachnospiraceae.g__unclassified.s__bacterium.t__3_1_57FAA_CT1
d__Bacteria.p__Firmicutes.c__Clostridia.o__Clostridiales.f__Clostridiaceae.g__Clostridium.s__sp_t__SY8519
d__Bacteria.p__Firmicutes.c__Clostridia.o__Clostridiales.f__Lachnospiraceae.g__Shuttleworthia.s__satelles.t__DSM_14600
d__Bacteria.p__Firmicutes.c__Clostridia.o__Clostridiales.f__Eubacteriaceae.g__Eubacterium.s__cellulosolvens.t__6
d__Bacteria.p__Firmicutes.c__Clostridia.o__Clostridiales.f__Clostridiaceae.g__Clostridium.s__nexile.t__DSM_1787
d__Bacteria.p__Firmicutes.c__Clostridia.o__Clostridiales.f__Lachnospiraceae.g__unclassified.s__bacterium.t__2_1_46FAA
d__Bacteria.p__Firmicutes.c__Clostridia.o__Clostridiales.f__Lachnospiraceae.g__unclassified.s__bacterium.t__4_1_37FAA
d__Bacteria.p__Firmicutes.c__Clostridia.o__Clostridiales.f__Lachnospiraceae.g__unclassified.s__bacterium.t__9_1_43BFAA
d__Bacteria.p__Firmicutes.c__Clostridia.o__Clostridiales.f__Lachnospiraceae.g__unclassified.s__bacterium.t__2_1_58FAA
d__Bacteria.p__Firmicutes.c__Clostridia.o__Clostridiales.f__Ruminococcaceae.g__Ruminococcus.s__gnavus.t__ATCC_29149
d__Bacteria.p__Firmicutes.c__Clostridia.o__Clostridiales.f__Clostridiaceae.g__Clostridium.s__sp_t__D5
d__Bacteria.p__Firmicutes.c__Clostridia.o__Clostridiales.f__Lachnospiraceae.g__unclassified.s__bacterium.t__1_4_56FAA
d__Bacteria.p__Firmicutes.c__Clostridia.o__Clostridiales.f__Lachnospiraceae.g__unclassified.s__bacterium.t__3_1_46FAA
d__Bacteria.p__Firmicutes.c__Clostridia.o__Clostridiales.f__Lachnospiraceae.g__unclassified.s__bacterium.t__8_1_57FAA
d__Bacteria.p__Firmicutes.c__Clostridia.o__Clostridiales.f__Lachnospiraceae.g__unclassified.s__bacterium.t__1_1_57FAA
d__Bacteria.p__Firmicutes.c__Clostridia.o__Clostridiales.f__Ruminococcaceae.g__Ruminococcus.s__torques.t__ATCC_27756
d__Bacteria.p__Firmicutes.c__Clostridia.o__Clostridiales.f__Ruminococcaceae.g__Ruminococcus.s__torques.t___1
d__Bacteria.p__Firmicutes.c__Clostridia.o__Clostridiales.f__Ruminococcaceae.g__Ruminococcus.s__lactaris.t__ATCC_29176
d__Bacteria.p__Firmicutes.c__Clostridia.o__Clostridiales.f__Lachnospiraceae.g__Coprococcus.s__comes.t__ATCC_27758
d__Bacteria.p__Firmicutes.c__Clostridia.o__Clostridiales.f__Clostridiaceae.g__Clostridium.s__hylemonae.t__DSM_15053
d__Bacteria.p__Firmicutes.c__Clostridia.o__Clostridiales.f__Clostridiaceae.g__Clostridium.s__scindens.t__ATCC_35704
d__Bacteria.p__Firmicutes.c__Clostridia.o__Clostridiales.f__Lachnospiraceae.g__unclassified.s__bacterium.t__5_1_57FAA
d__Bacteria.p__Firmicutes.c__Clostridia.o__Clostridiales.f__Lachnospiraceae.g__Dorea.s__longicatena.t__DSM_13814
d__Bacteria.p__Firmicutes.c__Clostridia.o__Clostridiales.f__Lachnospiraceae.g__Dorea.s__formicigenerans.t__ATCC_27755
d__Bacteria.p__Firmicutes.c__Clostridia.o__Clostridiales.f__Lachnospiraceae.g__Marvinbryantia.s__formatexigens.t__DSM_14469
d__Bacteria.p__Firmicutes.c__Clostridia.o__Clostridiales.f__Lachnospiraceae.g__unclassified.s__bacterium.t__6_1_63FAA
d__Bacteria.p__Firmicutes.c__Clostridia.o__Clostridiales.f__Lachnospiraceae.g__Blautia.s__hansenii.t__DSM_20583
d__Bacteria.p__Firmicutes.c__Clostridia.o__Clostridiales.f__Lachnospiraceae.g__Blautia.s__hydrogenotrophica.t__DSM_10507
d__Bacteria.p__Firmicutes.c__Clostridia.o__Clostridiales.f__Ruminococcaceae.g__Ruminococcus.s__sp_t__5_1_39BFAA
d__Bacteria.p__Firmicutes.c__Clostridia.o__Clostridiales.f__Ruminococcaceae.g__Ruminococcus.s__sp_t___1
d__Bacteria.p__Firmicutes.c__Clostridia.o__Clostridiales.f__Ruminococcaceae.g__Ruminococcus.s__obeum.t__ATCC_29174
d__Bacteria.p__Firmicutes.c__Clostridia.o__Clostridiales.f__Ruminococcaceae.g__Ruminococcus.s__obeum.t___1
d__Bacteria.p__Firmicutes.c__Clostridia.o__Clostridiales.f__Lachnospiraceae.g__Butyrivibrio.s__crossotus.t__DSM_2876
d__Bacteria.p__Firmicutes.c__Clostridia.o__Clostridiales.f__Clostridiaceae.g__Bacteroides.s__pectinophilus.t__ATCC_43243
d__Bacteria.p__Firmicutes.c__Clostridia.o__Clostridiales.f__Eubacteriaceae.g__Eubacterium.s__eligens.t__ATCC_27750
d__Bacteria.p__Firmicutes.c__Clostridia.o__Clostridiales.f__Eubacteriaceae.g__Eubacterium.s__ventriosum.t__ATCC_27560
d__Bacteria.p__Firmicutes.c__Clostridia.o__Clostridiales.f__Lachnospiraceae.g__Coprococcus.s__catus.t__GD_7
d__Bacteria.p__Firmicutes.c__Clostridia.o__Clostridiales.f__Clostridiaceae.g__Clostridium.s__sp_t__L2_50
d__Bacteria.p__Firmicutes.c__Clostridia.o__Clostridiales.f__Lachnospiraceae.g__Coprococcus.s__sp_t__ART55_1
d__Bacteria.p__Firmicutes.c__Clostridia.o__Clostridiales.f__Lachnospiraceae.g__Coprococcus.s__eutactus.t__ATCC_27759
d__Bacteria.p__Firmicutes.c__Clostridia.o__Clostridiales.f__Eubacteriaceae.g__Eubacterium.s__hallii.t__DSM_3353
d__Bacteria.p__Firmicutes.c__Clostridia.o__Clostridiales.f__Lachnospiraceae.g__Anaerostipes.s__caccae.t__DSM_14662
d__Bacteria.p__Firmicutes.c__Clostridia.o__Clostridiales.f__Lachnospiraceae.g__Anaerostipes.s__sp_t__3_2_56FAA
d__Bacteria.p__Firmicutes.c__Clostridia.o__Clostridiales.f__Lachnospiraceae.g__unclassified.s__bacterium.t__5_1_63FAA
d__Bacteria.p__Firmicutes.c__Clostridia.o__Clostridiales.f__unclassified.g__unclassified.s__bacterium.t__SSC_2
d__Bacteria.p__Firmicutes.c__Clostridia.o__Clostridiales.f__Clostridiaceae.g__Clostridium.s__sp_t__SS2_1

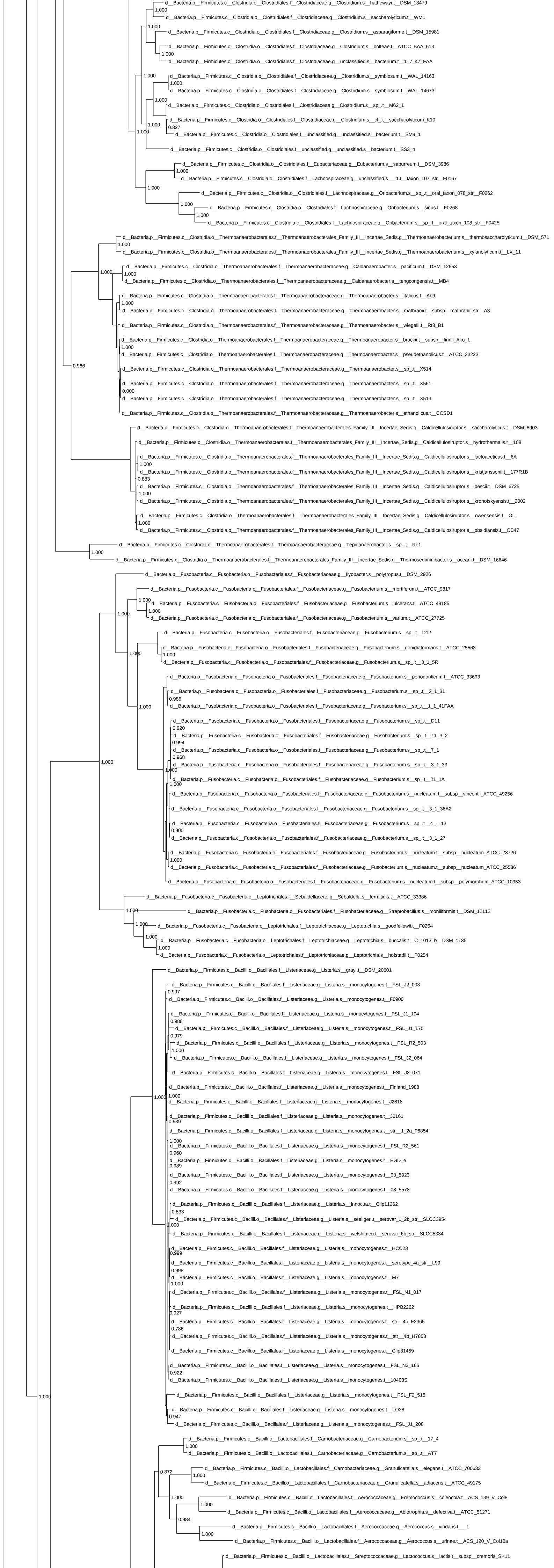

d__Bacteria.p__Firmicutes.c__Clostridia.o__Clostridiales.f__Clostridiaceae.g__Clostridium.s__hathewayi.t__DSM_13479
1.000
d__Bacteria.p__Firmicutes.c__Clostridia.o__Clostridiales.f__Clostridiaceae.g__Clostridium.s__saccharolyticum.t__WM1
1.000
d__Bacteria.p__Firmicutes.c__Clostridia.o__Clostridiales.f__Clostridiaceae.g__Clostridium.s__asparagiforme.t__DSM_15981
d__Bacteria.p__Firmicutes.c__Clostridia.o__Clostridiales.f__Clostridiaceae.g__Clostridium.s__bolteae.t__ATCC_BAA_613
1.000
d__Bacteria.p__Firmicutes.c__Clostridia.o__Clostridiales.f__Clostridiaceae.g__unclassified.s__bacterium.t__1_7_47_FAA
1.000
d__Bacteria.p__Firmicutes.c__Clostridia.o__Clostridiales.f__Clostridiaceae.g__Clostridium.s__symbiosum.t__WAL_14163
1.000
d__Bacteria.p__Firmicutes.c__Clostridia.o__Clostridiales.f__Clostridiaceae.g__Clostridium.s__symbiosum.t__WAL_14673
1.000
d__Bacteria.p__Firmicutes.c__Clostridia.o__Clostridiales.f__Clostridiaceae.g__Clostridium.s__sp_.t__M62_1
1.000
d__Bacteria.p__Firmicutes.c__Clostridia.o__Clostridiales.f__Clostridiaceae.g__Clostridium.s__cf_.t__saccharolyticum_K10
0.827
1.000
d__Bacteria.p__Firmicutes.c__Clostridia.o__Clostridiales.f__unclassified.g__unclassified.s__bacterium.t__SM4_1
d__Bacteria.p__Firmicutes.c__Clostridia.o__Clostridiales.f__unclassified.g__unclassified.s__bacterium.t__SS3_4
d__Bacteria.p__Firmicutes.c__Clostridia.o__Clostridiales.f__Eubacteriaceae.g__Eubacterium.s__saburreum.t__DSM_3986
1.000
d__Bacteria.p__Firmicutes.c__Clostridia.o__Clostridiales.f__Lachnospiraceae.g__unclassified.s___1.t__taxon_107_str__F0167
1.000
d__Bacteria.p__Firmicutes.c__Clostridia.o__Clostridiales.f__Lachnospiraceae.g__Oribacterium.s__sp_.t__oral_taxon_078_str__F0262
1.000
d__Bacteria.p__Firmicutes.c__Clostridia.o__Clostridiales.f__Lachnospiraceae.g__Oribacterium.s__sinus.t__F0268
1.000
d__Bacteria.p__Firmicutes.c__Clostridia.o__Clostridiales.f__Lachnospiraceae.g__Oribacterium.s__sp_.t__oral_taxon_108_str__F0425
d__Bacteria.p__Firmicutes.c__Clostridia.o__Thermoanaerobacterales.f__Thermoanaerobacterales_Family_III__Incertae_Sedis.g__Thermoanaerobacterium.s__thermosaccharolyticum.t__DSM_571
1.000
d__Bacteria.p__Firmicutes.c__Clostridia.o__Thermoanaerobacterales.f__Thermoanaerobacterales_Family_III__Incertae_Sedis.g__Thermoanaerobacterium.s__xylanolyticum.t__LX_11
d__Bacteria.p__Firmicutes.c__Clostridia.o__Thermoanaerobacterales.f__Thermoanaerobacteraceae.g__Caldanaerobacter.s__pacificum.t__DSM_12653
1.000
1.000
d__Bacteria.p__Firmicutes.c__Clostridia.o__Thermoanaerobacterales.f__Thermoanaerobacteraceae.g__Caldanaerobacter.s__tengcongensis.t__MB4
d__Bacteria.p__Firmicutes.c__Clostridia.o__Thermoanaerobacterales.f__Thermoanaerobacteraceae.g__Thermoanaerobacter.s__italicus.t__Ab9
1.000
d__Bacteria.p__Firmicutes.c__Clostridia.o__Thermoanaerobacterales.f__Thermoanaerobacteraceae.g__Thermoanaerobacter.s__mathranii.t__subsp__mathranii_str__A3
d__Bacteria.p__Firmicutes.c__Clostridia.o__Thermoanaerobacterales.f__Thermoanaerobacteraceae.g__Thermoanaerobacter.s__wiegelii.t__Rt8_B1
d__Bacteria.p__Firmicutes.c__Clostridia.o__Thermoanaerobacterales.f__Thermoanaerobacteraceae.g__Thermoanaerobacter.s__brockii.t__subsp__finnii_Ako_1
1.000
d__Bacteria.p__Firmicutes.c__Clostridia.o__Thermoanaerobacterales.f__Thermoanaerobacteraceae.g__Thermoanaerobacter.s__pseudethanolicus.t__ATCC_33223
0.966
d__Bacteria.p__Firmicutes.c__Clostridia.o__Thermoanaerobacterales.f__Thermoanaerobacteraceae.g__Thermoanaerobacter.s__sp_.t__X514
d__Bacteria.p__Firmicutes.c__Clostridia.o__Thermoanaerobacterales.f__Thermoanaerobacteraceae.g__Thermoanaerobacter.s__sp_.t__X561
0.000
d__Bacteria.p__Firmicutes.c__Clostridia.o__Thermoanaerobacterales.f__Thermoanaerobacteraceae.g__Thermoanaerobacter.s__sp_.t__X513
d__Bacteria.p__Firmicutes.c__Clostridia.o__Thermoanaerobacterales.f__Thermoanaerobacteraceae.g__Thermoanaerobacter.s__ethanolicus.t__CCSD1
d__Bacteria.p__Firmicutes.c__Clostridia.o__Thermoanaerobacterales.f__Thermoanaerobacterales_Family_III__Incertae_Sedis.g__Caldicellulosiruptor.s__saccharolyticus.t__DSM_8903
d__Bacteria.p__Firmicutes.c__Clostridia.o__Thermoanaerobacterales.f__Thermoanaerobacterales_Family_III__Incertae_Sedis.g__Caldicellulosiruptor.s__hydrothermalis.t__108
d__Bacteria.p__Firmicutes.c__Clostridia.o__Thermoanaerobacterales.f__Thermoanaerobacterales_Family_III__Incertae_Sedis.g__Caldicellulosiruptor.s__lactoaceticus.t__6A
1.000
d__Bacteria.p__Firmicutes.c__Clostridia.o__Thermoanaerobacterales.f__Thermoanaerobacterales_Family_III__Incertae_Sedis.g__Caldicellulosiruptor.s__kristjanssonii.t__177R1B
0.883
d__Bacteria.p__Firmicutes.c__Clostridia.o__Thermoanaerobacterales.f__Thermoanaerobacterales_Family_III__Incertae_Sedis.g__Caldicellulosiruptor.s__bescii.t__DSM_6725
1.000
d__Bacteria.p__Firmicutes.c__Clostridia.o__Thermoanaerobacterales.f__Thermoanaerobacterales_Family_III__Incertae_Sedis.g__Caldicellulosiruptor.s__kronotskyensis.t__2002
d__Bacteria.p__Firmicutes.c__Clostridia.o__Thermoanaerobacterales.f__Thermoanaerobacterales_Family_III__Incertae_Sedis.g__Caldicellulosiruptor.s__owensensis.t__OL
1.000
d__Bacteria.p__Firmicutes.c__Clostridia.o__Thermoanaerobacterales.f__Thermoanaerobacterales_Family_III__Incertae_Sedis.g__Caldicellulosiruptor.s__obsidiansis.t__OB47
d__Bacteria.p__Firmicutes.c__Clostridia.o__Thermoanaerobacterales.f__Thermoanaerobacteraceae.g__Tepidanaerobacter.s__sp_.t__Re1
1.000
d__Bacteria.p__Firmicutes.c__Clostridia.o__Thermoanaerobacterales.f__Thermoanaerobacterales_Family_III__Incertae_Sedis.g__Thermosediminibacter.s__oceani.t__DSM_16646
d__Bacteria.p__Fusobacteria.c__Fusobacteria.o__Fusobacteriales.f__Fusobacteriaceae.g__Ilyobacter.s__polytropus.t__DSM_2926
d__Bacteria.p__Fusobacteria.c__Fusobacteria.o__Fusobacteriales.f__Fusobacteriaceae.g__Fusobacterium.s__mortiferum.t__ATCC_9817
1.000
d__Bacteria.p__Fusobacteria.c__Fusobacteria.o__Fusobacteriales.f__Fusobacteriaceae.g__Fusobacterium.s__ulcerans.t__ATCC_49185
1.000
1.000
d__Bacteria.p__Fusobacteria.c__Fusobacteria.o__Fusobacteriales.f__Fusobacteriaceae.g__Fusobacterium.s__varium.t__ATCC_27725
d__Bacteria.p__Fusobacteria.c__Fusobacteria.o__Fusobacteriales.f__Fusobacteriaceae.g__Fusobacterium.s__sp_.t__D12
d__Bacteria.p__Fusobacteria.c__Fusobacteria.o__Fusobacteriales.f__Fusobacteriaceae.g__Fusobacterium.s__gonidiaformans.t__ATCC_25563
1.000
1.000
d__Bacteria.p__Fusobacteria.c__Fusobacteria.o__Fusobacteriales.f__Fusobacteriaceae.g__Fusobacterium.s__sp_.t__3_1_5R
d__Bacteria.p__Fusobacteria.c__Fusobacteria.o__Fusobacteriales.f__Fusobacteriaceae.g__Fusobacterium.s__periodonticum.t__ATCC_33693
d__Bacteria.p__Fusobacteria.c__Fusobacteria.o__Fusobacteriales.f__Fusobacteriaceae.g__Fusobacterium.s__sp_.t__2_1_31
0.985
d__Bacteria.p__Fusobacteria.c__Fusobacteria.o__Fusobacteriales.f__Fusobacteriaceae.g__Fusobacterium.s__sp_.t__1_1_41FAA
1.000
d__Bacteria.p__Fusobacteria.c__Fusobacteria.o__Fusobacteriales.f__Fusobacteriaceae.g__Fusobacterium.s__sp_.t__D11
0.920
d__Bacteria.p__Fusobacteria.c__Fusobacteria.o__Fusobacteriales.f__Fusobacteriaceae.g__Fusobacterium.s__sp_.t__11_3_2
0.994
d__Bacteria.p__Fusobacteria.c__Fusobacteria.o__Fusobacteriales.f__Fusobacteriaceae.g__Fusobacterium.s__sp_.t__7_1
0.968
d__Bacteria.p__Fusobacteria.c__Fusobacteria.o__Fusobacteriales.f__Fusobacteriaceae.g__Fusobacterium.s__sp_.t__3_1_33
1.000
1.000
d__Bacteria.p__Fusobacteria.c__Fusobacteria.o__Fusobacteriales.f__Fusobacteriaceae.g__Fusobacterium.s__sp_.t__21_1A
1.000
d__Bacteria.p__Fusobacteria.c__Fusobacteria.o__Fusobacteriales.f__Fusobacteriaceae.g__Fusobacterium.s__nucleatum.t__subsp__vincentii_ATCC_49256
d__Bacteria.p__Fusobacteria.c__Fusobacteria.o__Fusobacteriales.f__Fusobacteriaceae.g__Fusobacterium.s__sp_.t__3_1_36A2
d__Bacteria.p__Fusobacteria.c__Fusobacteria.o__Fusobacteriales.f__Fusobacteriaceae.g__Fusobacterium.s__sp_.t__4_1_13
0.900
d__Bacteria.p__Fusobacteria.c__Fusobacteria.o__Fusobacteriales.f__Fusobacteriaceae.g__Fusobacterium.s__sp_.t__3_1_27
d__Bacteria.p__Fusobacteria.c__Fusobacteria.o__Fusobacteriales.f__Fusobacteriaceae.g__Fusobacterium.s__nucleatum.t__subsp__nucleatum_ATCC_23726
1.000
d__Bacteria.p__Fusobacteria.c__Fusobacteria.o__Fusobacteriales.f__Fusobacteriaceae.g__Fusobacterium.s__nucleatum.t__subsp__nucleatum_ATCC_25586
d__Bacteria.p__Fusobacteria.c__Fusobacteria.o__Fusobacteriales.f__Fusobacteriaceae.g__Fusobacterium.s__nucleatum.t__subsp__polymorphum_ATCC_10953
d__Bacteria.p__Fusobacteria.c__Fusobacteria.o__Leptotrichales.f__Sebaldellaceae.g__Sebaldella.s__termitidis.t__ATCC_33386
1.000
d__Bacteria.p__Fusobacteria.c__Fusobacteria.o__Fusobacteriales.f__Fusobacteriaceae.g__Streptobacillus.s__moniliformis.t__DSM_12112
1.000
d__Bacteria.p__Fusobacteria.c__Fusobacteria.o__Leptotrichales.f__Leptotrichiaceae.g__Leptotrichia.s__goodfellowii.t__F0264
1.000
d__Bacteria.p__Fusobacteria.c__Fusobacteria.o__Leptotrichales.f__Leptotrichiaceae.g__Leptotrichia.s__buccalis.t__C_1013_b__DSM_1135
1.000
d__Bacteria.p__Fusobacteria.c__Fusobacteria.o__Leptotrichales.f__Leptotrichiaceae.g__Leptotrichia.s__hofstadii.t__F0254
d__Bacteria.p__Firmicutes.c__Bacilli.o__Bacillales.f__Listeriaceae.g__Listeria.s__grayi.t__DSM_20601
d__Bacteria.p__Firmicutes.c__Bacilli.o__Bacillales.f__Listeriaceae.g__Listeria.s__monocytogenes.t__FSL_J2_003
0.997
d__Bacteria.p__Firmicutes.c__Bacilli.o__Bacillales.f__Listeriaceae.g__Listeria.s__monocytogenes.t__F6900
d__Bacteria.p__Firmicutes.c__Bacilli.o__Bacillales.f__Listeriaceae.g__Listeria.s__monocytogenes.t__FSL_J1_194
0.988
d__Bacteria.p__Firmicutes.c__Bacilli.o__Bacillales.f__Listeriaceae.g__Listeria.s__monocytogenes.t__FSL_J1_175
0.979
d__Bacteria.p__Firmicutes.c__Bacilli.o__Bacillales.f__Listeriaceae.g__Listeria.s__monocytogenes.t__FSL_R2_503
1.000
d__Bacteria.p__Firmicutes.c__Bacilli.o__Bacillales.f__Listeriaceae.g__Listeria.s__monocytogenes.t__FSL_J2_064
d__Bacteria.p__Firmicutes.c__Bacilli.o__Bacillales.f__Listeriaceae.g__Listeria.s__monocytogenes.t__FSL_J2_071
d__Bacteria.p__Firmicutes.c__Bacilli.o__Bacillales.f__Listeriaceae.g__Listeria.s__monocytogenes.t__Finland_1988
1.000
1.000
d__Bacteria.p__Firmicutes.c__Bacilli.o__Bacillales.f__Listeriaceae.g__Listeria.s__monocytogenes.t__J2818
d__Bacteria.p__Firmicutes.c__Bacilli.o__Bacillales.f__Listeriaceae.g__Listeria.s__monocytogenes.t__J0161
0.939
d__Bacteria.p__Firmicutes.c__Bacilli.o__Bacillales.f__Listeriaceae.g__Listeria.s__monocytogenes.t__str__1_2a_F6854
1.000
d__Bacteria.p__Firmicutes.c__Bacilli.o__Bacillales.f__Listeriaceae.g__Listeria.s__monocytogenes.t__FSL_R2_561
0.960
d__Bacteria.p__Firmicutes.c__Bacilli.o__Bacillales.f__Listeriaceae.g__Listeria.s__monocytogenes.t__EGD_e
0.989
d__Bacteria.p__Firmicutes.c__Bacilli.o__Bacillales.f__Listeriaceae.g__Listeria.s__monocytogenes.t__08_5923
0.992
d__Bacteria.p__Firmicutes.c__Bacilli.o__Bacillales.f__Listeriaceae.g__Listeria.s__monocytogenes.t__08_5578
d__Bacteria.p__Firmicutes.c__Bacilli.o__Bacillales.f__Listeriaceae.g__Listeria.s__innocua.t__Clip11262
0.833
d__Bacteria.p__Firmicutes.c__Bacilli.o__Bacillales.f__Listeriaceae.g__Listeria.s__seeligeri.t__serovar_1_2b_str__SLCC3954
1.000
d__Bacteria.p__Firmicutes.c__Bacilli.o__Bacillales.f__Listeriaceae.g__Listeria.s__welshimeri.t__serovar_6b_str__SLCC5334
d__Bacteria.p__Firmicutes.c__Bacilli.o__Bacillales.f__Listeriaceae.g__Listeria.s__monocytogenes.t__HCC23
0.999
d__Bacteria.p__Firmicutes.c__Bacilli.o__Bacillales.f__Listeriaceae.g__Listeria.s__monocytogenes.t__serotype_4a_str__L99
0.998
d__Bacteria.p__Firmicutes.c__Bacilli.o__Bacillales.f__Listeriaceae.g__Listeria.s__monocytogenes.t__M7
1.000
d__Bacteria.p__Firmicutes.c__Bacilli.o__Bacillales.f__Listeriaceae.g__Listeria.s__monocytogenes.t__FSL_N1_017
d__Bacteria.p__Firmicutes.c__Bacilli.o__Bacillales.f__Listeriaceae.g__Listeria.s__monocytogenes.t__HPB2262
0.927
d__Bacteria.p__Firmicutes.c__Bacilli.o__Bacillales.f__Listeriaceae.g__Listeria.s__monocytogenes.t__str__4b_F2365
0.786
d__Bacteria.p__Firmicutes.c__Bacilli.o__Bacillales.f__Listeriaceae.g__Listeria.s__monocytogenes.t__str__4b_H7858
d__Bacteria.p__Firmicutes.c__Bacilli.o__Bacillales.f__Listeriaceae.g__Listeria.s__monocytogenes.t__Clip81459
d__Bacteria.p__Firmicutes.c__Bacilli.o__Bacillales.f__Listeriaceae.g__Listeria.s__monocytogenes.t__FSL_N3_165
0.922
d__Bacteria.p__Firmicutes.c__Bacilli.o__Bacillales.f__Listeriaceae.g__Listeria.s__monocytogenes.t__10403S
1.000
d__Bacteria.p__Firmicutes.c__Bacilli.o__Bacillales.f__Listeriaceae.g__Listeria.s__monocytogenes.t__FSL_F2_515
d__Bacteria.p__Firmicutes.c__Bacilli.o__Bacillales.f__Listeriaceae.g__Listeria.s__monocytogenes.t__LO28
0.947
d__Bacteria.p__Firmicutes.c__Bacilli.o__Bacillales.f__Listeriaceae.g__Listeria.s__monocytogenes.t__FSL_J1_208
d__Bacteria.p__Firmicutes.c__Bacilli.o__Lactobacillales.f__Carnobacteriaceae.g__Carnobacterium.s__sp_.t__17_4
1.000
d__Bacteria.p__Firmicutes.c__Bacilli.o__Lactobacillales.f__Carnobacteriaceae.g__Carnobacterium.s__sp_.t__AT7
d__Bacteria.p__Firmicutes.c__Bacilli.o__Lactobacillales.f__Carnobacteriaceae.g__Granulicatella.s__elegans.t__ATCC_700633
0.872
1.000
d__Bacteria.p__Firmicutes.c__Bacilli.o__Lactobacillales.f__Carnobacteriaceae.g__Granulicatella.s__adiacens.t__ATCC_49175
1.000
d__Bacteria.p__Firmicutes.c__Bacilli.o__Lactobacillales.f__Aerococcaceae.g__Eremococcus.s__coleocola.t__ACS_139_V_Col8
1.000
d__Bacteria.p__Firmicutes.c__Bacilli.o__Lactobacillales.f__Aerococcaceae.g__Abiotrophia.s__defectiva.t__ATCC_51271
0.984
d__Bacteria.p__Firmicutes.c__Bacilli.o__Lactobacillales.f__Aerococcaceae.g__Aerococcus.s__viridans.t___1
1.000
d__Bacteria.p__Firmicutes.c__Bacilli.o__Lactobacillales.f__Aerococcaceae.g__Aerococcus.s__urinae.t__ACS_120_V_Col10a
d__Bacteria.p__Firmicutes.c__Bacilli.o__Lactobacillales.f__Streptococcaceae.g__Lactococcus.s__lactis.t__subsp__cremoris_SK11

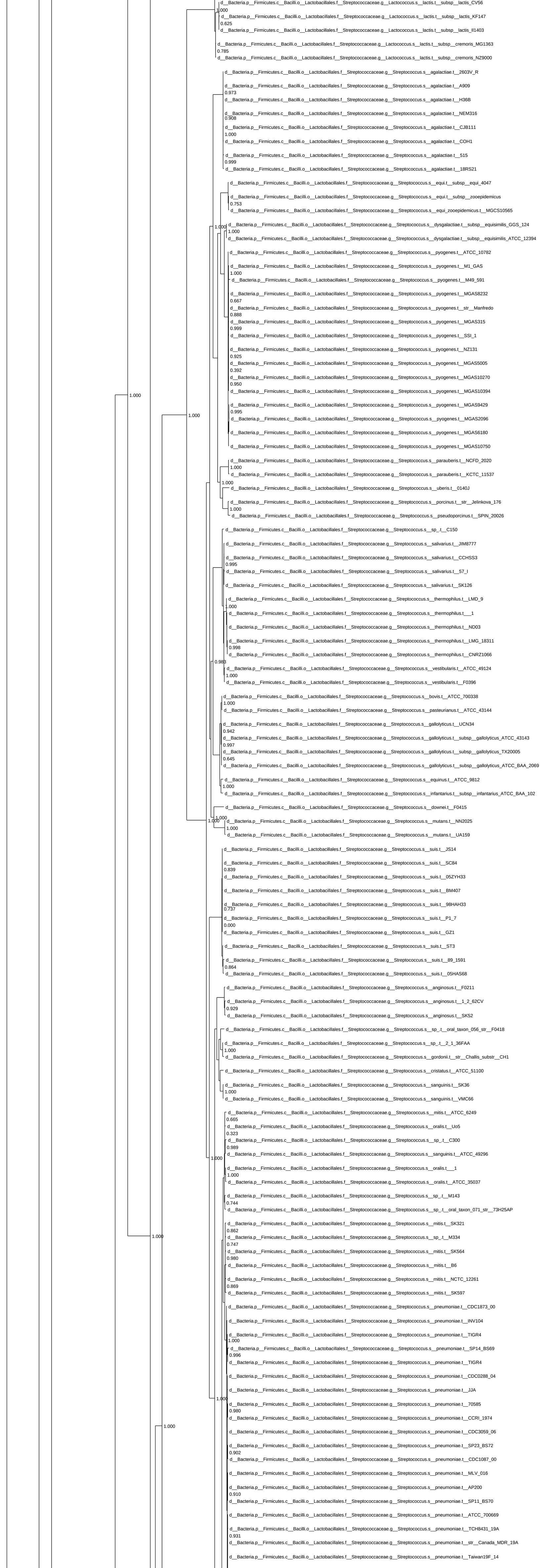

d__Bacteria.p__Firmicutes.c__Bacilli.o__Lactobacillales.f__Streptococcaceae.g__Lactococcus.s__lactis.t__subsp__lactis_CV56
1.000
d__Bacteria.p__Firmicutes.c__Bacilli.o__Lactobacillales.f__Streptococcaceae.g__Lactococcus.s__lactis.t__subsp__lactis_KF147
0.625
d__Bacteria.p__Firmicutes.c__Bacilli.o__Lactobacillales.f__Streptococcaceae.g__Lactococcus.s__lactis.t__subsp__lactis_Il1403
d__Bacteria.p__Firmicutes.c__Bacilli.o__Lactobacillales.f__Streptococcaceae.g__Lactococcus.s__lactis.t__subsp__cremoris_MG1363
0.785
d__Bacteria.p__Firmicutes.c__Bacilli.o__Lactobacillales.f__Streptococcaceae.g__Lactococcus.s__lactis.t__subsp__cremoris_NZ9000
d__Bacteria.p__Firmicutes.c__Bacilli.o__Lactobacillales.f__Streptococcaceae.g__Streptococcus.s__agalactiae.t__2603V_R
d__Bacteria.p__Firmicutes.c__Bacilli.o__Lactobacillales.f__Streptococcaceae.g__Streptococcus.s__agalactiae.t__A909
0.973
d__Bacteria.p__Firmicutes.c__Bacilli.o__Lactobacillales.f__Streptococcaceae.g__Streptococcus.s__agalactiae.t__H36B
d__Bacteria.p__Firmicutes.c__Bacilli.o__Lactobacillales.f__Streptococcaceae.g__Streptococcus.s__agalactiae.t__NEM316
0.908
d__Bacteria.p__Firmicutes.c__Bacilli.o__Lactobacillales.f__Streptococcaceae.g__Streptococcus.s__agalactiae.t__CJB111
1.000
d__Bacteria.p__Firmicutes.c__Bacilli.o__Lactobacillales.f__Streptococcaceae.g__Streptococcus.s__agalactiae.t__COH1
d__Bacteria.p__Firmicutes.c__Bacilli.o__Lactobacillales.f__Streptococcaceae.g__Streptococcus.s__agalactiae.t__515
0.999
d__Bacteria.p__Firmicutes.c__Bacilli.o__Lactobacillales.f__Streptococcaceae.g__Streptococcus.s__agalactiae.t__18RS21
d__Bacteria.p__Firmicutes.c__Bacilli.o__Lactobacillales.f__Streptococcaceae.g__Streptococcus.s__equi.t__subsp__equi_4047
d__Bacteria.p__Firmicutes.c__Bacilli.o__Lactobacillales.f__Streptococcaceae.g__Streptococcus.s__equi.t__subsp__zooepidemicus
0.753
d__Bacteria.p__Firmicutes.c__Bacilli.o__Lactobacillales.f__Streptococcaceae.g__Streptococcus.s__equi_zooepidemicus.t__MGCS10565
1.000
d__Bacteria.p__Firmicutes.c__Bacilli.o__Lactobacillales.f__Streptococcaceae.g__Streptococcus.s__dysgalactiae.t__subsp__equisimilis_GGS_124
1.000
d__Bacteria.p__Firmicutes.c__Bacilli.o__Lactobacillales.f__Streptococcaceae.g__Streptococcus.s__dysgalactiae.t__subsp__equisimilis_ATCC_12394
d__Bacteria.p__Firmicutes.c__Bacilli.o__Lactobacillales.f__Streptococcaceae.g__Streptococcus.s__pyogenes.t__ATCC_10782
d__Bacteria.p__Firmicutes.c__Bacilli.o__Lactobacillales.f__Streptococcaceae.g__Streptococcus.s__pyogenes.t__M1_GAS
1.000
d__Bacteria.p__Firmicutes.c__Bacilli.o__Lactobacillales.f__Streptococcaceae.g__Streptococcus.s__pyogenes.t__M49_591
d__Bacteria.p__Firmicutes.c__Bacilli.o__Lactobacillales.f__Streptococcaceae.g__Streptococcus.s__pyogenes.t__MGAS8232
0.667
d__Bacteria.p__Firmicutes.c__Bacilli.o__Lactobacillales.f__Streptococcaceae.g__Streptococcus.s__pyogenes.t__str__Manfredo
0.888
d__Bacteria.p__Firmicutes.c__Bacilli.o__Lactobacillales.f__Streptococcaceae.g__Streptococcus.s__pyogenes.t__MGAS315
0.999
d__Bacteria.p__Firmicutes.c__Bacilli.o__Lactobacillales.f__Streptococcaceae.g__Streptococcus.s__pyogenes.t__SSI_1
d__Bacteria.p__Firmicutes.c__Bacilli.o__Lactobacillales.f__Streptococcaceae.g__Streptococcus.s__pyogenes.t__NZ131
0.925
d__Bacteria.p__Firmicutes.c__Bacilli.o__Lactobacillales.f__Streptococcaceae.g__Streptococcus.s__pyogenes.t__MGAS5005
0.392
d__Bacteria.p__Firmicutes.c__Bacilli.o__Lactobacillales.f__Streptococcaceae.g__Streptococcus.s__pyogenes.t__MGAS10270
0.950
d__Bacteria.p__Firmicutes.c__Bacilli.o__Lactobacillales.f__Streptococcaceae.g__Streptococcus.s__pyogenes.t__MGAS10394
d__Bacteria.p__Firmicutes.c__Bacilli.o__Lactobacillales.f__Streptococcaceae.g__Streptococcus.s__pyogenes.t__MGAS9429
0.995
d__Bacteria.p__Firmicutes.c__Bacilli.o__Lactobacillales.f__Streptococcaceae.g__Streptococcus.s__pyogenes.t__MGAS2096
d__Bacteria.p__Firmicutes.c__Bacilli.o__Lactobacillales.f__Streptococcaceae.g__Streptococcus.s__pyogenes.t__MGAS6180
d__Bacteria.p__Firmicutes.c__Bacilli.o__Lactobacillales.f__Streptococcaceae.g__Streptococcus.s__pyogenes.t__MGAS10750
1.000
d__Bacteria.p__Firmicutes.c__Bacilli.o__Lactobacillales.f__Streptococcaceae.g__Streptococcus.s__parauberis.t__NCFD_2020
1.000
d__Bacteria.p__Firmicutes.c__Bacilli.o__Lactobacillales.f__Streptococcaceae.g__Streptococcus.s__parauberis.t__KCTC_11537
1.000
d__Bacteria.p__Firmicutes.c__Bacilli.o__Lactobacillales.f__Streptococcaceae.g__Streptococcus.s__uberis.t__0140J
d__Bacteria.p__Firmicutes.c__Bacilli.o__Lactobacillales.f__Streptococcaceae.g__Streptococcus.s__porcinus.t__str__Jelinkova_176
1.000
d__Bacteria.p__Firmicutes.c__Bacilli.o__Lactobacillales.f__Streptococcaceae.g__Streptococcus.s__pseudoporcinus.t__SPIN_20026
1.000
1.000
d__Bacteria.p__Firmicutes.c__Bacilli.o__Lactobacillales.f__Streptococcaceae.g__Streptococcus.s__sp_.t__C150
d__Bacteria.p__Firmicutes.c__Bacilli.o__Lactobacillales.f__Streptococcaceae.g__Streptococcus.s__salivarius.t__JIM8777
d__Bacteria.p__Firmicutes.c__Bacilli.o__Lactobacillales.f__Streptococcaceae.g__Streptococcus.s__salivarius.t__CCHSS3
0.995
d__Bacteria.p__Firmicutes.c__Bacilli.o__Lactobacillales.f__Streptococcaceae.g__Streptococcus.s__salivarius.t__57_I
d__Bacteria.p__Firmicutes.c__Bacilli.o__Lactobacillales.f__Streptococcaceae.g__Streptococcus.s__salivarius.t__SK126
d__Bacteria.p__Firmicutes.c__Bacilli.o__Lactobacillales.f__Streptococcaceae.g__Streptococcus.s__thermophilus.t__LMD_9
1.000
d__Bacteria.p__Firmicutes.c__Bacilli.o__Lactobacillales.f__Streptococcaceae.g__Streptococcus.s__thermophilus.t___1
d__Bacteria.p__Firmicutes.c__Bacilli.o__Lactobacillales.f__Streptococcaceae.g__Streptococcus.s__thermophilus.t__ND03
d__Bacteria.p__Firmicutes.c__Bacilli.o__Lactobacillales.f__Streptococcaceae.g__Streptococcus.s__thermophilus.t__LMG_18311
0.998
d__Bacteria.p__Firmicutes.c__Bacilli.o__Lactobacillales.f__Streptococcaceae.g__Streptococcus.s__thermophilus.t__CNRZ1066
0.983
d__Bacteria.p__Firmicutes.c__Bacilli.o__Lactobacillales.f__Streptococcaceae.g__Streptococcus.s__vestibularis.t__ATCC_49124
1.000
d__Bacteria.p__Firmicutes.c__Bacilli.o__Lactobacillales.f__Streptococcaceae.g__Streptococcus.s__vestibularis.t__F0396
d__Bacteria.p__Firmicutes.c__Bacilli.o__Lactobacillales.f__Streptococcaceae.g__Streptococcus.s__bovis.t__ATCC_700338
1.000
d__Bacteria.p__Firmicutes.c__Bacilli.o__Lactobacillales.f__Streptococcaceae.g__Streptococcus.s__pasteurianus.t__ATCC_43144
d__Bacteria.p__Firmicutes.c__Bacilli.o__Lactobacillales.f__Streptococcaceae.g__Streptococcus.s__gallolyticus.t__UCN34
0.942
d__Bacteria.p__Firmicutes.c__Bacilli.o__Lactobacillales.f__Streptococcaceae.g__Streptococcus.s__gallolyticus.t__subsp__gallolyticus_ATCC_43143
0.997
d__Bacteria.p__Firmicutes.c__Bacilli.o__Lactobacillales.f__Streptococcaceae.g__Streptococcus.s__gallolyticus.t__subsp__gallolyticus_TX20005
0.645
d__Bacteria.p__Firmicutes.c__Bacilli.o__Lactobacillales.f__Streptococcaceae.g__Streptococcus.s__gallolyticus.t__subsp__gallolyticus_ATCC_BAA_2069
d__Bacteria.p__Firmicutes.c__Bacilli.o__Lactobacillales.f__Streptococcaceae.g__Streptococcus.s__equinus.t__ATCC_9812
1.000
d__Bacteria.p__Firmicutes.c__Bacilli.o__Lactobacillales.f__Streptococcaceae.g__Streptococcus.s__infantarius.t__subsp__infantarius_ATCC_BAA_102
d__Bacteria.p__Firmicutes.c__Bacilli.o__Lactobacillales.f__Streptococcaceae.g__Streptococcus.s__downei.t__F0415
1.000
1.000
d__Bacteria.p__Firmicutes.c__Bacilli.o__Lactobacillales.f__Streptococcaceae.g__Streptococcus.s__mutans.t__NN2025
1.000
d__Bacteria.p__Firmicutes.c__Bacilli.o__Lactobacillales.f__Streptococcaceae.g__Streptococcus.s__mutans.t__UA159
d__Bacteria.p__Firmicutes.c__Bacilli.o__Lactobacillales.f__Streptococcaceae.g__Streptococcus.s__suis.t__JS14
d__Bacteria.p__Firmicutes.c__Bacilli.o__Lactobacillales.f__Streptococcaceae.g__Streptococcus.s__suis.t__SC84
0.839
d__Bacteria.p__Firmicutes.c__Bacilli.o__Lactobacillales.f__Streptococcaceae.g__Streptococcus.s__suis.t__05ZYH33
d__Bacteria.p__Firmicutes.c__Bacilli.o__Lactobacillales.f__Streptococcaceae.g__Streptococcus.s__suis.t__BM407
d__Bacteria.p__Firmicutes.c__Bacilli.o__Lactobacillales.f__Streptococcaceae.g__Streptococcus.s__suis.t__98HAH33
0.737
d__Bacteria.p__Firmicutes.c__Bacilli.o__Lactobacillales.f__Streptococcaceae.g__Streptococcus.s__suis.t__P1_7
0.000
d__Bacteria.p__Firmicutes.c__Bacilli.o__Lactobacillales.f__Streptococcaceae.g__Streptococcus.s__suis.t__GZ1
d__Bacteria.p__Firmicutes.c__Bacilli.o__Lactobacillales.f__Streptococcaceae.g__Streptococcus.s__suis.t__ST3
d__Bacteria.p__Firmicutes.c__Bacilli.o__Lactobacillales.f__Streptococcaceae.g__Streptococcus.s__suis.t__89_1591
0.864
d__Bacteria.p__Firmicutes.c__Bacilli.o__Lactobacillales.f__Streptococcaceae.g__Streptococcus.s__suis.t__05HAS68
d__Bacteria.p__Firmicutes.c__Bacilli.o__Lactobacillales.f__Streptococcaceae.g__Streptococcus.s__anginosus.t__F0211
d__Bacteria.p__Firmicutes.c__Bacilli.o__Lactobacillales.f__Streptococcaceae.g__Streptococcus.s__anginosus.t__1_2_62CV
0.929
d__Bacteria.p__Firmicutes.c__Bacilli.o__Lactobacillales.f__Streptococcaceae.g__Streptococcus.s__anginosus.t__SK52
d__Bacteria.p__Firmicutes.c__Bacilli.o__Lactobacillales.f__Streptococcaceae.g__Streptococcus.s__sp_.t__oral_taxon_056_str__F0418
d__Bacteria.p__Firmicutes.c__Bacilli.o__Lactobacillales.f__Streptococcaceae.g__Streptococcus.s__sp_.t__2_1_36FAA
1.000
d__Bacteria.p__Firmicutes.c__Bacilli.o__Lactobacillales.f__Streptococcaceae.g__Streptococcus.s__gordonii.t__str__Challis_substr__CH1
d__Bacteria.p__Firmicutes.c__Bacilli.o__Lactobacillales.f__Streptococcaceae.g__Streptococcus.s__cristatus.t__ATCC_51100
d__Bacteria.p__Firmicutes.c__Bacilli.o__Lactobacillales.f__Streptococcaceae.g__Streptococcus.s__sanguinis.t__SK36
1.000
d__Bacteria.p__Firmicutes.c__Bacilli.o__Lactobacillales.f__Streptococcaceae.g__Streptococcus.s__sanguinis.t__VMC66
d__Bacteria.p__Firmicutes.c__Bacilli.o__Lactobacillales.f__Streptococcaceae.g__Streptococcus.s__mitis.t__ATCC_6249
0.665
d__Bacteria.p__Firmicutes.c__Bacilli.o__Lactobacillales.f__Streptococcaceae.g__Streptococcus.s__oralis.t__Uo5
0.323
d__Bacteria.p__Firmicutes.c__Bacilli.o__Lactobacillales.f__Streptococcaceae.g__Streptococcus.s__sp_.t__C300
0.989
d__Bacteria.p__Firmicutes.c__Bacilli.o__Lactobacillales.f__Streptococcaceae.g__Streptococcus.s__sanguinis.t__ATCC_49296
1.000
d__Bacteria.p__Firmicutes.c__Bacilli.o__Lactobacillales.f__Streptococcaceae.g__Streptococcus.s__oralis.t___1
1.000
d__Bacteria.p__Firmicutes.c__Bacilli.o__Lactobacillales.f__Streptococcaceae.g__Streptococcus.s__oralis.t__ATCC_35037
d__Bacteria.p__Firmicutes.c__Bacilli.o__Lactobacillales.f__Streptococcaceae.g__Streptococcus.s__sp_.t__M143
0.744
d__Bacteria.p__Firmicutes.c__Bacilli.o__Lactobacillales.f__Streptococcaceae.g__Streptococcus.s__sp_.t__oral_taxon_071_str__73H25AP
d__Bacteria.p__Firmicutes.c__Bacilli.o__Lactobacillales.f__Streptococcaceae.g__Streptococcus.s__mitis.t__SK321
0.862
d__Bacteria.p__Firmicutes.c__Bacilli.o__Lactobacillales.f__Streptococcaceae.g__Streptococcus.s__sp_.t__M334
0.747
d__Bacteria.p__Firmicutes.c__Bacilli.o__Lactobacillales.f__Streptococcaceae.g__Streptococcus.s__mitis.t__SK564
0.980
d__Bacteria.p__Firmicutes.c__Bacilli.o__Lactobacillales.f__Streptococcaceae.g__Streptococcus.s__mitis.t__B6
d__Bacteria.p__Firmicutes.c__Bacilli.o__Lactobacillales.f__Streptococcaceae.g__Streptococcus.s__mitis.t__NCTC_12261
0.869
d__Bacteria.p__Firmicutes.c__Bacilli.o__Lactobacillales.f__Streptococcaceae.g__Streptococcus.s__mitis.t__SK597
d__Bacteria.p__Firmicutes.c__Bacilli.o__Lactobacillales.f__Streptococcaceae.g__Streptococcus.s__pneumoniae.t__CDC1873_00
d__Bacteria.p__Firmicutes.c__Bacilli.o__Lactobacillales.f__Streptococcaceae.g__Streptococcus.s__pneumoniae.t__INV104
d__Bacteria.p__Firmicutes.c__Bacilli.o__Lactobacillales.f__Streptococcaceae.g__Streptococcus.s__pneumoniae.t__TIGR4
1.000
d__Bacteria.p__Firmicutes.c__Bacilli.o__Lactobacillales.f__Streptococcaceae.g__Streptococcus.s__pneumoniae.t__SP14_BS69
0.996
d__Bacteria.p__Firmicutes.c__Bacilli.o__Lactobacillales.f__Streptococcaceae.g__Streptococcus.s__pneumoniae.t__TIGR4
d__Bacteria.p__Firmicutes.c__Bacilli.o__Lactobacillales.f__Streptococcaceae.g__Streptococcus.s__pneumoniae.t__CDC0288_04
d__Bacteria.p__Firmicutes.c__Bacilli.o__Lactobacillales.f__Streptococcaceae.g__Streptococcus.s__pneumoniae.t__JJA
1.000
d__Bacteria.p__Firmicutes.c__Bacilli.o__Lactobacillales.f__Streptococcaceae.g__Streptococcus.s__pneumoniae.t__70585
0.980
d__Bacteria.p__Firmicutes.c__Bacilli.o__Lactobacillales.f__Streptococcaceae.g__Streptococcus.s__pneumoniae.t__CCRI_1974
d__Bacteria.p__Firmicutes.c__Bacilli.o__Lactobacillales.f__Streptococcaceae.g__Streptococcus.s__pneumoniae.t__CDC3059_06
d__Bacteria.p__Firmicutes.c__Bacilli.o__Lactobacillales.f__Streptococcaceae.g__Streptococcus.s__pneumoniae.t__SP23_BS72
0.902
d__Bacteria.p__Firmicutes.c__Bacilli.o__Lactobacillales.f__Streptococcaceae.g__Streptococcus.s__pneumoniae.t__CDC1087_00
d__Bacteria.p__Firmicutes.c__Bacilli.o__Lactobacillales.f__Streptococcaceae.g__Streptococcus.s__pneumoniae.t__MLV_016
d__Bacteria.p__Firmicutes.c__Bacilli.o__Lactobacillales.f__Streptococcaceae.g__Streptococcus.s__pneumoniae.t__AP200
0.910
d__Bacteria.p__Firmicutes.c__Bacilli.o__Lactobacillales.f__Streptococcaceae.g__Streptococcus.s__pneumoniae.t__SP11_BS70
d__Bacteria.p__Firmicutes.c__Bacilli.o__Lactobacillales.f__Streptococcaceae.g__Streptococcus.s__pneumoniae.t__ATCC_700669
d__Bacteria.p__Firmicutes.c__Bacilli.o__Lactobacillales.f__Streptococcaceae.g__Streptococcus.s__pneumoniae.t__TCH8431_19A
0.931
d__Bacteria.p__Firmicutes.c__Bacilli.o__Lactobacillales.f__Streptococcaceae.g__Streptococcus.s__pneumoniae.t__str__Canada_MDR_19A
d__Bacteria.p__Firmicutes.c__Bacilli.o__Lactobacillales.f__Streptococcaceae.g__Streptococcus.s__pneumoniae.t__Taiwan19F_14
1.000
1.000
1.000
1.000

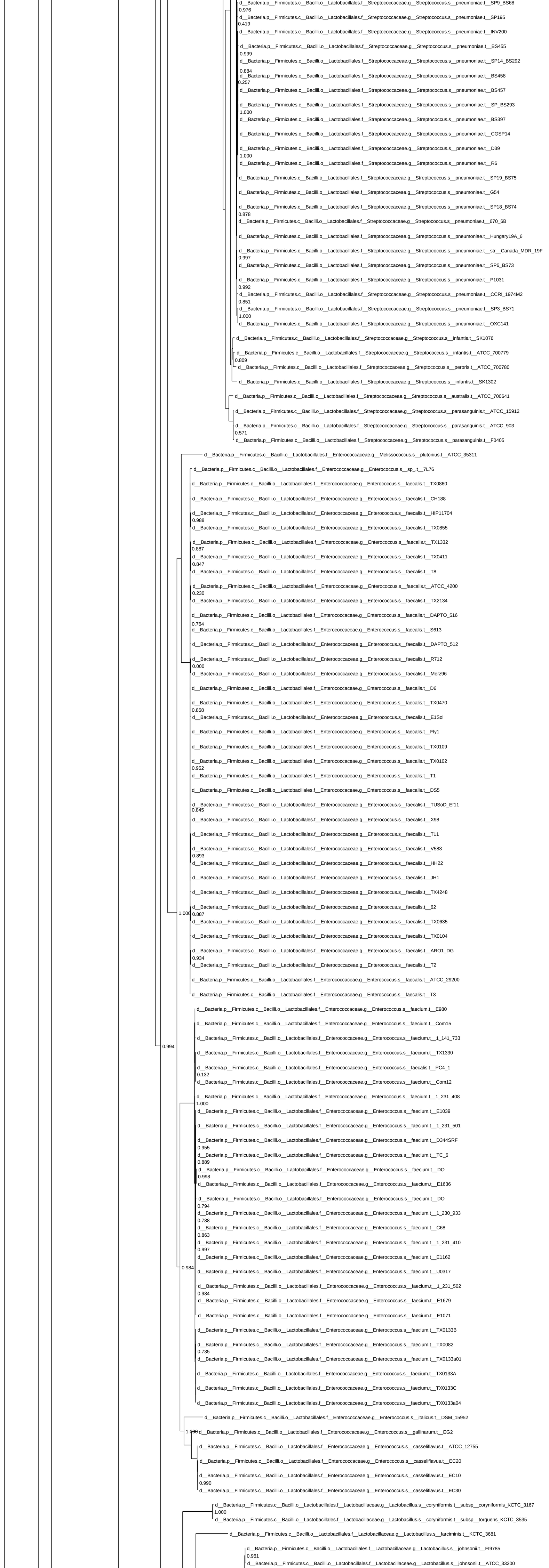

d__Bacteria.p__Firmicutes.c__Bacilli.o__Lactobacillales.f__Streptococcaceae.g__Streptococcus.s__pneumoniae.t__SP9_BS68
0.976
d__Bacteria.p__Firmicutes.c__Bacilli.o__Lactobacillales.f__Streptococcaceae.g__Streptococcus.s__pneumoniae.t__SP195
0.419
d__Bacteria.p__Firmicutes.c__Bacilli.o__Lactobacillales.f__Streptococcaceae.g__Streptococcus.s__pneumoniae.t__INV200
d__Bacteria.p__Firmicutes.c__Bacilli.o__Lactobacillales.f__Streptococcaceae.g__Streptococcus.s__pneumoniae.t__BS455
0.999
d__Bacteria.p__Firmicutes.c__Bacilli.o__Lactobacillales.f__Streptococcaceae.g__Streptococcus.s__pneumoniae.t__SP14_BS292
0.884
d__Bacteria.p__Firmicutes.c__Bacilli.o__Lactobacillales.f__Streptococcaceae.g__Streptococcus.s__pneumoniae.t__BS458
0.257
d__Bacteria.p__Firmicutes.c__Bacilli.o__Lactobacillales.f__Streptococcaceae.g__Streptococcus.s__pneumoniae.t__BS457
d__Bacteria.p__Firmicutes.c__Bacilli.o__Lactobacillales.f__Streptococcaceae.g__Streptococcus.s__pneumoniae.t__SP_BS293
1.000
d__Bacteria.p__Firmicutes.c__Bacilli.o__Lactobacillales.f__Streptococcaceae.g__Streptococcus.s__pneumoniae.t__BS397
d__Bacteria.p__Firmicutes.c__Bacilli.o__Lactobacillales.f__Streptococcaceae.g__Streptococcus.s__pneumoniae.t__CGSP14
d__Bacteria.p__Firmicutes.c__Bacilli.o__Lactobacillales.f__Streptococcaceae.g__Streptococcus.s__pneumoniae.t__D39
1.000
d__Bacteria.p__Firmicutes.c__Bacilli.o__Lactobacillales.f__Streptococcaceae.g__Streptococcus.s__pneumoniae.t__R6
d__Bacteria.p__Firmicutes.c__Bacilli.o__Lactobacillales.f__Streptococcaceae.g__Streptococcus.s__pneumoniae.t__SP19_BS75
d__Bacteria.p__Firmicutes.c__Bacilli.o__Lactobacillales.f__Streptococcaceae.g__Streptococcus.s__pneumoniae.t__G54
d__Bacteria.p__Firmicutes.c__Bacilli.o__Lactobacillales.f__Streptococcaceae.g__Streptococcus.s__pneumoniae.t__SP18_BS74
0.878
d__Bacteria.p__Firmicutes.c__Bacilli.o__Lactobacillales.f__Streptococcaceae.g__Streptococcus.s__pneumoniae.t__670_6B
d__Bacteria.p__Firmicutes.c__Bacilli.o__Lactobacillales.f__Streptococcaceae.g__Streptococcus.s__pneumoniae.t__Hungary19A_6
d__Bacteria.p__Firmicutes.c__Bacilli.o__Lactobacillales.f__Streptococcaceae.g__Streptococcus.s__pneumoniae.t__str__Canada_MDR_19F
0.997
d__Bacteria.p__Firmicutes.c__Bacilli.o__Lactobacillales.f__Streptococcaceae.g__Streptococcus.s__pneumoniae.t__SP6_BS73
d__Bacteria.p__Firmicutes.c__Bacilli.o__Lactobacillales.f__Streptococcaceae.g__Streptococcus.s__pneumoniae.t__P1031
0.992
d__Bacteria.p__Firmicutes.c__Bacilli.o__Lactobacillales.f__Streptococcaceae.g__Streptococcus.s__pneumoniae.t__CCRI_1974M2
0.851
d__Bacteria.p__Firmicutes.c__Bacilli.o__Lactobacillales.f__Streptococcaceae.g__Streptococcus.s__pneumoniae.t__SP3_BS71
1.000
d__Bacteria.p__Firmicutes.c__Bacilli.o__Lactobacillales.f__Streptococcaceae.g__Streptococcus.s__pneumoniae.t__OXC141
d__Bacteria.p__Firmicutes.c__Bacilli.o__Lactobacillales.f__Streptococcaceae.g__Streptococcus.s__infantis.t__SK1076
d__Bacteria.p__Firmicutes.c__Bacilli.o__Lactobacillales.f__Streptococcaceae.g__Streptococcus.s__infantis.t__ATCC_700779
0.809
d__Bacteria.p__Firmicutes.c__Bacilli.o__Lactobacillales.f__Streptococcaceae.g__Streptococcus.s__peroris.t__ATCC_700780
d__Bacteria.p__Firmicutes.c__Bacilli.o__Lactobacillales.f__Streptococcaceae.g__Streptococcus.s__infantis.t__SK1302
d__Bacteria.p__Firmicutes.c__Bacilli.o__Lactobacillales.f__Streptococcaceae.g__Streptococcus.s__australis.t__ATCC_700641
d__Bacteria.p__Firmicutes.c__Bacilli.o__Lactobacillales.f__Streptococcaceae.g__Streptococcus.s__parasanguinis.t__ATCC_15912
d__Bacteria.p__Firmicutes.c__Bacilli.o__Lactobacillales.f__Streptococcaceae.g__Streptococcus.s__parasanguinis.t__ATCC_903
0.571
d__Bacteria.p__Firmicutes.c__Bacilli.o__Lactobacillales.f__Streptococcaceae.g__Streptococcus.s__parasanguinis.t__F0405
d__Bacteria.p__Firmicutes.c__Bacilli.o__Lactobacillales.f__Enterococcaceae.g__Melissococcus.s__plutonius.t__ATCC_35311
d__Bacteria.p__Firmicutes.c__Bacilli.o__Lactobacillales.f__Enterococcaceae.g__Enterococcus.s__sp_t__7L76
d__Bacteria.p__Firmicutes.c__Bacilli.o__Lactobacillales.f__Enterococcaceae.g__Enterococcus.s__faecalis.t__TX0860
d__Bacteria.p__Firmicutes.c__Bacilli.o__Lactobacillales.f__Enterococcaceae.g__Enterococcus.s__faecalis.t__CH188
d__Bacteria.p__Firmicutes.c__Bacilli.o__Lactobacillales.f__Enterococcaceae.g__Enterococcus.s__faecalis.t__HIP11704
0.988
d__Bacteria.p__Firmicutes.c__Bacilli.o__Lactobacillales.f__Enterococcaceae.g__Enterococcus.s__faecalis.t__TX0855
d__Bacteria.p__Firmicutes.c__Bacilli.o__Lactobacillales.f__Enterococcaceae.g__Enterococcus.s__faecalis.t__TX1332
0.887
d__Bacteria.p__Firmicutes.c__Bacilli.o__Lactobacillales.f__Enterococcaceae.g__Enterococcus.s__faecalis.t__TX0411
0.847
d__Bacteria.p__Firmicutes.c__Bacilli.o__Lactobacillales.f__Enterococcaceae.g__Enterococcus.s__faecalis.t__T8
d__Bacteria.p__Firmicutes.c__Bacilli.o__Lactobacillales.f__Enterococcaceae.g__Enterococcus.s__faecalis.t__ATCC_4200
0.230
d__Bacteria.p__Firmicutes.c__Bacilli.o__Lactobacillales.f__Enterococcaceae.g__Enterococcus.s__faecalis.t__TX2134
d__Bacteria.p__Firmicutes.c__Bacilli.o__Lactobacillales.f__Enterococcaceae.g__Enterococcus.s__faecalis.t__DAPTO_516
0.764
d__Bacteria.p__Firmicutes.c__Bacilli.o__Lactobacillales.f__Enterococcaceae.g__Enterococcus.s__faecalis.t__S613
d__Bacteria.p__Firmicutes.c__Bacilli.o__Lactobacillales.f__Enterococcaceae.g__Enterococcus.s__faecalis.t__DAPTO_512
d__Bacteria.p__Firmicutes.c__Bacilli.o__Lactobacillales.f__Enterococcaceae.g__Enterococcus.s__faecalis.t__R712
0.000
d__Bacteria.p__Firmicutes.c__Bacilli.o__Lactobacillales.f__Enterococcaceae.g__Enterococcus.s__faecalis.t__Merz96
d__Bacteria.p__Firmicutes.c__Bacilli.o__Lactobacillales.f__Enterococcaceae.g__Enterococcus.s__faecalis.t__D6
d__Bacteria.p__Firmicutes.c__Bacilli.o__Lactobacillales.f__Enterococcaceae.g__Enterococcus.s__faecalis.t__TX0470
0.858
d__Bacteria.p__Firmicutes.c__Bacilli.o__Lactobacillales.f__Enterococcaceae.g__Enterococcus.s__faecalis.t__E1Sol
d__Bacteria.p__Firmicutes.c__Bacilli.o__Lactobacillales.f__Enterococcaceae.g__Enterococcus.s__faecalis.t__Fly1
d__Bacteria.p__Firmicutes.c__Bacilli.o__Lactobacillales.f__Enterococcaceae.g__Enterococcus.s__faecalis.t__TX0109
d__Bacteria.p__Firmicutes.c__Bacilli.o__Lactobacillales.f__Enterococcaceae.g__Enterococcus.s__faecalis.t__TX0102
0.952
d__Bacteria.p__Firmicutes.c__Bacilli.o__Lactobacillales.f__Enterococcaceae.g__Enterococcus.s__faecalis.t__T1
d__Bacteria.p__Firmicutes.c__Bacilli.o__Lactobacillales.f__Enterococcaceae.g__Enterococcus.s__faecalis.t__DS5
d__Bacteria.p__Firmicutes.c__Bacilli.o__Lactobacillales.f__Enterococcaceae.g__Enterococcus.s__faecalis.t__TUSoD_Ef11
0.845
d__Bacteria.p__Firmicutes.c__Bacilli.o__Lactobacillales.f__Enterococcaceae.g__Enterococcus.s__faecalis.t__X98
d__Bacteria.p__Firmicutes.c__Bacilli.o__Lactobacillales.f__Enterococcaceae.g__Enterococcus.s__faecalis.t__T11
d__Bacteria.p__Firmicutes.c__Bacilli.o__Lactobacillales.f__Enterococcaceae.g__Enterococcus.s__faecalis.t__V583
0.893
d__Bacteria.p__Firmicutes.c__Bacilli.o__Lactobacillales.f__Enterococcaceae.g__Enterococcus.s__faecalis.t__HH22
d__Bacteria.p__Firmicutes.c__Bacilli.o__Lactobacillales.f__Enterococcaceae.g__Enterococcus.s__faecalis.t__JH1
d__Bacteria.p__Firmicutes.c__Bacilli.o__Lactobacillales.f__Enterococcaceae.g__Enterococcus.s__faecalis.t__TX4248
d__Bacteria.p__Firmicutes.c__Bacilli.o__Lactobacillales.f__Enterococcaceae.g__Enterococcus.s__faecalis.t__62
1.000
0.887
d__Bacteria.p__Firmicutes.c__Bacilli.o__Lactobacillales.f__Enterococcaceae.g__Enterococcus.s__faecalis.t__TX0635
d__Bacteria.p__Firmicutes.c__Bacilli.o__Lactobacillales.f__Enterococcaceae.g__Enterococcus.s__faecalis.t__TX0104
d__Bacteria.p__Firmicutes.c__Bacilli.o__Lactobacillales.f__Enterococcaceae.g__Enterococcus.s__faecalis.t__ARO1_DG
0.934
d__Bacteria.p__Firmicutes.c__Bacilli.o__Lactobacillales.f__Enterococcaceae.g__Enterococcus.s__faecalis.t__T2
d__Bacteria.p__Firmicutes.c__Bacilli.o__Lactobacillales.f__Enterococcaceae.g__Enterococcus.s__faecalis.t__ATCC_29200
d__Bacteria.p__Firmicutes.c__Bacilli.o__Lactobacillales.f__Enterococcaceae.g__Enterococcus.s__faecalis.t__T3
d__Bacteria.p__Firmicutes.c__Bacilli.o__Lactobacillales.f__Enterococcaceae.g__Enterococcus.s__faecium.t__E980
d__Bacteria.p__Firmicutes.c__Bacilli.o__Lactobacillales.f__Enterococcaceae.g__Enterococcus.s__faecium.t__Com15
d__Bacteria.p__Firmicutes.c__Bacilli.o__Lactobacillales.f__Enterococcaceae.g__Enterococcus.s__faecium.t__1_141_733
0.994
d__Bacteria.p__Firmicutes.c__Bacilli.o__Lactobacillales.f__Enterococcaceae.g__Enterococcus.s__faecium.t__TX1330
d__Bacteria.p__Firmicutes.c__Bacilli.o__Lactobacillales.f__Enterococcaceae.g__Enterococcus.s__faecalis.t__PC4_1
0.132
d__Bacteria.p__Firmicutes.c__Bacilli.o__Lactobacillales.f__Enterococcaceae.g__Enterococcus.s__faecium.t__Com12
d__Bacteria.p__Firmicutes.c__Bacilli.o__Lactobacillales.f__Enterococcaceae.g__Enterococcus.s__faecium.t__1_231_408
1.000
d__Bacteria.p__Firmicutes.c__Bacilli.o__Lactobacillales.f__Enterococcaceae.g__Enterococcus.s__faecium.t__E1039
d__Bacteria.p__Firmicutes.c__Bacilli.o__Lactobacillales.f__Enterococcaceae.g__Enterococcus.s__faecium.t__1_231_501
d__Bacteria.p__Firmicutes.c__Bacilli.o__Lactobacillales.f__Enterococcaceae.g__Enterococcus.s__faecium.t__D344SRF
0.955
d__Bacteria.p__Firmicutes.c__Bacilli.o__Lactobacillales.f__Enterococcaceae.g__Enterococcus.s__faecium.t__TC_6
0.889
d__Bacteria.p__Firmicutes.c__Bacilli.o__Lactobacillales.f__Enterococcaceae.g__Enterococcus.s__faecium.t__DO
0.998
d__Bacteria.p__Firmicutes.c__Bacilli.o__Lactobacillales.f__Enterococcaceae.g__Enterococcus.s__faecium.t__E1636
d__Bacteria.p__Firmicutes.c__Bacilli.o__Lactobacillales.f__Enterococcaceae.g__Enterococcus.s__faecium.t__DO
0.794
d__Bacteria.p__Firmicutes.c__Bacilli.o__Lactobacillales.f__Enterococcaceae.g__Enterococcus.s__faecium.t__1_230_933
0.788
d__Bacteria.p__Firmicutes.c__Bacilli.o__Lactobacillales.f__Enterococcaceae.g__Enterococcus.s__faecium.t__C68
0.863
d__Bacteria.p__Firmicutes.c__Bacilli.o__Lactobacillales.f__Enterococcaceae.g__Enterococcus.s__faecium.t__1_231_410
0.997
d__Bacteria.p__Firmicutes.c__Bacilli.o__Lactobacillales.f__Enterococcaceae.g__Enterococcus.s__faecium.t__E1162
d__Bacteria.p__Firmicutes.c__Bacilli.o__Lactobacillales.f__Enterococcaceae.g__Enterococcus.s__faecium.t__U0317
0.984
d__Bacteria.p__Firmicutes.c__Bacilli.o__Lactobacillales.f__Enterococcaceae.g__Enterococcus.s__faecium.t__1_231_502
0.984
d__Bacteria.p__Firmicutes.c__Bacilli.o__Lactobacillales.f__Enterococcaceae.g__Enterococcus.s__faecium.t__E1679
d__Bacteria.p__Firmicutes.c__Bacilli.o__Lactobacillales.f__Enterococcaceae.g__Enterococcus.s__faecium.t__E1071
d__Bacteria.p__Firmicutes.c__Bacilli.o__Lactobacillales.f__Enterococcaceae.g__Enterococcus.s__faecium.t__TX0133B
d__Bacteria.p__Firmicutes.c__Bacilli.o__Lactobacillales.f__Enterococcaceae.g__Enterococcus.s__faecium.t__TX0082
0.735
d__Bacteria.p__Firmicutes.c__Bacilli.o__Lactobacillales.f__Enterococcaceae.g__Enterococcus.s__faecium.t__TX0133a01
d__Bacteria.p__Firmicutes.c__Bacilli.o__Lactobacillales.f__Enterococcaceae.g__Enterococcus.s__faecium.t__TX0133A
d__Bacteria.p__Firmicutes.c__Bacilli.o__Lactobacillales.f__Enterococcaceae.g__Enterococcus.s__faecium.t__TX0133C
d__Bacteria.p__Firmicutes.c__Bacilli.o__Lactobacillales.f__Enterococcaceae.g__Enterococcus.s__faecium.t__TX0133a04
d__Bacteria.p__Firmicutes.c__Bacilli.o__Lactobacillales.f__Enterococcaceae.g__Enterococcus.s__italicus.t__DSM_15952
1.000
d__Bacteria.p__Firmicutes.c__Bacilli.o__Lactobacillales.f__Enterococcaceae.g__Enterococcus.s__gallinarum.t__EG2
d__Bacteria.p__Firmicutes.c__Bacilli.o__Lactobacillales.f__Enterococcaceae.g__Enterococcus.s__casseliflavus.t__ATCC_12755
d__Bacteria.p__Firmicutes.c__Bacilli.o__Lactobacillales.f__Enterococcaceae.g__Enterococcus.s__casseliflavus.t__EC20
d__Bacteria.p__Firmicutes.c__Bacilli.o__Lactobacillales.f__Enterococcaceae.g__Enterococcus.s__casseliflavus.t__EC10
0.990
d__Bacteria.p__Firmicutes.c__Bacilli.o__Lactobacillales.f__Enterococcaceae.g__Enterococcus.s__casseliflavus.t__EC30
d__Bacteria.p__Firmicutes.c__Bacilli.o__Lactobacillales.f__Lactobacillaceae.g__Lactobacillus.s__coryniformis.t__subsp__coryniformis_KCTC_3167
1.000
d__Bacteria.p__Firmicutes.c__Bacilli.o__Lactobacillales.f__Lactobacillaceae.g__Lactobacillus.s__coryniformis.t__subsp__torquens_KCTC_3535
d__Bacteria.p__Firmicutes.c__Bacilli.o__Lactobacillales.f__Lactobacillaceae.g__Lactobacillus.s__farciminis.t__KCTC_3681
d__Bacteria.p__Firmicutes.c__Bacilli.o__Lactobacillales.f__Lactobacillaceae.g__Lactobacillus.s__johnsonii.t__FI9785
0.961
d__Bacteria.p__Firmicutes.c__Bacilli.o__Lactobacillales.f__Lactobacillaceae.g__Lactobacillus.s__johnsonii.t__ATCC_33200

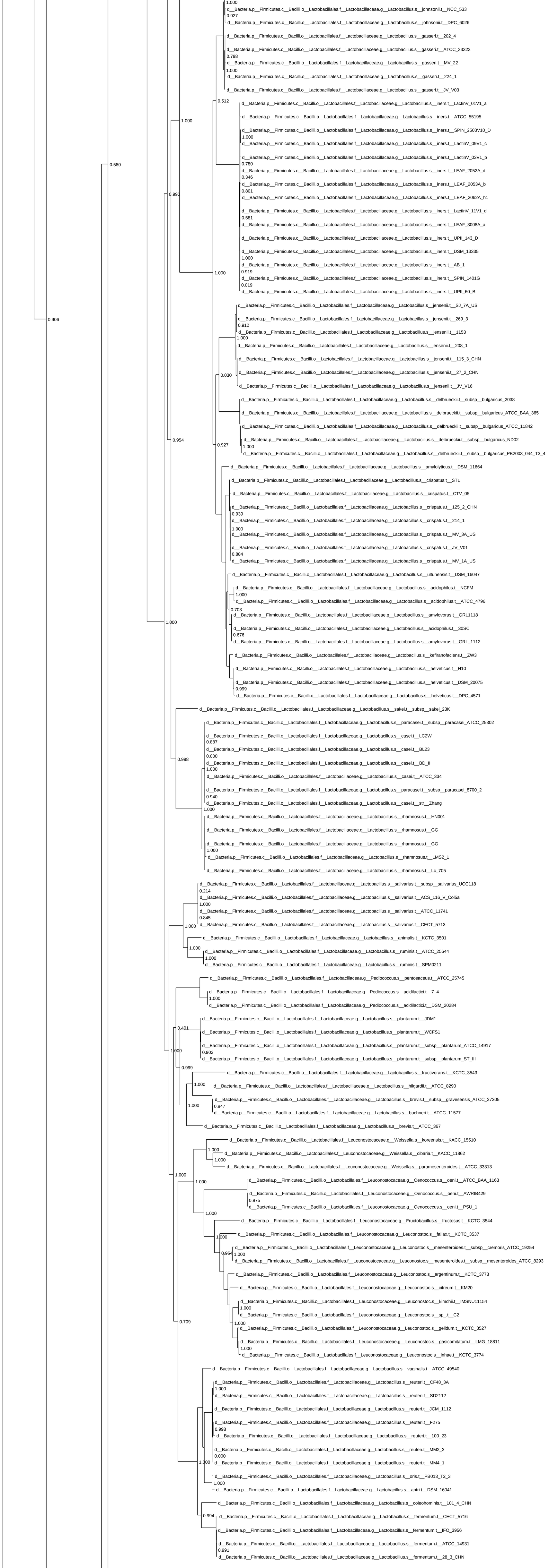

1.000
d__Bacteria.p__Firmicutes.c__Bacilli.o__Lactobacillales.f__Lactobacillaceae.g__Lactobacillus.s__johnsonii.t__NCC_533
0.927
d__Bacteria.p__Firmicutes.c__Bacilli.o__Lactobacillales.f__Lactobacillaceae.g__Lactobacillus.s__johnsonii.t__DPC_6026
d__Bacteria.p__Firmicutes.c__Bacilli.o__Lactobacillales.f__Lactobacillaceae.g__Lactobacillus.s__gasseri.t__202_4
d__Bacteria.p__Firmicutes.c__Bacilli.o__Lactobacillales.f__Lactobacillaceae.g__Lactobacillus.s__gasseri.t__ATCC_33323
0.798
d__Bacteria.p__Firmicutes.c__Bacilli.o__Lactobacillales.f__Lactobacillaceae.g__Lactobacillus.s__gasseri.t__MV_22
1.000
d__Bacteria.p__Firmicutes.c__Bacilli.o__Lactobacillales.f__Lactobacillaceae.g__Lactobacillus.s__gasseri.t__224_1
d__Bacteria.p__Firmicutes.c__Bacilli.o__Lactobacillales.f__Lactobacillaceae.g__Lactobacillus.s__gasseri.t__JV_V03
0.512
d__Bacteria.p__Firmicutes.c__Bacilli.o__Lactobacillales.f__Lactobacillaceae.g__Lactobacillus.s__iners.t__LactinV_01V1_a
1.000
d__Bacteria.p__Firmicutes.c__Bacilli.o__Lactobacillales.f__Lactobacillaceae.g__Lactobacillus.s__iners.t__ATCC_55195
d__Bacteria.p__Firmicutes.c__Bacilli.o__Lactobacillales.f__Lactobacillaceae.g__Lactobacillus.s__iners.t__SPIN_2503V10_D
1.000
d__Bacteria.p__Firmicutes.c__Bacilli.o__Lactobacillales.f__Lactobacillaceae.g__Lactobacillus.s__iners.t__LactinV_09V1_c
d__Bacteria.p__Firmicutes.c__Bacilli.o__Lactobacillales.f__Lactobacillaceae.g__Lactobacillus.s__iners.t__LactinV_03V1_b
0.580
0.780
d__Bacteria.p__Firmicutes.c__Bacilli.o__Lactobacillales.f__Lactobacillaceae.g__Lactobacillus.s__iners.t__LEAF_2052A_d
0.346
d__Bacteria.p__Firmicutes.c__Bacilli.o__Lactobacillales.f__Lactobacillaceae.g__Lactobacillus.s__iners.t__LEAF_2053A_b
0.801
0.990
d__Bacteria.p__Firmicutes.c__Bacilli.o__Lactobacillales.f__Lactobacillaceae.g__Lactobacillus.s__iners.t__LEAF_2062A_h1
d__Bacteria.p__Firmicutes.c__Bacilli.o__Lactobacillales.f__Lactobacillaceae.g__Lactobacillus.s__iners.t__LactinV_11V1_d
0.581
d__Bacteria.p__Firmicutes.c__Bacilli.o__Lactobacillales.f__Lactobacillaceae.g__Lactobacillus.s__iners.t__LEAF_3008A_a
d__Bacteria.p__Firmicutes.c__Bacilli.o__Lactobacillales.f__Lactobacillaceae.g__Lactobacillus.s__iners.t__UPII_143_D
d__Bacteria.p__Firmicutes.c__Bacilli.o__Lactobacillales.f__Lactobacillaceae.g__Lactobacillus.s__iners.t__DSM_13335
1.000
d__Bacteria.p__Firmicutes.c__Bacilli.o__Lactobacillales.f__Lactobacillaceae.g__Lactobacillus.s__iners.t__AB_1
1.000
0.919
d__Bacteria.p__Firmicutes.c__Bacilli.o__Lactobacillales.f__Lactobacillaceae.g__Lactobacillus.s__iners.t__SPIN_1401G
0.019
d__Bacteria.p__Firmicutes.c__Bacilli.o__Lactobacillales.f__Lactobacillaceae.g__Lactobacillus.s__iners.t__UPII_60_B
d__Bacteria.p__Firmicutes.c__Bacilli.o__Lactobacillales.f__Lactobacillaceae.g__Lactobacillus.s__jensenii.t__SJ_7A_US
0.906
d__Bacteria.p__Firmicutes.c__Bacilli.o__Lactobacillales.f__Lactobacillaceae.g__Lactobacillus.s__jensenii.t__269_3
0.912
d__Bacteria.p__Firmicutes.c__Bacilli.o__Lactobacillales.f__Lactobacillaceae.g__Lactobacillus.s__jensenii.t__1153
1.000
d__Bacteria.p__Firmicutes.c__Bacilli.o__Lactobacillales.f__Lactobacillaceae.g__Lactobacillus.s__jensenii.t__208_1
d__Bacteria.p__Firmicutes.c__Bacilli.o__Lactobacillales.f__Lactobacillaceae.g__Lactobacillus.s__jensenii.t__115_3_CHN
d__Bacteria.p__Firmicutes.c__Bacilli.o__Lactobacillales.f__Lactobacillaceae.g__Lactobacillus.s__jensenii.t__27_2_CHN
0.030
d__Bacteria.p__Firmicutes.c__Bacilli.o__Lactobacillales.f__Lactobacillaceae.g__Lactobacillus.s__jensenii.t__JV_V16
d__Bacteria.p__Firmicutes.c__Bacilli.o__Lactobacillales.f__Lactobacillaceae.g__Lactobacillus.s__delbrueckii.t__subsp__bulgaricus_2038
d__Bacteria.p__Firmicutes.c__Bacilli.o__Lactobacillales.f__Lactobacillaceae.g__Lactobacillus.s__delbrueckii.t__subsp__bulgaricus_ATCC_BAA_365
d__Bacteria.p__Firmicutes.c__Bacilli.o__Lactobacillales.f__Lactobacillaceae.g__Lactobacillus.s__delbrueckii.t__subsp__bulgaricus_ATCC_11842
0.954
d__Bacteria.p__Firmicutes.c__Bacilli.o__Lactobacillales.f__Lactobacillaceae.g__Lactobacillus.s__delbrueckii.t__subsp__bulgaricus_ND02
0.927
1.000
d__Bacteria.p__Firmicutes.c__Bacilli.o__Lactobacillales.f__Lactobacillaceae.g__Lactobacillus.s__delbrueckii.t__subsp__bulgaricus_PB2003_044_T3_4
d__Bacteria.p__Firmicutes.c__Bacilli.o__Lactobacillales.f__Lactobacillaceae.g__Lactobacillus.s__amylolyticus.t__DSM_11664
d__Bacteria.p__Firmicutes.c__Bacilli.o__Lactobacillales.f__Lactobacillaceae.g__Lactobacillus.s__crispatus.t__ST1
d__Bacteria.p__Firmicutes.c__Bacilli.o__Lactobacillales.f__Lactobacillaceae.g__Lactobacillus.s__crispatus.t__CTV_05
d__Bacteria.p__Firmicutes.c__Bacilli.o__Lactobacillales.f__Lactobacillaceae.g__Lactobacillus.s__crispatus.t__125_2_CHN
0.939
d__Bacteria.p__Firmicutes.c__Bacilli.o__Lactobacillales.f__Lactobacillaceae.g__Lactobacillus.s__crispatus.t__214_1
1.000
d__Bacteria.p__Firmicutes.c__Bacilli.o__Lactobacillales.f__Lactobacillaceae.g__Lactobacillus.s__crispatus.t__MV_3A_US
d__Bacteria.p__Firmicutes.c__Bacilli.o__Lactobacillales.f__Lactobacillaceae.g__Lactobacillus.s__crispatus.t__JV_V01
0.884
d__Bacteria.p__Firmicutes.c__Bacilli.o__Lactobacillales.f__Lactobacillaceae.g__Lactobacillus.s__crispatus.t__MV_1A_US
d__Bacteria.p__Firmicutes.c__Bacilli.o__Lactobacillales.f__Lactobacillaceae.g__Lactobacillus.s__ultunensis.t__DSM_16047
d__Bacteria.p__Firmicutes.c__Bacilli.o__Lactobacillales.f__Lactobacillaceae.g__Lactobacillus.s__acidophilus.t__NCFM
1.000
d__Bacteria.p__Firmicutes.c__Bacilli.o__Lactobacillales.f__Lactobacillaceae.g__Lactobacillus.s__acidophilus.t__ATCC_4796
0.703
d__Bacteria.p__Firmicutes.c__Bacilli.o__Lactobacillales.f__Lactobacillaceae.g__Lactobacillus.s__amylovorus.t__GRL1118
1.000
d__Bacteria.p__Firmicutes.c__Bacilli.o__Lactobacillales.f__Lactobacillaceae.g__Lactobacillus.s__acidophilus.t__30SC
0.676
d__Bacteria.p__Firmicutes.c__Bacilli.o__Lactobacillales.f__Lactobacillaceae.g__Lactobacillus.s__amylovorus.t__GRL_1112
d__Bacteria.p__Firmicutes.c__Bacilli.o__Lactobacillales.f__Lactobacillaceae.g__Lactobacillus.s__kefiranofaciens.t__ZW3
d__Bacteria.p__Firmicutes.c__Bacilli.o__Lactobacillales.f__Lactobacillaceae.g__Lactobacillus.s__helveticus.t__H10
d__Bacteria.p__Firmicutes.c__Bacilli.o__Lactobacillales.f__Lactobacillaceae.g__Lactobacillus.s__helveticus.t__DSM_20075
0.999
d__Bacteria.p__Firmicutes.c__Bacilli.o__Lactobacillales.f__Lactobacillaceae.g__Lactobacillus.s__helveticus.t__DPC_4571
d__Bacteria.p__Firmicutes.c__Bacilli.o__Lactobacillales.f__Lactobacillaceae.g__Lactobacillus.s__sakei.t__subsp__sakei_23K
d__Bacteria.p__Firmicutes.c__Bacilli.o__Lactobacillales.f__Lactobacillaceae.g__Lactobacillus.s__paracasei.t__subsp__paracasei_ATCC_25302
d__Bacteria.p__Firmicutes.c__Bacilli.o__Lactobacillales.f__Lactobacillaceae.g__Lactobacillus.s__casei.t__LC2W
0.887
d__Bacteria.p__Firmicutes.c__Bacilli.o__Lactobacillales.f__Lactobacillaceae.g__Lactobacillus.s__casei.t__BL23
0.000
0.998
d__Bacteria.p__Firmicutes.c__Bacilli.o__Lactobacillales.f__Lactobacillaceae.g__Lactobacillus.s__casei.t__BD_II
1.000
d__Bacteria.p__Firmicutes.c__Bacilli.o__Lactobacillales.f__Lactobacillaceae.g__Lactobacillus.s__casei.t__ATCC_334
d__Bacteria.p__Firmicutes.c__Bacilli.o__Lactobacillales.f__Lactobacillaceae.g__Lactobacillus.s__paracasei.t__subsp__paracasei_8700_2
0.940
d__Bacteria.p__Firmicutes.c__Bacilli.o__Lactobacillales.f__Lactobacillaceae.g__Lactobacillus.s__casei.t__str__Zhang
1.000
d__Bacteria.p__Firmicutes.c__Bacilli.o__Lactobacillales.f__Lactobacillaceae.g__Lactobacillus.s__rhamnosus.t__HN001
d__Bacteria.p__Firmicutes.c__Bacilli.o__Lactobacillales.f__Lactobacillaceae.g__Lactobacillus.s__rhamnosus.t__GG
d__Bacteria.p__Firmicutes.c__Bacilli.o__Lactobacillales.f__Lactobacillaceae.g__Lactobacillus.s__rhamnosus.t__GG
1.000
d__Bacteria.p__Firmicutes.c__Bacilli.o__Lactobacillales.f__Lactobacillaceae.g__Lactobacillus.s__rhamnosus.t__LMS2_1
d__Bacteria.p__Firmicutes.c__Bacilli.o__Lactobacillales.f__Lactobacillaceae.g__Lactobacillus.s__rhamnosus.t__Lc_705
d__Bacteria.p__Firmicutes.c__Bacilli.o__Lactobacillales.f__Lactobacillaceae.g__Lactobacillus.s__salivarius.t__subsp__salivarius_UCC118
0.214
d__Bacteria.p__Firmicutes.c__Bacilli.o__Lactobacillales.f__Lactobacillaceae.g__Lactobacillus.s__salivarius.t__ACS_116_V_Col5a
1.000
d__Bacteria.p__Firmicutes.c__Bacilli.o__Lactobacillales.f__Lactobacillaceae.g__Lactobacillus.s__salivarius.t__ATCC_11741
0.845
1.000
d__Bacteria.p__Firmicutes.c__Bacilli.o__Lactobacillales.f__Lactobacillaceae.g__Lactobacillus.s__salivarius.t__CECT_5713
d__Bacteria.p__Firmicutes.c__Bacilli.o__Lactobacillales.f__Lactobacillaceae.g__Lactobacillus.s__animalis.t__KCTC_3501
1.000
d__Bacteria.p__Firmicutes.c__Bacilli.o__Lactobacillales.f__Lactobacillaceae.g__Lactobacillus.s__ruminis.t__ATCC_25644
1.000
d__Bacteria.p__Firmicutes.c__Bacilli.o__Lactobacillales.f__Lactobacillaceae.g__Lactobacillus.s__ruminis.t__SPM0211
d__Bacteria.p__Firmicutes.c__Bacilli.o__Lactobacillales.f__Lactobacillaceae.g__Pediococcus.s__pentosaceus.t__ATCC_25745
d__Bacteria.p__Firmicutes.c__Bacilli.o__Lactobacillales.f__Lactobacillaceae.g__Pediococcus.s__acidilactici.t__7_4
1.000
d__Bacteria.p__Firmicutes.c__Bacilli.o__Lactobacillales.f__Lactobacillaceae.g__Pediococcus.s__acidilactici.t__DSM_20284
d__Bacteria.p__Firmicutes.c__Bacilli.o__Lactobacillales.f__Lactobacillaceae.g__Lactobacillus.s__plantarum.t__JDM1
0.401
d__Bacteria.p__Firmicutes.c__Bacilli.o__Lactobacillales.f__Lactobacillaceae.g__Lactobacillus.s__plantarum.t__WCFS1
d__Bacteria.p__Firmicutes.c__Bacilli.o__Lactobacillales.f__Lactobacillaceae.g__Lactobacillus.s__plantarum.t__subsp__plantarum_ATCC_14917
0.903
1.000
d__Bacteria.p__Firmicutes.c__Bacilli.o__Lactobacillales.f__Lactobacillaceae.g__Lactobacillus.s__plantarum.t__subsp__plantarum_ST_III
0.999
d__Bacteria.p__Firmicutes.c__Bacilli.o__Lactobacillales.f__Lactobacillaceae.g__Lactobacillus.s__fructivorans.t__KCTC_3543
1.000
d__Bacteria.p__Firmicutes.c__Bacilli.o__Lactobacillales.f__Lactobacillaceae.g__Lactobacillus.s__hilgardii.t__ATCC_8290
d__Bacteria.p__Firmicutes.c__Bacilli.o__Lactobacillales.f__Lactobacillaceae.g__Lactobacillus.s__brevis.t__subsp__gravesensis_ATCC_27305
0.847
1.000
d__Bacteria.p__Firmicutes.c__Bacilli.o__Lactobacillales.f__Lactobacillaceae.g__Lactobacillus.s__buchneri.t__ATCC_11577
d__Bacteria.p__Firmicutes.c__Bacilli.o__Lactobacillales.f__Lactobacillaceae.g__Lactobacillus.s__brevis.t__ATCC_367
d__Bacteria.p__Firmicutes.c__Bacilli.o__Lactobacillales.f__Leuconostocaceae.g__Weissella.s__koreensis.t__KACC_15510
1.000
d__Bacteria.p__Firmicutes.c__Bacilli.o__Lactobacillales.f__Leuconostocaceae.g__Weissella.s__cibaria.t__KACC_11862
1.000
d__Bacteria.p__Firmicutes.c__Bacilli.o__Lactobacillales.f__Leuconostocaceae.g__Weissella.s__paramesenteroides.t__ATCC_33313
1.000
1.000
d__Bacteria.p__Firmicutes.c__Bacilli.o__Lactobacillales.f__Leuconostocaceae.g__Oenococcus.s__oeni.t__ATCC_BAA_1163
d__Bacteria.p__Firmicutes.c__Bacilli.o__Lactobacillales.f__Leuconostocaceae.g__Oenococcus.s__oeni.t__AWRIB429
0.975
d__Bacteria.p__Firmicutes.c__Bacilli.o__Lactobacillales.f__Leuconostocaceae.g__Oenococcus.s__oeni.t__PSU_1
1.000
d__Bacteria.p__Firmicutes.c__Bacilli.o__Lactobacillales.f__Leuconostocaceae.g__Fructobacillus.s__fructosus.t__KCTC_3544
1.000
d__Bacteria.p__Firmicutes.c__Bacilli.o__Lactobacillales.f__Leuconostocaceae.g__Leuconostoc.s__fallax.t__KCTC_3537
d__Bacteria.p__Firmicutes.c__Bacilli.o__Lactobacillales.f__Leuconostocaceae.g__Leuconostoc.s__mesenteroides.t__subsp__cremoris_ATCC_19254
0.964
1.000
d__Bacteria.p__Firmicutes.c__Bacilli.o__Lactobacillales.f__Leuconostocaceae.g__Leuconostoc.s__mesenteroides.t__subsp__mesenteroides_ATCC_8293
d__Bacteria.p__Firmicutes.c__Bacilli.o__Lactobacillales.f__Leuconostocaceae.g__Leuconostoc.s__argentinum.t__KCTC_3773
d__Bacteria.p__Firmicutes.c__Bacilli.o__Lactobacillales.f__Leuconostocaceae.g__Leuconostoc.s__citreum.t__KM20
d__Bacteria.p__Firmicutes.c__Bacilli.o__Lactobacillales.f__Leuconostocaceae.g__Leuconostoc.s__kimchii.t__IMSNU11154
1.000
0.709
d__Bacteria.p__Firmicutes.c__Bacilli.o__Lactobacillales.f__Leuconostocaceae.g__Leuconostoc.s__sp_.t__C2
1.000
d__Bacteria.p__Firmicutes.c__Bacilli.o__Lactobacillales.f__Leuconostocaceae.g__Leuconostoc.s__gelidum.t__KCTC_3527
d__Bacteria.p__Firmicutes.c__Bacilli.o__Lactobacillales.f__Leuconostocaceae.g__Leuconostoc.s__gasicomitatum.t__LMG_18811
1.000
d__Bacteria.p__Firmicutes.c__Bacilli.o__Lactobacillales.f__Leuconostocaceae.g__Leuconostoc.s__inhae.t__KCTC_3774
d__Bacteria.p__Firmicutes.c__Bacilli.o__Lactobacillales.f__Lactobacillaceae.g__Lactobacillus.s__vaginalis.t__ATCC_49540
d__Bacteria.p__Firmicutes.c__Bacilli.o__Lactobacillales.f__Lactobacillaceae.g__Lactobacillus.s__reuteri.t__CF48_3A
1.000
d__Bacteria.p__Firmicutes.c__Bacilli.o__Lactobacillales.f__Lactobacillaceae.g__Lactobacillus.s__reuteri.t__SD2112
d__Bacteria.p__Firmicutes.c__Bacilli.o__Lactobacillales.f__Lactobacillaceae.g__Lactobacillus.s__reuteri.t__JCM_1112
d__Bacteria.p__Firmicutes.c__Bacilli.o__Lactobacillales.f__Lactobacillaceae.g__Lactobacillus.s__reuteri.t__F275
0.998
d__Bacteria.p__Firmicutes.c__Bacilli.o__Lactobacillales.f__Lactobacillaceae.g__Lactobacillus.s__reuteri.t__100_23
d__Bacteria.p__Firmicutes.c__Bacilli.o__Lactobacillales.f__Lactobacillaceae.g__Lactobacillus.s__reuteri.t__MM2_3
0.000
1.000
d__Bacteria.p__Firmicutes.c__Bacilli.o__Lactobacillales.f__Lactobacillaceae.g__Lactobacillus.s__reuteri.t__MM4_1
d__Bacteria.p__Firmicutes.c__Bacilli.o__Lactobacillales.f__Lactobacillaceae.g__Lactobacillus.s__oris.t__PB013_T2_3
1.000
d__Bacteria.p__Firmicutes.c__Bacilli.o__Lactobacillales.f__Lactobacillaceae.g__Lactobacillus.s__antri.t__DSM_16041
d__Bacteria.p__Firmicutes.c__Bacilli.o__Lactobacillales.f__Lactobacillaceae.g__Lactobacillus.s__coleohominis.t__101_4_CHN
0.994
d__Bacteria.p__Firmicutes.c__Bacilli.o__Lactobacillales.f__Lactobacillaceae.g__Lactobacillus.s__fermentum.t__CECT_5716
d__Bacteria.p__Firmicutes.c__Bacilli.o__Lactobacillales.f__Lactobacillaceae.g__Lactobacillus.s__fermentum.t__IFO_3956
d__Bacteria.p__Firmicutes.c__Bacilli.o__Lactobacillales.f__Lactobacillaceae.g__Lactobacillus.s__fermentum.t__ATCC_14931
0.991
d__Bacteria.p__Firmicutes.c__Bacilli.o__Lactobacillales.f__Lactobacillaceae.g__Lactobacillus.s__fermentum.t__28_3_CHN

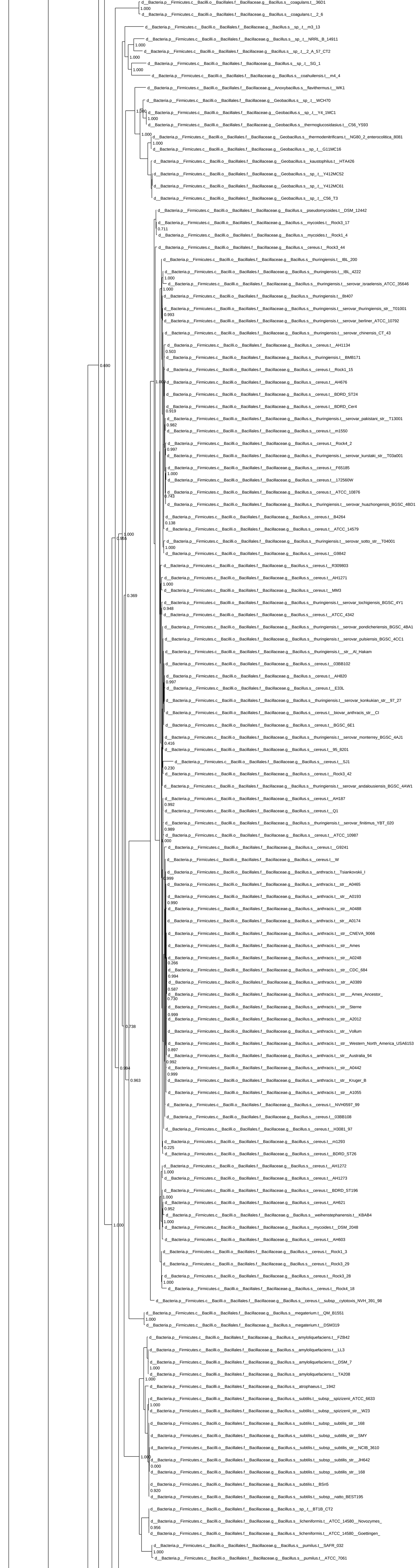

d__Bacteria.p__Firmicutes.c__Bacilli.o__Bacillales.f__Bacillaceae.g__Bacillus.s__coagulans.t__36D1
1.000
d__Bacteria.p__Firmicutes.c__Bacilli.o__Bacillales.f__Bacillaceae.g__Bacillus.s__coagulans.t__2_6
d__Bacteria.p__Firmicutes.c__Bacilli.o__Bacillales.f__Bacillaceae.g__Bacillus.s__sp_.t__m3_13
d__Bacteria.p__Firmicutes.c__Bacilli.o__Bacillales.f__Bacillaceae.g__Bacillus.s__sp_.t__NRRL_B_14911
1.000
d__Bacteria.p__Firmicutes.c__Bacilli.o__Bacillales.f__Bacillaceae.g__Bacillus.s__sp_.t__2_A_57_CT2
1.000
d__Bacteria.p__Firmicutes.c__Bacilli.o__Bacillales.f__Bacillaceae.g__Bacillus.s__sp_.t__SG_1
1.000
d__Bacteria.p__Firmicutes.c__Bacilli.o__Bacillales.f__Bacillaceae.g__Bacillus.s__coahuilensis.t__m4_4
d__Bacteria.p__Firmicutes.c__Bacilli.o__Bacillales.f__Bacillaceae.g__Anoxybacillus.s__flavithermus.t__WK1
d__Bacteria.p__Firmicutes.c__Bacilli.o__Bacillales.f__Bacillaceae.g__Geobacillus.s__sp_.t__WCH70
1.000
d__Bacteria.p__Firmicutes.c__Bacilli.o__Bacillales.f__Bacillaceae.g__Geobacillus.s__sp_.t__Y4_1MC1
1.000
d__Bacteria.p__Firmicutes.c__Bacilli.o__Bacillales.f__Bacillaceae.g__Geobacillus.s__thermoglucosidasius.t__C56_YS93
1.000
d__Bacteria.p__Firmicutes.c__Bacilli.o__Bacillales.f__Bacillaceae.g__Geobacillus.s__thermodenitrificans.t__NG80_2_enterocolitica_8081
1.000
d__Bacteria.p__Firmicutes.c__Bacilli.o__Bacillales.f__Bacillaceae.g__Geobacillus.s__sp_.t__G11MC16
d__Bacteria.p__Firmicutes.c__Bacilli.o__Bacillales.f__Bacillaceae.g__Geobacillus.s__kaustophilus.t__HTA426
d__Bacteria.p__Firmicutes.c__Bacilli.o__Bacillales.f__Bacillaceae.g__Geobacillus.s__sp_.t__Y412MC52
d__Bacteria.p__Firmicutes.c__Bacilli.o__Bacillales.f__Bacillaceae.g__Geobacillus.s__sp_.t__Y412MC61
d__Bacteria.p__Firmicutes.c__Bacilli.o__Bacillales.f__Bacillaceae.g__Geobacillus.s__sp_.t__C56_T3
d__Bacteria.p__Firmicutes.c__Bacilli.o__Bacillales.f__Bacillaceae.g__Bacillus.s__pseudomycoides.t__DSM_12442
d__Bacteria.p__Firmicutes.c__Bacilli.o__Bacillales.f__Bacillaceae.g__Bacillus.s__mycoides.t__Rock3_17
0.711
d__Bacteria.p__Firmicutes.c__Bacilli.o__Bacillales.f__Bacillaceae.g__Bacillus.s__mycoides.t__Rock1_4
d__Bacteria.p__Firmicutes.c__Bacilli.o__Bacillales.f__Bacillaceae.g__Bacillus.s__cereus.t__Rock3_44
d__Bacteria.p__Firmicutes.c__Bacilli.o__Bacillales.f__Bacillaceae.g__Bacillus.s__thuringiensis.t__IBL_200
d__Bacteria.p__Firmicutes.c__Bacilli.o__Bacillales.f__Bacillaceae.g__Bacillus.s__thuringiensis.t__IBL_4222
1.000
d__Bacteria.p__Firmicutes.c__Bacilli.o__Bacillales.f__Bacillaceae.g__Bacillus.s__thuringiensis.t__serovar_israelensis_ATCC_35646
1.000
d__Bacteria.p__Firmicutes.c__Bacilli.o__Bacillales.f__Bacillaceae.g__Bacillus.s__thuringiensis.t__Bt407
d__Bacteria.p__Firmicutes.c__Bacilli.o__Bacillales.f__Bacillaceae.g__Bacillus.s__thuringiensis.t__serovar_thuringiensis_str__T01001
0.993
d__Bacteria.p__Firmicutes.c__Bacilli.o__Bacillales.f__Bacillaceae.g__Bacillus.s__thuringiensis.t__serovar_berliner_ATCC_10792
d__Bacteria.p__Firmicutes.c__Bacilli.o__Bacillales.f__Bacillaceae.g__Bacillus.s__thuringiensis.t__serovar_chinensis_CT_43
d__Bacteria.p__Firmicutes.c__Bacilli.o__Bacillales.f__Bacillaceae.g__Bacillus.s__cereus.t__AH1134
0.503
d__Bacteria.p__Firmicutes.c__Bacilli.o__Bacillales.f__Bacillaceae.g__Bacillus.s__thuringiensis.t__BMB171
d__Bacteria.p__Firmicutes.c__Bacilli.o__Bacillales.f__Bacillaceae.g__Bacillus.s__cereus.t__Rock1_15
1.000
d__Bacteria.p__Firmicutes.c__Bacilli.o__Bacillales.f__Bacillaceae.g__Bacillus.s__cereus.t__AH676
d__Bacteria.p__Firmicutes.c__Bacilli.o__Bacillales.f__Bacillaceae.g__Bacillus.s__cereus.t__BDRD_ST24
d__Bacteria.p__Firmicutes.c__Bacilli.o__Bacillales.f__Bacillaceae.g__Bacillus.s__cereus.t__BDRD_Cer4
0.919
d__Bacteria.p__Firmicutes.c__Bacilli.o__Bacillales.f__Bacillaceae.g__Bacillus.s__thuringiensis.t__serovar_pakistani_str__T13001
0.982
d__Bacteria.p__Firmicutes.c__Bacilli.o__Bacillales.f__Bacillaceae.g__Bacillus.s__cereus.t__m1550
d__Bacteria.p__Firmicutes.c__Bacilli.o__Bacillales.f__Bacillaceae.g__Bacillus.s__cereus.t__Rock4_2
0.997
d__Bacteria.p__Firmicutes.c__Bacilli.o__Bacillales.f__Bacillaceae.g__Bacillus.s__thuringiensis.t__serovar_kurstaki_str__T03a001
d__Bacteria.p__Firmicutes.c__Bacilli.o__Bacillales.f__Bacillaceae.g__Bacillus.s__cereus.t__F65185
1.000
d__Bacteria.p__Firmicutes.c__Bacilli.o__Bacillales.f__Bacillaceae.g__Bacillus.s__cereus.t__172560W
d__Bacteria.p__Firmicutes.c__Bacilli.o__Bacillales.f__Bacillaceae.g__Bacillus.s__cereus.t__ATCC_10876
0.743
d__Bacteria.p__Firmicutes.c__Bacilli.o__Bacillales.f__Bacillaceae.g__Bacillus.s__thuringiensis.t__serovar_huazhongensis_BGSC_4BD1
d__Bacteria.p__Firmicutes.c__Bacilli.o__Bacillales.f__Bacillaceae.g__Bacillus.s__cereus.t__B4264
0.138
d__Bacteria.p__Firmicutes.c__Bacilli.o__Bacillales.f__Bacillaceae.g__Bacillus.s__cereus.t__ATCC_14579
d__Bacteria.p__Firmicutes.c__Bacilli.o__Bacillales.f__Bacillaceae.g__Bacillus.s__thuringiensis.t__serovar_sotto_str__T04001
1.000
d__Bacteria.p__Firmicutes.c__Bacilli.o__Bacillales.f__Bacillaceae.g__Bacillus.s__cereus.t__G9842
d__Bacteria.p__Firmicutes.c__Bacilli.o__Bacillales.f__Bacillaceae.g__Bacillus.s__cereus.t__R309803
d__Bacteria.p__Firmicutes.c__Bacilli.o__Bacillales.f__Bacillaceae.g__Bacillus.s__cereus.t__AH1271
1.000
d__Bacteria.p__Firmicutes.c__Bacilli.o__Bacillales.f__Bacillaceae.g__Bacillus.s__cereus.t__MM3
d__Bacteria.p__Firmicutes.c__Bacilli.o__Bacillales.f__Bacillaceae.g__Bacillus.s__thuringiensis.t__serovar_tochigiensis_BGSC_4Y1
0.948
d__Bacteria.p__Firmicutes.c__Bacilli.o__Bacillales.f__Bacillaceae.g__Bacillus.s__cereus.t__ATCC_4342
d__Bacteria.p__Firmicutes.c__Bacilli.o__Bacillales.f__Bacillaceae.g__Bacillus.s__thuringiensis.t__serovar_pondicheriensis_BGSC_4BA1
d__Bacteria.p__Firmicutes.c__Bacilli.o__Bacillales.f__Bacillaceae.g__Bacillus.s__thuringiensis.t__serovar_pulsiensis_BGSC_4CC1
d__Bacteria.p__Firmicutes.c__Bacilli.o__Bacillales.f__Bacillaceae.g__Bacillus.s__thuringiensis.t__str__Al_Hakam
d__Bacteria.p__Firmicutes.c__Bacilli.o__Bacillales.f__Bacillaceae.g__Bacillus.s__cereus.t__03BB102
d__Bacteria.p__Firmicutes.c__Bacilli.o__Bacillales.f__Bacillaceae.g__Bacillus.s__cereus.t__AH820
0.997
d__Bacteria.p__Firmicutes.c__Bacilli.o__Bacillales.f__Bacillaceae.g__Bacillus.s__cereus.t__E33L
d__Bacteria.p__Firmicutes.c__Bacilli.o__Bacillales.f__Bacillaceae.g__Bacillus.s__thuringiensis.t__serovar_konkukian_str__97_27
d__Bacteria.p__Firmicutes.c__Bacilli.o__Bacillales.f__Bacillaceae.g__Bacillus.s__cereus.t__biovar_anthracis_str__CI
d__Bacteria.p__Firmicutes.c__Bacilli.o__Bacillales.f__Bacillaceae.g__Bacillus.s__cereus.t__BGSC_6E1
d__Bacteria.p__Firmicutes.c__Bacilli.o__Bacillales.f__Bacillaceae.g__Bacillus.s__thuringiensis.t__serovar_monterrey_BGSC_4AJ1
0.416
d__Bacteria.p__Firmicutes.c__Bacilli.o__Bacillales.f__Bacillaceae.g__Bacillus.s__cereus.t__95_8201
d__Bacteria.p__Firmicutes.c__Bacilli.o__Bacillales.f__Bacillaceae.g__Bacillus.s__cereus.t__SJ1
0.230
d__Bacteria.p__Firmicutes.c__Bacilli.o__Bacillales.f__Bacillaceae.g__Bacillus.s__cereus.t__Rock3_42
d__Bacteria.p__Firmicutes.c__Bacilli.o__Bacillales.f__Bacillaceae.g__Bacillus.s__thuringiensis.t__serovar_andalousiensis_BGSC_4AW1
d__Bacteria.p__Firmicutes.c__Bacilli.o__Bacillales.f__Bacillaceae.g__Bacillus.s__cereus.t__AH187
0.992
d__Bacteria.p__Firmicutes.c__Bacilli.o__Bacillales.f__Bacillaceae.g__Bacillus.s__cereus.t__Q1
d__Bacteria.p__Firmicutes.c__Bacilli.o__Bacillales.f__Bacillaceae.g__Bacillus.s__thuringiensis.t__serovar_finitimus_YBT_020
0.989
d__Bacteria.p__Firmicutes.c__Bacilli.o__Bacillales.f__Bacillaceae.g__Bacillus.s__cereus.t__ATCC_10987
1.000
d__Bacteria.p__Firmicutes.c__Bacilli.o__Bacillales.f__Bacillaceae.g__Bacillus.s__cereus.t__G9241
d__Bacteria.p__Firmicutes.c__Bacilli.o__Bacillales.f__Bacillaceae.g__Bacillus.s__cereus.t__W
d__Bacteria.p__Firmicutes.c__Bacilli.o__Bacillales.f__Bacillaceae.g__Bacillus.s__anthracis.t__Tsiankovskii_I
0.999
d__Bacteria.p__Firmicutes.c__Bacilli.o__Bacillales.f__Bacillaceae.g__Bacillus.s__anthracis.t__str__A0465
d__Bacteria.p__Firmicutes.c__Bacilli.o__Bacillales.f__Bacillaceae.g__Bacillus.s__anthracis.t__str__A0193
0.990
d__Bacteria.p__Firmicutes.c__Bacilli.o__Bacillales.f__Bacillaceae.g__Bacillus.s__anthracis.t__str__A0488
d__Bacteria.p__Firmicutes.c__Bacilli.o__Bacillales.f__Bacillaceae.g__Bacillus.s__anthracis.t__str__A0174
d__Bacteria.p__Firmicutes.c__Bacilli.o__Bacillales.f__Bacillaceae.g__Bacillus.s__anthracis.t__str__CNEVA_9066
d__Bacteria.p__Firmicutes.c__Bacilli.o__Bacillales.f__Bacillaceae.g__Bacillus.s__anthracis.t__str__Ames
d__Bacteria.p__Firmicutes.c__Bacilli.o__Bacillales.f__Bacillaceae.g__Bacillus.s__anthracis.t__str__A0248
0.266
d__Bacteria.p__Firmicutes.c__Bacilli.o__Bacillales.f__Bacillaceae.g__Bacillus.s__anthracis.t__str__CDC_684
0.994
d__Bacteria.p__Firmicutes.c__Bacilli.o__Bacillales.f__Bacillaceae.g__Bacillus.s__anthracis.t__str__A0389
0.587
d__Bacteria.p__Firmicutes.c__Bacilli.o__Bacillales.f__Bacillaceae.g__Bacillus.s__anthracis.t__str___Ames_Ancestor_
0.730
d__Bacteria.p__Firmicutes.c__Bacilli.o__Bacillales.f__Bacillaceae.g__Bacillus.s__anthracis.t__str__Sterne
0.999
d__Bacteria.p__Firmicutes.c__Bacilli.o__Bacillales.f__Bacillaceae.g__Bacillus.s__anthracis.t__str__A2012
d__Bacteria.p__Firmicutes.c__Bacilli.o__Bacillales.f__Bacillaceae.g__Bacillus.s__anthracis.t__str__Vollum
d__Bacteria.p__Firmicutes.c__Bacilli.o__Bacillales.f__Bacillaceae.g__Bacillus.s__anthracis.t__str__Western_North_America_USA6153
0.897
d__Bacteria.p__Firmicutes.c__Bacilli.o__Bacillales.f__Bacillaceae.g__Bacillus.s__anthracis.t__str__Australia_94
0.992
d__Bacteria.p__Firmicutes.c__Bacilli.o__Bacillales.f__Bacillaceae.g__Bacillus.s__anthracis.t__str__A0442
0.999
d__Bacteria.p__Firmicutes.c__Bacilli.o__Bacillales.f__Bacillaceae.g__Bacillus.s__anthracis.t__str__Kruger_B
d__Bacteria.p__Firmicutes.c__Bacilli.o__Bacillales.f__Bacillaceae.g__Bacillus.s__anthracis.t__str__A1055
d__Bacteria.p__Firmicutes.c__Bacilli.o__Bacillales.f__Bacillaceae.g__Bacillus.s__cereus.t__NVH0597_99
d__Bacteria.p__Firmicutes.c__Bacilli.o__Bacillales.f__Bacillaceae.g__Bacillus.s__cereus.t__03BB108
d__Bacteria.p__Firmicutes.c__Bacilli.o__Bacillales.f__Bacillaceae.g__Bacillus.s__cereus.t__H3081_97
d__Bacteria.p__Firmicutes.c__Bacilli.o__Bacillales.f__Bacillaceae.g__Bacillus.s__cereus.t__m1293
0.225
d__Bacteria.p__Firmicutes.c__Bacilli.o__Bacillales.f__Bacillaceae.g__Bacillus.s__cereus.t__BDRD_ST26
d__Bacteria.p__Firmicutes.c__Bacilli.o__Bacillales.f__Bacillaceae.g__Bacillus.s__cereus.t__AH1272
1.000
d__Bacteria.p__Firmicutes.c__Bacilli.o__Bacillales.f__Bacillaceae.g__Bacillus.s__cereus.t__AH1273
d__Bacteria.p__Firmicutes.c__Bacilli.o__Bacillales.f__Bacillaceae.g__Bacillus.s__cereus.t__BDRD_ST196
1.000
d__Bacteria.p__Firmicutes.c__Bacilli.o__Bacillales.f__Bacillaceae.g__Bacillus.s__cereus.t__AH621
0.952
d__Bacteria.p__Firmicutes.c__Bacilli.o__Bacillales.f__Bacillaceae.g__Bacillus.s__weihenstephanensis.t__KBAB4
1.000
d__Bacteria.p__Firmicutes.c__Bacilli.o__Bacillales.f__Bacillaceae.g__Bacillus.s__mycoides.t__DSM_2048
d__Bacteria.p__Firmicutes.c__Bacilli.o__Bacillales.f__Bacillaceae.g__Bacillus.s__cereus.t__AH603
d__Bacteria.p__Firmicutes.c__Bacilli.o__Bacillales.f__Bacillaceae.g__Bacillus.s__cereus.t__Rock1_3
d__Bacteria.p__Firmicutes.c__Bacilli.o__Bacillales.f__Bacillaceae.g__Bacillus.s__cereus.t__Rock3_29
d__Bacteria.p__Firmicutes.c__Bacilli.o__Bacillales.f__Bacillaceae.g__Bacillus.s__cereus.t__Rock3_28
1.000
d__Bacteria.p__Firmicutes.c__Bacilli.o__Bacillales.f__Bacillaceae.g__Bacillus.s__cereus.t__Rock4_18
d__Bacteria.p__Firmicutes.c__Bacilli.o__Bacillales.f__Bacillaceae.g__Bacillus.s__cereus.t__subsp__cytotoxis_NVH_391_98
d__Bacteria.p__Firmicutes.c__Bacilli.o__Bacillales.f__Bacillaceae.g__Bacillus.s__megaterium.t__QM_B1551
1.000
d__Bacteria.p__Firmicutes.c__Bacilli.o__Bacillales.f__Bacillaceae.g__Bacillus.s__megaterium.t__DSM319
d__Bacteria.p__Firmicutes.c__Bacilli.o__Bacillales.f__Bacillaceae.g__Bacillus.s__amyloliquefaciens.t__FZB42
d__Bacteria.p__Firmicutes.c__Bacilli.o__Bacillales.f__Bacillaceae.g__Bacillus.s__amyloliquefaciens.t__LL3
d__Bacteria.p__Firmicutes.c__Bacilli.o__Bacillales.f__Bacillaceae.g__Bacillus.s__amyloliquefaciens.t__DSM_7
1.000
d__Bacteria.p__Firmicutes.c__Bacilli.o__Bacillales.f__Bacillaceae.g__Bacillus.s__amyloliquefaciens.t__TA208
1.000
d__Bacteria.p__Firmicutes.c__Bacilli.o__Bacillales.f__Bacillaceae.g__Bacillus.s__atrophaeus.t__1942
d__Bacteria.p__Firmicutes.c__Bacilli.o__Bacillales.f__Bacillaceae.g__Bacillus.s__subtilis.t__subsp__spizizenii_ATCC_6633
1.000
d__Bacteria.p__Firmicutes.c__Bacilli.o__Bacillales.f__Bacillaceae.g__Bacillus.s__subtilis.t__subsp__spizizenii_str__W23
d__Bacteria.p__Firmicutes.c__Bacilli.o__Bacillales.f__Bacillaceae.g__Bacillus.s__subtilis.t__subsp__subtilis_str__168
d__Bacteria.p__Firmicutes.c__Bacilli.o__Bacillales.f__Bacillaceae.g__Bacillus.s__subtilis.t__subsp__subtilis_str__SMY
d__Bacteria.p__Firmicutes.c__Bacilli.o__Bacillales.f__Bacillaceae.g__Bacillus.s__subtilis.t__subsp__subtilis_str__NCIB_3610
1.000
d__Bacteria.p__Firmicutes.c__Bacilli.o__Bacillales.f__Bacillaceae.g__Bacillus.s__subtilis.t__subsp__subtilis_str__JH642
0.000
d__Bacteria.p__Firmicutes.c__Bacilli.o__Bacillales.f__Bacillaceae.g__Bacillus.s__subtilis.t__subsp__subtilis_str__168
d__Bacteria.p__Firmicutes.c__Bacilli.o__Bacillales.f__Bacillaceae.g__Bacillus.s__subtilis.t__BSn5
0.920
d__Bacteria.p__Firmicutes.c__Bacilli.o__Bacillales.f__Bacillaceae.g__Bacillus.s__subtilis.t__subsp__natto_BEST195
d__Bacteria.p__Firmicutes.c__Bacilli.o__Bacillales.f__Bacillaceae.g__Bacillus.s__sp_.t__BT1B_CT2
d__Bacteria.p__Firmicutes.c__Bacilli.o__Bacillales.f__Bacillaceae.g__Bacillus.s__licheniformis.t__ATCC_14580__Novozymes_
0.956
d__Bacteria.p__Firmicutes.c__Bacilli.o__Bacillales.f__Bacillaceae.g__Bacillus.s__licheniformis.t__ATCC_14580__Goettingen_
d__Bacteria.p__Firmicutes.c__Bacilli.o__Bacillales.f__Bacillaceae.g__Bacillus.s__pumilus.t__SAFR_032
1.000
d__Bacteria.p__Firmicutes.c__Bacilli.o__Bacillales.f__Bacillaceae.g__Bacillus.s__pumilus.t__ATCC_7061
0.690
1.000
0.955
0.369
0.738
0.994
0.963
1.000

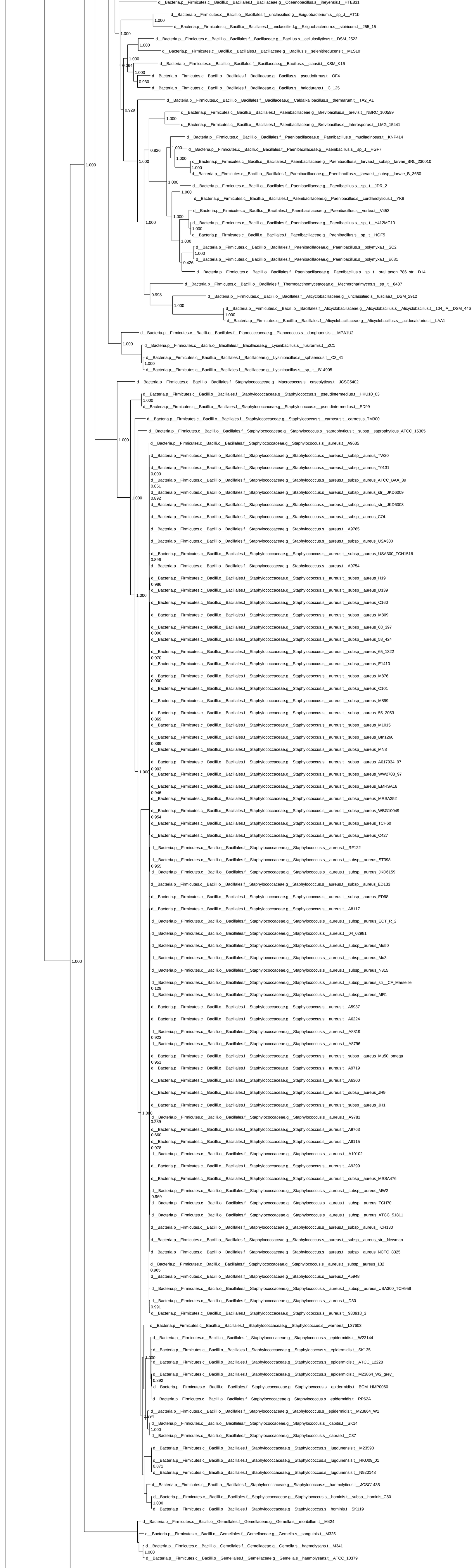
d__Bacteria.p__Firmicutes.c__Bacilli.o__Bacillales.f__Bacillaceae.g__Oceanobacillus.s__iheyensis.t__HTE831
d__Bacteria.p__Firmicutes.c__Bacilli.o__Bacillales.f__unclassified.g__Exiguobacterium.s__sp_.t__AT1b
1.000
d__Bacteria.p__Firmicutes.c__Bacilli.o__Bacillales.f__unclassified.g__Exiguobacterium.s__sibiricum.t__255_15
1.000
d__Bacteria.p__Firmicutes.c__Bacilli.o__Bacillales.f__Bacillaceae.g__Bacillus.s__cellulosilyticus.t__DSM_2522
1.000
d__Bacteria.p__Firmicutes.c__Bacilli.o__Bacillales.f__Bacillaceae.g__Bacillus.s__selenitireducens.t__MLS10
1.000
0.064
d__Bacteria.p__Firmicutes.c__Bacilli.o__Bacillales.f__Bacillaceae.g__Bacillus.s__clausii.t__KSM_K16
1.000
d__Bacteria.p__Firmicutes.c__Bacilli.o__Bacillales.f__Bacillaceae.g__Bacillus.s__pseudofirmus.t__OF4
0.930
d__Bacteria.p__Firmicutes.c__Bacilli.o__Bacillales.f__Bacillaceae.g__Bacillus.s__halodurans.t__C_125
d__Bacteria.p__Firmicutes.c__Bacilli.o__Bacillales.f__Bacillaceae.g__Caldalkalibacillus.s__thermarum.t__TA2_A1
0.929
d__Bacteria.p__Firmicutes.c__Bacilli.o__Bacillales.f__Paenibacillaceae.g__Brevibacillus.s__brevis.t__NBRC_100599
1.000
d__Bacteria.p__Firmicutes.c__Bacilli.o__Bacillales.f__Paenibacillaceae.g__Brevibacillus.s__laterosporus.t__LMG_15441
d__Bacteria.p__Firmicutes.c__Bacilli.o__Bacillales.f__Paenibacillaceae.g__Paenibacillus.s__mucilaginosus.t__KNP414
1.000
0.826
d__Bacteria.p__Firmicutes.c__Bacilli.o__Bacillales.f__Paenibacillaceae.g__Paenibacillus.s__sp_.t__HGF7
1.000
d__Bacteria.p__Firmicutes.c__Bacilli.o__Bacillales.f__Paenibacillaceae.g__Paenibacillus.s__larvae.t__subsp__larvae_BRL_230010
1.000
1.000
1.000
d__Bacteria.p__Firmicutes.c__Bacilli.o__Bacillales.f__Paenibacillaceae.g__Paenibacillus.s__larvae.t__subsp__larvae_B_3650
1.000
d__Bacteria.p__Firmicutes.c__Bacilli.o__Bacillales.f__Paenibacillaceae.g__Paenibacillus.s__sp_.t__JDR_2
1.000
d__Bacteria.p__Firmicutes.c__Bacilli.o__Bacillales.f__Paenibacillaceae.g__Paenibacillus.s__curdlanolyticus.t__YK9
d__Bacteria.p__Firmicutes.c__Bacilli.o__Bacillales.f__Paenibacillaceae.g__Paenibacillus.s__vortex.t__V453
1.000
d__Bacteria.p__Firmicutes.c__Bacilli.o__Bacillales.f__Paenibacillaceae.g__Paenibacillus.s__sp_.t__Y412MC10
1.000
1.000
d__Bacteria.p__Firmicutes.c__Bacilli.o__Bacillales.f__Paenibacillaceae.g__Paenibacillus.s__sp_.t__HGF5
1.000
d__Bacteria.p__Firmicutes.c__Bacilli.o__Bacillales.f__Paenibacillaceae.g__Paenibacillus.s__polymyxa.t__SC2
1.000
d__Bacteria.p__Firmicutes.c__Bacilli.o__Bacillales.f__Paenibacillaceae.g__Paenibacillus.s__polymyxa.t__E681
0.426
d__Bacteria.p__Firmicutes.c__Bacilli.o__Bacillales.f__Paenibacillaceae.g__Paenibacillus.s__sp_.t__oral_taxon_786_str__D14
d__Bacteria.p__Firmicutes.c__Bacilli.o__Bacillales.f__Thermoactinomycetaceae.g__Mechercharimyces.s__sp_.t__8437
0.998
d__Bacteria.p__Firmicutes.c__Bacilli.o__Bacillales.f__Alicyclobacillaceae.g__unclassified.s__tusciae.t__DSM_2912
1.000
d__Bacteria.p__Firmicutes.c__Bacilli.o__Bacillales.f__Alicyclobacillaceae.g__Alicyclobacillus.s__Alicyclobacillus.t__104_IA__DSM_446
1.000
d__Bacteria.p__Firmicutes.c__Bacilli.o__Bacillales.f__Alicyclobacillaceae.g__Alicyclobacillus.s__acidocaldarius.t__LAA1
d__Bacteria.p__Firmicutes.c__Bacilli.o__Bacillales.f__Planococcaceae.g__Planococcus.s__donghaensis.t__MPA1U2
1.000
d__Bacteria.p__Firmicutes.c__Bacilli.o__Bacillales.f__Bacillaceae.g__Lysinibacillus.s__fusiformis.t__ZC1
d__Bacteria.p__Firmicutes.c__Bacilli.o__Bacillales.f__Bacillaceae.g__Lysinibacillus.s__sphaericus.t__C3_41
1.000
d__Bacteria.p__Firmicutes.c__Bacilli.o__Bacillales.f__Bacillaceae.g__Lysinibacillus.s__sp_.t__B14905
d__Bacteria.p__Firmicutes.c__Bacilli.o__Bacillales.f__Staphylococcaceae.g__Macrococcus.s__caseolyticus.t__JCSC5402
d__Bacteria.p__Firmicutes.c__Bacilli.o__Bacillales.f__Staphylococcaceae.g__Staphylococcus.s__pseudintermedius.t__HKU10_03
1.000
d__Bacteria.p__Firmicutes.c__Bacilli.o__Bacillales.f__Staphylococcaceae.g__Staphylococcus.s__pseudintermedius.t__ED99
d__Bacteria.p__Firmicutes.c__Bacilli.o__Bacillales.f__Staphylococcaceae.g__Staphylococcus.s__carnosus.t__carnosus_TM300
d__Bacteria.p__Firmicutes.c__Bacilli.o__Bacillales.f__Staphylococcaceae.g__Staphylococcus.s__saprophyticus.t__subsp__saprophyticus_ATCC_15305
1.000
d__Bacteria.p__Firmicutes.c__Bacilli.o__Bacillales.f__Staphylococcaceae.g__Staphylococcus.s__aureus.t__A9635
d__Bacteria.p__Firmicutes.c__Bacilli.o__Bacillales.f__Staphylococcaceae.g__Staphylococcus.s__aureus.t__subsp__aureus_TW20
d__Bacteria.p__Firmicutes.c__Bacilli.o__Bacillales.f__Staphylococcaceae.g__Staphylococcus.s__aureus.t__subsp__aureus_T0131
0.000
d__Bacteria.p__Firmicutes.c__Bacilli.o__Bacillales.f__Staphylococcaceae.g__Staphylococcus.s__aureus.t__subsp__aureus_ATCC_BAA_39
0.851
d__Bacteria.p__Firmicutes.c__Bacilli.o__Bacillales.f__Staphylococcaceae.g__Staphylococcus.s__aureus.t__subsp__aureus_str__JKD6009
0.892
1.000
d__Bacteria.p__Firmicutes.c__Bacilli.o__Bacillales.f__Staphylococcaceae.g__Staphylococcus.s__aureus.t__subsp__aureus_str__JKD6008
d__Bacteria.p__Firmicutes.c__Bacilli.o__Bacillales.f__Staphylococcaceae.g__Staphylococcus.s__aureus.t__subsp__aureus_COL
d__Bacteria.p__Firmicutes.c__Bacilli.o__Bacillales.f__Staphylococcaceae.g__Staphylococcus.s__aureus.t__A9765
d__Bacteria.p__Firmicutes.c__Bacilli.o__Bacillales.f__Staphylococcaceae.g__Staphylococcus.s__aureus.t__subsp__aureus_USA300
d__Bacteria.p__Firmicutes.c__Bacilli.o__Bacillales.f__Staphylococcaceae.g__Staphylococcus.s__aureus.t__subsp__aureus_USA300_TCH1516
0.896
d__Bacteria.p__Firmicutes.c__Bacilli.o__Bacillales.f__Staphylococcaceae.g__Staphylococcus.s__aureus.t__A9754
d__Bacteria.p__Firmicutes.c__Bacilli.o__Bacillales.f__Staphylococcaceae.g__Staphylococcus.s__aureus.t__subsp__aureus_H19
0.986
d__Bacteria.p__Firmicutes.c__Bacilli.o__Bacillales.f__Staphylococcaceae.g__Staphylococcus.s__aureus.t__subsp__aureus_D139
1.000
d__Bacteria.p__Firmicutes.c__Bacilli.o__Bacillales.f__Staphylococcaceae.g__Staphylococcus.s__aureus.t__subsp__aureus_C160
d__Bacteria.p__Firmicutes.c__Bacilli.o__Bacillales.f__Staphylococcaceae.g__Staphylococcus.s__aureus.t__subsp__aureus_M809
d__Bacteria.p__Firmicutes.c__Bacilli.o__Bacillales.f__Staphylococcaceae.g__Staphylococcus.s__aureus.t__subsp__aureus_68_397
0.000
d__Bacteria.p__Firmicutes.c__Bacilli.o__Bacillales.f__Staphylococcaceae.g__Staphylococcus.s__aureus.t__subsp__aureus_58_424
d__Bacteria.p__Firmicutes.c__Bacilli.o__Bacillales.f__Staphylococcaceae.g__Staphylococcus.s__aureus.t__subsp__aureus_65_1322
0.970
d__Bacteria.p__Firmicutes.c__Bacilli.o__Bacillales.f__Staphylococcaceae.g__Staphylococcus.s__aureus.t__subsp__aureus_E1410
d__Bacteria.p__Firmicutes.c__Bacilli.o__Bacillales.f__Staphylococcaceae.g__Staphylococcus.s__aureus.t__subsp__aureus_M876
0.000
d__Bacteria.p__Firmicutes.c__Bacilli.o__Bacillales.f__Staphylococcaceae.g__Staphylococcus.s__aureus.t__subsp__aureus_C101
d__Bacteria.p__Firmicutes.c__Bacilli.o__Bacillales.f__Staphylococcaceae.g__Staphylococcus.s__aureus.t__subsp__aureus_M899
d__Bacteria.p__Firmicutes.c__Bacilli.o__Bacillales.f__Staphylococcaceae.g__Staphylococcus.s__aureus.t__subsp__aureus_55_2053
0.869
d__Bacteria.p__Firmicutes.c__Bacilli.o__Bacillales.f__Staphylococcaceae.g__Staphylococcus.s__aureus.t__subsp__aureus_M1015
d__Bacteria.p__Firmicutes.c__Bacilli.o__Bacillales.f__Staphylococcaceae.g__Staphylococcus.s__aureus.t__subsp__aureus_Btn1260
0.889
d__Bacteria.p__Firmicutes.c__Bacilli.o__Bacillales.f__Staphylococcaceae.g__Staphylococcus.s__aureus.t__subsp__aureus_MN8
d__Bacteria.p__Firmicutes.c__Bacilli.o__Bacillales.f__Staphylococcaceae.g__Staphylococcus.s__aureus.t__subsp__aureus_A017934_97
0.903
1.000
d__Bacteria.p__Firmicutes.c__Bacilli.o__Bacillales.f__Staphylococcaceae.g__Staphylococcus.s__aureus.t__subsp__aureus_WW2703_97
d__Bacteria.p__Firmicutes.c__Bacilli.o__Bacillales.f__Staphylococcaceae.g__Staphylococcus.s__aureus.t__subsp__aureus_EMRSA16
0.946
d__Bacteria.p__Firmicutes.c__Bacilli.o__Bacillales.f__Staphylococcaceae.g__Staphylococcus.s__aureus.t__subsp__aureus_MRSA252
d__Bacteria.p__Firmicutes.c__Bacilli.o__Bacillales.f__Staphylococcaceae.g__Staphylococcus.s__aureus.t__subsp__aureus_WBG10049
0.954
d__Bacteria.p__Firmicutes.c__Bacilli.o__Bacillales.f__Staphylococcaceae.g__Staphylococcus.s__aureus.t__subsp__aureus_TCH60
d__Bacteria.p__Firmicutes.c__Bacilli.o__Bacillales.f__Staphylococcaceae.g__Staphylococcus.s__aureus.t__subsp__aureus_C427
d__Bacteria.p__Firmicutes.c__Bacilli.o__Bacillales.f__Staphylococcaceae.g__Staphylococcus.s__aureus.t__RF122
d__Bacteria.p__Firmicutes.c__Bacilli.o__Bacillales.f__Staphylococcaceae.g__Staphylococcus.s__aureus.t__subsp__aureus_ST398
0.955
d__Bacteria.p__Firmicutes.c__Bacilli.o__Bacillales.f__Staphylococcaceae.g__Staphylococcus.s__aureus.t__subsp__aureus_JKD6159
d__Bacteria.p__Firmicutes.c__Bacilli.o__Bacillales.f__Staphylococcaceae.g__Staphylococcus.s__aureus.t__subsp__aureus_ED133
d__Bacteria.p__Firmicutes.c__Bacilli.o__Bacillales.f__Staphylococcaceae.g__Staphylococcus.s__aureus.t__subsp__aureus_ED98
d__Bacteria.p__Firmicutes.c__Bacilli.o__Bacillales.f__Staphylococcaceae.g__Staphylococcus.s__aureus.t__A8117
d__Bacteria.p__Firmicutes.c__Bacilli.o__Bacillales.f__Staphylococcaceae.g__Staphylococcus.s__aureus.t__subsp__aureus_ECT_R_2
d__Bacteria.p__Firmicutes.c__Bacilli.o__Bacillales.f__Staphylococcaceae.g__Staphylococcus.s__aureus.t__04_02981
d__Bacteria.p__Firmicutes.c__Bacilli.o__Bacillales.f__Staphylococcaceae.g__Staphylococcus.s__aureus.t__subsp__aureus_Mu50
d__Bacteria.p__Firmicutes.c__Bacilli.o__Bacillales.f__Staphylococcaceae.g__Staphylococcus.s__aureus.t__subsp__aureus_Mu3
1.000
d__Bacteria.p__Firmicutes.c__Bacilli.o__Bacillales.f__Staphylococcaceae.g__Staphylococcus.s__aureus.t__subsp__aureus_N315
d__Bacteria.p__Firmicutes.c__Bacilli.o__Bacillales.f__Staphylococcaceae.g__Staphylococcus.s__aureus.t__subsp__aureus_str__CF_Marseille
0.129
d__Bacteria.p__Firmicutes.c__Bacilli.o__Bacillales.f__Staphylococcaceae.g__Staphylococcus.s__aureus.t__subsp__aureus_MR1
d__Bacteria.p__Firmicutes.c__Bacilli.o__Bacillales.f__Staphylococcaceae.g__Staphylococcus.s__aureus.t__A5937
d__Bacteria.p__Firmicutes.c__Bacilli.o__Bacillales.f__Staphylococcaceae.g__Staphylococcus.s__aureus.t__A6224
d__Bacteria.p__Firmicutes.c__Bacilli.o__Bacillales.f__Staphylococcaceae.g__Staphylococcus.s__aureus.t__A8819
0.923
d__Bacteria.p__Firmicutes.c__Bacilli.o__Bacillales.f__Staphylococcaceae.g__Staphylococcus.s__aureus.t__A8796
d__Bacteria.p__Firmicutes.c__Bacilli.o__Bacillales.f__Staphylococcaceae.g__Staphylococcus.s__aureus.t__subsp__aureus_Mu50_omega
0.951
d__Bacteria.p__Firmicutes.c__Bacilli.o__Bacillales.f__Staphylococcaceae.g__Staphylococcus.s__aureus.t__A9719
d__Bacteria.p__Firmicutes.c__Bacilli.o__Bacillales.f__Staphylococcaceae.g__Staphylococcus.s__aureus.t__A6300
d__Bacteria.p__Firmicutes.c__Bacilli.o__Bacillales.f__Staphylococcaceae.g__Staphylococcus.s__aureus.t__subsp__aureus_JH9
d__Bacteria.p__Firmicutes.c__Bacilli.o__Bacillales.f__Staphylococcaceae.g__Staphylococcus.s__aureus.t__subsp__aureus_JH1
1.000
d__Bacteria.p__Firmicutes.c__Bacilli.o__Bacillales.f__Staphylococcaceae.g__Staphylococcus.s__aureus.t__A9781
0.289
d__Bacteria.p__Firmicutes.c__Bacilli.o__Bacillales.f__Staphylococcaceae.g__Staphylococcus.s__aureus.t__A9763
0.660
d__Bacteria.p__Firmicutes.c__Bacilli.o__Bacillales.f__Staphylococcaceae.g__Staphylococcus.s__aureus.t__A8115
0.978
d__Bacteria.p__Firmicutes.c__Bacilli.o__Bacillales.f__Staphylococcaceae.g__Staphylococcus.s__aureus.t__A10102
d__Bacteria.p__Firmicutes.c__Bacilli.o__Bacillales.f__Staphylococcaceae.g__Staphylococcus.s__aureus.t__A9299
d__Bacteria.p__Firmicutes.c__Bacilli.o__Bacillales.f__Staphylococcaceae.g__Staphylococcus.s__aureus.t__subsp__aureus_MSSA476
d__Bacteria.p__Firmicutes.c__Bacilli.o__Bacillales.f__Staphylococcaceae.g__Staphylococcus.s__aureus.t__subsp__aureus_MW2
0.969
d__Bacteria.p__Firmicutes.c__Bacilli.o__Bacillales.f__Staphylococcaceae.g__Staphylococcus.s__aureus.t__subsp__aureus_TCH70
d__Bacteria.p__Firmicutes.c__Bacilli.o__Bacillales.f__Staphylococcaceae.g__Staphylococcus.s__aureus.t__subsp__aureus_ATCC_51811
d__Bacteria.p__Firmicutes.c__Bacilli.o__Bacillales.f__Staphylococcaceae.g__Staphylococcus.s__aureus.t__subsp__aureus_TCH130
d__Bacteria.p__Firmicutes.c__Bacilli.o__Bacillales.f__Staphylococcaceae.g__Staphylococcus.s__aureus.t__subsp__aureus_str__Newman
d__Bacteria.p__Firmicutes.c__Bacilli.o__Bacillales.f__Staphylococcaceae.g__Staphylococcus.s__aureus.t__subsp__aureus_NCTC_8325
d__Bacteria.p__Firmicutes.c__Bacilli.o__Bacillales.f__Staphylococcaceae.g__Staphylococcus.s__aureus.t__subsp__aureus_132
0.965
d__Bacteria.p__Firmicutes.c__Bacilli.o__Bacillales.f__Staphylococcaceae.g__Staphylococcus.s__aureus.t__A5948
d__Bacteria.p__Firmicutes.c__Bacilli.o__Bacillales.f__Staphylococcaceae.g__Staphylococcus.s__aureus.t__subsp__aureus_USA300_TCH959
d__Bacteria.p__Firmicutes.c__Bacilli.o__Bacillales.f__Staphylococcaceae.g__Staphylococcus.s__aureus.t__D30
0.991
d__Bacteria.p__Firmicutes.c__Bacilli.o__Bacillales.f__Staphylococcaceae.g__Staphylococcus.s__aureus.t__930918_3
d__Bacteria.p__Firmicutes.c__Bacilli.o__Bacillales.f__Staphylococcaceae.g__Staphylococcus.s__warneri.t__L37603
d__Bacteria.p__Firmicutes.c__Bacilli.o__Bacillales.f__Staphylococcaceae.g__Staphylococcus.s__epidermidis.t__W23144
d__Bacteria.p__Firmicutes.c__Bacilli.o__Bacillales.f__Staphylococcaceae.g__Staphylococcus.s__epidermidis.t__SK135
1.000
d__Bacteria.p__Firmicutes.c__Bacilli.o__Bacillales.f__Staphylococcaceae.g__Staphylococcus.s__epidermidis.t__ATCC_12228
d__Bacteria.p__Firmicutes.c__Bacilli.o__Bacillales.f__Staphylococcaceae.g__Staphylococcus.s__epidermidis.t__M23864_W2_grey_
0.392
d__Bacteria.p__Firmicutes.c__Bacilli.o__Bacillales.f__Staphylococcaceae.g__Staphylococcus.s__epidermidis.t__BCM_HMP0060
d__Bacteria.p__Firmicutes.c__Bacilli.o__Bacillales.f__Staphylococcaceae.g__Staphylococcus.s__epidermidis.t__RP62A
d__Bacteria.p__Firmicutes.c__Bacilli.o__Bacillales.f__Staphylococcaceae.g__Staphylococcus.s__epidermidis.t__M23864_W1
0.994
d__Bacteria.p__Firmicutes.c__Bacilli.o__Bacillales.f__Staphylococcaceae.g__Staphylococcus.s__capitis.t__SK14
1.000
d__Bacteria.p__Firmicutes.c__Bacilli.o__Bacillales.f__Staphylococcaceae.g__Staphylococcus.s__caprae.t__C87
d__Bacteria.p__Firmicutes.c__Bacilli.o__Bacillales.f__Staphylococcaceae.g__Staphylococcus.s__lugdunensis.t__M23590
d__Bacteria.p__Firmicutes.c__Bacilli.o__Bacillales.f__Staphylococcaceae.g__Staphylococcus.s__lugdunensis.t__HKU09_01
0.871
d__Bacteria.p__Firmicutes.c__Bacilli.o__Bacillales.f__Staphylococcaceae.g__Staphylococcus.s__lugdunensis.t__N920143
d__Bacteria.p__Firmicutes.c__Bacilli.o__Bacillales.f__Staphylococcaceae.g__Staphylococcus.s__haemolyticus.t__JCSC1435
d__Bacteria.p__Firmicutes.c__Bacilli.o__Bacillales.f__Staphylococcaceae.g__Staphylococcus.s__hominis.t__subsp__hominis_C80
1.000
d__Bacteria.p__Firmicutes.c__Bacilli.o__Bacillales.f__Staphylococcaceae.g__Staphylococcus.s__hominis.t__SK119
d__Bacteria.p__Firmicutes.c__Bacilli.o__Gemellales.f__Gemellaceae.g__Gemella.s__moribillum.t__M424
d__Bacteria.p__Firmicutes.c__Bacilli.o__Gemellales.f__Gemellaceae.g__Gemella.s__sanguinis.t__M325
d__Bacteria.p__Firmicutes.c__Bacilli.o__Gemellales.f__Gemellaceae.g__Gemella.s__haemolysans.t__M341
1.000
d__Bacteria.p__Firmicutes.c__Bacilli.o__Gemellales.f__Gemellaceae.g__Gemella.s__haemolysans.t__ATCC_10379

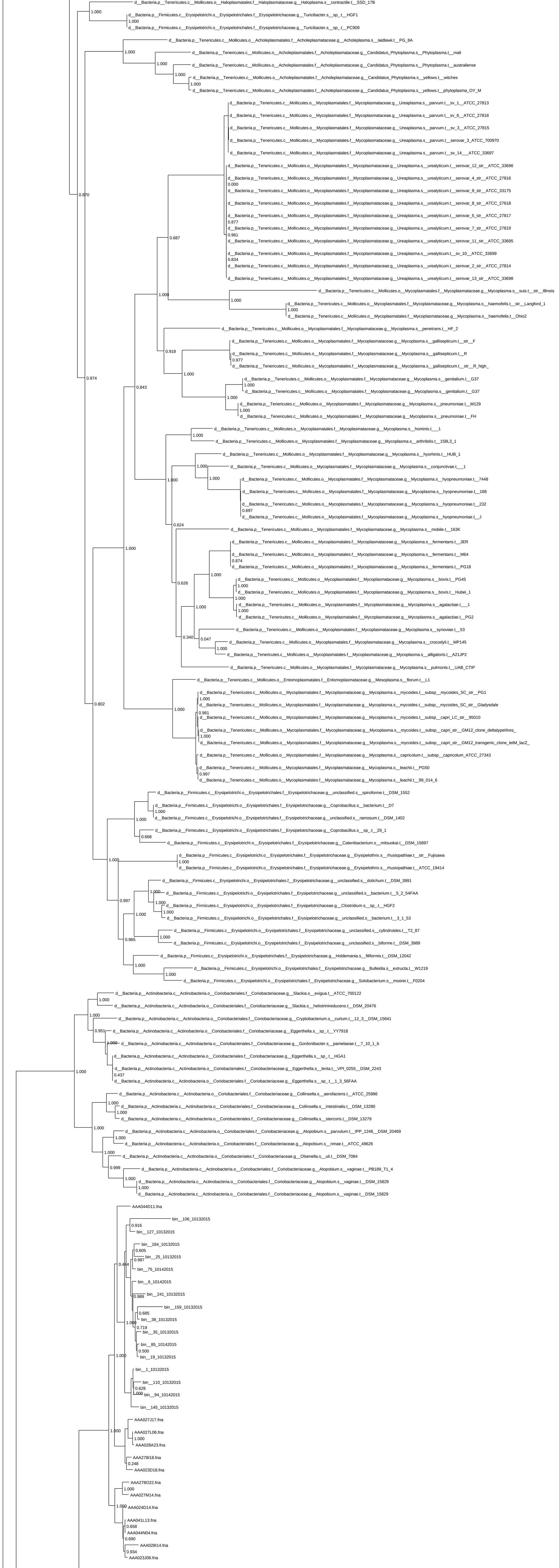

d__Bacteria.p__Tenericutes.c__Mollicutes.o__Haloplasmatales.f__Haloplasmataceae.g__Haloplasma.s__contractile.t__SSD_17B
1.000
d__Bacteria.p__Firmicutes.c__Erysipelotrichi.o__Erysipelotrichales.f__Erysipelotrichaceae.g__Turicibacter.s__sp_.t__HGF1
1.000
d__Bacteria.p__Firmicutes.c__Erysipelotrichi.o__Erysipelotrichales.f__Erysipelotrichaceae.g__Turicibacter.s__sp_.t__PC909
d__Bacteria.p__Tenericutes.c__Mollicutes.o__Acholeplasmatales.f__Acholeplasmataceae.g__Acholeplasma.s__laidlawii.t__PG_8A
1.000
d__Bacteria.p__Tenericutes.c__Mollicutes.o__Acholeplasmatales.f__Acholeplasmataceae.g__Candidatus_Phytoplasma.s__Phytoplasma.t__mali
1.000
d__Bacteria.p__Tenericutes.c__Mollicutes.o__Acholeplasmatales.f__Acholeplasmataceae.g__Candidatus_Phytoplasma.s__Phytoplasma.t__australiense
1.000
d__Bacteria.p__Tenericutes.c__Mollicutes.o__Acholeplasmatales.f__Acholeplasmataceae.g__Candidatus_Phytoplasma.s__yellows.t__witches
1.000
d__Bacteria.p__Tenericutes.c__Mollicutes.o__Acholeplasmatales.f__Acholeplasmataceae.g__Candidatus_Phytoplasma.s__yellows.t__phytoplasma_OY_M
d__Bacteria.p__Tenericutes.c__Mollicutes.o__Mycoplasmatales.f__Mycoplasmataceae.g__Ureaplasma.s__parvum.t__sv_1__ATCC_27813
d__Bacteria.p__Tenericutes.c__Mollicutes.o__Mycoplasmatales.f__Mycoplasmataceae.g__Ureaplasma.s__parvum.t__sv_6__ATCC_27818
d__Bacteria.p__Tenericutes.c__Mollicutes.o__Mycoplasmatales.f__Mycoplasmataceae.g__Ureaplasma.s__parvum.t__sv_3__ATCC_27815
d__Bacteria.p__Tenericutes.c__Mollicutes.o__Mycoplasmatales.f__Mycoplasmataceae.g__Ureaplasma.s__parvum.t__serovar_3_ATCC_700970
d__Bacteria.p__Tenericutes.c__Mollicutes.o__Mycoplasmatales.f__Mycoplasmataceae.g__Ureaplasma.s__parvum.t__sv_14__ATCC_33697
d__Bacteria.p__Tenericutes.c__Mollicutes.o__Mycoplasmatales.f__Mycoplasmataceae.g__Ureaplasma.s__urealyticum.t__serovar_12_str__ATCC_33696
d__Bacteria.p__Tenericutes.c__Mollicutes.o__Mycoplasmatales.f__Mycoplasmataceae.g__Ureaplasma.s__urealyticum.t__serovar_4_str__ATCC_27816
0.000
d__Bacteria.p__Tenericutes.c__Mollicutes.o__Mycoplasmatales.f__Mycoplasmataceae.g__Ureaplasma.s__urealyticum.t__serovar_9_str__ATCC_33175
d__Bacteria.p__Tenericutes.c__Mollicutes.o__Mycoplasmatales.f__Mycoplasmataceae.g__Ureaplasma.s__urealyticum.t__serovar_8_str__ATCC_27618
d__Bacteria.p__Tenericutes.c__Mollicutes.o__Mycoplasmatales.f__Mycoplasmataceae.g__Ureaplasma.s__urealyticum.t__serovar_5_str__ATCC_27817
0.877
d__Bacteria.p__Tenericutes.c__Mollicutes.o__Mycoplasmatales.f__Mycoplasmataceae.g__Ureaplasma.s__urealyticum.t__serovar_7_str__ATCC_27819
0.961
d__Bacteria.p__Tenericutes.c__Mollicutes.o__Mycoplasmatales.f__Mycoplasmataceae.g__Ureaplasma.s__urealyticum.t__serovar_11_str__ATCC_33695
d__Bacteria.p__Tenericutes.c__Mollicutes.o__Mycoplasmatales.f__Mycoplasmataceae.g__Ureaplasma.s__urealyticum.t__sv_10__ATCC_33699
0.834
d__Bacteria.p__Tenericutes.c__Mollicutes.o__Mycoplasmatales.f__Mycoplasmataceae.g__Ureaplasma.s__urealyticum.t__serovar_2_str__ATCC_27814
d__Bacteria.p__Tenericutes.c__Mollicutes.o__Mycoplasmatales.f__Mycoplasmataceae.g__Ureaplasma.s__urealyticum.t__serovar_13_str__ATCC_33698
0.870
0.687
d__Bacteria.p__Tenericutes.c__Mollicutes.o__Mycoplasmatales.f__Mycoplasmataceae.g__Mycoplasma.s__suis.t__str__Illinois
1.000
1.000
d__Bacteria.p__Tenericutes.c__Mollicutes.o__Mycoplasmatales.f__Mycoplasmataceae.g__Mycoplasma.s__haemofelis.t__str__Langford_1
1.000
d__Bacteria.p__Tenericutes.c__Mollicutes.o__Mycoplasmatales.f__Mycoplasmataceae.g__Mycoplasma.s__haemofelis.t__Ohio2
d__Bacteria.p__Tenericutes.c__Mollicutes.o__Mycoplasmatales.f__Mycoplasmataceae.g__Mycoplasma.s__penetrans.t__HF_2
d__Bacteria.p__Tenericutes.c__Mollicutes.o__Mycoplasmatales.f__Mycoplasmataceae.g__Mycoplasma.s__gallisepticum.t__str__F
0.918
d__Bacteria.p__Tenericutes.c__Mollicutes.o__Mycoplasmatales.f__Mycoplasmataceae.g__Mycoplasma.s__gallisepticum.t__str__R
0.977
d__Bacteria.p__Tenericutes.c__Mollicutes.o__Mycoplasmatales.f__Mycoplasmataceae.g__Mycoplasma.s__gallisepticum.t__str__R_high_
1.000
0.974
d__Bacteria.p__Tenericutes.c__Mollicutes.o__Mycoplasmatales.f__Mycoplasmataceae.g__Mycoplasma.s__genitalium.t__G37
1.000
d__Bacteria.p__Tenericutes.c__Mollicutes.o__Mycoplasmatales.f__Mycoplasmataceae.g__Mycoplasma.s__genitalium.t__G37
0.843
1.000
d__Bacteria.p__Tenericutes.c__Mollicutes.o__Mycoplasmatales.f__Mycoplasmataceae.g__Mycoplasma.s__pneumoniae.t__M129
1.000
d__Bacteria.p__Tenericutes.c__Mollicutes.o__Mycoplasmatales.f__Mycoplasmataceae.g__Mycoplasma.s__pneumoniae.t__FH
d__Bacteria.p__Tenericutes.c__Mollicutes.o__Mycoplasmatales.f__Mycoplasmataceae.g__Mycoplasma.s__hominis.t___1
1.000
d__Bacteria.p__Tenericutes.c__Mollicutes.o__Mycoplasmatales.f__Mycoplasmataceae.g__Mycoplasma.s__arthritidis.t__158L3_1
d__Bacteria.p__Tenericutes.c__Mollicutes.o__Mycoplasmatales.f__Mycoplasmataceae.g__Mycoplasma.s__hyorhinis.t__HUB_1
1.000
d__Bacteria.p__Tenericutes.c__Mollicutes.o__Mycoplasmatales.f__Mycoplasmataceae.g__Mycoplasma.s__conjunctivae.t___1
1.000
1.000
d__Bacteria.p__Tenericutes.c__Mollicutes.o__Mycoplasmatales.f__Mycoplasmataceae.g__Mycoplasma.s__hyopneumoniae.t__7448
d__Bacteria.p__Tenericutes.c__Mollicutes.o__Mycoplasmatales.f__Mycoplasmataceae.g__Mycoplasma.s__hyopneumoniae.t__168
d__Bacteria.p__Tenericutes.c__Mollicutes.o__Mycoplasmatales.f__Mycoplasmataceae.g__Mycoplasma.s__hyopneumoniae.t__232
0.697
d__Bacteria.p__Tenericutes.c__Mollicutes.o__Mycoplasmatales.f__Mycoplasmataceae.g__Mycoplasma.s__hyopneumoniae.t__J
0.624
d__Bacteria.p__Tenericutes.c__Mollicutes.o__Mycoplasmatales.f__Mycoplasmataceae.g__Mycoplasma.s__mobile.t__163K
d__Bacteria.p__Tenericutes.c__Mollicutes.o__Mycoplasmatales.f__Mycoplasmataceae.g__Mycoplasma.s__fermentans.t__JER
1.000
d__Bacteria.p__Tenericutes.c__Mollicutes.o__Mycoplasmatales.f__Mycoplasmataceae.g__Mycoplasma.s__fermentans.t__M64
0.874
d__Bacteria.p__Tenericutes.c__Mollicutes.o__Mycoplasmatales.f__Mycoplasmataceae.g__Mycoplasma.s__fermentans.t__PG18
1.000
d__Bacteria.p__Tenericutes.c__Mollicutes.o__Mycoplasmatales.f__Mycoplasmataceae.g__Mycoplasma.s__bovis.t__PG45
0.626
1.000
d__Bacteria.p__Tenericutes.c__Mollicutes.o__Mycoplasmatales.f__Mycoplasmataceae.g__Mycoplasma.s__bovis.t__Hubei_1
1.000
d__Bacteria.p__Tenericutes.c__Mollicutes.o__Mycoplasmatales.f__Mycoplasmataceae.g__Mycoplasma.s__agalactiae.t___1
1.000
1.000
d__Bacteria.p__Tenericutes.c__Mollicutes.o__Mycoplasmatales.f__Mycoplasmataceae.g__Mycoplasma.s__agalactiae.t__PG2
d__Bacteria.p__Tenericutes.c__Mollicutes.o__Mycoplasmatales.f__Mycoplasmataceae.g__Mycoplasma.s__synoviae.t__53
0.340
0.047
d__Bacteria.p__Tenericutes.c__Mollicutes.o__Mycoplasmatales.f__Mycoplasmataceae.g__Mycoplasma.s__crocodyli.t__MP145
1.000
d__Bacteria.p__Tenericutes.c__Mollicutes.o__Mycoplasmatales.f__Mycoplasmataceae.g__Mycoplasma.s__alligatoris.t__A21JP2
d__Bacteria.p__Tenericutes.c__Mollicutes.o__Mycoplasmatales.f__Mycoplasmataceae.g__Mycoplasma.s__pulmonis.t__UAB_CTIP
d__Bacteria.p__Tenericutes.c__Mollicutes.o__Entomoplasmatales.f__Entomoplasmataceae.g__Mesoplasma.s__florum.t__L1
d__Bacteria.p__Tenericutes.c__Mollicutes.o__Mycoplasmatales.f__Mycoplasmataceae.g__Mycoplasma.s__mycoides.t__subsp__mycoides_SC_str__PG1
1.000
d__Bacteria.p__Tenericutes.c__Mollicutes.o__Mycoplasmatales.f__Mycoplasmataceae.g__Mycoplasma.s__mycoides.t__subsp__mycoides_SC_str__Gladysdale
0.802
1.000
0.961
d__Bacteria.p__Tenericutes.c__Mollicutes.o__Mycoplasmatales.f__Mycoplasmataceae.g__Mycoplasma.s__mycoides.t__subsp__capri_LC_str__95010
d__Bacteria.p__Tenericutes.c__Mollicutes.o__Mycoplasmatales.f__Mycoplasmataceae.g__Mycoplasma.s__mycoides.t__subsp__capri_str__GM12_clone_deltatypeIIIres_
1.000
d__Bacteria.p__Tenericutes.c__Mollicutes.o__Mycoplasmatales.f__Mycoplasmataceae.g__Mycoplasma.s__mycoides.t__subsp__capri_str__GM12_transgenic_clone_tetM_lacZ_
d__Bacteria.p__Tenericutes.c__Mollicutes.o__Mycoplasmatales.f__Mycoplasmataceae.g__Mycoplasma.s__capricolum.t__subsp__capricolum_ATCC_27343
d__Bacteria.p__Tenericutes.c__Mollicutes.o__Mycoplasmatales.f__Mycoplasmataceae.g__Mycoplasma.s__leachii.t__PG50
0.997
d__Bacteria.p__Tenericutes.c__Mollicutes.o__Mycoplasmatales.f__Mycoplasmataceae.g__Mycoplasma.s__leachii.t__99_014_6
d__Bacteria.p__Firmicutes.c__Erysipelotrichi.o__Erysipelotrichales.f__Erysipelotrichaceae.g__unclassified.s__spiroforme.t__DSM_1552
d__Bacteria.p__Firmicutes.c__Erysipelotrichi.o__Erysipelotrichales.f__Erysipelotrichaceae.g__Coprobacillus.s__bacterium.t__D7
1.000
d__Bacteria.p__Firmicutes.c__Erysipelotrichi.o__Erysipelotrichales.f__Erysipelotrichaceae.g__unclassified.s__ramosum.t__DSM_1402
1.000
d__Bacteria.p__Firmicutes.c__Erysipelotrichi.o__Erysipelotrichales.f__Erysipelotrichaceae.g__Coprobacillus.s__sp_.t__29_1
0.668
d__Bacteria.p__Firmicutes.c__Erysipelotrichi.o__Erysipelotrichales.f__Erysipelotrichaceae.g__Catenibacterium.s__mitsuokai.t__DSM_15897
d__Bacteria.p__Firmicutes.c__Erysipelotrichi.o__Erysipelotrichales.f__Erysipelotrichaceae.g__Erysipelothrix.s__rhusiopathiae.t__str__Fujisawa
1.000
1.000
d__Bacteria.p__Firmicutes.c__Erysipelotrichi.o__Erysipelotrichales.f__Erysipelotrichaceae.g__Erysipelothrix.s__rhusiopathiae.t__ATCC_19414
d__Bacteria.p__Firmicutes.c__Erysipelotrichi.o__Erysipelotrichales.f__Erysipelotrichaceae.g__unclassified.s__dolichum.t__DSM_3991
1.000
d__Bacteria.p__Firmicutes.c__Erysipelotrichi.o__Erysipelotrichales.f__Erysipelotrichaceae.g__unclassified.s__bacterium.t__5_2_54FAA
0.997
1.000
d__Bacteria.p__Firmicutes.c__Erysipelotrichi.o__Erysipelotrichales.f__Erysipelotrichaceae.g__Clostridium.s__sp_.t__HGF2
1.000
1.000
d__Bacteria.p__Firmicutes.c__Erysipelotrichi.o__Erysipelotrichales.f__Erysipelotrichaceae.g__unclassified.s__bacterium.t__3_1_53
d__Bacteria.p__Firmicutes.c__Erysipelotrichi.o__Erysipelotrichales.f__Erysipelotrichaceae.g__unclassified.s__cylindroides.t__T2_87
0.965
1.000
d__Bacteria.p__Firmicutes.c__Erysipelotrichi.o__Erysipelotrichales.f__Erysipelotrichaceae.g__unclassified.s__biforme.t__DSM_3989
d__Bacteria.p__Firmicutes.c__Erysipelotrichi.o__Erysipelotrichales.f__Erysipelotrichaceae.g__Holdemania.s__filiformis.t__DSM_12042
1.000
d__Bacteria.p__Firmicutes.c__Erysipelotrichi.o__Erysipelotrichales.f__Erysipelotrichaceae.g__Bulleidia.s__extructa.t__W1219
1.000
d__Bacteria.p__Firmicutes.c__Erysipelotrichi.o__Erysipelotrichales.f__Erysipelotrichaceae.g__Solobacterium.s__moorei.t__F0204
d__Bacteria.p__Actinobacteria.c__Actinobacteria.o__Coriobacteriales.f__Coriobacteriaceae.g__Slackia.s__exigua.t__ATCC_700122
1.000
d__Bacteria.p__Actinobacteria.c__Actinobacteria.o__Coriobacteriales.f__Coriobacteriaceae.g__Slackia.s__heliotrinireducens.t__DSM_20476
1.000
d__Bacteria.p__Actinobacteria.c__Actinobacteria.o__Coriobacteriales.f__Coriobacteriaceae.g__Cryptobacterium.s__curtum.t__12_3__DSM_15641
0.951
d__Bacteria.p__Actinobacteria.c__Actinobacteria.o__Coriobacteriales.f__Coriobacteriaceae.g__Eggerthella.s__sp_.t__YY7918
1.000
d__Bacteria.p__Actinobacteria.c__Actinobacteria.o__Coriobacteriales.f__Coriobacteriaceae.g__Gordonibacter.s__pamelaeae.t__7_10_1_b
d__Bacteria.p__Actinobacteria.c__Actinobacteria.o__Coriobacteriales.f__Coriobacteriaceae.g__Eggerthella.s__sp_.t__HGA1
1.000
d__Bacteria.p__Actinobacteria.c__Actinobacteria.o__Coriobacteriales.f__Coriobacteriaceae.g__Eggerthella.s__lenta.t__VPI_0255__DSM_2243
0.437
d__Bacteria.p__Actinobacteria.c__Actinobacteria.o__Coriobacteriales.f__Coriobacteriaceae.g__Eggerthella.s__sp_.t__1_3_56FAA
d__Bacteria.p__Actinobacteria.c__Actinobacteria.o__Coriobacteriales.f__Coriobacteriaceae.g__Collinsella.s__aerofaciens.t__ATCC_25986
1.000
d__Bacteria.p__Actinobacteria.c__Actinobacteria.o__Coriobacteriales.f__Coriobacteriaceae.g__Collinsella.s__intestinalis.t__DSM_13280
1.000
d__Bacteria.p__Actinobacteria.c__Actinobacteria.o__Coriobacteriales.f__Coriobacteriaceae.g__Collinsella.s__stercoris.t__DSM_13279
1.000
d__Bacteria.p__Actinobacteria.c__Actinobacteria.o__Coriobacteriales.f__Coriobacteriaceae.g__Atopobium.s__parvulum.t__IPP_1246__DSM_20469
1.000
d__Bacteria.p__Actinobacteria.c__Actinobacteria.o__Coriobacteriales.f__Coriobacteriaceae.g__Atopobium.s__rimae.t__ATCC_49626
1.000
d__Bacteria.p__Actinobacteria.c__Actinobacteria.o__Coriobacteriales.f__Coriobacteriaceae.g__Olsenella.s__uli.t__DSM_7084
0.999
d__Bacteria.p__Actinobacteria.c__Actinobacteria.o__Coriobacteriales.f__Coriobacteriaceae.g__Atopobium.s__vaginae.t__PB189_T1_4
1.000
d__Bacteria.p__Actinobacteria.c__Actinobacteria.o__Coriobacteriales.f__Coriobacteriaceae.g__Atopobium.s__vaginae.t__DSM_15829
1.000
d__Bacteria.p__Actinobacteria.c__Actinobacteria.o__Coriobacteriales.f__Coriobacteriaceae.g__Atopobium.s__vaginae.t__DSM_15829
AAA044D11.fna
bin__106_10132015
0.916
bin__127_10132015
bin__184_10132015
0.605
bin__25_10132015
0.987
0.464
bin__75_10142015
bin__6_10142015
bin__241_10132015
0.989
bin__159_10132015
0.685
bin__38_10132015
1.000
0.719
bin__35_10132015
bin__85_10142015
1.000
0.500
bin__19_10132015
bin__1_10132015
bin__110_10132015
0.628
1.000
bin__94_10142015
bin__145_10132015
AAA027J17.fna
1.000
AAA027L06.fna
1.000
AAA028A23.fna
AAA278I18.fna
0.248
AAA023D18.fna
AAA278O22.fna
1.000
AAA027M14.fna
1.000
AAA024D14.fna
AAA041L13.fna
0.658
AAA044N04.fna
0.690
AAA028I14.fna
0.934
AAA023J06.fna

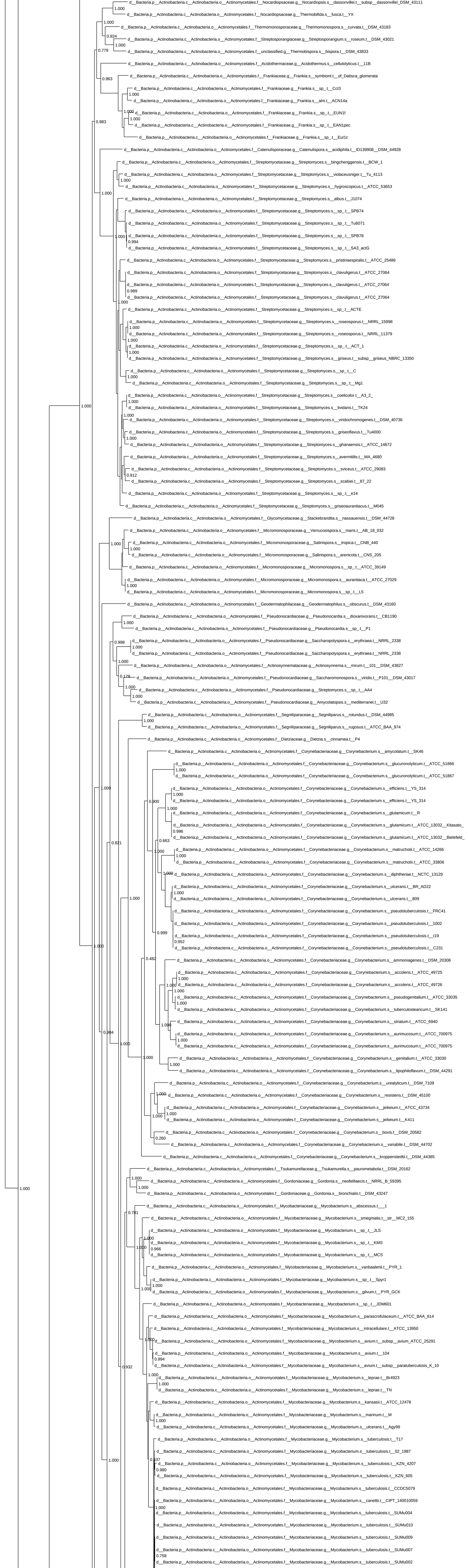
d__Bacteria.p__Actinobacteria.c__Actinobacteria.o__Actinomycetales.f__Nocardiopsaceae.g__Nocardiopsis.s__dassonvillei.t__subsp__dassonvillei_DSM_43111
d__Bacteria.p__Actinobacteria.c__Actinobacteria.o__Actinomycetales.f__Nocardiopsaceae.g__Thermobifida.s__fusca.t__YX
d__Bacteria.p__Actinobacteria.c__Actinobacteria.o__Actinomycetales.f__Thermomonosporaceae.g__Thermomonospora.s__curvata.t__DSM_43183
d__Bacteria.p__Actinobacteria.c__Actinobacteria.o__Actinomycetales.f__Streptosporangiaceae.g__Streptosporangium.s__roseum.t__DSM_43021
d__Bacteria.p__Actinobacteria.c__Actinobacteria.o__Actinomycetales.f__unclassified.g__Thermobispora.s__bispora.t__DSM_43833
d__Bacteria.p__Actinobacteria.c__Actinobacteria.o__Actinomycetales.f__Acidothermaceae.g__Acidothermus.s__cellulolyticus.t__11B
d__Bacteria.p__Actinobacteria.c__Actinobacteria.o__Actinomycetales.f__Frankiaceae.g__Frankia.s__symbiont.t__of_Datisca_glomerata
d__Bacteria.p__Actinobacteria.c__Actinobacteria.o__Actinomycetales.f__Frankiaceae.g__Frankia.s__sp_.t__CcI3
d__Bacteria.p__Actinobacteria.c__Actinobacteria.o__Actinomycetales.f__Frankiaceae.g__Frankia.s__alni.t__ACN14a
d__Bacteria.p__Actinobacteria.c__Actinobacteria.o__Actinomycetales.f__Frankiaceae.g__Frankia.s__sp_.t__EUN1f
d__Bacteria.p__Actinobacteria.c__Actinobacteria.o__Actinomycetales.f__Frankiaceae.g__Frankia.s__sp_.t__EAN1pec
d__Bacteria.p__Actinobacteria.c__Actinobacteria.o__Actinomycetales.f__Frankiaceae.g__Frankia.s__sp_.t__EuI1c
d__Bacteria.p__Actinobacteria.c__Actinobacteria.o__Actinomycetales.f__Catenulisporaceae.g__Catenulispora.s__acidiphila.t__ID139908__DSM_44928
d__Bacteria.p__Actinobacteria.c__Actinobacteria.o__Actinomycetales.f__Streptomycetaceae.g__Streptomyces.s__bingchenggensis.t__BCW_1
d__Bacteria.p__Actinobacteria.c__Actinobacteria.o__Actinomycetales.f__Streptomycetaceae.g__Streptomyces.s__violaceusniger.t__Tu_4113
d__Bacteria.p__Actinobacteria.c__Actinobacteria.o__Actinomycetales.f__Streptomycetaceae.g__Streptomyces.s__hygroscopicus.t__ATCC_53653
d__Bacteria.p__Actinobacteria.c__Actinobacteria.o__Actinomycetales.f__Streptomycetaceae.g__Streptomyces.s__albus.t__J1074
d__Bacteria.p__Actinobacteria.c__Actinobacteria.o__Actinomycetales.f__Streptomycetaceae.g__Streptomyces.s__sp_.t__SPB74
d__Bacteria.p__Actinobacteria.c__Actinobacteria.o__Actinomycetales.f__Streptomycetaceae.g__Streptomyces.s__sp_.t__Tu6071
d__Bacteria.p__Actinobacteria.c__Actinobacteria.o__Actinomycetales.f__Streptomycetaceae.g__Streptomyces.s__sp_.t__SPB78
d__Bacteria.p__Actinobacteria.c__Actinobacteria.o__Actinomycetales.f__Streptomycetaceae.g__Streptomyces.s__sp_.t__SA3_actG
d__Bacteria.p__Actinobacteria.c__Actinobacteria.o__Actinomycetales.f__Streptomycetaceae.g__Streptomyces.s__pristinaespiralis.t__ATCC_25486
d__Bacteria.p__Actinobacteria.c__Actinobacteria.o__Actinomycetales.f__Streptomycetaceae.g__Streptomyces.s__clavuligerus.t__ATCC_27064
d__Bacteria.p__Actinobacteria.c__Actinobacteria.o__Actinomycetales.f__Streptomycetaceae.g__Streptomyces.s__clavuligerus.t__ATCC_27064
d__Bacteria.p__Actinobacteria.c__Actinobacteria.o__Actinomycetales.f__Streptomycetaceae.g__Streptomyces.s__clavuligerus.t__ATCC_27064
d__Bacteria.p__Actinobacteria.c__Actinobacteria.o__Actinomycetales.f__Streptomycetaceae.g__Streptomyces.s__sp_.t__ACTE
d__Bacteria.p__Actinobacteria.c__Actinobacteria.o__Actinomycetales.f__Streptomycetaceae.g__Streptomyces.s__roseosporus.t__NRRL_15998
d__Bacteria.p__Actinobacteria.c__Actinobacteria.o__Actinomycetales.f__Streptomycetaceae.g__Streptomyces.s__roseosporus.t__NRRL_11379
d__Bacteria.p__Actinobacteria.c__Actinobacteria.o__Actinomycetales.f__Streptomycetaceae.g__Streptomyces.s__sp_.t__ACT_1
d__Bacteria.p__Actinobacteria.c__Actinobacteria.o__Actinomycetales.f__Streptomycetaceae.g__Streptomyces.s__griseus.t__subsp__griseus_NBRC_13350
d__Bacteria.p__Actinobacteria.c__Actinobacteria.o__Actinomycetales.f__Streptomycetaceae.g__Streptomyces.s__sp_.t__C
d__Bacteria.p__Actinobacteria.c__Actinobacteria.o__Actinomycetales.f__Streptomycetaceae.g__Streptomyces.s__sp_.t__Mg1
d__Bacteria.p__Actinobacteria.c__Actinobacteria.o__Actinomycetales.f__Streptomycetaceae.g__Streptomyces.s__coelicolor.t__A3_2_
d__Bacteria.p__Actinobacteria.c__Actinobacteria.o__Actinomycetales.f__Streptomycetaceae.g__Streptomyces.s__lividans.t__TK24
d__Bacteria.p__Actinobacteria.c__Actinobacteria.o__Actinomycetales.f__Streptomycetaceae.g__Streptomyces.s__viridochromogenes.t__DSM_40736
d__Bacteria.p__Actinobacteria.c__Actinobacteria.o__Actinomycetales.f__Streptomycetaceae.g__Streptomyces.s__griseoflavus.t__Tu4000
d__Bacteria.p__Actinobacteria.c__Actinobacteria.o__Actinomycetales.f__Streptomycetaceae.g__Streptomyces.s__ghanaensis.t__ATCC_14672
d__Bacteria.p__Actinobacteria.c__Actinobacteria.o__Actinomycetales.f__Streptomycetaceae.g__Streptomyces.s__avermitilis.t__MA_4680
d__Bacteria.p__Actinobacteria.c__Actinobacteria.o__Actinomycetales.f__Streptomycetaceae.g__Streptomyces.s__sviceus.t__ATCC_29083
d__Bacteria.p__Actinobacteria.c__Actinobacteria.o__Actinomycetales.f__Streptomycetaceae.g__Streptomyces.s__scabiei.t__87_22
d__Bacteria.p__Actinobacteria.c__Actinobacteria.o__Actinomycetales.f__Streptomycetaceae.g__Streptomyces.s__sp_.t__e14
d__Bacteria.p__Actinobacteria.c__Actinobacteria.o__Actinomycetales.f__Streptomycetaceae.g__Streptomyces.s__griseoaurantiacus.t__M045
d__Bacteria.p__Actinobacteria.c__Actinobacteria.o__Actinomycetales.f__Glycomycetaceae.g__Stackebrandtia.s__nassauensis.t__DSM_44728
d__Bacteria.p__Actinobacteria.c__Actinobacteria.o__Actinomycetales.f__Micromonosporaceae.g__Verrucosispora.s__maris.t__AB_18_032
d__Bacteria.p__Actinobacteria.c__Actinobacteria.o__Actinomycetales.f__Micromonosporaceae.g__Salinispora.s__tropica.t__CNB_440
d__Bacteria.p__Actinobacteria.c__Actinobacteria.o__Actinomycetales.f__Micromonosporaceae.g__Salinispora.s__arenicola.t__CNS_205
d__Bacteria.p__Actinobacteria.c__Actinobacteria.o__Actinomycetales.f__Micromonosporaceae.g__Micromonospora.s__sp_.t__ATCC_39149
d__Bacteria.p__Actinobacteria.c__Actinobacteria.o__Actinomycetales.f__Micromonosporaceae.g__Micromonospora.s__aurantiaca.t__ATCC_27029
d__Bacteria.p__Actinobacteria.c__Actinobacteria.o__Actinomycetales.f__Micromonosporaceae.g__Micromonospora.s__sp_.t__L5
d__Bacteria.p__Actinobacteria.c__Actinobacteria.o__Actinomycetales.f__Geodermatophilaceae.g__Geodermatophilus.s__obscurus.t__DSM_43160
d__Bacteria.p__Actinobacteria.c__Actinobacteria.o__Actinomycetales.f__Pseudonocardiaceae.g__Pseudonocardia.s__dioxanivorans.t__CB1190
d__Bacteria.p__Actinobacteria.c__Actinobacteria.o__Actinomycetales.f__Pseudonocardiaceae.g__Pseudonocardia.s__sp_.t__P1
d__Bacteria.p__Actinobacteria.c__Actinobacteria.o__Actinomycetales.f__Pseudonocardiaceae.g__Saccharopolyspora.s__erythraea.t__NRRL_2338
d__Bacteria.p__Actinobacteria.c__Actinobacteria.o__Actinomycetales.f__Pseudonocardiaceae.g__Saccharopolyspora.s__erythraea.t__NRRL_2338
d__Bacteria.p__Actinobacteria.c__Actinobacteria.o__Actinomycetales.f__Actinosynnemataceae.g__Actinosynnema.s__mirum.t__101__DSM_43827
d__Bacteria.p__Actinobacteria.c__Actinobacteria.o__Actinomycetales.f__Pseudonocardiaceae.g__Saccharomonospora.s__viridis.t__P101__DSM_43017
d__Bacteria.p__Actinobacteria.c__Actinobacteria.o__Actinomycetales.f__Pseudonocardiaceae.g__Streptomyces.s__sp_.t__AA4
d__Bacteria.p__Actinobacteria.c__Actinobacteria.o__Actinomycetales.f__Pseudonocardiaceae.g__Amycolatopsis.s__mediterranei.t__U32
d__Bacteria.p__Actinobacteria.c__Actinobacteria.o__Actinomycetales.f__Segniliparaceae.g__Segniliparus.s__rotundus.t__DSM_44985
d__Bacteria.p__Actinobacteria.c__Actinobacteria.o__Actinomycetales.f__Segniliparaceae.g__Segniliparus.s__rugosus.t__ATCC_BAA_974
d__Bacteria.p__Actinobacteria.c__Actinobacteria.o__Actinomycetales.f__Dietziaceae.g__Dietzia.s__cinnamea.t__P4
d__Bacteria.p__Actinobacteria.c__Actinobacteria.o__Actinomycetales.f__Corynebacteriaceae.g__Corynebacterium.s__amycolatum.t__SK46
d__Bacteria.p__Actinobacteria.c__Actinobacteria.o__Actinomycetales.f__Corynebacteriaceae.g__Corynebacterium.s__glucuronolyticum.t__ATCC_51866
d__Bacteria.p__Actinobacteria.c__Actinobacteria.o__Actinomycetales.f__Corynebacteriaceae.g__Corynebacterium.s__glucuronolyticum.t__ATCC_51867
d__Bacteria.p__Actinobacteria.c__Actinobacteria.o__Actinomycetales.f__Corynebacteriaceae.g__Corynebacterium.s__efficiens.t__YS_314
d__Bacteria.p__Actinobacteria.c__Actinobacteria.o__Actinomycetales.f__Corynebacteriaceae.g__Corynebacterium.s__efficiens.t__YS_314
d__Bacteria.p__Actinobacteria.c__Actinobacteria.o__Actinomycetales.f__Corynebacteriaceae.g__Corynebacterium.s__glutamicum.t__R
d__Bacteria.p__Actinobacteria.c__Actinobacteria.o__Actinomycetales.f__Corynebacteriaceae.g__Corynebacterium.s__glutamicum.t__ATCC_13032__Kitasato_
d__Bacteria.p__Actinobacteria.c__Actinobacteria.o__Actinomycetales.f__Corynebacteriaceae.g__Corynebacterium.s__glutamicum.t__ATCC_13032__Bielefeld_
d__Bacteria.p__Actinobacteria.c__Actinobacteria.o__Actinomycetales.f__Corynebacteriaceae.g__Corynebacterium.s__matruchotii.t__ATCC_14266
d__Bacteria.p__Actinobacteria.c__Actinobacteria.o__Actinomycetales.f__Corynebacteriaceae.g__Corynebacterium.s__matruchotii.t__ATCC_33806
d__Bacteria.p__Actinobacteria.c__Actinobacteria.o__Actinomycetales.f__Corynebacteriaceae.g__Corynebacterium.s__diphtheriae.t__NCTC_13129
d__Bacteria.p__Actinobacteria.c__Actinobacteria.o__Actinomycetales.f__Corynebacteriaceae.g__Corynebacterium.s__ulcerans.t__BR_AD22
d__Bacteria.p__Actinobacteria.c__Actinobacteria.o__Actinomycetales.f__Corynebacteriaceae.g__Corynebacterium.s__ulcerans.t__809
d__Bacteria.p__Actinobacteria.c__Actinobacteria.o__Actinomycetales.f__Corynebacteriaceae.g__Corynebacterium.s__pseudotuberculosis.t__FRC41
d__Bacteria.p__Actinobacteria.c__Actinobacteria.o__Actinomycetales.f__Corynebacteriaceae.g__Corynebacterium.s__pseudotuberculosis.t__1002
d__Bacteria.p__Actinobacteria.c__Actinobacteria.o__Actinomycetales.f__Corynebacteriaceae.g__Corynebacterium.s__pseudotuberculosis.t__I19
d__Bacteria.p__Actinobacteria.c__Actinobacteria.o__Actinomycetales.f__Corynebacteriaceae.g__Corynebacterium.s__pseudotuberculosis.t__C231
d__Bacteria.p__Actinobacteria.c__Actinobacteria.o__Actinomycetales.f__Corynebacteriaceae.g__Corynebacterium.s__ammoniagenes.t__DSM_20306
d__Bacteria.p__Actinobacteria.c__Actinobacteria.o__Actinomycetales.f__Corynebacteriaceae.g__Corynebacterium.s__accolens.t__ATCC_49725
d__Bacteria.p__Actinobacteria.c__Actinobacteria.o__Actinomycetales.f__Corynebacteriaceae.g__Corynebacterium.s__accolens.t__ATCC_49726
d__Bacteria.p__Actinobacteria.c__Actinobacteria.o__Actinomycetales.f__Corynebacteriaceae.g__Corynebacterium.s__pseudogenitalium.t__ATCC_33035
d__Bacteria.p__Actinobacteria.c__Actinobacteria.o__Actinomycetales.f__Corynebacteriaceae.g__Corynebacterium.s__tuberculostearicum.t__SK141
d__Bacteria.p__Actinobacteria.c__Actinobacteria.o__Actinomycetales.f__Corynebacteriaceae.g__Corynebacterium.s__striatum.t__ATCC_6940
d__Bacteria.p__Actinobacteria.c__Actinobacteria.o__Actinomycetales.f__Corynebacteriaceae.g__Corynebacterium.s__aurimucosum.t__ATCC_700975
d__Bacteria.p__Actinobacteria.c__Actinobacteria.o__Actinomycetales.f__Corynebacteriaceae.g__Corynebacterium.s__aurimucosum.t__ATCC_700975
d__Bacteria.p__Actinobacteria.c__Actinobacteria.o__Actinomycetales.f__Corynebacteriaceae.g__Corynebacterium.s__genitalium.t__ATCC_33030
d__Bacteria.p__Actinobacteria.c__Actinobacteria.o__Actinomycetales.f__Corynebacteriaceae.g__Corynebacterium.s__lipophiloflavum.t__DSM_44291
d__Bacteria.p__Actinobacteria.c__Actinobacteria.o__Actinomycetales.f__Corynebacteriaceae.g__Corynebacterium.s__urealyticum.t__DSM_7109
d__Bacteria.p__Actinobacteria.c__Actinobacteria.o__Actinomycetales.f__Corynebacteriaceae.g__Corynebacterium.s__resistens.t__DSM_45100
d__Bacteria.p__Actinobacteria.c__Actinobacteria.o__Actinomycetales.f__Corynebacteriaceae.g__Corynebacterium.s__jeikeium.t__ATCC_43734
d__Bacteria.p__Actinobacteria.c__Actinobacteria.o__Actinomycetales.f__Corynebacteriaceae.g__Corynebacterium.s__jeikeium.t__K411
d__Bacteria.p__Actinobacteria.c__Actinobacteria.o__Actinomycetales.f__Corynebacteriaceae.g__Corynebacterium.s__bovis.t__DSM_20582
d__Bacteria.p__Actinobacteria.c__Actinobacteria.o__Actinomycetales.f__Corynebacteriaceae.g__Corynebacterium.s__variabile.t__DSM_44702
d__Bacteria.p__Actinobacteria.c__Actinobacteria.o__Actinomycetales.f__Corynebacteriaceae.g__Corynebacterium.s__kroppenstedtii.t__DSM_44385
d__Bacteria.p__Actinobacteria.c__Actinobacteria.o__Actinomycetales.f__Tsukamurellaceae.g__Tsukamurella.s__paurometabola.t__DSM_20162
d__Bacteria.p__Actinobacteria.c__Actinobacteria.o__Actinomycetales.f__Gordoniaceae.g__Gordonia.s__neofelifaecis.t__NRRL_B_59395
d__Bacteria.p__Actinobacteria.c__Actinobacteria.o__Actinomycetales.f__Gordoniaceae.g__Gordonia.s__bronchialis.t__DSM_43247
d__Bacteria.p__Actinobacteria.c__Actinobacteria.o__Actinomycetales.f__Mycobacteriaceae.g__Mycobacterium.s__abscessus.t__1
d__Bacteria.p__Actinobacteria.c__Actinobacteria.o__Actinomycetales.f__Mycobacteriaceae.g__Mycobacterium.s__smegmatis.t__str__MC2_155
d__Bacteria.p__Actinobacteria.c__Actinobacteria.o__Actinomycetales.f__Mycobacteriaceae.g__Mycobacterium.s__sp_.t__JLS
d__Bacteria.p__Actinobacteria.c__Actinobacteria.o__Actinomycetales.f__Mycobacteriaceae.g__Mycobacterium.s__sp_.t__KMS
d__Bacteria.p__Actinobacteria.c__Actinobacteria.o__Actinomycetales.f__Mycobacteriaceae.g__Mycobacterium.s__sp_.t__MCS
d__Bacteria.p__Actinobacteria.c__Actinobacteria.o__Actinomycetales.f__Mycobacteriaceae.g__Mycobacterium.s__vanbaalenii.t__PYR_1
d__Bacteria.p__Actinobacteria.c__Actinobacteria.o__Actinomycetales.f__Mycobacteriaceae.g__Mycobacterium.s__sp_.t__Spyr1
d__Bacteria.p__Actinobacteria.c__Actinobacteria.o__Actinomycetales.f__Mycobacteriaceae.g__Mycobacterium.s__gilvum.t__PYR_GCK
d__Bacteria.p__Actinobacteria.c__Actinobacteria.o__Actinomycetales.f__Mycobacteriaceae.g__Mycobacterium.s__sp_.t__JDM601
d__Bacteria.p__Actinobacteria.c__Actinobacteria.o__Actinomycetales.f__Mycobacteriaceae.g__Mycobacterium.s__parascrofulaceum.t__ATCC_BAA_614
d__Bacteria.p__Actinobacteria.c__Actinobacteria.o__Actinomycetales.f__Mycobacteriaceae.g__Mycobacterium.s__intracellulare.t__ATCC_13950
d__Bacteria.p__Actinobacteria.c__Actinobacteria.o__Actinomycetales.f__Mycobacteriaceae.g__Mycobacterium.s__avium.t__subsp__avium_ATCC_25291
d__Bacteria.p__Actinobacteria.c__Actinobacteria.o__Actinomycetales.f__Mycobacteriaceae.g__Mycobacterium.s__avium.t__104
d__Bacteria.p__Actinobacteria.c__Actinobacteria.o__Actinomycetales.f__Mycobacteriaceae.g__Mycobacterium.s__avium.t__subsp__paratuberculosis_K_10
d__Bacteria.p__Actinobacteria.c__Actinobacteria.o__Actinomycetales.f__Mycobacteriaceae.g__Mycobacterium.s__leprae.t__Br4923
d__Bacteria.p__Actinobacteria.c__Actinobacteria.o__Actinomycetales.f__Mycobacteriaceae.g__Mycobacterium.s__leprae.t__TN
d__Bacteria.p__Actinobacteria.c__Actinobacteria.o__Actinomycetales.f__Mycobacteriaceae.g__Mycobacterium.s__kansasii.t__ATCC_12478
d__Bacteria.p__Actinobacteria.c__Actinobacteria.o__Actinomycetales.f__Mycobacteriaceae.g__Mycobacterium.s__marinum.t__M
d__Bacteria.p__Actinobacteria.c__Actinobacteria.o__Actinomycetales.f__Mycobacteriaceae.g__Mycobacterium.s__ulcerans.t__Agy99
d__Bacteria.p__Actinobacteria.c__Actinobacteria.o__Actinomycetales.f__Mycobacteriaceae.g__Mycobacterium.s__tuberculosis.t__T17
d__Bacteria.p__Actinobacteria.c__Actinobacteria.o__Actinomycetales.f__Mycobacteriaceae.g__Mycobacterium.s__tuberculosis.t__02_1987
d__Bacteria.p__Actinobacteria.c__Actinobacteria.o__Actinomycetales.f__Mycobacteriaceae.g__Mycobacterium.s__tuberculosis.t__KZN_4207
d__Bacteria.p__Actinobacteria.c__Actinobacteria.o__Actinomycetales.f__Mycobacteriaceae.g__Mycobacterium.s__tuberculosis.t__KZN_605
d__Bacteria.p__Actinobacteria.c__Actinobacteria.o__Actinomycetales.f__Mycobacteriaceae.g__Mycobacterium.s__tuberculosis.t__CCDC5079
d__Bacteria.p__Actinobacteria.c__Actinobacteria.o__Actinomycetales.f__Mycobacteriaceae.g__Mycobacterium.s__canettii.t__CIPT_140010059
d__Bacteria.p__Actinobacteria.c__Actinobacteria.o__Actinomycetales.f__Mycobacteriaceae.g__Mycobacterium.s__tuberculosis.t__SUMu004
d__Bacteria.p__Actinobacteria.c__Actinobacteria.o__Actinomycetales.f__Mycobacteriaceae.g__Mycobacterium.s__tuberculosis.t__SUMu010
d__Bacteria.p__Actinobacteria.c__Actinobacteria.o__Actinomycetales.f__Mycobacteriaceae.g__Mycobacterium.s__tuberculosis.t__SUMu009
d__Bacteria.p__Actinobacteria.c__Actinobacteria.o__Actinomycetales.f__Mycobacteriaceae.g__Mycobacterium.s__tuberculosis.t__SUMu007
d__Bacteria.p__Actinobacteria.c__Actinobacteria.o__Actinomycetales.f__Mycobacteriaceae.g__Mycobacterium.s__tuberculosis.t__SUMu002

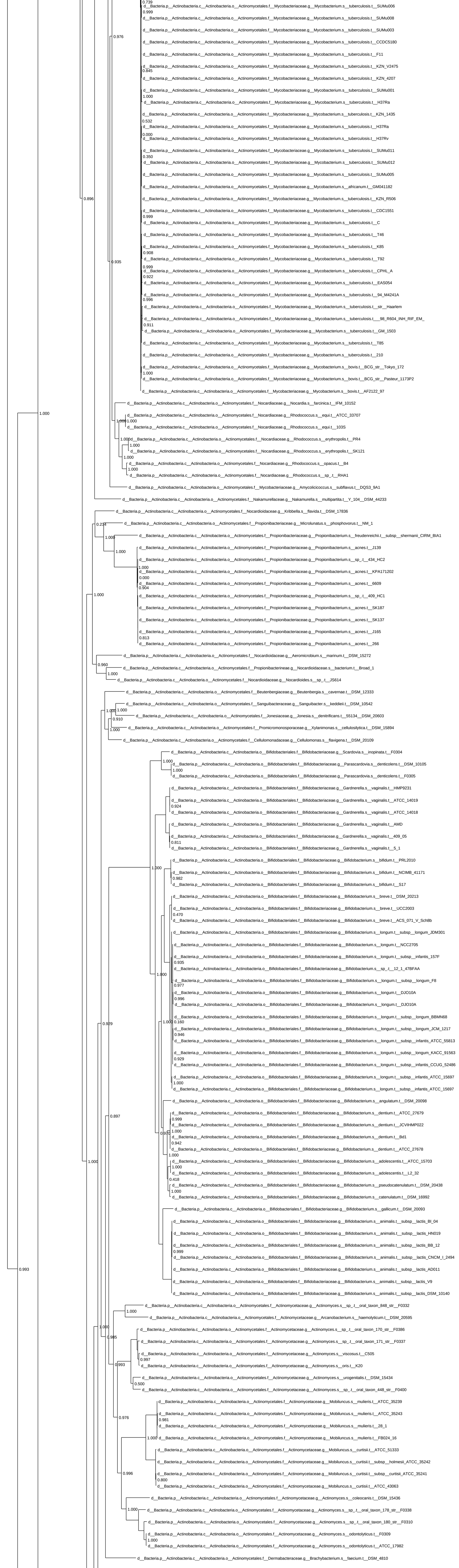

0.739
d__Bacteria.p__Actinobacteria.c__Actinobacteria.o__Actinomycetales.f__Mycobacteriaceae.g__Mycobacterium.s__tuberculosis.t__SUMu006
0.999
d__Bacteria.p__Actinobacteria.c__Actinobacteria.o__Actinomycetales.f__Mycobacteriaceae.g__Mycobacterium.s__tuberculosis.t__SUMu008
0.976
d__Bacteria.p__Actinobacteria.c__Actinobacteria.o__Actinomycetales.f__Mycobacteriaceae.g__Mycobacterium.s__tuberculosis.t__SUMu003
d__Bacteria.p__Actinobacteria.c__Actinobacteria.o__Actinomycetales.f__Mycobacteriaceae.g__Mycobacterium.s__tuberculosis.t__CCDC5180
d__Bacteria.p__Actinobacteria.c__Actinobacteria.o__Actinomycetales.f__Mycobacteriaceae.g__Mycobacterium.s__tuberculosis.t__F11
d__Bacteria.p__Actinobacteria.c__Actinobacteria.o__Actinomycetales.f__Mycobacteriaceae.g__Mycobacterium.s__tuberculosis.t__KZN_V2475
0.845
d__Bacteria.p__Actinobacteria.c__Actinobacteria.o__Actinomycetales.f__Mycobacteriaceae.g__Mycobacterium.s__tuberculosis.t__KZN_4207
d__Bacteria.p__Actinobacteria.c__Actinobacteria.o__Actinomycetales.f__Mycobacteriaceae.g__Mycobacterium.s__tuberculosis.t__SUMu001
1.000
d__Bacteria.p__Actinobacteria.c__Actinobacteria.o__Actinomycetales.f__Mycobacteriaceae.g__Mycobacterium.s__tuberculosis.t__H37Ra
d__Bacteria.p__Actinobacteria.c__Actinobacteria.o__Actinomycetales.f__Mycobacteriaceae.g__Mycobacterium.s__tuberculosis.t__KZN_1435
0.532
d__Bacteria.p__Actinobacteria.c__Actinobacteria.o__Actinomycetales.f__Mycobacteriaceae.g__Mycobacterium.s__tuberculosis.t__H37Ra
0.000
d__Bacteria.p__Actinobacteria.c__Actinobacteria.o__Actinomycetales.f__Mycobacteriaceae.g__Mycobacterium.s__tuberculosis.t__H37Rv
d__Bacteria.p__Actinobacteria.c__Actinobacteria.o__Actinomycetales.f__Mycobacteriaceae.g__Mycobacterium.s__tuberculosis.t__SUMu011
0.350
d__Bacteria.p__Actinobacteria.c__Actinobacteria.o__Actinomycetales.f__Mycobacteriaceae.g__Mycobacterium.s__tuberculosis.t__SUMu012
d__Bacteria.p__Actinobacteria.c__Actinobacteria.o__Actinomycetales.f__Mycobacteriaceae.g__Mycobacterium.s__tuberculosis.t__SUMu005
d__Bacteria.p__Actinobacteria.c__Actinobacteria.o__Actinomycetales.f__Mycobacteriaceae.g__Mycobacterium.s__africanum.t__GM041182
0.896
d__Bacteria.p__Actinobacteria.c__Actinobacteria.o__Actinomycetales.f__Mycobacteriaceae.g__Mycobacterium.s__tuberculosis.t__KZN_R506
d__Bacteria.p__Actinobacteria.c__Actinobacteria.o__Actinomycetales.f__Mycobacteriaceae.g__Mycobacterium.s__tuberculosis.t__CDC1551
0.999
d__Bacteria.p__Actinobacteria.c__Actinobacteria.o__Actinomycetales.f__Mycobacteriaceae.g__Mycobacterium.s__tuberculosis.t__C
d__Bacteria.p__Actinobacteria.c__Actinobacteria.o__Actinomycetales.f__Mycobacteriaceae.g__Mycobacterium.s__tuberculosis.t__T46
d__Bacteria.p__Actinobacteria.c__Actinobacteria.o__Actinomycetales.f__Mycobacteriaceae.g__Mycobacterium.s__tuberculosis.t__K85
0.908
0.935
d__Bacteria.p__Actinobacteria.c__Actinobacteria.o__Actinomycetales.f__Mycobacteriaceae.g__Mycobacterium.s__tuberculosis.t__T92
0.999
d__Bacteria.p__Actinobacteria.c__Actinobacteria.o__Actinomycetales.f__Mycobacteriaceae.g__Mycobacterium.s__tuberculosis.t__CPHL_A
0.922
d__Bacteria.p__Actinobacteria.c__Actinobacteria.o__Actinomycetales.f__Mycobacteriaceae.g__Mycobacterium.s__tuberculosis.t__EAS054
d__Bacteria.p__Actinobacteria.c__Actinobacteria.o__Actinomycetales.f__Mycobacteriaceae.g__Mycobacterium.s__tuberculosis.t__94_M4241A
0.996
d__Bacteria.p__Actinobacteria.c__Actinobacteria.o__Actinomycetales.f__Mycobacteriaceae.g__Mycobacterium.s__tuberculosis.t__str__Haarlem
d__Bacteria.p__Actinobacteria.c__Actinobacteria.o__Actinomycetales.f__Mycobacteriaceae.g__Mycobacterium.s__tuberculosis.t___98_R604_INH_RIF_EM_
0.911
d__Bacteria.p__Actinobacteria.c__Actinobacteria.o__Actinomycetales.f__Mycobacteriaceae.g__Mycobacterium.s__tuberculosis.t__GM_1503
d__Bacteria.p__Actinobacteria.c__Actinobacteria.o__Actinomycetales.f__Mycobacteriaceae.g__Mycobacterium.s__tuberculosis.t__T85
d__Bacteria.p__Actinobacteria.c__Actinobacteria.o__Actinomycetales.f__Mycobacteriaceae.g__Mycobacterium.s__tuberculosis.t__210
d__Bacteria.p__Actinobacteria.c__Actinobacteria.o__Actinomycetales.f__Mycobacteriaceae.g__Mycobacterium.s__bovis.t__BCG_str__Tokyo_172
1.000
d__Bacteria.p__Actinobacteria.c__Actinobacteria.o__Actinomycetales.f__Mycobacteriaceae.g__Mycobacterium.s__bovis.t__BCG_str__Pasteur_1173P2
d__Bacteria.p__Actinobacteria.c__Actinobacteria.o__Actinomycetales.f__Mycobacteriaceae.g__Mycobacterium.s__bovis.t__AF2122_97
d__Bacteria.p__Actinobacteria.c__Actinobacteria.o__Actinomycetales.f__Nocardiaceae.g__Nocardia.s__farcinica.t__IFM_10152
d__Bacteria.p__Actinobacteria.c__Actinobacteria.o__Actinomycetales.f__Nocardiaceae.g__Rhodococcus.s__equi.t__ATCC_33707
1.000 1.000
d__Bacteria.p__Actinobacteria.c__Actinobacteria.o__Actinomycetales.f__Nocardiaceae.g__Rhodococcus.s__equi.t__103S
1.000
d__Bacteria.p__Actinobacteria.c__Actinobacteria.o__Actinomycetales.f__Nocardiaceae.g__Rhodococcus.s__erythropolis.t__PR4
1.000
d__Bacteria.p__Actinobacteria.c__Actinobacteria.o__Actinomycetales.f__Nocardiaceae.g__Rhodococcus.s__erythropolis.t__SK121
1.000
1.000
d__Bacteria.p__Actinobacteria.c__Actinobacteria.o__Actinomycetales.f__Nocardiaceae.g__Rhodococcus.s__opacus.t__B4
1.000
d__Bacteria.p__Actinobacteria.c__Actinobacteria.o__Actinomycetales.f__Nocardiaceae.g__Rhodococcus.s__sp_.t__RHA1
d__Bacteria.p__Actinobacteria.c__Actinobacteria.o__Actinomycetales.f__Mycobacteriaceae.g__Amycolicicoccus.s__subflavus.t__DQS3_9A1
d__Bacteria.p__Actinobacteria.c__Actinobacteria.o__Actinomycetales.f__Nakamurellaceae.g__Nakamurella.s__multipartita.t__Y_104__DSM_44233
d__Bacteria.p__Actinobacteria.c__Actinobacteria.o__Actinomycetales.f__Nocardioidaceae.g__Kribbella.s__flavida.t__DSM_17836
0.234
d__Bacteria.p__Actinobacteria.c__Actinobacteria.o__Actinomycetales.f__Propionibacteriaceae.g__Microlunatus.s__phosphovorus.t__NM_1
1.000
d__Bacteria.p__Actinobacteria.c__Actinobacteria.o__Actinomycetales.f__Propionibacteriaceae.g__Propionibacterium.s__freudenreichii.t__subsp__shermanii_CIRM_BIA1
1.000
d__Bacteria.p__Actinobacteria.c__Actinobacteria.o__Actinomycetales.f__Propionibacteriaceae.g__Propionibacterium.s__acnes.t__J139
d__Bacteria.p__Actinobacteria.c__Actinobacteria.o__Actinomycetales.f__Propionibacteriaceae.g__Propionibacterium.s__sp_.t__434_HC2
1.000
d__Bacteria.p__Actinobacteria.c__Actinobacteria.o__Actinomycetales.f__Propionibacteriaceae.g__Propionibacterium.s__acnes.t__KPA171202
0.000
d__Bacteria.p__Actinobacteria.c__Actinobacteria.o__Actinomycetales.f__Propionibacteriaceae.g__Propionibacterium.s__acnes.t__6609
0.904
1.000
d__Bacteria.p__Actinobacteria.c__Actinobacteria.o__Actinomycetales.f__Propionibacteriaceae.g__Propionibacterium.s__sp_.t__409_HC1
d__Bacteria.p__Actinobacteria.c__Actinobacteria.o__Actinomycetales.f__Propionibacteriaceae.g__Propionibacterium.s__acnes.t__SK187
d__Bacteria.p__Actinobacteria.c__Actinobacteria.o__Actinomycetales.f__Propionibacteriaceae.g__Propionibacterium.s__acnes.t__SK137
d__Bacteria.p__Actinobacteria.c__Actinobacteria.o__Actinomycetales.f__Propionibacteriaceae.g__Propionibacterium.s__acnes.t__J165
0.813
d__Bacteria.p__Actinobacteria.c__Actinobacteria.o__Actinomycetales.f__Propionibacteriaceae.g__Propionibacterium.s__acnes.t__266
d__Bacteria.p__Actinobacteria.c__Actinobacteria.o__Actinomycetales.f__Nocardioidaceae.g__Aeromicrobium.s__marinum.t__DSM_15272
0.960
d__Bacteria.p__Actinobacteria.c__Actinobacteria.o__Actinomycetales.f__Propionibacterineae.g__Nocardioidaceae.s__bacterium.t__Broad_1
1.000
d__Bacteria.p__Actinobacteria.c__Actinobacteria.o__Actinomycetales.f__Nocardioidaceae.g__Nocardioides.s__sp_.t__JS614
d__Bacteria.p__Actinobacteria.c__Actinobacteria.o__Actinomycetales.f__Beutenbergiaceae.g__Beutenbergia.s__cavernae.t__DSM_12333
d__Bacteria.p__Actinobacteria.c__Actinobacteria.o__Actinomycetales.f__Sanguibacteraceae.g__Sanguibacter.s__keddieii.t__DSM_10542
1.000 1.000
d__Bacteria.p__Actinobacteria.c__Actinobacteria.o__Actinomycetales.f__Jonesiaceae.g__Jonesia.s__denitrificans.t__55134__DSM_20603
0.910
d__Bacteria.p__Actinobacteria.c__Actinobacteria.o__Actinomycetales.f__Promicromonosporaceae.g__Xylanimonas.s__cellulosilytica.t__DSM_15894
1.000
d__Bacteria.p__Actinobacteria.c__Actinobacteria.o__Actinomycetales.f__Cellulomonadaceae.g__Cellulomonas.s__flavigena.t__DSM_20109
d__Bacteria.p__Actinobacteria.c__Actinobacteria.o__Bifidobacteriales.f__Bifidobacteriaceae.g__Scardovia.s__inopinata.t__F0304
1.000
d__Bacteria.p__Actinobacteria.c__Actinobacteria.o__Bifidobacteriales.f__Bifidobacteriaceae.g__Parascardovia.s__denticolens.t__DSM_10105
1.000
d__Bacteria.p__Actinobacteria.c__Actinobacteria.o__Bifidobacteriales.f__Bifidobacteriaceae.g__Parascardovia.s__denticolens.t__F0305
d__Bacteria.p__Actinobacteria.c__Actinobacteria.o__Bifidobacteriales.f__Bifidobacteriaceae.g__Gardnerella.s__vaginalis.t__HMP9231
d__Bacteria.p__Actinobacteria.c__Actinobacteria.o__Bifidobacteriales.f__Bifidobacteriaceae.g__Gardnerella.s__vaginalis.t__ATCC_14019
0.924
d__Bacteria.p__Actinobacteria.c__Actinobacteria.o__Bifidobacteriales.f__Bifidobacteriaceae.g__Gardnerella.s__vaginalis.t__ATCC_14018
d__Bacteria.p__Actinobacteria.c__Actinobacteria.o__Bifidobacteriales.f__Bifidobacteriaceae.g__Gardnerella.s__vaginalis.t__AMD
d__Bacteria.p__Actinobacteria.c__Actinobacteria.o__Bifidobacteriales.f__Bifidobacteriaceae.g__Gardnerella.s__vaginalis.t__409_05
0.811
d__Bacteria.p__Actinobacteria.c__Actinobacteria.o__Bifidobacteriales.f__Bifidobacteriaceae.g__Gardnerella.s__vaginalis.t__5_1
d__Bacteria.p__Actinobacteria.c__Actinobacteria.o__Bifidobacteriales.f__Bifidobacteriaceae.g__Bifidobacterium.s__bifidum.t__PRL2010
1.000
d__Bacteria.p__Actinobacteria.c__Actinobacteria.o__Bifidobacteriales.f__Bifidobacteriaceae.g__Bifidobacterium.s__bifidum.t__NCIMB_41171
0.982
d__Bacteria.p__Actinobacteria.c__Actinobacteria.o__Bifidobacteriales.f__Bifidobacteriaceae.g__Bifidobacterium.s__bifidum.t__S17
d__Bacteria.p__Actinobacteria.c__Actinobacteria.o__Bifidobacteriales.f__Bifidobacteriaceae.g__Bifidobacterium.s__breve.t__DSM_20213
d__Bacteria.p__Actinobacteria.c__Actinobacteria.o__Bifidobacteriales.f__Bifidobacteriaceae.g__Bifidobacterium.s__breve.t__UCC2003
0.470
d__Bacteria.p__Actinobacteria.c__Actinobacteria.o__Bifidobacteriales.f__Bifidobacteriaceae.g__Bifidobacterium.s__breve.t__ACS_071_V_Sch8b
d__Bacteria.p__Actinobacteria.c__Actinobacteria.o__Bifidobacteriales.f__Bifidobacteriaceae.g__Bifidobacterium.s__longum.t__subsp__longum_JDM301
d__Bacteria.p__Actinobacteria.c__Actinobacteria.o__Bifidobacteriales.f__Bifidobacteriaceae.g__Bifidobacterium.s__longum.t__NCC2705
d__Bacteria.p__Actinobacteria.c__Actinobacteria.o__Bifidobacteriales.f__Bifidobacteriaceae.g__Bifidobacterium.s__longum.t__subsp__infantis_157F
0.935
d__Bacteria.p__Actinobacteria.c__Actinobacteria.o__Bifidobacteriales.f__Bifidobacteriaceae.g__Bifidobacterium.s__sp_.t__12_1_47BFAA
1.000
d__Bacteria.p__Actinobacteria.c__Actinobacteria.o__Bifidobacteriales.f__Bifidobacteriaceae.g__Bifidobacterium.s__longum.t__subsp__longum_F8
0.977
d__Bacteria.p__Actinobacteria.c__Actinobacteria.o__Bifidobacteriales.f__Bifidobacteriaceae.g__Bifidobacterium.s__longum.t__DJO10A
0.996
d__Bacteria.p__Actinobacteria.c__Actinobacteria.o__Bifidobacteriales.f__Bifidobacteriaceae.g__Bifidobacterium.s__longum.t__DJO10A
d__Bacteria.p__Actinobacteria.c__Actinobacteria.o__Bifidobacteriales.f__Bifidobacteriaceae.g__Bifidobacterium.s__longum.t__subsp__longum_BBMN68
1.000 0.160
0.929
d__Bacteria.p__Actinobacteria.c__Actinobacteria.o__Bifidobacteriales.f__Bifidobacteriaceae.g__Bifidobacterium.s__longum.t__subsp__longum_JCM_1217
0.946
d__Bacteria.p__Actinobacteria.c__Actinobacteria.o__Bifidobacteriales.f__Bifidobacteriaceae.g__Bifidobacterium.s__longum.t__subsp__infantis_ATCC_55813
d__Bacteria.p__Actinobacteria.c__Actinobacteria.o__Bifidobacteriales.f__Bifidobacteriaceae.g__Bifidobacterium.s__longum.t__subsp__longum_KACC_91563
0.929
d__Bacteria.p__Actinobacteria.c__Actinobacteria.o__Bifidobacteriales.f__Bifidobacteriaceae.g__Bifidobacterium.s__longum.t__subsp__infantis_CCUG_52486
d__Bacteria.p__Actinobacteria.c__Actinobacteria.o__Bifidobacteriales.f__Bifidobacteriaceae.g__Bifidobacterium.s__longum.t__subsp__infantis_ATCC_15697
1.000
d__Bacteria.p__Actinobacteria.c__Actinobacteria.o__Bifidobacteriales.f__Bifidobacteriaceae.g__Bifidobacterium.s__longum.t__subsp__infantis_ATCC_15697
d__Bacteria.p__Actinobacteria.c__Actinobacteria.o__Bifidobacteriales.f__Bifidobacteriaceae.g__Bifidobacterium.s__angulatum.t__DSM_20098
d__Bacteria.p__Actinobacteria.c__Actinobacteria.o__Bifidobacteriales.f__Bifidobacteriaceae.g__Bifidobacterium.s__dentium.t__ATCC_27679
0.999
0.897
d__Bacteria.p__Actinobacteria.c__Actinobacteria.o__Bifidobacteriales.f__Bifidobacteriaceae.g__Bifidobacterium.s__dentium.t__JCVIHMP022
1.000
0.972
d__Bacteria.p__Actinobacteria.c__Actinobacteria.o__Bifidobacteriales.f__Bifidobacteriaceae.g__Bifidobacterium.s__dentium.t__Bd1
0.942
d__Bacteria.p__Actinobacteria.c__Actinobacteria.o__Bifidobacteriales.f__Bifidobacteriaceae.g__Bifidobacterium.s__dentium.t__ATCC_27678
1.000
1.000
d__Bacteria.p__Actinobacteria.c__Actinobacteria.o__Bifidobacteriales.f__Bifidobacteriaceae.g__Bifidobacterium.s__adolescentis.t__ATCC_15703
1.000
d__Bacteria.p__Actinobacteria.c__Actinobacteria.o__Bifidobacteriales.f__Bifidobacteriaceae.g__Bifidobacterium.s__adolescentis.t__L2_32
0.418
d__Bacteria.p__Actinobacteria.c__Actinobacteria.o__Bifidobacteriales.f__Bifidobacteriaceae.g__Bifidobacterium.s__pseudocatenulatum.t__DSM_20438
1.000
d__Bacteria.p__Actinobacteria.c__Actinobacteria.o__Bifidobacteriales.f__Bifidobacteriaceae.g__Bifidobacterium.s__catenulatum.t__DSM_16992
d__Bacteria.p__Actinobacteria.c__Actinobacteria.o__Bifidobacteriales.f__Bifidobacteriaceae.g__Bifidobacterium.s__gallicum.t__DSM_20093
d__Bacteria.p__Actinobacteria.c__Actinobacteria.o__Bifidobacteriales.f__Bifidobacteriaceae.g__Bifidobacterium.s__animalis.t__subsp__lactis_Bl_04
d__Bacteria.p__Actinobacteria.c__Actinobacteria.o__Bifidobacteriales.f__Bifidobacteriaceae.g__Bifidobacterium.s__animalis.t__subsp__lactis_HN019
d__Bacteria.p__Actinobacteria.c__Actinobacteria.o__Bifidobacteriales.f__Bifidobacteriaceae.g__Bifidobacterium.s__animalis.t__subsp__lactis_BB_12
0.999
d__Bacteria.p__Actinobacteria.c__Actinobacteria.o__Bifidobacteriales.f__Bifidobacteriaceae.g__Bifidobacterium.s__animalis.t__subsp__lactis_CNCM_I_2494
0.993
d__Bacteria.p__Actinobacteria.c__Actinobacteria.o__Bifidobacteriales.f__Bifidobacteriaceae.g__Bifidobacterium.s__animalis.t__subsp__lactis_AD011
d__Bacteria.p__Actinobacteria.c__Actinobacteria.o__Bifidobacteriales.f__Bifidobacteriaceae.g__Bifidobacterium.s__animalis.t__subsp__lactis_V9
d__Bacteria.p__Actinobacteria.c__Actinobacteria.o__Bifidobacteriales.f__Bifidobacteriaceae.g__Bifidobacterium.s__animalis.t__subsp__lactis_DSM_10140
d__Bacteria.p__Actinobacteria.c__Actinobacteria.o__Actinomycetales.f__Actinomycetaceae.g__Actinomyces.s__sp_.t__oral_taxon_848_str__F0332
1.000
d__Bacteria.p__Actinobacteria.c__Actinobacteria.o__Actinomycetales.f__Actinomycetaceae.g__Arcanobacterium.s__haemolyticum.t__DSM_20595
1.000
d__Bacteria.p__Actinobacteria.c__Actinobacteria.o__Actinomycetales.f__Actinomycetaceae.g__Actinomyces.s__sp_.t__oral_taxon_170_str__F0386
0.985
d__Bacteria.p__Actinobacteria.c__Actinobacteria.o__Actinomycetales.f__Actinomycetaceae.g__Actinomyces.s__sp_.t__oral_taxon_171_str__F0337
d__Bacteria.p__Actinobacteria.c__Actinobacteria.o__Actinomycetales.f__Actinomycetaceae.g__Actinomyces.s__viscosus.t__C505
0.997
0.993
d__Bacteria.p__Actinobacteria.c__Actinobacteria.o__Actinomycetales.f__Actinomycetaceae.g__Actinomyces.s__oris.t__K20
d__Bacteria.p__Actinobacteria.c__Actinobacteria.o__Actinomycetales.f__Actinomycetaceae.g__Actinomyces.s__urogenitalis.t__DSM_15434
0.500
d__Bacteria.p__Actinobacteria.c__Actinobacteria.o__Actinomycetales.f__Actinomycetaceae.g__Actinomyces.s__sp_.t__oral_taxon_448_str__F0400
d__Bacteria.p__Actinobacteria.c__Actinobacteria.o__Actinomycetales.f__Actinomycetaceae.g__Mobiluncus.s__mulieris.t__ATCC_35239
0.976
d__Bacteria.p__Actinobacteria.c__Actinobacteria.o__Actinomycetales.f__Actinomycetaceae.g__Mobiluncus.s__mulieris.t__ATCC_35243
0.981
d__Bacteria.p__Actinobacteria.c__Actinobacteria.o__Actinomycetales.f__Actinomycetaceae.g__Mobiluncus.s__mulieris.t__28_1
1.000
d__Bacteria.p__Actinobacteria.c__Actinobacteria.o__Actinomycetales.f__Actinomycetaceae.g__Mobiluncus.s__mulieris.t__FB024_16
d__Bacteria.p__Actinobacteria.c__Actinobacteria.o__Actinomycetales.f__Actinomycetaceae.g__Mobiluncus.s__curtisii.t__ATCC_51333
d__Bacteria.p__Actinobacteria.c__Actinobacteria.o__Actinomycetales.f__Actinomycetaceae.g__Mobiluncus.s__curtisii.t__subsp__holmesii_ATCC_35242
0.996
d__Bacteria.p__Actinobacteria.c__Actinobacteria.o__Actinomycetales.f__Actinomycetaceae.g__Mobiluncus.s__curtisii.t__subsp__curtisii_ATCC_35241
0.800
d__Bacteria.p__Actinobacteria.c__Actinobacteria.o__Actinomycetales.f__Actinomycetaceae.g__Mobiluncus.s__curtisii.t__ATCC_43063
d__Bacteria.p__Actinobacteria.c__Actinobacteria.o__Actinomycetales.f__Actinomycetaceae.g__Actinomyces.s__coleocanis.t__DSM_15436
1.000
d__Bacteria.p__Actinobacteria.c__Actinobacteria.o__Actinomycetales.f__Actinomycetaceae.g__Actinomyces.s__sp_.t__oral_taxon_178_str__F0338
d__Bacteria.p__Actinobacteria.c__Actinobacteria.o__Actinomycetales.f__Actinomycetaceae.g__Actinomyces.s__sp_.t__oral_taxon_180_str__F0310
d__Bacteria.p__Actinobacteria.c__Actinobacteria.o__Actinomycetales.f__Actinomycetaceae.g__Actinomyces.s__odontolyticus.t__F0309
1.000
d__Bacteria.p__Actinobacteria.c__Actinobacteria.o__Actinomycetales.f__Actinomycetaceae.g__Actinomyces.s__odontolyticus.t__ATCC_17982
d__Bacteria.p__Actinobacteria.c__Actinobacteria.o__Actinomycetales.f__Dermabacteraceae.g__Brachybacterium.s__faecium.t__DSM_4810
1.000

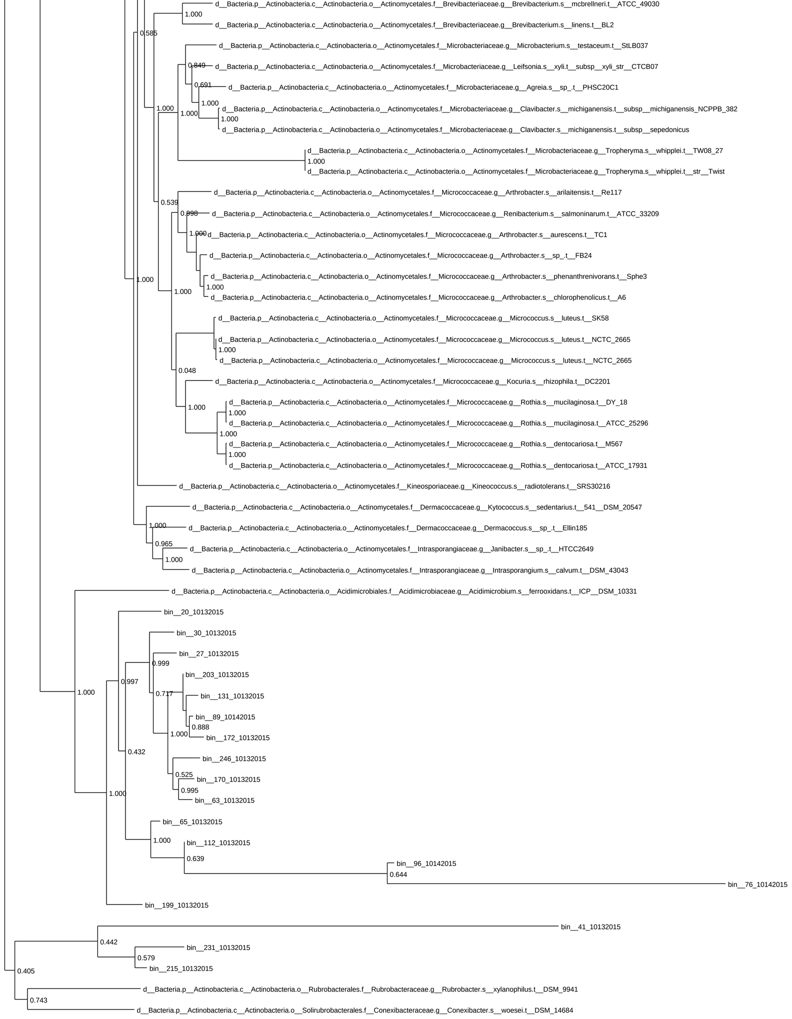
d__Bacteria.p__Actinobacteria.c__Actinobacteria.o__Actinomycetales.f__Brevibacteriaceae.g__Brevibacterium.s__mcbrellneri.t__ATCC_49030
1.000
d__Bacteria.p__Actinobacteria.c__Actinobacteria.o__Actinomycetales.f__Brevibacteriaceae.g__Brevibacterium.s__linens.t__BL2
0.585
d__Bacteria.p__Actinobacteria.c__Actinobacteria.o__Actinomycetales.f__Microbacteriaceae.g__Microbacterium.s__testaceum.t__StLB037
0.849
d__Bacteria.p__Actinobacteria.c__Actinobacteria.o__Actinomycetales.f__Microbacteriaceae.g__Leifsonia.s__xyli.t__subsp__xyli_str__CTCB07
0.691
d__Bacteria.p__Actinobacteria.c__Actinobacteria.o__Actinomycetales.f__Microbacteriaceae.g__Agreia.s__sp_.t__PHSC20C1
1.000
1.000
1.000
d__Bacteria.p__Actinobacteria.c__Actinobacteria.o__Actinomycetales.f__Microbacteriaceae.g__Clavibacter.s__michiganensis.t__subsp__michiganensis_NCPPB_382
1.000
d__Bacteria.p__Actinobacteria.c__Actinobacteria.o__Actinomycetales.f__Microbacteriaceae.g__Clavibacter.s__michiganensis.t__subsp__sepedonicus
d__Bacteria.p__Actinobacteria.c__Actinobacteria.o__Actinomycetales.f__Microbacteriaceae.g__Tropheryma.s__whipplei.t__TW08_27
1.000
d__Bacteria.p__Actinobacteria.c__Actinobacteria.o__Actinomycetales.f__Microbacteriaceae.g__Tropheryma.s__whipplei.t__str__Twist
d__Bacteria.p__Actinobacteria.c__Actinobacteria.o__Actinomycetales.f__Micrococcaceae.g__Arthrobacter.s__arilaitensis.t__Re117
0.539
0.898
d__Bacteria.p__Actinobacteria.c__Actinobacteria.o__Actinomycetales.f__Micrococcaceae.g__Renibacterium.s__salmoninarum.t__ATCC_33209
1.000
d__Bacteria.p__Actinobacteria.c__Actinobacteria.o__Actinomycetales.f__Micrococcaceae.g__Arthrobacter.s__aurescens.t__TC1
d__Bacteria.p__Actinobacteria.c__Actinobacteria.o__Actinomycetales.f__Micrococcaceae.g__Arthrobacter.s__sp_.t__FB24
d__Bacteria.p__Actinobacteria.c__Actinobacteria.o__Actinomycetales.f__Micrococcaceae.g__Arthrobacter.s__phenanthrenivorans.t__Sphe3
1.000
1.000
1.000
d__Bacteria.p__Actinobacteria.c__Actinobacteria.o__Actinomycetales.f__Micrococcaceae.g__Arthrobacter.s__chlorophenolicus.t__A6
d__Bacteria.p__Actinobacteria.c__Actinobacteria.o__Actinomycetales.f__Micrococcaceae.g__Micrococcus.s__luteus.t__SK58
d__Bacteria.p__Actinobacteria.c__Actinobacteria.o__Actinomycetales.f__Micrococcaceae.g__Micrococcus.s__luteus.t__NCTC_2665
1.000
d__Bacteria.p__Actinobacteria.c__Actinobacteria.o__Actinomycetales.f__Micrococcaceae.g__Micrococcus.s__luteus.t__NCTC_2665
0.048
d__Bacteria.p__Actinobacteria.c__Actinobacteria.o__Actinomycetales.f__Micrococcaceae.g__Kocuria.s__rhizophila.t__DC2201
1.000
d__Bacteria.p__Actinobacteria.c__Actinobacteria.o__Actinomycetales.f__Micrococcaceae.g__Rothia.s__mucilaginosa.t__DY_18
1.000
d__Bacteria.p__Actinobacteria.c__Actinobacteria.o__Actinomycetales.f__Micrococcaceae.g__Rothia.s__mucilaginosa.t__ATCC_25296
1.000
d__Bacteria.p__Actinobacteria.c__Actinobacteria.o__Actinomycetales.f__Micrococcaceae.g__Rothia.s__dentocariosa.t__M567
1.000
d__Bacteria.p__Actinobacteria.c__Actinobacteria.o__Actinomycetales.f__Micrococcaceae.g__Rothia.s__dentocariosa.t__ATCC_17931
d__Bacteria.p__Actinobacteria.c__Actinobacteria.o__Actinomycetales.f__Kineosporiaceae.g__Kineococcus.s__radiotolerans.t__SRS30216
d__Bacteria.p__Actinobacteria.c__Actinobacteria.o__Actinomycetales.f__Dermacoccaceae.g__Kytococcus.s__sedentarius.t__541__DSM_20547
1.000
d__Bacteria.p__Actinobacteria.c__Actinobacteria.o__Actinomycetales.f__Dermacoccaceae.g__Dermacoccus.s__sp_.t__Ellin185
0.965
d__Bacteria.p__Actinobacteria.c__Actinobacteria.o__Actinomycetales.f__Intrasporangiaceae.g__Janibacter.s__sp_.t__HTCC2649
1.000
d__Bacteria.p__Actinobacteria.c__Actinobacteria.o__Actinomycetales.f__Intrasporangiaceae.g__Intrasporangium.s__calvum.t__DSM_43043
d__Bacteria.p__Actinobacteria.c__Actinobacteria.o__Acidimicrobiales.f__Acidimicrobiaceae.g__Acidimicrobium.s__ferrooxidans.t__ICP__DSM_10331
bin__20_10132015
bin__30_10132015
bin__27_10132015
0.999
bin__203_10132015
0.997
1.000
0.717
bin__131_10132015
bin__89_10142015
0.888
1.000
bin__172_10132015
0.432
bin__246_10132015
0.525
bin__170_10132015
0.995
1.000
bin__63_10132015
bin__65_10132015
1.000
bin__112_10132015
0.639
bin__96_10142015
0.644
bin__76_10142015
bin__199_10132015
bin__41_10132015
0.442
bin__231_10132015
0.579
bin__215_10132015
0.405
d__Bacteria.p__Actinobacteria.c__Actinobacteria.o__Rubrobacterales.f__Rubrobacteraceae.g__Rubrobacter.s__xylanophilus.t__DSM_9941
0.743
d__Bacteria.p__Actinobacteria.c__Actinobacteria.o__Solirubrobacterales.f__Conexibacteraceae.g__Conexibacter.s__woesei.t__DSM_14684
